# Differentiation State-Specific Mitochondrial Dynamic Regulatory Networks Are Revealed by Global Transcriptional Analysis of the Developing Chicken Lens

**DOI:** 10.1534/g3.114.012120

**Published:** 2014-06-13

**Authors:** Daniel Chauss, Subhasree Basu, Suren Rajakaruna, Zhiwei Ma, Victoria Gau, Sara Anastas, Lisa A. Brennan, J. Fielding Hejtmancik, A. Sue Menko, Marc Kantorow

**Affiliations:** * Department of Biomedical Science, Florida Atlantic University, Boca Raton, Florida 33431; † Department of Pathology, Anatomy and Cell Biology, Thomas Jefferson University, Philadelphia, Pennsylvania 19107; ‡ Ophthalmic Genetics and Visual Function Branch, National Eye Institute, National Institutes of Health, Bethesda, Maryland 20892

**Keywords:** RNA sequencing, eye, lens, differentiation, mitochondria, mitophagy, mitochondrial dynamics

## Abstract

The mature eye lens contains a surface layer of epithelial cells called the lens epithelium that requires a functional mitochondrial population to maintain the homeostasis and transparency of the entire lens. The lens epithelium overlies a core of terminally differentiated fiber cells that must degrade their mitochondria to achieve lens transparency. These distinct mitochondrial populations make the lens a useful model system to identify those genes that regulate the balance between mitochondrial homeostasis and elimination. Here we used an RNA sequencing and bioinformatics approach to identify the transcript levels of all genes expressed by distinct regions of the lens epithelium and maturing fiber cells of the embryonic *Gallus gallus* (chicken) lens. Our analysis detected more than 15,000 unique transcripts expressed by the embryonic chicken lens. Of these, more than 3000 transcripts exhibited significant differences in expression between lens epithelial cells and fiber cells. Multiple transcripts coding for separate mitochondrial homeostatic and degradation mechanisms were identified to exhibit preferred patterns of expression in lens epithelial cells that require mitochondria relative to lens fiber cells that require mitochondrial elimination. These included differences in the expression levels of metabolic (DUT, PDK1, SNPH), autophagy (ATG3, ATG4B, BECN1, FYCO1, WIPI1), and mitophagy (BNIP3L/NIX, BNIP3, PARK2, p62/SQSTM1) transcripts between lens epithelial cells and lens fiber cells. These data provide a comprehensive window into all genes transcribed by the lens and those mitochondrial regulatory and degradation pathways that function to maintain mitochondrial populations in the lens epithelium and to eliminate mitochondria in maturing lens fiber cells.

The vertebrate eye lens functions to focus light onto the retina, where visual signals are processed and ultimately transmitted to the brain (Bassnett *et al.* 2011). The lens consists of an anterior layer of cuboidal mitochondrial and organelle-containing epithelial cells that overlie a core of elongated organelle-free fiber cells (Rabl 1899; Cohen 1965; Bassnett 2009). Lens epithelial cells located at the equator of the lens undergo cell-cycle exit, elongation, and loss of mitochondria and other organelles to form mature lens fibers cells during embryogenesis and throughout the life of the lens (Piatigorsky 1981). Lens epithelial cell mitochondrial function is required for the homeostasis of the entire lens (Bloemendal 1981; Brown and Bron 1996; Bantseev *et al.* 1999; Brennan and Kantorow 2009; Delamere and Tamiya 2009).

Lens epithelial cell mitochondria are abundant (Bassnett and Beebe 1992) and metabolically active (Weber and Menko 2005; Basu *et al.* 2014a), consistent with the function of the lens epithelium in a wide range of lens processes ranging from ion exchange to protein synthesis (Bloemendal 1981; Brown and Bron 1996; Bantseev *et al.* 1999; Brennan and Kantorow 2009; Delamere and Tamiya 2009). In contrast to the mitochondrial population in the lens epithelium that is required for lens homeostasis, mitochondria are completely eliminated from lens fiber cells upon their maturation. During lens fiber cell maturation, mitochondria lose their membrane potential (Weber and Menko 2005; Basu *et al.* 2014a), fragment (Bassnett and Beebe 1992; Zandy and Bassnett 2007), and are ultimately degraded by mitophagy (Costello *et al.* 2013; Basu *et al.* 2014b; Frost *et al.* 2014). Mitophagy is the selective sequestration and degradation of mitochondria using the autophagy machinery (for review, see: Youle and Narendra 2011; Wang and Klionsky 2011; Ding and Yin 2012; Ashrafi and Schwarz 2013; Randow and Youle 2014). Mitophagy is directed by distinct regulatory proteins and pathways, including the PARK2/Parkin pathway, which targets damaged mitochondria for degradation (Randow and Youle 2014). In this pathway, cytosolic Parkin is phosphorylated by the mitochondrial protein phosphatase and tensin homolog−induced putative kinase 1 (PINK1) that accumulates on the outer membrane of damaged mitochondria (Randow and Youle 2014). Upon Parkin phosphorylation, Parkin ubiquitinates outer mitochondrial membrane proteins and broadly activates the ubiquitin-proteasome system (Randow and Youle 2014). These ubiquitinated proteins are then degraded by the ubiquitin-proteasome system or used as substrates for targeting by selective macroautophagy adaptor proteins such as sequestosome 1 (P62/SQSTM1) (Randow and Youle 2014). In addition to the Parkin pathway, a separate, Parkin-independent form of mitophagy has been identified that uses BCL2/adenovirus E1B interacting protein 3-like (BNIP3L/NIX) (Zhang and Ney 2009; Randow and Youle 2014). This pathway eliminates mitochondria in mammalian erythrocytes by disrupting mitochondrial membrane potential and directly recruiting microtubule-associated protein 1 light chain 3 beta homologs to the mitochondria via an LC3-interacting region motif (Sandoval *et al.* 2008; Zhang and Ney 2009; Kanki 2010; Novak *et al.* 2010; Birgisdottir *et al.* 2013).

The opposing mitochondrial requirements of lens epithelial cells and lens fiber cells suggest that the Parkin, NIX, or other distinct mitochondrial regulatory and degradation pathways operate in the separate compartments of the eye lens. Because the lens is composed primarily of lens epithelial cells and fiber cells, it provides a unique way of identifying mitochondrial regulatory and degradation pathways that could govern the maintenance of mitochondrial populations under different cellular metabolic requirements and the elimination of mitochondria under different states of cellular differentiation. Identifying these mitochondrial pathways is important because loss of lens epithelial cell mitochondria function (Bantseev *et al.* 1999; Lou 2003; Kantorow *et al.* 2004; Brennan *et al.* 2009b; Wu *et al.* 2011) or failure to eliminate mitochondria during lens fiber cell differentiation (Pendergrass *et al.* 2005; Zandy and Bassnett 2007) results in eye lens cataract formation (for review, see: Shiels and Hejtmancik 2013).

To date, those mitochondrial regulatory mechanisms and pathways that maintain the homeostasis of mitochondria in the lens epithelium and eliminate mitochondria in lens fiber cells have not been fully elucidated. It has been demonstrated that multiple mitophagy genes are expressed throughout the human lens (Brennan *et al.* 2012) and that lens epithelial cells respond to stress through induction of mitophagy (Costello *et al.* 2013). Importantly, it has recently been demonstrated that induction of autophagy drives early differentiation and organelle degradation in the lens including the early degradation of mitochondria (Basu *et al.* 2014b). Collectively, these data support the existence of specific mitochondrial regulatory and degradation pathways operating in functionally distinct regions of the lens.

Here, we used high-throughput mRNA-sequencing and bioinformatics analysis to identify the entire transcriptional complement of genes expressed in specific regions of differentiation of the *Gallus gallus* (chicken) eye lens, including the lens epithelial cells with active mitochondrial populations and the differentiating lens fiber cells that are in the process of eliminating their mitochondria. This approach enabled us to identify the entire complement of transcripts expressed by these regions of the lens and the full range and spectrum of mitochondrial-associated transcripts expressed by these regions. Our analysis identified more than 3000 differentially expressed transcripts between these lens regions, including the differential expression of multiple mitochondrial regulatory and degradation transcripts that point to specific mitochondrial pathways operating in these regions. The data provide insight into the specific mitochondrial regulatory and degradation pathways operating to maintain functional mitochondrial populations in the lens epithelium and eliminate mitochondria upon lens fiber cell maturation.

## Materials and Methods

### Microdissection of embryonic chicken lenses

**Figure 1:**
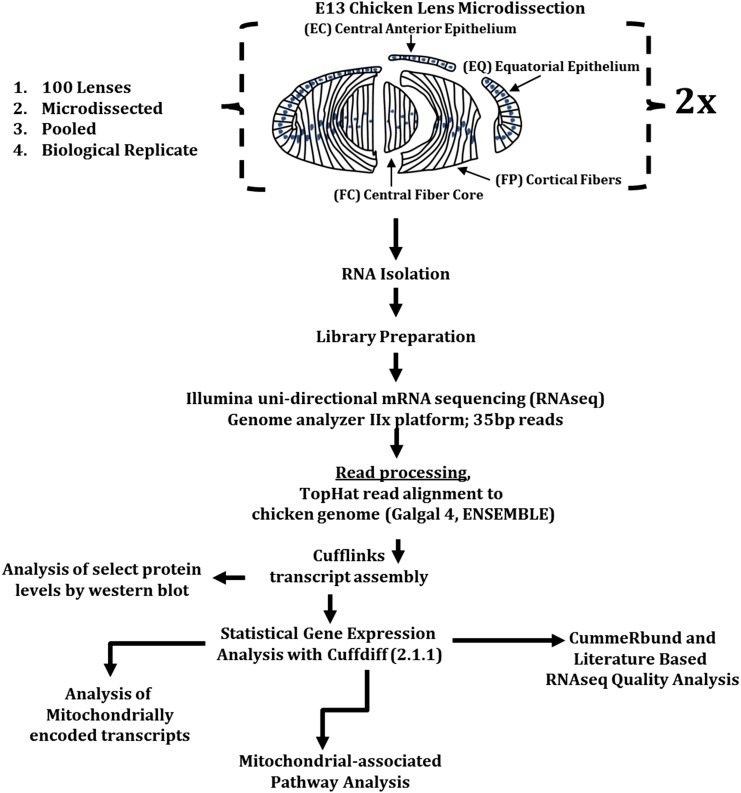
Lens microdissection, RNA isolation, RNA-sequencing, and data analysis. Lens microdissections were performed as described by Walker and Menko (1999). Two sets (N = 2) of pooled (n = 100) lens differentiation-state specific fractions were subjected to RNA isolation using Trizol and Illumina directional mRNAseq library preparation performed. High-throughput sequencing using the Illumina GAIIx platform with 35-bp unidirectional reads generated millions of sequencing reads that were processed, aligned by Tophat to Galgal4 and biological replicate based statistical modeling and transcript abundance and identity assembled by cufflinks using the maximum transcript abundance likelihood estimate model described by Trapnell *et al.* (2010). Statistical testing was performed using cuffdiff pairwise statistical gene expression analysis. CummeRbund was used to statistically assess data and literature searches were performed to verify expression results. Ontologically based pathway analysis was performed using DAVID and GenoMatix software packages. Gene clustering was performed that placed mitochondrial-associated transcripts into mitochondrial regulation, biogenesis, homeostasis or degradation functional clusters.

Fertilized chicken eggs (B&E Eggs, York Springs, PA) were incubated to embryonic day 13 (E13) at 33.67° in a humidified incubator with automated rotation (GQF Manufacturing Company Inc., Savannah, GA). Differentiation-state analysis of embryonic chicken lenses was performed after microdissection of 100 E13 chicken lenses into four distinct regions (Figure 1) that represent a continuum of lens cell differentiation states: lens central epithelium (EC), equatorial epithelium (EQ), cortical fibers (FP), and central fibers (FC) as described previously (Walker and Menko 1999). Further analysis of the transcriptional content of these samples was performed by RNA sequencing (Figure 1).

### High-throughput RNA sequencing of pooled microdissected chicken lenses

**Figure 2:**
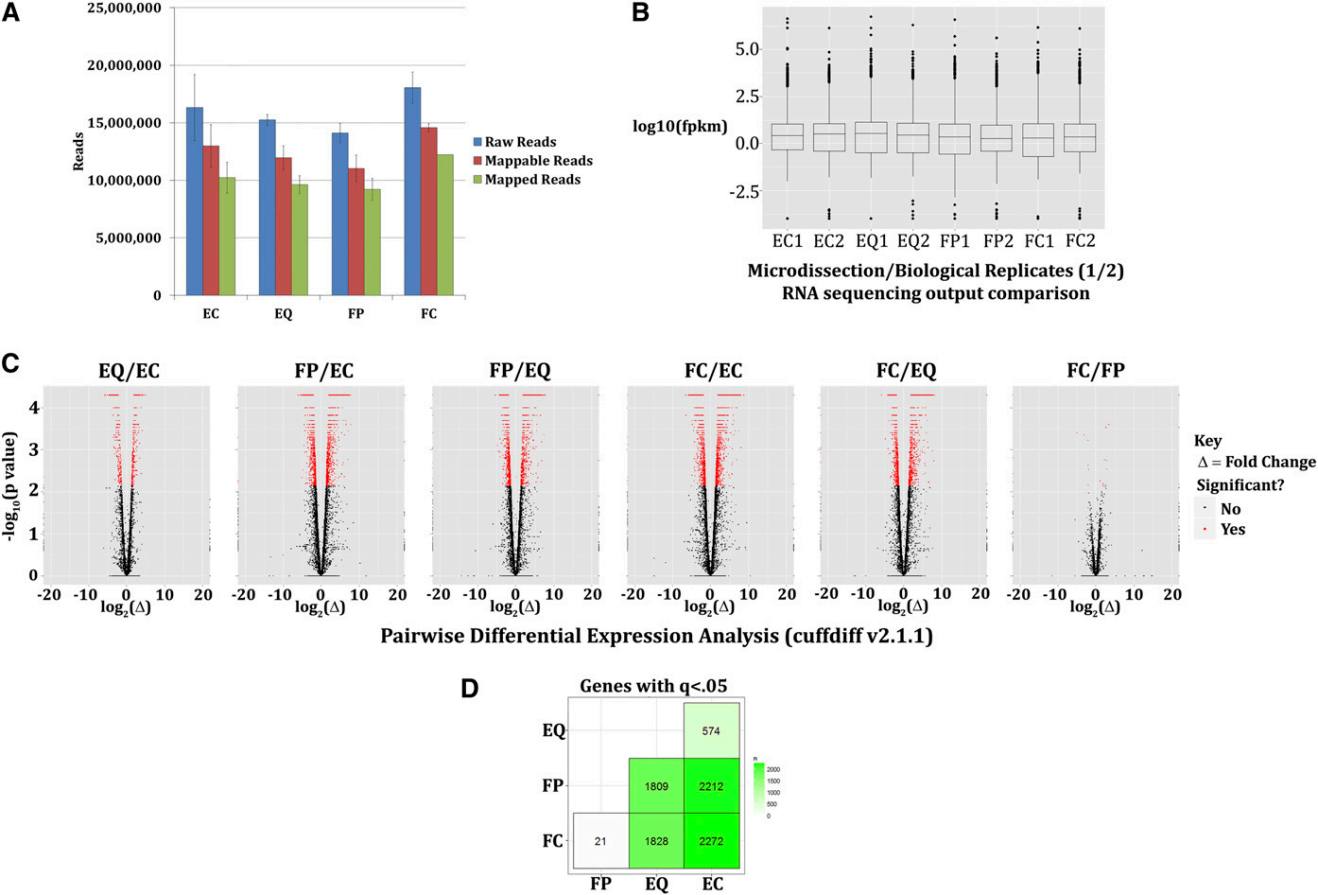
Identification of differentially expressed transcripts. RNA sequencing of microdissected E13 embryonic chicken lenses revealed the expression of more than 16,000 genes, with 3000 (false discovery rate adjusted *P* < 0.05, termed q < 0.05) genes displaying differential expression between lens cell differentiation-state specific zones (lens central epithelium [EC], equatorial epithelium [EQ], cortical fibers [FP], and central fibers [FC]). (A) Reads generated by Illumina mRNA-sequencing and mapping of reads to the Galgal4 chicken genome (ENSEMBLE). (B) Boxplot analysis of the sum estimate abundance (FPKM) between samples to demonstrate sample skew revealed little to no skew between samples. (C) Differential expression analysis shows differentially expressed genes between each embryonic lens region in pairwise comparison as demonstrated by volcano plot analysis (red indicates differentially expressed). (D) Sum of nonunique differentially expressed gene-specific transcripts between lens differentiation state−specific zones (Table S1, Table S2, Table S3, Table S4, Table S5, Table S6, and File S1). FPKM, fragments per kilobase of exon per million fragments mapped.

Chicken lenses were microdissected (n = 100) into the regions described previously, 100 regions were pooled, and total RNA prepared for each sample by established protocols (Trizol; Invitrogen, Carlsbad, CA). Two independent pools of total RNA from 100 microdissected lenses were used for RNA sequencing analysis as biological replicates. Total RNA was analyzed for quality and subjected to Illumina mRNA directional sequencing library preparation (Illumina, San Diego, CA). Total RNA also was analyzed for quality upon completion of library preparation using the Agilent Technologies 2100 Expert Bioanalyzer (Santa Clara, CA). Prepared libraries were then sequenced unidirectionally with the Genome Analyzer IIx as short 35-bp reads. Sequenced reads were considered mappable reads after >10 bp sequence remained following removal of the 3′ adaptor sequence (TGGAATTCTCGGGTGCCAAGG) (Figure 2A, Supporting Information, File S1, raw reads located at GEO ascension no. GSE53976).

Mappable reads were assembled and mapped with TopHat (Trapnell *et al.* 2010) to the reference chicken genome (Galgal4; ENSEMBLE, Galgal4, GCA_000002315.2; http://useast.ensembl.org/Gallus_gallus/Info/Index). The TopHat output was directly processed by cufflinks under default parameters unless otherwise noted. In cufflinks, per sequenced sample, transcript identity and abundance were statistically estimated and computed as expected number of fragments per kilobase of exon per million fragments mapped (FPKM) (Trapnell *et al.* 2010, 2012). FPKM is an estimate of transcriptional abundance estimated by the approximately linear maximum abundance likelihood model, taking into account stochastic sequencing biases, described in detail by Trapnell *et al.* (2010). 

To summarize, in this model, assigned fragment abundances are defined as asymptotically multivariate normal and an accompanying variance-covariance matrix is obtained from the inverse of the Fisher information likelihood matrix. The fragment abundances are subsequently converted into transcript abundances using the Lemma 14 model as explained by Trapnell *et al.* (2010). FPKMs are estimated at the 95% confidence interval and are proportional to relative transcript levels after adjustment using a scalar calculation (Trapnell *et al.* 2010). A boxplot generated using cummeRbund of the log FPKM between samples and sample replicates displayed no grossly detectable library bias toward any one sample or sample replicate (Figure 2B). Statistical analysis was performed by the cuffdiff (v2.1.1) module of cufflinks that takes into consideration replicate data and sequencing biases (Trapnell *et al.* 2010). p-values are adjusted for multiple testing using the Benjamini-Hochberg false discovery rate method as described by Trapnell *et al.* (2010) (Table S1, Table S2, Table S3, Table S4, Table S5, and Table S6). The resulting cuffdiff output that includes transcript FPKM and differential expression tracking files are provided as File S1 and the resulting differential expression output and raw sequencing reads can also be found at GEO ascension no. GSE53976.

### Western analysis of select proteins

Microdissected tissue samples were extracted in Triton/octylglucoside buffer (44.4 mM *n*-octyl β-D-glucopyranoside, 1% Triton X-100, 100 mM NaCl, 1 mM MgCl_2_, 5mM ethylenediaminetetraacetic acid, and 10 mM imidazole) containing 1mM sodium vanadate, 0.2 mM H_2_O_2_, and protease inhibitor Cocktail (Sigma-Aldrich, St. Louis, MO). The protein concentration was determined with the BCA assay (Thermo Fisher Scientific, Inc., Waltham, MA). A total of 50 μg of protein from each sample was subjected to sodium dodecyl sulfate polyacrylamide gel electrophoresis on precast 8–16% Tris/glycine (Novex, San Diego, CA). Proteins were electrophoretically transferred onto Immobilon-P (polyvinylidene difluoride) membranes (Millipore, Billerica, MA) and membranes were blocked in 5% skim milk for 1 hr. Membranes were probed for primary antibody followed by secondary antibody-conjugated to horseradish peroxidase (Bio-Rad Laboratories Inc., Hercules, CA). Protein bands were detected using ECL reagent or ECL Plus reagent (Thermo Fisher Scientific, Inc.). Images of immunoblots were acquired using the FluorChem E & M imager from Protein Simple (#FM0418), a digital darkroom technology. Antibodies used for immunblotting included BFSP1 (a gift from Paul Fitzgerald, PhD, UC Davis, Davis, CA), CP49/BFSP2 (a gift from Paul Fitzgerald, PhD), RB1CC1/FIP200 (cat. no. A301-536A; Bethyl Laboratories, Montgomery, TX), FYCO1 (cat no. ab126603; Abcam, Cambridge, UK), mTOR (cat. no. 2983; Cell Signaling, Boston, MA), mitofusin (MFN)1 (cat no. ABC41; Millipore), MFN2 (cat. no. ABC42; Millipore), RAB9 (ab2810; Abcam), BNIP3L (cat no. ADI-905-185; Enzo Life Sciences, Farmingdale, NY), GATE16 (cat. no. PMO38; MBL International Corporation, Woburn, MA), PARK2/Parkin (cat. no. sc-30130; Santa Cruz Biotechnology, Dallas, TX), BECN1 (cat. no. 3495; Cell Signaling), GAPDH (cat. no. sc-25778; Santa Cruz Biotechnology), succinate dehydrogenase complex, subunit A (cat no. ab14715; Abcam), and translocase of the outer mitochondrial membrane 20 homolog (TOMM20: cat. no. sc-17764; Santa Cruz Biotechnology).

### Immunofluorescent analysis of developing chicken lenses

Freshly isolated embryonic day 10, 13, and 15 chicken lenses were fixed in 3.7% paraformaldehyde/phosphate-buffered saline (PBS) solution overnight at 4° and transferred to a 30% sucrose/PBS solution for cryopreservation. Lenses were prepared for cryosectioning by embedment in OCT compound, and 20-μm thick sections were cut in series in anterior to posterior orientation. Midsagittal lens sections were then permeabilized in 0.25% Triton-X buffer for 10 min, blocked for 1 hr in blocking buffer (5% goat serum, 0.5 g of bovine serum albumin in 50 mL of PBS), and then incubated overnight in primary antibody (antibodies described previously) diluted in blocking buffer at 4° followed by the addition of a fluorescent-conjugated secondary antibody (Jackson ImmunoResearch Laboratories, West Grove, PA) for 2 hr at 37°. Nuclei were counterstained with TO-PRO-3. Sections were washed with PBS 3 times between buffer incubations throughout the staining protocol. The Zeiss LSM510 META confocal microscope was used for imaging. Single optical planes were selected from z-stacks, each 1 μm thick, using the LSM5 Image Browser. No staining was observed using secondary antibody alone (data not shown).

### Mitochondrial dynamic pathway clustering of expressed transcripts and fold change (Δ) analysis

Assembly of nuclear-encoded mitochondrial regulatory transcripts was performed with the Pagliarini *et al.* (2008) human defined mitocarta as a template (Figure 6 and File S2). Assembly of genes involved in mitochondrial dynamic gene categories comprising mitochondrial fusion and fission, mitochondrial DNA replication and biogenesis regulation, mitochondrial repair and protection systems, macroautophagy initiation and nucleation regulation, autophagosome expansion and ATG conjugation, syntaxin/SNAREs [SNAP (Soluble NSF Attachment Protein) REceptor], selective macroautophagy (including mitophagy), autophagosome trafficking facilitation, and lysosomal fusion, lysosomal biogenesis, proteasome assembly, E1 ubiquitin activators, E2 ubiquitin conjugation enzymes, E3 ubiquitin ligases, ubiquitin, etc, and heat shock proteins70/40/27/22kDa also were analyzed using a combination of resources as a template, including the HUGO gene name consortium (www.genenames.org), NCBI tools, ENSEMBLE genome browser, by hand search of the literature, and autophagy genes determined largely based around the 2nd edition glossary of autophagy terms and processes (Klionsky *et al.* 2011) (Figure 7 and File S3).

Determination of lens epithelial (EC and EQ regions) or fiber cell (FP and FC regions) expression preference for a given nuclear encoded mitochondrial regulatory transcript was determined by standard fold change (Δ) analysis (Figure 6). The fold change of a given transcript (denoted as _(X)_) was calculated as the FPKM fold differences between the sum of the estimated FPKM per _(X)_ between FP plus FC divided by the sum of the estimated FPKM per _(X)_ between EC plus EQ [Transcript_(X)_ Δ = (ΣFPKM_(X)_(FP+FC)/ ΣFPKM_(X)_(EC+EQ))]. If the resulting number was greater than or equal to positive 2 [((ΣFPKM_(X)_(FP+FC)/ ΣFPKM_(X)_(EC+EQ))≥2] for a given _(X)_, the transcript was identified as fiber cell preferred; if the resulting negative inverse of the calculated fold difference for _(X)_ was less than or equal to negative 2 [−2≤-((ΣFPKM_(X)_(FP+FC))/ (ΣFPKM_(X)_(EC+EQ)))^-1^], the transcript was identified as lens epithelial cell preferred (Figure 6). Direct comparisons of EC with EQ or EQ with FP for transcript up-regulation or down-regulation during cellular transition for the analysis of nuclear encoded mitochondrial transcripts are presented in Table S7, Table S8, Table S9, and Table S10. Comparison of Δ between transitional zones was performed by dividing the estimated FPKM for a given _(X)_ in the region ahead in the continuum of differentiation (lens cell differentiation proceeds stepwise EC to EQ, EQ to FP, and FP to FC) of the estimated FPKM for the given _(X)_ from the region before in the continuum of differentiation. For example, the fold change for up-regulated genes during the EC to EQ transition was determined by dividing the estimated FPKM of _(X)_ detected in EQ divided by the estimated FPKM of _(X)_ detected in EC [Δ(x) = FPKM_(x)_(EQ)/FPKM_(x)_(EC)] that gives positive numbers (considered up-regulated at Δ ≥ 2) and decimals that are converted into negative numbers (considered down-regulated at Δ ≤ -2) by the negative inverse of the Δ(x) [Δ(x) = −(FPKM_(x)_(EQ)/FPKM_(x)_(EC))^-1^]. Down-regulated genes in some instances (Figure 7) are made non-negative through multiplication by negative 1 and are considered to be preferred to the region of the denominator in each respective fold change calculation.

## Results

### High-throughput RNA-sequencing of undifferentiated lens epithelial cells and differentiating lens fiber cells of the E13 chicken lens

To establish the spectrum and range of genes involved in mitochondrial regulation and degradation in undifferentiated lens epithelial cells relative to differentiating lens fiber cells, E13 chicken lenses were microdissected into four regions: EC (anterior central epithelium, contains mitochondria and is not differentiating), EQ (equatorial epithelium, contains mitochondria and is starting to differentiate), FP (cortical lens fiber cells, contains actively degrading mitochondria and differentiating lens fiber cells), and FC (maturing nuclear lens fiber cells that are eliminating mitochondria) (Walker and Menko 1999). These regions are diagrammed in Figure 1. The elimination of organelles from the FC region of the chicken lens begins at E12 and a transparent organelle-free zone (OFZ) is apparent by E15 (Bassnett and McNulty 2003; Costello *et al.* 2013; Basu *et al.* 2014b). The E13 stage of embryonic chicken lens development (E13) was chosen because this represents an early stage of OFZ formation when molecules required for removal of organelles would be expected to be up-regulated in the fiber cell zones. Therefore, E13 lenses provide a window in which gene expression of proteins responsible for both mitochondrial homeostasis in lens epithelial cells and mitochondrial elimination in lens fiber cells can be detected. 

Mitochondria degradation precedes much of the rest of organelle degradation in the developing lens (Costello *et al.* 2013; Basu *et al.* 2014b). In the E13 chicken lens, the EC and EQ zones have large numbers of functional mitochondria whereas cells in the FP zone are actively degrading their mitochondria during these initial states of differentiation and cells in the FC zone are actively eliminating their mitochondria as the OFZ begins to form (Bassnett and McNulty 2003). Two separate pools of total RNA were isolated from each of the EC, EQ, FP, and FC regions microdissected from 100 lenses, and the resulting total RNA preparations were subjected to Illumina mRNA directional library preparation followed by high-throughput RNA-sequencing. The resulting 35-bp single-ended reads were analyzed using the tuxedo protocol (Trapnell *et al.* 2010) with the Galgal4 annotated chicken genome assembly. A schematic of these procedures is summarized in Figure 1. A total of more than 15,000 gene-specific transcripts were identified to be expressed by the E13 chicken lens by cufflinks analysis (Figure 2; see cufflinks tab-delineated file: genes.fpkm_tracking; File S1). Approximately 10−15 million reads were generated per microdissected lens area after read filtering (Figure 2A). A comparison of total FPKMs between two biological replicates revealed agreement within replicates and between individual samples (Figure 2B). Cuffdiff analysis identified more than 3000 unique gene-level (see cufflinks tab-delineated file: genes.tracking.diff; File S1) transcripts exhibiting significantly different (false discovery rate adjusted *P*-value < 0.05, or termed q < 0.05) expression levels between the examined lens subregions (Figure 2C) and differentially expressed genes were manually identified (Figure 2D, File S1, Table S1, Table S2, Table S3, Table S4, Table S5, and Table S6). Of these, 574 gene-specific transcript differences were detected between EC and EQ (Figure 2D), 2212 gene-specific transcript differences were detected between EC and FP (Figure 2D), 2272 gene-specific transcript differences were detected between EC and FC (Figure 2D), 1809 gene-specific transcript differences were detected between EQ and FP (Figure 2D), 1828 gene-specific transcript differences were detected between EQ and FC (Figure 2D), and only 21 gene-specific transcript differences were detected between FP and FC (Figure 2D). The amount of unique gene-specific transcripts for each specific transition compared with other transitions were not specifically examined. On the basis of the limited gene expression differences between FP and FC regions, we focused mainly on comparisons between EC and EQ and EQ and FP for most of our analysis.

### Comparison of selected transcript levels with corresponding protein levels in microdissected lens subregions and with protein levels reported in previous studies

**Figure 3:**
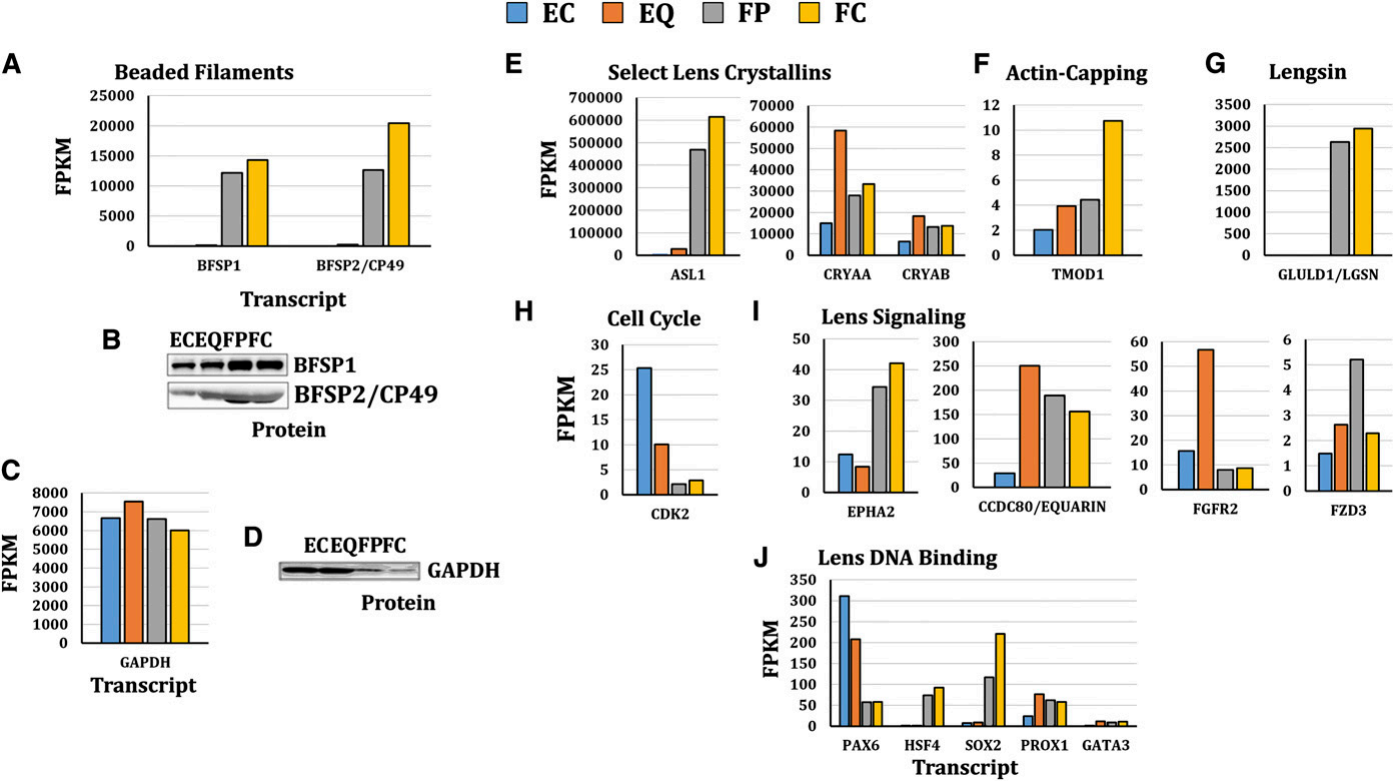
Transcript comparison to western blot protein level and literature based comparison of select canonical lens development pathways. Transcript levels of (A) beaded filament structural protein 1, filensin (BFSP1) (Ireland *et al.* 2000, Perng *et al.* 2007), beaded filament structural protein 2, phakinin (BFSP2/CP49) (Ireland *et al.* 2000, Perng *et al.* 2007), and (C) glyceraldehyde-3-phosphate dehydrogenase (GAPDH) were compared with protein levels (B, D) determined by western analysis. (E−J) Well-studied lens protein encoding transcripts were examined to assess agreement of E13 spatial RNAseq expression data with previously reported expression patterns in lens differentiation-state specific regions. These transcripts included (E) select lens crystallins encoding transcripts δ−crystallin (CRYD1/ASL1) (Gunhaga 2011), αA-crystallin (CRYAA) (Hawse *et al.* 2005), and αB-crystallin (Hawse *et al.* 2005); (F) actin-capping regulator encoding transcript tropomodulin 1 (TMOD1) (Nowak and Fowler 2012); (G) Lensgin (GLULD1/LGSN) (Wyatt *et al.* 2008); (H) cell-cycle regulator encoding transcript cyclin-dependent kinase 2 (CDK2) (Gao *et al.* 1999); (I) lens signaling encoding transcripts coiled-coil domain containing 80 (CCDC80/EQUARIN) (Song *et al.* 2012), EPH receptor type A2 (EPHA2) (Shi *et al.* 2012; Cheng *et al.* 2013), fibroblast growth factor receptor 2 (FGFR2) (Robinson 2006), and frizzled class receptor 3 (FZD3) (Dawes *et al.* 2013); and (J) lens DNA binding encoding transcripts paired box 6 (PAX6) (Cvekl and Piatigorsky 1996), heat shock transcription factor 4 (HSF4) (Fujimoto *et al.* 2004; Somasundaram and Bhat 2004), SRY (sex determining region Y)-box 2 (SOX2) (Kondoh *et al.* 2004), prospero homeobox 1 (PROX1) (Duncan *et al.* 2002), and GATA binding protein 3 (GATA3) (Maeda *et al.* 2009).

The relationship between transcript levels and protein levels of selected genes was analyzed by western blot using soluble protein extracts from the same microdissected portions of the E13 lens (modeled in Figure 1 and Figure 8). Comparison of transcript and protein levels for beaded filament structural protein 1 (BFSP1/filensin) and beaded filament structural protein 2 (BFSP2/phakinin) revealed similar trends between transcript and protein levels (Figure 3, A and B), whereas there were small differences in transcript and protein expression patterns for glyceraldehyde-3-phosphate dehydrogenase (Figure 3, C and D). For comparison, the levels of selected transcripts exhibiting gene-expression differences between lens epithelial cells and fiber cells were also compared with their relative protein levels reported in previous studies (Figure 3, E−J: E, lens crystallins; F, actin-capping; G, lengsin; H, cell cycle; I, lens signaling; and J, lens DNA binding). This comparison confirmed that the differences detected by the present study were comparable with those reported previously. It is important to mention that in the cufflinks transcript abundance likelihood model (presented throughout this article) delta-(δ) crystallin (CRYD1/ASL1) was omitted from cufflinks analysis because ASL1 surpassed the default mapped reads set by cufflinks. In a separate cufflinks analysis performed to include ASL1, ASL1 demonstrated lens fiber cell preferred expression as expected (Figure 3E) (Gunhaga 2011).

### Transcript expression levels of the mitochondrial genome and select nuclear encoded mitochondrial transcript and corresponding protein expression levels

**Figure 4:**
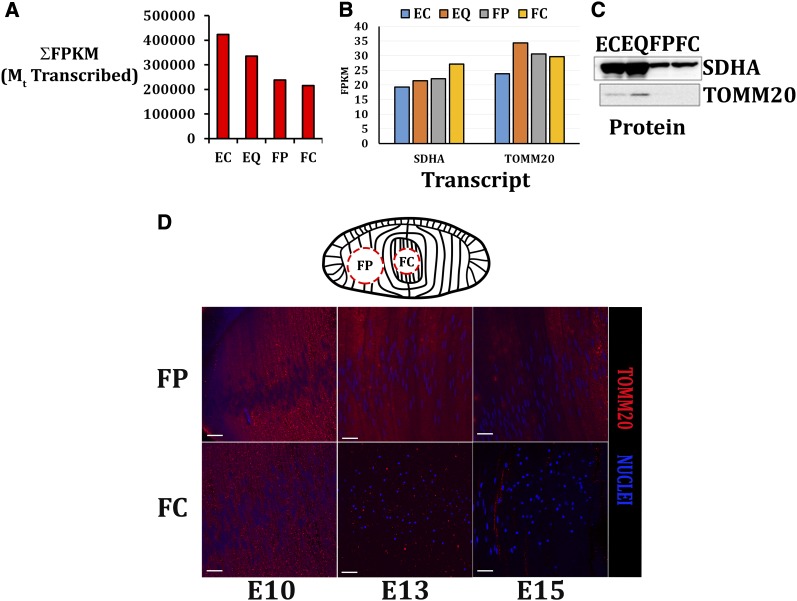
Detected polyadenylated mitochondrial-encoded transcripts display an approximate decrease in expression during lens epithelial to fiber cell differentiation. (A) Detected mitochondrial transcripts (M_t_) display an approximate linear decrease in expression during lens fiber cell differentiation. Transcript expression levels of nuclear encoded inner and outer mitochondrial membrane proteins succinate dehydrogenase complex, subunit A (SDHA) and translocase of the outer mitochondrial membrane 20 homolog (TOMM20) display largely unaltered transcript levels (B) with decreased in protein levels detected by western blotting (C) as lens cell differentiation proceeded. (D) TOMM20 (red) immunofluorescence analysis of differentiating lens fiber cells in sections of the embryonic chicken lens at E10, E13, and E15. Results showed largely decreased TOMM20 levels at E13 compared with E10 that expanded at E15 and preceded the loss of nuclei (blue). Scale bars: 20 μm.

The reference chicken mitochondrial genome has a length of 16,775 bp that encodes 22 tRNAs, 2 rRNAs, and 13 polypeptides (Desjardins and Morais 1990; Guan *et al.* 2007). Expression of mitochondrial encoded genes in the E13 chicken lens exhibited an approximately linear decrease (Figure 4A) from undifferentiated lens epithelial cells to differentiating lens fiber cells. Transcript levels of the respective inner and outer mitochondrial membrane subunit proteins succinate dehydrogenase complex, subunit A (SDHA) and translocase of the outer mitochondrial membrane 20 homolog (yeast) (TOMM20) revealed relatively uniform expression during lens cell differentiation (Figure 4B); however, the protein level was decreased in differentiating lens fiber cells (Figure 4C). Consistently, reduced levels of TOMM20 protein were detected in the region where the onset of OFZ formation occurs as demonstrated by confocal microscopy image analysis (Figure 4D) of midsagittal sections of E10, 13, and 15 embryonic chicken lenses. These results demonstrate the presence of mitochondria in the lens epithelium, and, by contrast, the elimination of mitochondria during lens fiber cell differentiation.

### Comparison of the levels of mitochondrial regulatory- and degradation-associated transcripts with corresponding protein levels

**Figure 5:**
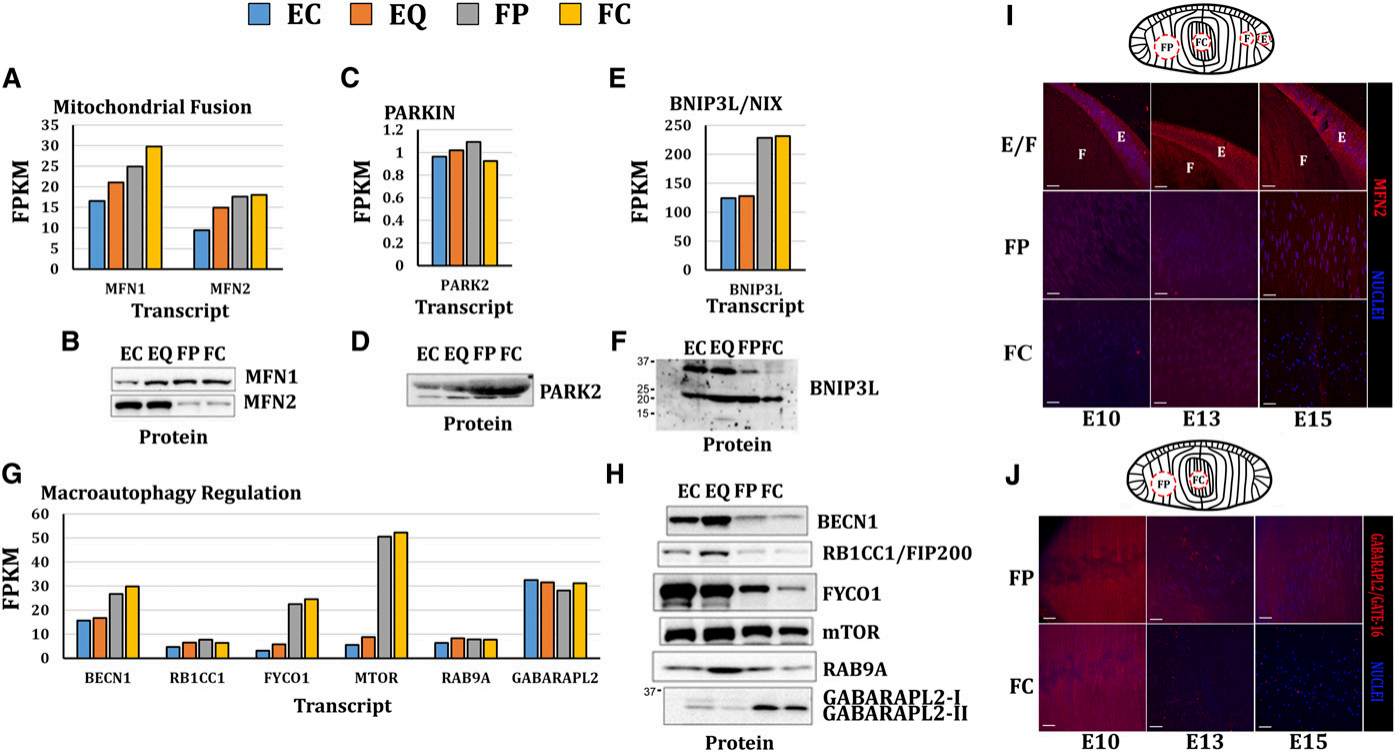
Select mitochondrial regulation, mitophagy, and macroautophagy regulatory protein comparison with transcript levels by western blot and immunofluorescent analysis. Protein levels determined by western analysis were compared with respective transcript levels for mitochondrial fusion transcripts encoding proteins mitofusin-1 and mitofusin-2 (MFN-1, -2) (A, B); mitophagy transcripts encoding proteins Parkin E3 ubiquitin ligase (PARK2/Parkin) (C, D) and BCL2/adenovirus E1B 19kDa interacting protein 3-like (BNIP3L/NIX) (E, F); and macroautophagy regulation transcripts encoding proteins (G, H) beclin 1, autophagy related (BECN1), RB1-inducible coiled-coil 1 (RB1CC1), FYVE and coiled-coil domain containing 1 (FYCO1), mechanistic target of rapamycin (serine/threonine kinase) (mTOR), RAB9A, member RAS oncogene family (RAB9A), GABA(A) receptor-associated protein-like 2 (GABARAPL2). Cryosections from embryonic E10, E13, and E15 chicken lenses were immunostained for MFN2 (I, Red) or GABARAPL2/GATE16 (J, red) (nuclei, blue) and analyzed by confocal microscopy. Scale bars: 20 μm.

To determine the relationship between transcript levels and protein levels of selected mitochondrial regulators western blots were performed using soluble protein extracts isolated on the same microdissected lens regions (Figure 5). MFN1 (Chen *et al.* 2003) and RAB9A, a member of the RAS oncogene family (Nishida *et al.* 2009), revealed similar expression trends between transcript and protein levels, whereas there were small differences in transcript and protein expression patterns for RB1-inducible coiled-coil 1 (RB1CC1/FIP200) (Kim *et al.* 2013), BNIP3L/NIX (Randow and Youle 2014), and Parkin (Randow and Youle 2014). FYVE and coiled-coil domain containing 1 (FYCO1) (Pankiv *et al.* 2010, Chen *et al.* 2011), mechanistic target of rapamycin serine/threonine kinase (mTOR) (Jung *et al.* 2010), mitofusin-2 (MFN2) (Chen *et al.* 2003), LC3 homolog GABA(A) receptor-associated protein-like 2 (GATE16/GABARAPL2) (Weidberg *et al.* 2010), beclin 1, and autophagy related 6 (BECN1) (Kan *et al.* 2011) all revealed differences between the transcript and protein levels.

As a further comparison, immunoflourescent staining of lens sections from E10, a stage of development before the formation of the OFZ, E13, a stage of development after OFZ formation was initiated, and E15, where the OFZ has formed, was performed for selected proteins. Immunofluorescent staining of MFN2 (Figure 5I) in lens epithelium and differentiating lens fiber cells during the transition from E10 to E13 to E15 revealed greater staining of MFN2 in the lens epithelial zones than in the fiber cell zones, consistent with MFN2 levels by western blotting (Figure 5, B−I comparison) with punctate staining patterns for MFN2 in differentiating lens fiber cells at E15. Greater levels of GABARAPL2-II relative to GABARAPL2-I were found by western blot analysis in the FP and FC regions of the microdissected E13 lens (Figure 5H). Immunolocalization studies showed that by E13, GABARAPL2 was localized almost exclusively to puncta in the central fiber cells (FC), a pattern consistent with the association of GABARAPL2 with autophagic vesicles (Figure 5J).

Collectively, these comparisons (Figure 3, Figure 4, and Figure 5) suggest that at least for some mRNAs posttranscriptional and/or posttranslational mechanisms may participate in regulating the availability (levels) and/or translation of mitochondrial-associated mRNAs in the lens. Of particular interest are differences between transcript level and protein level for GABARAPL2. GABARAPL2 immunostaining reveals distinct puncta consistent with its potential role in autophagy and mitophagy. The relatively high level of GABARAPL2 transcript expression in differentiating lens fibers could indicate a need for high levels of GABARAPL2 mRNA to compensate for the degradation of GABARAPL2 protein that occurs during autophagy. Likewise, FYCO1, BECN1, and BNIP3L/NIX also display greater levels of mRNA than corresponding protein levels, suggesting that increased levels of mRNA could compensate to replenish these proteins upon their degradation in the autophagy and mitophagy processes.

### Gene clustering and statistical analysis of mitochondrial regulatory pathways

**Figure 6:**
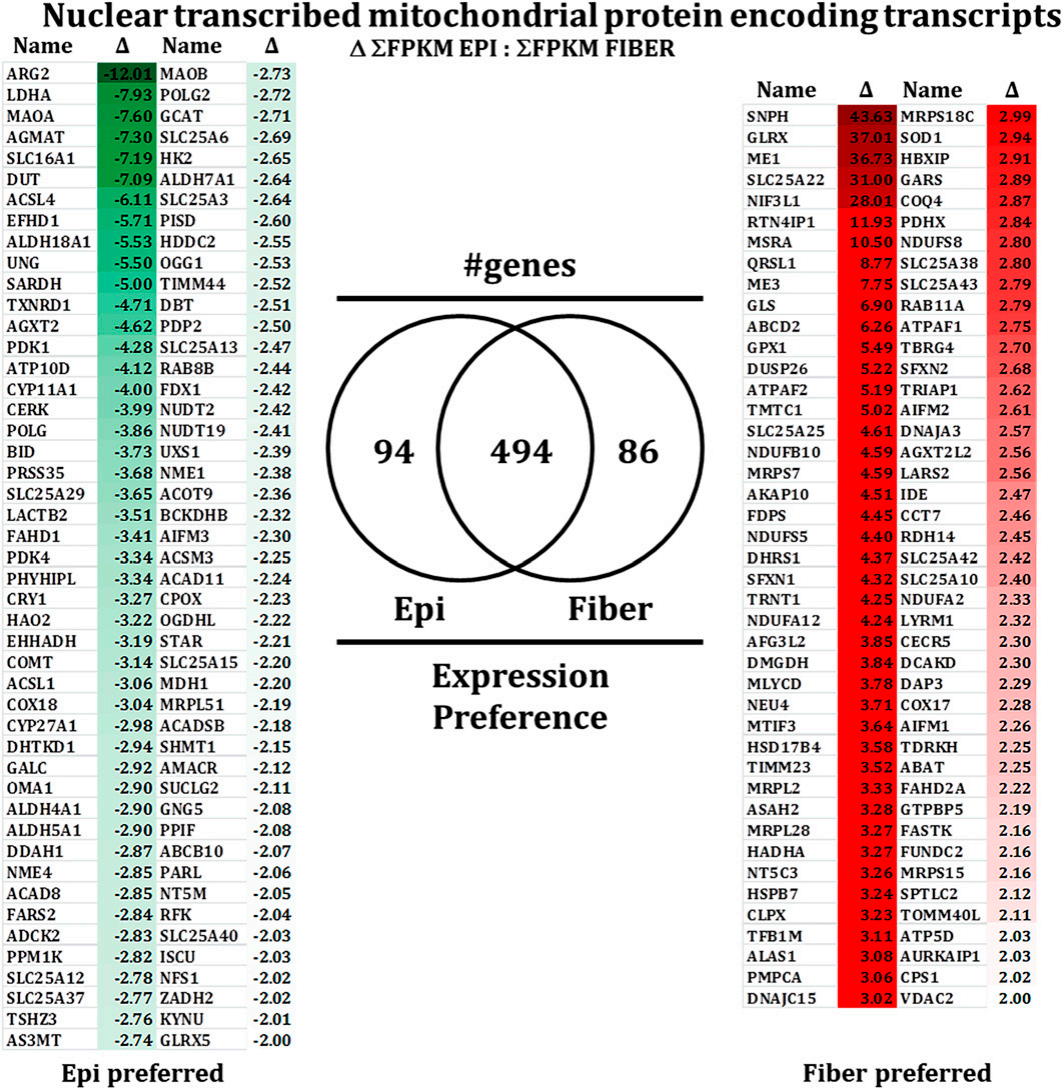
Identification of nuclear transcribed mitochondrial protein encoding transcripts. Our analysis revealed the expression of more than 650 nuclear transcribed mitochondrial protein encoding transcripts with 94 transcripts preferred to the lens epithelia and 86 transcripts preferred to the lens fibers. Transcripts encoding proteins involved in mitochondrial based apoptotic induction (BID, APAF1) demonstrated high levels of expression in the lens epithelium. Transcripts encoding proteins involved in mitochondrial immobilization (SNPH), respiratory chain complex inhibition (DNAJC15), mitochondrial fragmentation (DNAJA3) and mitochondrial repair and protection (GLRX, MSRA) demonstrated high levels of expression in differentiating lens fiber cells. Direct comparisons of central epithelium to equatorial epithelium or equatorial epithelium to peripheral fibers for this analysis are presented as supplementary (Table S7, Table S8, Table S9, and Table S10).

**Figure 7:**
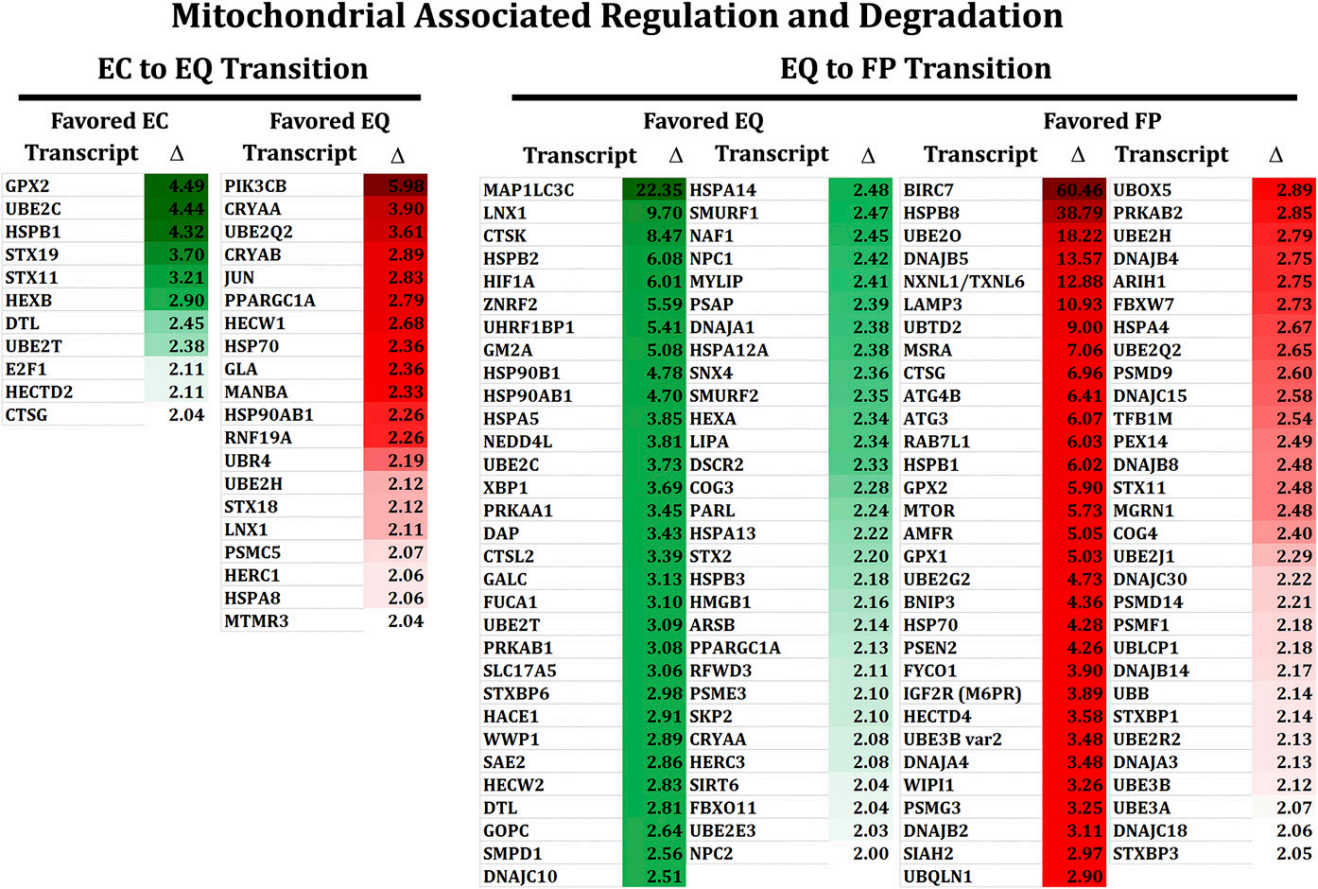
Fold change analysis of detected nuclear encoded mitochondrial-associated degradation and regulation pathways between EC to EQ and EQ to FP transitions. Transcripts encoding proteins involved in mitophagy, autophagy, lysosomal biogenesis, mitochondrial biogenesis, mitochondrial repair/protection, the ubiquitin-proteasome system or heat shock proteins 70/40/27/22 family were compared for the (A) EC to EQ transition and the (B) EQ to FP transition. The FPKMs generated for all genes from these categories are provided as supplementary (File S3). Fold change (Δ) was calculated as discussed in the methods. EC, lens central epithelium; EQ, equatorial epithelium; FP, cortical fibers; FPKM, fragments per kilobase of exon per million fragments mapped.

Unique gene transcripts were sorted into nuclear transcribed mitochondrial proteins (Pagliarini *et al.* 2008) (Figure 6, Table S7, Table S8, Table S9, Table S10, and File S2) and mitochondrial-associated degradation and regulatory proteins (Figure 7 and File S3) as explained in the section *Materials and Methods*. The statistical analysis performed by cuffdiff (ver. 2.1.1; Table S1) revealed that multiple mitochondrial regulatory pathways exhibited statistically significant differences in their levels of expression between differentiation-state specific regions of the embryonic lens. More than 3000 differently expressed transcripts (Figure 2) between lens epithelium and differentiating lens fibers were identified in total. Of these, more than 75 transcripts have roles in mitochondrial regulation and degradation.

Gene clustering of these transcripts (Figure 7 and File S3) revealed their participation in macroautophagy regulation, mitophagy initiation, autophagosome initiation, autophagosome expansion/maturation, autophagosome trafficking/fusion, lysosomal biogenesis, mitochondrial biogenesis, mitochondrial repair/protection, proteasome assembly, E1 ubiquitin-activating activity, E2 ubiquitin conjugating activity, E3 ubiquitin ligase activity, or heat-shock chaperone functions. Transcripts encoding proteins involved in mitophagy and selective macroautophagy were detected within all lens cell subregions consistent with their playing roles in mitochondrial degradation in separate lens subregions (Figure 7 and File S3). These included members of the separate mitophagy pathways, Parkin pathway (Randow and Youle 2014), BNIP3L/NIX (Zhang and Ney 2009, Randow and Youle 2014), and BNIP3 (Zhang and Ney 2009). Interestingly, Parkin members were expressed throughout the lens indicating a possible role in degradation of damaged mitochondria while BNIP3L/NIX and BNIP3 exhibited preferential expression in maturing lens fiber cells indicating a specific role in the elimination of lens mitochondria during lens fiber cell maturation (Figure 5, Figure 7, and File S3).

## Discussion

To identify those mitochondrial regulatory and degradation genes that maintain functional populations of mitochondria in the lens epithelium and eliminate mitochondria in the lens fiber cells, we used high-throughput mRNA sequencing combined with bioinformatics analysis to determine the entire spectrum and range of transcripts expressed by EC, EQ, FP, and FC cells of the E13 chicken lens. The E13 chicken lens maintains large numbers of mitochondria in the lens epithelium and eliminates mitochondria in the cortical fiber cells and therefore is a good model to identify mitochondrial regulatory and degradation mechanisms that regulate the different requirements of these distinct mitochondrial populations. We hypothesized that distinct mitophagy genes and their associated pathways would operate to maintain mitochondria in lens epithelial cells and eliminate mitochondria in lens fiber cells for lens transparency. Because these processes are critical for the development, homeostasis, and transparency of the entire lens, we also hypothesize that the identified mitochondrial regulatory and degradation transcripts could be candidates for causing cataract formation if their normal functions were lost.

**Figure 8:**
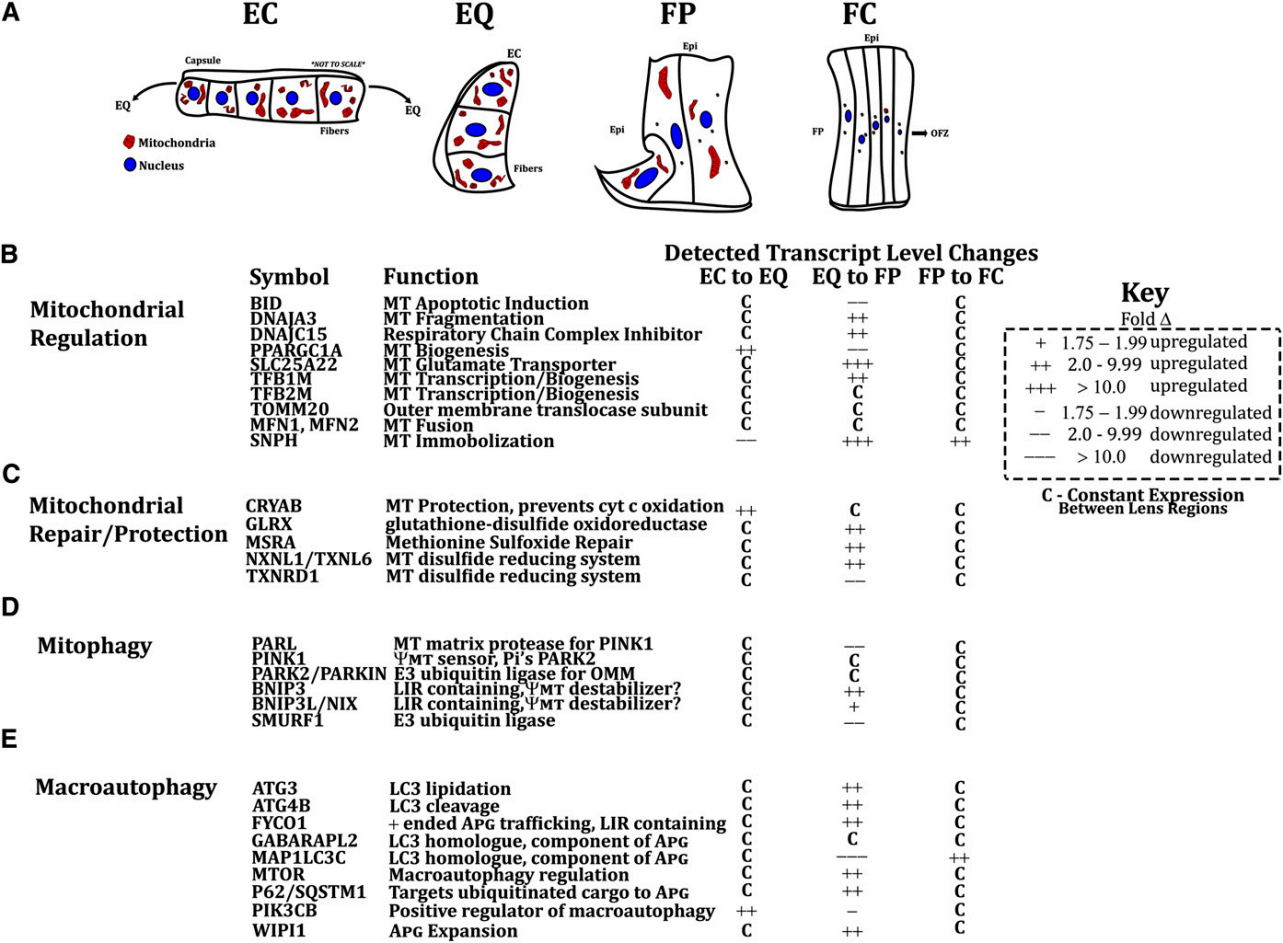
Novel and noteworthy identified mitochondrial regulators between specific lens subregions. (A) A schematic diagram of mitochondrial distribution between the EC, EQ, FP, and FC regions of the day 13 embryonic chicken lens showing active mitochondrial populations and elimination of mitochondria during lens fiber cell maturation. (B) Mitochondrial regulatory transcripts exhibiting increased (+) or decreased (−) levels of expression between indicated lens subregions. (C) Mitochondrial repair and protection transcripts exhibiting increased (+) or decreased (−) levels of expression between indicated lens subregions. (D) Mitophagy transcripts exhibiting increased (+) or decreased (−) levels of expression between indicated lens subregions. (E) Macroautophagy transcripts exhibiting increased (+) or decreased (−) levels of expression between indicated lens sub-regions. APG, autophagic vesicle; EC, central lens epithelium; EQ, equatorial lens epithelium; FP, cortical lens fibers; FC, central lens fibers; LIR, LC3-interacting region; MT, mitochondria(l); OFZ, organelle-free zone; OMM, outer mitochondrial membrane; Pi’s, phosphorylates; ψMT, mitochondrial transmembrane potential.

Our data identified more than 3000 transcripts that exhibited significant lens differentiation-state specific gene expression differences (Figure 2). Of these, more than 75 mitochondrial-associated transcripts exhibited significant differences in expression levels between the lens subregions examined. These included multiple transcripts specific for mitochondrial regulatory, homeostatic, and degradation functions. In total more than 900 transcripts encoding proteins that are directly or are *de facto*-associated with mitochondrial regulation, structure, function and/or degradation were expressed at different levels at different states of lens cell differentiation (Figure 4, Figure 5, Figure 6, Figure 7, and Figure 8).

These data provide evidence that the expression of distinct mitochondria-associated genes is important for maintaining functional populations of mitochondria in lens epithelial cells. For example, transcripts encoding proteins involved in oxidoreductase (TXNRD1, NQO2), nitric oxide/polyamine metabolism (ARG2, AGMAT), dUTP nucleotide metabolism (DUT), lipid metabolism (ACSL1, ACSL4) pyruvate decarboxylation (PDK1), and mitochondrial-based apoptotic induction (BID, APAF1) (Li *et al.* 1998; Yin *et al.* 1999; Wang and Youle 2009) encoding transcripts were significantly higher in the lens epithelium than in lens fiber cells (Figure 6). These expression differences are consistent with the requirement for energy production by the mitochondria for the many lens homeostatic functions carried out by the lens epithelium. By contrast, expression of multiple repair and protective genes (MSRA, NXNL1/TXNL6, GLRX, GPX1, CRYAB) (Brennan and Kantorow 2009, Brennan *et al.* 2009a,b, Brennan *et al.* 2010, McGreal *et al.* 2012, 2013; Kantorow *et al.* 2012), mitochondrial transcription/biogenesis (TFB1M) (Gleyzer *et al.* 2005), mitochondrial immobilization (SNPH) (Kang *et al.* 2008; Chen and Sheng 2013), mitochondrial respiratory chain complex inhibition (DNAJC15) (Hatle *et al.* 2013), mitochondrial fragmentation (DNAJA3) (Elwi *et al.* 2012), and the mitochondrial glutamate transporter (SLC25A22) exhibited greater expression in the newly forming lens fiber cells than in the lens epithelium, suggesting the need for increased mitochondrial biogenesis, protection-repair, and mitochondrial metabolic activity in lens cells undergoing differentiation, cellular remodeling, and high rates of protein synthesis.

The most striking mitochondrial-associated gene expression differences between lens epithelial cells and lens fiber cells were for mitochondrial degradation and elimination genes. Significant increases in gene expression levels occurred for mitophagy-, macroautophagy-, and ubiquitin-proteasome−associated transcripts in the maturing lens fiber cells (Figure 6, Figure 7, and File S3, respectively). Interestingly, the majority of the mitochondrial degradation−associated transcripts were expressed at the greatest levels in differentiating lens fibers, consistent with the need to eliminate mitochondria in these cells for lens transparency. Two major mitophagy transcripts that increased in lens fiber cells were BNIP3L/NIX and BNIP3, which are associated with mitochondrial membrane depolarization and LC3B/GABARAP homolog based tethering and therefore autophagosome recruitment (Novak *et al.* 2010) to the mitochondria (Ding and Yin 2012; Randow and Youle 2014). By contrast, lens epithelial cells had greater levels of PARL, a member of the Parkin-mediated mitophagy pathway (Jin and Youle 2012; Randow and Youle 2014), and similar levels of phosphatase and tensin homolog−induced putative kinase 1 (PINK1) and Parkin to lens fiber cells. Collectively, these results suggest that a balance between different mitophagy mechanisms mirrors the need to maintain functional mitochondrial populations in lens epithelial cells and eliminate mitochondria in lens fiber cells.

In addition to decreased PARL expression and increased expression of the BNIP3L/NIX and BNIP3 mitophagy genes in the lens fiber cells, the levels of p62/SQSTM1 transcript whose encoded protein binds ubiquitinated outer mitochondrial membrane proteins, and acts as a macroautophagy receptor for ubiquitinated cargos, is also increased in expression in differentiating lens fibers relative to lens epithelial cells suggesting the possibility that both the ubiquitin-proteasome degradation system (Caceres *et al.* 2010) and mitophagy (Costello *et al.* 2013) pathways could play combined roles in lens fiber cell mitochondrial elimination. Other studies have reported that, in contrast to the transcript levels analyzed in the present study, p62/SQSTM1 protein levels actually decreased in differentiating lens fiber cells (Wignes *et al.* 2013; Basu *et al.* 2014b) raising the possibility that posttranscriptional and/or translational regulatory mechanisms could operate to regulate p62/SQSTM1 levels in lens cells.

Finally, increased levels of mRNA relative to protein levels were detected in lens fibers for GABARAPL2, FYCO1, BECN1, and BNIP3L/NIX. Because all of these proteins are involved in autophagy and mitophagy and are likely degraded during these processes, our data suggest that transcriptional regulatory mechanisms may increase the level of available mRNA expression for translation of new protein to compensate for the degradation of these proteins during autophagy and mitophagy and thereby ensure their availability.

Collectively, these data combined with previous studies suggests that the distinct mitochondrial populations within different differentiating cell populations of the lens are regulated by distinct mitochondrial associated genes during the transition from lens epithelial cells to lens fiber cells. A selection of these specific genes, their functions, and their lens locations are summarized in Figure 8.

Although our analysis focused mainly on transcriptional expression changes associated with mitochondrial regulation in the lens, the RNA sequencing dataset and bioinformatics analysis reported in the present study also provides a comprehensive window of the entire chicken lens transcriptome and therefore the entire complement of gene expression differences occurring between differentiating lens regions. Combining these data with regulatory RNA analysis during lens cell differentiation (Wolf *et al.* 2013) could pinpoint novel regulators of transcriptional content and the transcriptional program that occurs during the differentiation of lens cells. Further analysis of the data will provide a greater understanding of a wide range of other important processes in the lens, including lens cell survival, homeostasis, differentiation, and degradation of other lens organelles for lens cell differentiation including the nucleus, endoplasmic reticulum and Golgi apparatus, all of which are also degraded during formation of the organelle free zone.

In summary, the present data provide evidence that a plethora of genes orchestrate differentiation-state specific mitochondrial biogenesis, homeostatic and elimination pathways in the eye lens. Further functional and mechanistic studies will be required to identify the individual roles of specific transcripts and their encoded proteins in lens function. Mechanistic studies of the genes identified in the present study, though beyond the scope of the present report, are ongoing by our combined laboratories that aim to pinpoint the precise functions and mechanisms of the identified transcripts for lens epithelial cell mitochondrial regulation, lens cell survival, lens cell differentiation, lens development and the maintenance of lens transparency.

## Supplementary Material

Supporting Information
