# Supplementary material for: Differentiation State-Specific Mitochondrial Dynamic Regulatory Networks Are Revealed by Global Transcriptional Analysis of the Developing Chicken Lens
Source: G3 (Bethesda). 2014 Jun 13;4(8):1515–27. doi: 10.1534/g3.114.012120 (PMC4132181; doi:10.1534/g3.114.012120)
Supplement: Supporting Information [file supp_4_8_1515__index.html]

Differentiation State-Specific Mitochondrial Dynamic Regulatory Networks Are Revealed by Global Transcriptional Analysis of the Developing Chicken Lens — Supporting Information 

# Differentiation State-Specific Mitochondrial Dynamic Regulatory Networks Are Revealed by Global Transcriptional Analysis of the Developing Chicken Lens

## Supporting Information for Chauss *et al.*, 2014

**Files in this Data Supplement:**

- Supporting Information - Tables S1-S10 and Files S1-S3 (PDF, 418 KB)
- Table S1 - Detected EC gene-specific transcripts statistically decreased in expression during EC to EQ transition. (PDF, 144 KB)
- Table S2 - Detected EC gene-specific transcripts statistically decreased in expression during EC to EQ transition. (PDF, 149 KB)
- Table S3 - Detected EQ gene-specific transcripts statistically decreased in expression during EQ to FP transition. (PDF, 228 KB)
- Table S4 - Detected EQ gene-specific transcripts statistically increased in expression during EQ to FP transition. (PDF, 182 KB)
- Table S5 - Detected FP gene-specific transcripts statistically decreased in expression during FP to FC transition. (PDF, 121 KB)
- Table S6 - Detected FP gene-specific transcripts statistically increased in expression during FP to FC transition. (PDF, 121 KB)
- Table S7 - Nuclear encoded mitochondrial protein transcript that demonstrated a two-fold decrease in expression or greater during EC to EQ transition. (PDF, 122 KB)
- Table S8 - Nuclear encoded mitochondrial protein transcript that demonstrated a two-fold increase in expression or greater during EC to EQ transition. (PDF, 123 KB)
- Table S9 - Nuclear encoded mitochondrial protein transcript that demonstrated a two-fold decrease in expression or greater during EQ to FP transition. (PDF, 133 KB)
- Table S10 - Nuclear encoded mitochondrial protein transcript that demonstrated a two-fold increase in expression or greater during EQ to FP transition. (PDF, 128 KB)
- File S1 - Raw Cufflinks output including the raw total cuffdiff differential expression analysis. These files are tab delimited and can be opened with a Text editor or Microsoft Excel. (.zip, 12 MB)
- File S2 - Total assembled nuclear transcribed mitochondrial transcript FPKMs. (.xlsx, 113 KB)
- File S3 - Total assembled mitochondrial dynamic pathways transcript FPKMs. (.xlsx, 43 KB)
