# Supplementary material for: Differentiation State-Specific Mitochondrial Dynamic Regulatory Networks Are Revealed by Global Transcriptional Analysis of the Developing Chicken Lens
Source: G3 (Bethesda). 2014 Jun 13;4(8):1515–27. doi: 10.1534/g3.114.012120 (PMC4132181; doi:10.1534/g3.114.012120)
Supplement: Supporting Information [file supp_g3.114.012120_012120SI.pdf]

G3· Genes | Genomes | Genetics

Differentiation state-specific mitochondrial dynamic regulatory networks are revealed by global transcriptional analysis of the developing chicken lens.

Chauss D[1], Basu S[2], Rajakaruna S[2], Ma Z[3], Gau V[1], Anastas S[1], Brennan LA[1], Hejtmancik JF[3], Menko AS[2], Kantorow M[1*].

[1]Department of Biomedical Science, Florida Atlantic University, Boca Raton, FL. [2]Department of Pathology, Anatomy and Cell Biology, Thomas Jefferson University. [3]Ophthalmic Genetics and Visual Function Branch, National Eye Institute, National Institutes of Health, Bethesda, MD.

[*]Corresponding Author: Florida Atlantic University, Charles E. Schmidt College of Medicine, Dept. of Biomedical Sciences, Boca Raton, FL 33431, email: mkantoro@fau.edu

DOI: 10.1534/g3.114.012120

Files S1-S3

Available for download at http://www.g3journal.org/lookup/suppl/doi:10.1534/g3.114.012120/-/DC1

File S1 Raw Cufflinks output including the raw total cuffdiff differential expression analysis. These files are tab delimited and can be opened with a Text editor or Microsoft Excel.

File S2 .xls file of total assembled nuclear transcribed mitochondrial transcript FPKMs.

File S3 .xls file of total assembled mitochondrial dynamic pathways transcript FPKMs.

Table S1 Detected EC gene-specific transcripts statistically decreased in expression during EC to EQ transition.

Gene	Description	log2(Fold Change)	p-value*
SERPINI1	Neuroserpin	-2.1	1.2E-03
NANOS1	nanos homolog 1 (Drosophila)	-2.1	1.2E-03
APOA1	Apolipoprotein A-I	-2.2	1.2E-03
FABP7	Fatty acid-binding protein, brain	-2.2	1.2E-03
MAPK10	mitogen-activated protein kinase 10	-2.2	1.2E-03
MGP	Matrix Gla protein	-2.2	1.2E-03
FAM169A	family with sequence similarity 169, member A	-2.4	1.2E-03
DPP6	dipeptidyl-peptidase 6	-2.4	1.2E-03
PCDH1	protocadherin-1 precursor	-2.4	1.2E-03
SLC6A1	Transporter	-2.4	1.2E-03
DCP1A	decapping mRNA 1A	-2.4	1.2E-03
SOD3	Superoxide dismutase [Cu-Zn]	-2.4	1.2E-03
KIAA1239	*KIAA1239*	-2.4	1.2E-03
SCUBE3	signal peptide, CUB domain, EGF-like 3	-2.5	1.2E-03
CHMP4C	chromatin modifying protein 4C	-2.6	1.2E-03
HBAD	Hemoglobin subunit alpha-D	-2.6	1.2E-03
HBAA	Hemoglobin subunit alpha-A	-2.6	1.2E-03
ALDH6	aldehyde dehydrogenase family 1 member A3	-2.6	1.2E-03
TFRC	transferrin receptor protein 1	-2.7	1.2E-03
CLEC19A	C-type lectin domain family 19, member A	-2.7	1.2E-03
SIX6	Homeobox protein *SIX6*	-2.7	1.2E-03
CRABP-I	Cellular retinoic acid-binding protein 1	-2.7	1.2E-03
ENSGALG00000005470	novel gene	-2.7	1.2E-03
CNTN3	contactin 3 (plasmacytoma associated)	-2.7	1.2E-03
SLCO4A1	solute carrier organic anion transporter family member 4A1	-2.7	1.2E-03
HBB	Hemoglobin subunit beta	-2.7	1.2E-03
EFEMP1	EGF containing fibulin-like extracellular matrix protein 1	-2.8	1.2E-03
RDH10	retinol dehydrogenase 10 (all-trans)	-2.8	1.2E-03
SLC45A4	solute carrier family 45, member 4	-2.9	1.2E-03
MYLK	myosin light chain kinase, smooth muscle	-2.9	1.2E-03
GPR20	Uncharacterized protein	-2.9	1.2E-03
NPR3	natriuretic peptide receptor C/guanylate cyclase C (atrionatriuretic peptide receptor C)	-2.9	1.2E-03
LRRC38	leucine rich repeat containing 38	-2.9	1.2E-03
ENSGALG00000013280	novel gene	-2.9	1.2E-03
LPL	Lipoprotein lipase	-2.9	1.2E-03
AXDND1	axonemal dynein light chain domain containing 1	-2.9	1.2E-03
AKR	aldo-keto reductase family 1 member B10	-3.0	1.2E-03
TSPAN4	tetraspanin 4	-3.0	1.2E-03
SLC5A7	high affinity choline transporter 1	-3.0	1.2E-03
GALNT5	UDP-N-acetyl-alpha-D-galactosamine:polypeptide N-acetylgalactosaminyltransferase 5 (GalNAc-T5)	-3.1	1.2E-03
PCDHA11	protocadherin alpha 11 precursor	-3.1	1.2E-03
COL9A2	collagen, type IX, alpha 2	-3.1	1.2E-03
WBSCR17	Williams-Beuren syndrome chromosome region 17	-3.2	1.2E-03
FAM5B	family with sequence similarity 5, member B	-3.2	1.2E-03
PARM1	prostate androgen-regulated mucin-like protein 1	-3.3	1.2E-03
LGR5	leucine-rich repeat containing G protein-coupled receptor 5	-3.3	1.2E-03
TMEM72	transmembrane protein 72	-3.3	1.2E-03
NXPH2	neurexophilin 2	-3.3	1.2E-03
VIPR1	vasoactive intestinal polypeptide receptor 1 precursor	-3.4	1.2E-03
KCNT1	Potassium channel subfamily T member 1	-3.5	1.2E-03
FAR-2	contactin-5 precursor	-3.5	1.2E-03
ADPRHL1	ADP-ribosylhydrolase like 1	-3.6	1.2E-03
GEM	GTP-binding protein *GEM*	-3.6	1.2E-03
CPXM2	carboxypeptidase X (M14 family), member 2	-3.8	1.2E-03
SLC29A4	solute carrier family 29 (equilibrative nucleoside transporter), member 4	-3.9	1.2E-03

ADAMTS3	ADAM metallopeptidase with thrombospondin type 1 motif, 3	-4.0	1.2E-03
PTPRO	receptor-type tyrosine-protein phosphatase O	-4.3	1.2E-03
CYTL1	cytokine-like 1	-4.3	1.2E-03
RGS2	regulator of G-protein signaling 2	-4.4	1.2E-03
R3HDML	R3H domain containing-like	-4.4	1.2E-03
CHGB	chromogranin B (secretogranin 1)	-4.6	1.2E-03
PLEKHG4	pleckstrin homology domain containing, family G (with RhoGef domain) member 4 **	-4.7	1.2E-03
CGN	cingulin	-5.2	1.2E-03
DKK-1	*Dkk-1*; Uncharacterized protein	-5.6	1.2E-03
RASD1	dexamethasone-induced Ras-related protein 1	-5.7	1.2E-03
SIVA1	*SIVA1*, apoptosis-inducing factor	-1.9	2.0E-03
COL5A1	collagen alpha-1(V) chain precursor	-1.9	2.0E-03
FSTL4	follistatin-related protein 4 precursor	-2.2	2.0E-03
CLSTN2	calsyntenin 2	-2.3	2.0E-03
ZIC1	zinc finger protein *ZIC 1*	-2.4	2.0E-03
LRFN5	leucine rich repeat and fibronectin type III domain containing 5	-2.6	2.0E-03
FMO6P	flavin containing monooxygenase 6 pseudogene	-2.7	2.0E-03
STK17A	serine/threonine-protein kinase 17A	-2.8	2.0E-03
PTRF	polymerase I and transcript release factor	-3.0	2.0E-03
ST3GAL1	CMP-N-acetylneuraminate-beta-galactosamide-alpha-2,3-sialyltransferase 1	-3.1	2.0E-03
VWF	von Willebrand factor	-3.1	2.0E-03
COL6A1	collagen alpha-1(VI) chain precursor	-3.5	2.0E-03
PLCD1	Uncharacterized protein	-2.0	2.8E-03
MGAT4A	Alpha-1,3-mannosyl-glycoprotein 4-beta-N-acetylglucosaminyltransferase A	-2.1	2.8E-03
UBE2C	ubiquitin-conjugating enzyme E2C	-2.2	2.8E-03
ENSGALG00000023581	Uncharacterized protein	-2.3	2.8E-03
COL12A1	collagen alpha-1(XII) chain precursor	-2.4	2.8E-03
NEFM	neurofilament medium polypeptide	-3.5	2.8E-03
CYP26B1	cytochrome P450, family 26, subfamily B, polypeptide 1	-3.7	2.8E-03
CACNG5	calcium channel, voltage-dependent, gamma subunit 5	-1.9	3.5E-03
SOCS2	suppressor of cytokine signaling 2	-2.0	3.5E-03
CDH11	cadherin-11 precursor	-2.4	3.5E-03
GAS1	growth arrest-specific 1	-2.8	3.5E-03
SPAG5	sperm associated antigen 5	-1.8	4.2E-03
AGRN	agrin	-2.0	4.2E-03
THBS4	thrombospondin 4	-2.1	4.2E-03
PDLIM3	PDZ and LIM domain protein 3	-2.2	4.2E-03
CPA6	carboxypeptidase A6	-2.3	4.2E-03
GRIK3	glutamate receptor, ionotropic, kainate 3	-2.4	4.2E-03
KCNS1	potassium voltage-gated channel, delayed-rectifier, subfamily S, member 1	-2.6	4.2E-03
LY6E	lymphocyte antigen 6E precursor	-1.9	4.8E-03
CAMK1D	calcium/calmodulin-dependent protein kinase ID	-2.2	4.8E-03
TLL1	tolloid-like protein 1 precursor	-2.4	4.8E-03
IGSF21	immunoglobin superfamily, member 21	-2.6	4.8E-03
CYR61	Protein *CYR61*	-2.8	4.8E-03
HTRA3	HtrA serine peptidase 3	-2.1	5.4E-03
GGA.4981	epsilon globin	-2.1	5.4E-03
MKP3	dual specificity protein phosphatase 6	-2.2	5.4E-03
NRN1	neuritin precursor	-2.7	5.4E-03
VEGFA	Vascular endothelial growth factor A	-2.0	6.0E-03
SHISA2	protein *shisa-2* homolog precursor	-2.1	6.0E-03
VSX2	Visual system homeobox 2	-2.7	6.0E-03
FAM110C	family with sequence similarity 110, member C	-1.8	6.5E-03
PDE4D	phosphodiesterase 4D, cAMP-specific	-2.0	6.5E-03
MDGA2	MAM domain containing glycosylphosphatidylinositol anchor 2	-2.2	6.5E-03
RASSF9	Ras association (RalGDS/AF-6) domain family (N-terminal)	-2.2	6.5E-03

	member 9		
PCDH8	protocadherin-8 precursor	-2.3	6.5E-03
ENSGALG00000028527	novel gene	-3.0	6.5E-03
C1QTNF4	C1q and tumor necrosis factor related protein 4	-3.3	6.5E-03
SLITRK6	SLIT and NTRK-like family, member 6	-2.4	7.0E-03
LAMC2	laminin, gamma 2	-2.7	7.0E-03
CCDC3	coiled-coil domain containing 3	-3.0	7.0E-03
ENSGALG00000006325	Uncharacterized protein	-3.1	7.0E-03
SEZ6L	seizure related 6 homolog (mouse)-like	-3.6	7.0E-03
LEPREL4	leprecan-like 4	-1.7	8.0E-03
GCH1	GTP cyclohydrolase 1	-1.8	8.0E-03
LOXL3	lysyl oxidase-like 3	-1.9	8.0E-03
PI15	Peptidase inhibitor 15	-2.0	8.0E-03
HSPB1	heat shock protein beta-1	-2.1	8.0E-03
ALDH3B1,ENSGALG00000003490	Aldehyde dehydrogenase	-2.4	8.0E-03
PCDH18	protocadherin 18	-3.0	8.0E-03
FBN2	Fibrillin-3; Uncharacterized protein	-1.8	9.5E-03
CACNA1G	calcium channel, voltage-dependent, T type, alpha 1G subunit	-1.8	1.0E-02
LINGO3	leucine rich repeat and Ig domain containing 3	-3.4	1.0E-02
VAV3	guanine nucleotide exchange factor *VAV3*	-1.7	1.1E-02
TFAP2C	transcription factor AP-2 gamma (activating enhancer binding protein 2 gamma)	-1.8	1.1E-02
ACSL4	acyl-CoA synthetase long-chain family member 4	-2.0	1.1E-02
IRK1	inward rectifier potassium channel 2	-3.2	1.1E-02
R3HCC1L	R3H domain and coiled-coil containing 1-like	-1.7	1.1E-02
RAX1	retinal homeobox protein Rx2	-2.2	1.1E-02
PAPLN	papilin, proteoglycan-like sulfated glycoprotein**	-3.0	1.1E-02
PIK3C2G	phosphatidylinositol-4-phosphate 3-kinase, catalytic subunit type 2 gamma	-2.3	1.2E-02
CSDC2	cold shock domain containing C2, RNA binding	-1.6	1.3E-02
ANLN	anillin, actin binding protein	-1.7	1.3E-02
LTF	ovotransferrin precursor	-2.1	1.3E-02
COL9A3	collagen alpha-3(IX) chain precursor	-2.2	1.3E-02
ZIC3	Uncharacterized protein	-2.6	1.3E-02
COL1A2	collagen alpha-2(I) chain precursor	-1.6	1.4E-02
NR2F1	nuclear receptor subfamily 2, group F, member 1	-3.0	1.4E-02
PODXL	podocalyxin-like	-2.0	1.4E-02
ENO2	gamma-enolase	-1.6	1.5E-02
NTN1	Netrin-1	-2.6	1.5E-02
ENSGALG00000012847	novel gene	-1.7	1.6E-02
CPN1	Uncharacterized protein	-2.2	1.6E-02
CXCR4	C-X-C chemokine receptor type 4	-2.6	1.6E-02
NIN	ninein (GSK3B interacting protein)	-1.6	1.6E-02
PLA2G10	phospholipase A2, group X	-2.2	1.6E-02
ISLR2	uncharacterized protein LOC429941 precursor	-2.4	1.6E-02
HEXB	hexosaminidase B (beta polypeptide)	-1.5	1.7E-02
SPATA5	protein sprouty homolog 1	-1.6	1.7E-02
GPX8	Glutathione peroxidase	-2.0	1.7E-02
SH3TC2	SH3 domain and tetratricopeptide repeats 2	-2.0	1.7E-02
ENSGALG00000005747	Uncharacterized protein	-2.2	1.8E-02
CTNND2	catenin (cadherin-associated protein), delta 2	-1.5	1.8E-02
ARHGAP19	rho GTPase-activating protein 19	-1.6	1.9E-02
FAIM2	Fas apoptotic inhibitory molecule 2	-1.9	1.9E-02
PALB2	partner and localizer of BRCA2	-1.9	1.9E-02
ENSGALG00000023973	Uncharacterized protein	-2.3	1.9E-02
KIAA1324L	KIAA1324-like	-1.7	2.0E-02
WFDC1	WAP four-disulfide core domain protein 1	-1.6	2.0E-02
CKS1B	CDC28 protein kinase regulatory subunit 1B	-1.6	2.0E-02
PDIA5	protein disulfide isomerase family A, member 5	-1.7	2.0E-02
NEIL1	endonuclease VIII-like 1	-1.9	2.1E-02
ATP8A2	ATPase, aminophospholipid transporter, class I, type 8A,	-1.6	2.2E-02

	member 2		
SLC39A12	solute carrier family 39 (zinc transporter), member 12	-2.0	2.2E-02
CHRD	chordin precursor	-2.3	2.2E-02
ZIC2	Zic family member 2	-2.4	2.3E-02
ENSGALG00000015363	Uncharacterized protein	-1.7	2.3E-02
VEPH1	ventricular zone expressed PH domain-containing 1	-2.3	2.3E-02
ENSGALG00000014513	novel gene	-1.6	2.3E-02
PLA2G15	phospholipase A2, group XV	-1.8	2.3E-02
SNX16	sorting nexin-16	-1.5	2.4E-02
SH3RF2	SH3 domain containing ring finger 2	-2.2	2.4E-02
PLK2	polo-like kinase 2	-1.5	2.4E-02
MMP11	matrix metallopeptidase 11 (stromelysin 3)	-1.9	2.5E-02
PLOD1	procollagen-lysine,2-oxoglutarate 5-dioxygenase 1 precursor	-2.3	2.5E-02
NBL1	Neuroblastoma suppressor of tumorigenicity 1	-1.8	2.5E-02
CDC45	cell division cycle 45	-1.5	2.6E-02
COL9A1	collagen alpha-1(IX) chain precursor	-2.0	2.6E-02
CHRDL1	chordin-like protein 1 precursor	-2.0	2.6E-02
BMP4	Bone morphogenetic protein 4	-2.1	2.6E-02
LECT1	leukocyte cell-derived chemotaxin 1	-2.2	2.6E-02
PAM	peptidylglycine alpha-amidating monooxygenase	-1.6	2.6E-02
CLDN19	Uncharacterized protein	-2.0	2.6E-02
OLFM1	Noelin	-2.1	2.6E-02
HK2	hexokinase-2	-2.0	2.7E-02
GPM6A	neuronal membrane glycoprotein M6-a	-1.6	2.8E-02
SYT10	synaptotagmin X	-2.1	2.8E-02
CITED2	cbp/p300-interacting transactivator 2	-1.6	2.8E-02
KIF23	kinesin-like protein *KIF23*	-1.4	2.9E-02
PLK1	serine/threonine-protein kinase *PLK1*	-1.4	2.9E-02
LATS2	large tumor suppressor kinase 2	-1.5	2.9E-02
OLFML2B	olfactomedin-like 2B	-1.9	3.0E-02
FZD7	frizzled-7 precursor	-1.5	3.0E-02
MYOC	myocilin, trabecular meshwork inducible glucocorticoid response	-1.7	3.0E-02
RASGRP1	RAS guanyl releasing protein 1 (calcium and DAG-regulated)	-1.8	3.1E-02
EDNRB	endothelin receptor type B precursor	-1.5	3.2E-02
NDC80	Kinetochore protein *NDC80* homolog	-1.5	3.2E-02
PRSS55	protease, serine, 55	-1.9	3.2E-02
PASK	PAS domain containing serine/threonine kinase	-1.5	3.2E-02
ANKRD29	ankyrin repeat domain 29	-1.8	3.2E-02
DEPDC1B	DEP domain-containing protein 1B	-1.6	3.3E-02
INCENP	inner centromere protein	-1.5	3.3E-02
S100A11	Protein *S100-A11*	-2.3	3.3E-02
CLIC6	Uncharacterized protein	-2.2	3.4E-02
AMH	muellerian-inhibiting factor precursor	-2.2	3.4E-02
PCDH19	protocadherin-19 precursor	-1.9	3.4E-02
TIFA	TRAF-interacting protein with forkhead-associated domain	-2.1	3.5E-02
U6	*U6* spliceosomal RNA	/0	3.6E-02
SLCO1C1	solute carrier organic anion transporter family member 1C1	-2.0	3.6E-02
BOC	*BOC* cell adhesion associated, oncogene regulated	-1.5	3.6E-02
LAPTM4B	lysosomal protein transmembrane 4 beta	-1.5	3.7E-02
SERPINB10	Heterochromatin-associated protein MENT [	-2.1	3.7E-02
KCNQ4	potassium voltage-gated channel, KQT-like subfamily, member 4	-1.4	3.8E-02
F10	Coagulation factor X Factor X light chain Factor X heavy chain Activated factor Xa heavy chain	-1.7	3.8E-02
FOXI2	forkhead box I2	-1.7	3.8E-02
TERT	telomerase reverse transcriptase	-1.4	3.8E-02
RPIA	ribose-5-phosphate isomerase	-1.4	3.8E-02
RNASET2	ribonuclease T2 precursor	-1.5	3.8E-02
FN1	fibronectin precursor	-1.5	3.9E-02
CLDN5	claudin 5	-3.1	3.9E-02
STRA6	stimulated by retinoic acid 6	-2.0	4.0E-02

UTS2D	urotensin-2B precursor	-2.1	4.0E-02
EMCN	endomucin	-1.9	4.0E-02
PDE4C	phosphodiesterase 4C, cAMP-specific	-1.4	4.1E-02
ENSGALG00000002749	novel gene	-1.4	4.1E-02
RBP3	retinol-binding protein 3 precursor	-1.4	4.1E-02
ENTPD2	ectonucleoside triphosphate diphosphohydrolase 2 precursor	-1.5	4.1E-02
BARD1	BRCA1-associated RING domain protein 1	-1.5	4.1E-02
TMEM132D	transmembrane protein 132D	-1.5	4.1E-02
HGF/SF	hepatocyte growth factor precursor	-1.4	4.1E-02
OSBPL3	oxysterol binding protein-like 3	-1.4	4.1E-02
ENSGALG00000002955	novel gene	-1.7	4.1E-02
SLC39A8	solute carrier family 39 (zinc transporter), member 8	-2.2	4.1E-02
PDE9A	phosphodiesterase 9A	-2.2	4.1E-02
KIF4A	chromosome-associated kinesin KIF4	-1.4	4.2E-02
GPC4	glypican 4	-2.1	4.2E-02
ENSGALG00000020899	Uncharacterized protein	-2.3	4.2E-02
TSC22D3	TSC22 domain family, member 3	-1.5	4.3E-02
GPX7	glutathione peroxidase 7	-1.7	4.3E-02
EGLN3	egl nine homolog 3 (C. elegans)	-1.9	4.4E-02
MXD3	MAX dimerization protein 3	-1.9	4.4E-02
DISP1	dispatched homolog 1 (Drosophila)	-1.3	4.4E-02
CCNB3	G2/mitotic-specific cyclin-B3	-1.4	4.5E-02
PRX	paired mesoderm homeobox protein 1	-1.8	4.5E-02
ENSGALG00000005114	novel gene	-1.8	4.5E-02
DOCK11	dedicator of cytokinesis 11	-1.4	4.6E-02
BRCA1	breast cancer 1, early onset	-1.3	4.8E-02
MCM3	DNA replication licensing factor *MCM3*	-1.4	4.9E-02
snoZ196	Small nucleolar RNA Z196/R39/R59 family	/0	5.0E-02

*p-values are corrected for multiple testing by the false discovery rate method as utilized by cuffdiff (version 2.1.1).

Table S2 Detected EC gene-specific transcripts statistically decreased in expression during EC to EQ transition.

Gene	Description	log2(Fold Change)	p-value*
ANXA1	annexin A1	4.9	1.2E-03
CER1	cerberus 1, DAN family BMP antagonist	4.4	1.2E-03
BMPER	BMP-binding endothelial regulator protein precursor	4.2	1.2E-03
HRH3	histamine receptor H3	3.9	1.2E-03
STEAP1	six transmembrane epithelial antigen of the prostate 1	3.9	1.2E-03
GATA3	GATA-binding factor 3	3.5	1.2E-03
CRYBB3	Beta-crystallin B3	3.5	1.2E-03
GCHFR	GTP cyclohydrolase 1 feedback regulatory protein	3.5	1.2E-03
CILP	cartilage intermediate layer protein, nucleotide pyrophosphohydrolase	3.4	1.2E-03
GABRA3	gamma-aminobutyric acid (GABA) A receptor, alpha 3	3.3	1.2E-03
ARSH	arylsulfatase H precursor	3.3	1.2E-03
PAG1	phosphoprotein associated with glycosphingolipid microdomains 1	3.3	1.2E-03
HGD	homogentisate 1,2-dioxygenase	3.3	1.2E-03
CYP1B1	cytochrome P450, family 1, subfamily B, polypeptide 1	3.2	1.2E-03
KCNK1	potassium channel, subfamily K, member 1	3.2	1.2E-03
WNT7A	wingless-type MMTV integration site family, member 7A	3.2	1.2E-03
CYP1A4	cytochrome P450 1A4	3.2	1.2E-03
VIT	vitrin precursor	3.2	1.2E-03
RCAN2	regulator of calcineurin 2	3.1	1.2E-03
SCIN	Adseverin	3.1	1.2E-03
CCDC80	coiled-coil domain-containing protein 80 precursor	3.1	1.2E-03
FAM46A	uncharacterized protein LOC421845	3.1	1.2E-03
WNT7B	Protein *Wnt-7b*	3.1	1.2E-03
CDK14	cyclin-dependent kinase 14	3.0	1.2E-03
HEYL	hairy/enhancer-of-split related with YRPW motif-like	3.0	1.2E-03
DLG2	discs, large homolog 2 (Drosophila)	3.0	1.2E-03
MEGF10	multiple EGF-like-domains 10	3.0	1.2E-03
SLC12A3	solute carrier family 12 (sodium/chloride transporters), member 3	2.9	1.2E-03
GLUR1/A	glutamate receptor 1 precursor	2.9	1.2E-03
ADAMTSL3	ADAMTS-like 3	2.9	1.2E-03
RSPO3	R-spondin 3	2.9	1.2E-03
ENSGALG00000015653	junctophilin 1	2.9	1.2E-03
LONRF3	LON peptidase N-terminal domain and ring finger 3	2.9	1.2E-03
ID1	DNA-binding protein inhibitor *ID-1*	2.9	1.2E-03
RELN	reelin	2.9	1.2E-03
KIAA1107	*KIAA1107*	2.8	1.2E-03
JAG1	Delta-like protein	2.7	1.2E-03
FZD10	frizzled-10 precursor	2.7	1.2E-03
SEMA5A	sema domain, seven thrombospondin repeats (type 1 and type 1-like), transmembrane domain (TM) and short cytoplasmic domain, (semaphorin) 5A	2.7	1.2E-03
TAPP2	pleckstrin homology domain-containing family A member 2	2.7	1.2E-03
ENSGALG00000005344	Uncharacterized protein	2.7	1.2E-03
SLC35A5	solute carrier family 35, member A5	2.6	1.2E-03
PIK3CB	phosphatidylinositol-4,5-bisphosphate 3-kinase catalytic subunit beta isoform	2.6	1.2E-03
JAM2	junctional adhesion molecule B precursor	2.6	1.2E-03
ABI3BP	ABI family, member 3 (NESH) binding protein	2.5	1.2E-03
EDC3	enhancer of mRNA-decapping protein 3	2.5	1.2E-03
PIWIL1	Piwi-like protein 1	2.5	1.2E-03
PLCL1	phospholipase C-like 1	2.5	1.2E-03
RHOBTB3	Rho-related BTB domain containing 3	2.5	1.2E-03
AGL	amylo-alpha-1, 6-glucosidase, 4-alpha-glucanotransferase	2.5	1.2E-03
CPM	carboxypeptidase M precursor	2.4	1.2E-03
EPS8	epidermal growth factor receptor pathway substrate 8	2.3	1.2E-03

ETV4	ets variant 4	2.3	1.2E-03
NDRG1	N-myc downstream regulated 1	2.3	1.2E-03
ST6GAL2	beta-galactoside alpha-2,6-sialyltransferase 2	2.3	1.2E-03
ADCY8	adenylate cyclase 8 (brain)	2.3	1.2E-03
TRAK1	trafficking protein, kinesin binding 1	2.3	1.2E-03
TBCB	Tubulin-folding cofactor B**	2.2	1.2E-03
SLC26A5	prestin	2.2	1.2E-03
EFR3B	EFR3 homolog B (S. cerevisiae)	2.2	1.2E-03
KCNV1	potassium channel, subfamily V, member 1	2.2	1.2E-03
PRTG	protogenin precursor	2.2	1.2E-03
CRYGS	beta-crystallin S	2.2	1.2E-03
TAC1	Tachykinin 1	2.2	1.2E-03
MYO3B	myosin IIIB	2.2	1.2E-03
MUC5B	Mucin-5B	2.2	1.2E-03
FRAS1	Fraser syndrome 1	2.2	1.2E-03
POLH	polymerase (DNA directed), eta	2.1	1.2E-03
EGFR	Epidermal growth factor receptor	2.1	1.2E-03
PARP16	poly (ADP-ribose) polymerase family, member 16	2.1	1.2E-03
KIAA1644	Uncharacterized protein	2.1	1.2E-03
AGBL1	ATP/GTP binding protein-like 1	2.1	1.2E-03
NRIP1	nuclear receptor interacting protein 1	2.0	1.2E-03
LAMA5	laminin, alpha 5	2.0	1.2E-03
C7ORF63	Uncharacterized protein	2.0	1.2E-03
SLC16A5	solute carrier family 16, member 5 (monocarboxylic acid transporter 6)	2.0	1.2E-03
RSPO1	R-spondin 1	3.9	2.0E-03
FSHB	follitropin subunit beta precursor	2.9	2.0E-03
MOV10L1	*Mov10l1*, Moloney leukemia virus 10-like 1, homolog (mouse)	2.9	2.0E-03
ID2	DNA-binding protein inhibitor *ID-2*	2.5	2.0E-03
FSIP1	fibrous sheath interacting protein 1	2.4	2.0E-03
CAMK1G	calcium/calmodulin-dependent protein kinase IG	2.4	2.0E-03
GRK5	G protein-coupled receptor kinase 5	2.4	2.0E-03
KCTD12	Uncharacterized protein	2.2	2.0E-03
SPSB4	splA/ryanodine receptor domain and SOCS box containing 4	2.1	2.0E-03
HPCAL1	Hippocalcin-like protein 1	2.0	2.0E-03
ENSGALG00000005204	novel gene	2.7	2.8E-03
DNER	delta/notch-like EGF repeat containing	2.6	2.8E-03
CHST9	Uncharacterized protein	2.5	2.8E-03
OSBPL2	oxysterol-binding protein-related protein 2	2.4	2.8E-03
SCRN1	secernin 1	2.3	2.8E-03
C3ORF64	uncharacterized glycosyltransferase AER61	2.1	2.8E-03
S1PR1	sphingosine-1-phosphate receptor 1	2.0	2.8E-03
PRR5	proline-rich protein 5	2.0	2.8E-03
TRPC3	Uncharacterized protein	2.4	3.5E-03
HOMER3	homer homolog 3 (Drosophila)	2.1	3.5E-03
SPON2	spondin 2, extracellular matrix protein	2.0	3.5E-03
NDNF	neuron-derived neurotrophic factor	2.0	3.5E-03
ENSGALG00000027002	Uncharacterized protein	1.9	3.5E-03
MXRA5	matrix-remodelling associated 5	3.2	4.2E-03
ENSGALG00000022857	Uncharacterized protein	3.0	4.2E-03
FAM198B	family with sequence similarity 198, member B	3.0	4.2E-03
SCNN1B	sodium channel, non-voltage-gated 1, beta subunit	2.7	4.2E-03
PDGFRA	platelet-derived growth factor receptor alpha precursor	2.3	4.2E-03
THSD7A	thrombospondin, type I, domain containing 7A	2.2	4.2E-03
ENSGALG00000004078	novel gene	2.2	4.2E-03
CXCR7	chemokine (C-X-C motif) receptor 7	2.0	4.2E-03
ENSGALG00000004322	Uncharacterized protein	2.0	4.2E-03
KLHL36	kelch-like family member 36	1.8	4.2E-03
PTPRD	protein tyrosine phosphatase, receptor type, D	1.8	4.2E-03
GPAM	glycerol-3-phosphate acyltransferase, mitochondrial	1.8	4.2E-03
MRPL39	mitochondrial ribosomal protein L39	1.7	4.2E-03
EDG7	lysophosphatidic acid receptor 3	3.3	4.8E-03

EPHX4	epoxide hydrolase 4	2.6	4.8E-03
SEMA3G	sema domain, immunoglobulin domain (Ig), short basic domain, secreted, (semaphorin) 3G	2.5	4.8E-03
RGS6	regulator of G-protein signaling 6	2.0	4.8E-03
CDS1	Phosphatidate cytidylyltransferase	1.9	4.8E-03
PLCL2	phospholipase C-like 2	1.9	4.8E-03
STARD8	StAR-related lipid transfer (START) domain containing 8	4.2	5.4E-03
NRARP	NOTCH-regulated ankyrin repeat protein	3.2	5.4E-03
RBM24	RNA-binding protein 24	2.4	5.4E-03
NHS	Nance-Horan syndrome (congenital cataracts and dental anomalies)	2.2	5.4E-03
MYPN	myopalladin	2.1	5.4E-03
DCLK2	doublecortin-like kinase 2	1.9	5.4E-03
VTN	vitronectin precursor	1.8	5.4E-03
ENSGALG00000014164	Uncharacterized protein	1.8	5.4E-03
DGKB	diacylglycerol kinase, beta 90kDa	1.7	5.4E-03
DRAXIN	*Draxin*	3.0	6.0E-03
SARM1	sterile alpha and TIR motif containing 1	2.4	6.0E-03
KIF26B	kinesin family member 26B	2.0	6.0E-03
CRYBA4	beta-crystallin A4	2.0	6.0E-03
HES5	transcription factor *HES-5*	1.9	6.0E-03
DUSP16	dual specificity phosphatase 16	1.8	6.0E-03
CYP26A1	cytochrome P450 26A1	1.7	6.0E-03
PERP	*PERP*, TP53 apoptosis effector	2.7	6.5E-03
CRYGN	gamma-crystallin N	2.0	6.5E-03
MAP4K4	mitogen-activated protein kinase kinase kinase kinase 4	1.9	6.5E-03
OSBPL6	oxysterol binding protein-like 6	1.8	6.5E-03
TMEM56	transmembrane protein 56	1.6	6.5E-03
WNK2	WNK lysine deficient protein kinase 2	2.2	7.0E-03
HTR7	5-hydroxytryptamine (serotonin) receptor 7, adenylate cyclase-coupled	2.2	7.0E-03
SULF2	sulfatase 2	1.8	7.0E-03
CAPRIN2	caprin family member 2	1.8	7.0E-03
PLCXD1	PI-PLC X domain-containing protein 1	1.8	7.0E-03
CUX2	cut-like homeobox 2	1.7	7.0E-03
MYO3A	myosin IIIA	1.9	7.5E-03
TMCC1	transmembrane and coiled-coil domain family 1	1.7	7.5E-03
NTNG1	netrin G1	1.6	7.5E-03
ARSD	arylsulfatase D	2.7	8.0E-03
ELOVL7	elongation of very long chain fatty acids protein 7	2.1	8.0E-03
TBXAS1	Uncharacterized protein	2.5	8.5E-03
RUFY2	RUN and FYVE domain containing 2	2.2	8.5E-03
CARHSP1	Uncharacterized protein	1.8	8.5E-03
FABP3	fatty acid-binding protein, heart	3.4	9.0E-03
PID1	PTB-containing, cubilin and LRP1-interacting protein	2.0	9.0E-03
SAT1	Diamine acetyltransferase 1	1.7	9.0E-03
SMAD6	Mothers against decapentaplegic homolog 6	1.7	9.0E-03
gga-mir-1661	*gga-mir-1661* [Source:miRBase;Acc:MI0007395]	1.9	9.5E-03
CRCP	CGRP receptor component	1.9	9.5E-03
FREM1	FRAS1 related extracellular matrix 1	1.8	9.5E-03
COTL1	coactosin-like protein	2.8	1.0E-02
DKK3	dickkopf-related protein 3 precursor	2.6	1.0E-02
ATP2B2	ATPase, Ca++ transporting, plasma membrane 2	1.6	1.0E-02
COL27A1,ENSGALG00000025797	Uncharacterized protein	1.7	1.1E-02
TOX3	TOX high mobility group box family member 3	2.5	1.1E-02
PCNXL2	pecanex-like 2 (Drosophila)	1.8	1.1E-02
SCARB1	scavenger receptor class B, member 1	1.7	1.1E-02
TPGS2	tubulin polyglutamylase complex subunit 2	1.9	1.2E-02
RAPGEF1	Rap guanine nucleotide exchange factor (GEF) 1	1.8	1.2E-02
PARVB	parvin, beta	1.8	1.2E-02
THBS1	thrombospondin-1 precursor	2.6	1.2E-02
EPB41L2	erythrocyte membrane protein band 4.1-like 2	1.9	1.2E-02

PKDCC	protein kinase domain containing, cytoplasmic	1.8	1.2E-02
ENSGALG00000002150	novel gene	1.7	1.2E-02
EMILIN3	elastin microfibril interfacer 3	2.1	1.3E-02
ENSGALG00000026183	novel gene	1.7	1.3E-02
GAS2	growth arrest-specific protein 2	2.2	1.3E-02
GRAMD4	GRAM domain containing 4	1.8	1.3E-02
SESN1	sestrin 1	1.6	1.3E-02
ENSGALG00000027182	novel gene	1.6	1.3E-02
SASH1	SAM and SH3 domain containing 1	1.6	1.4E-02
ACTR3B	ARP3 actin-related protein 3 homolog B (yeast)	1.5	1.4E-02
PTGDS	prostaglandin D2 synthase, brain precursor	1.8	1.5E-02
VCPIP1	deubiquitinating protein VCIP135	1.7	1.5E-02
CCDC108	coiled-coil domain containing 108	1.6	1.5E-02
SLC4A7	solute carrier family 4, sodium bicarbonate cotransporter, member 7	1.6	1.5E-02
ENSGALG00000006407	death domain-containing tumor necrosis factor receptor superfamily member 23 precursor	2.5	1.5E-02
ENSGALG00000003644	novel gene	2.0	1.5E-02
NCKAP5	NCK-associated protein 5	1.9	1.5E-02
SHC2	SHC (Src homology 2 domain containing) transforming protein 2	1.7	1.5E-02
UGCG	UDP-glucose ceramide glucosyltransferase	1.6	1.5E-02
FBP1	fructose-1,6-bisphosphatase 1	2.1	1.6E-02
CRYBB1	Beta-crystallin B1	2.1	1.6E-02
BEND4	BEN domain containing 4	1.9	1.6E-02
UBE2Q2	ubiquitin-conjugating enzyme E2Q family member 2	1.9	1.6E-02
GYPC	glycophorin C (Gerbich blood group)	1.7	1.6E-02
ENSGALG00000005895	Uncharacterized protein	1.6	1.6E-02
MATN2	matrilin 2	1.9	1.6E-02
PPIC	peptidylprolyl isomerase C (cyclophilin C)	1.5	1.6E-02
DHX32	DEAH (Asp-Glu-Ala-His) box polypeptide 32	3.4	1.7E-02
FGFR2	Fibroblast growth factor receptor 2	1.9	1.7E-02
SESN3	sestrin 3	1.9	1.7E-02
RCBTB1	regulator of chromosome condensation (RCC1) and BTB (POZ) domain containing protein 1	1.7	1.7E-02
AGTPBP1	Cytosolic carboxypeptidase 1	1.6	1.7E-02
CDC14A	CDC14 cell division cycle 14 homolog A	1.6	1.7E-02
LHFP	Uncharacterized protein	1.6	1.7E-02
FXYD6	FXYD domain-containing ion transport regulator 6 precursor	1.5	1.7E-02
GPR137C	G protein-coupled receptor 137C	1.5	1.7E-02
TAGLN	Transgelin	1.7	1.8E-02
BCKDHB	2-oxoisovalerate dehydrogenase subunit beta, mitochondrial precursor	1.6	1.8E-02
SRP14	signal recognition particle 14 kDa protein	1.6	1.8E-02
JUN	transcription factor AP-1	1.5	1.9E-02
SMAD9	mothers against decapentaplegic homolog 9	2.6	1.9E-02
ZP4	zona pellucida sperm-binding protein 4	2.0	1.9E-02
NAV2	neuron navigator 2	1.8	1.9E-02
PROX2	prospero homeobox 2	2.1	1.9E-02
SYBU	syntabulin (syntaxin-interacting)	1.8	1.9E-02
CCNA1	cyclin A1	2.3	2.0E-02
KCNIP2	Kv channel-interacting protein 2	1.9	2.0E-02
NDRG4	NDRG family member 4	1.8	2.0E-02
PKP4	plakophilin-4	1.8	2.0E-02
PMP22	peripheral myelin protein 22	1.6	2.0E-02
GAMT	guanidinoacetate N-methyltransferase	1.5	2.0E-02
ENTPD3	ectonucleoside triphosphate diphosphohydrolase 3	2.2	2.0E-02
SH3RF3	SH3 domain containing ring finger 3	1.7	2.1E-02
CSK	tyrosine-protein kinase *CSK*	1.7	2.2E-02
GPRIN2	GRIN2-like protein	1.5	2.2E-02
SLC44A3	solute carrier family 44, member 3	1.4	2.2E-02
ENSGALG00000004279	lectin, galactoside-binding, soluble, 12	1.9	2.2E-02
STEAP2	STEAP family member 2, metalloreductase	1.9	2.2E-02

LMNA	lamin A/C	1.5	2.2E-02
FAM110B	family with sequence similarity 110, member B	1.7	2.3E-02
RPS6KL1	ribosomal protein S6 kinase-like 1	1.7	2.3E-02
TNNC1	Troponin C, slow skeletal and cardiac muscles	1.8	2.3E-02
MAP1B	microtubule-associated protein 1B	1.6	2.3E-02
SLC24A2	sodium/potassium/calcium exchanger 2	1.5	2.4E-02
MBOAT1	membrane bound O-acyltransferase domain containing 1	1.8	2.4E-02
TPH2	tryptophan 5-hydroxylase 2	1.6	2.4E-02
CAP2	Adenylyl cyclase-associated protein	1.6	2.4E-02
PLA2G10	phospholipase A2, group X	1.5	2.4E-02
GSTT1	Glutathione S-transferase theta-1	2.2	2.5E-02
DMN	synemin	1.8	2.5E-02
ENSGALG00000010722	Schwann cell-specific EGF-like repeat autocrine factor precursor	1.7	2.5E-02
CPEB4	cytoplasmic polyadenylation element binding protein 4	1.7	2.6E-02
TBC1D1	TBC1 (tre-2/USP6, BUB2, cdc16) domain family, member 1	1.7	2.6E-02
ZNF704	zinc finger protein 704	1.4	2.7E-02
NACAD	NAC alpha domain containing	1.9	2.7E-02
LURAP1	leucine rich adaptor protein 1	2.7	2.7E-02
MICALL2	MICAL-like 2	2.5	2.7E-02
SLC38A2	sodium-coupled neutral amino acid transporter 2	2.2	2.7E-02
AGPAT4	1-acylglycerol-3-phosphate O-acyltransferase 4	1.7	2.8E-02
NRCAM	neuronal cell adhesion molecule	1.4	2.8E-02
STARD5	StAR-related lipid transfer (START) domain containing 5	1.7	2.9E-02
FAM102A	early estrogen-induced gene 1 protein	1.7	2.9E-02
GLUL	glutamine synthetase	1.4	3.0E-02
HECW1	HECT, C2 and WW domain containing E3 ubiquitin protein ligase 1	1.4	3.0E-02
PABPC4	poly(A) binding protein, cytoplasmic 4 (inducible form)	1.4	3.0E-02
ARHGAP39	Rho GTPase activating protein 39	1.6	3.1E-02
PNPLA2	patatin-like phospholipase domain-containing protein 2	1.5	3.1E-02
PLTP	phospholipid transfer protein precursor	1.5	3.1E-02
NPY	Pro-neuropeptide Y Neuropeptide Y C-flanking peptide of *NPY*	3.9	3.1E-02
ARHGAP22	Rho GTPase activating protein 22	2.4	3.2E-02
TEF	transcription factor VBP	2.4	3.2E-02
SSFA2	sperm specific antigen 2	1.4	3.2E-02
LRRC1	leucine rich repeat containing 1	1.5	3.2E-02
MAP7	ensconsin	1.4	3.2E-02
AMER3	APC membrane recruitment protein 3	2.8	3.2E-02
EPHA6	EPH receptor A6	1.7	3.2E-02
NFKBIE	nuclear factor of kappa light polypeptide gene enhancer in B-cells inhibitor, epsilon	1.5	3.3E-02
SNORD37	Small nucleolar RNA *SNORD37*	1.4	3.3E-02
MID2	Midline 2; Uncharacterized protein	2.1	3.4E-02
ASS1	argininosuccinate synthase	2.2	3.4E-02
MAEA	Macrophage erythroblast attacher	1.6	3.4E-02
TP53INP1	tumor protein p53-inducible nuclear protein 1	1.4	3.4E-02
METTL7A	methyltransferase like 7A	2.0	3.4E-02
FAM124A	family with sequence similarity 124A	1.9	3.4E-02
CD99	*CD99* antigen precursor	1.8	3.4E-02
CYP26C1	cytochrome P450, family 26, subfamily C, polypeptide 1	1.5	3.5E-02
ENSGALG00000026539	novel gene	1.4	3.5E-02
FHOD3	formin homology 2 domain containing 3	1.5	3.5E-02
SEMA6A	sema domain, transmembrane domain (TM), and cytoplasmic domain, (semaphorin) 6A	1.5	3.5E-02
LRRC48	leucine rich repeat containing 48	1.7	3.5E-02
ENSGALG00000017332	novel gene	2.0	3.6E-02
NFIL3	nuclear factor interleukin-3-regulated protein	1.4	3.6E-02
SEMA4G	sema domain, immunoglobulin domain (Ig), transmembrane domain (TM) and short cytoplasmic domain, (semaphorin) 4G	1.4	3.6E-02
RBM38	RNA-binding protein 38	1.4	3.6E-02

TTC7B	tetratricopeptide repeat protein 7B	1.4	3.6E-02
OSGIN2	oxidative stress induced growth inhibitor family member 2	1.5	3.7E-02
OTUD7A	OTU domain containing 7A	1.4	3.7E-02
SGK3	serine/threonine-protein kinase Sgk3	1.4	3.7E-02
CNN3	calponin 3, acidic	1.7	3.8E-02
PHKA2	phosphorylase kinase, alpha 2 (liver)	1.5	3.8E-02
APAF1	apoptotic peptidase activating factor 1	1.4	3.8E-02
ARHGAP24	Rho GTPase activating protein 24	1.5	3.8E-02
SORCS1	sortilin-related VPS10 domain containing receptor 1	1.4	3.8E-02
DAAM2	dishevelled associated activator of morphogenesis 2	1.6	3.9E-02
MSANTD3	Myb/SANT-like DNA-binding domain containing 3	1.4	3.9E-02
PIK3CA	phosphatidylinositol-4,5-bisphosphate 3-kinase catalytic subunit alpha isoform	1.3	3.9E-02
TUBB6	Tubulin beta-5 chain	1.4	3.9E-02
ENSGALG00000021838	novel gene	1.6	4.0E-02
ARHGAP10	Rho GTPase activating protein 10	1.4	4.1E-02
CAB39L	calcium binding protein 39-like	1.9	4.1E-02
FSD1L	fibronectin type III and SPRY domain containing 1-like	1.5	4.2E-02
TRIB1	tribbles homolog 1 (Drosophila)	1.5	4.2E-02
KCTD1	potassium channel tetramerization domain containing 1	1.5	4.2E-02
RASSF2	ras association domain-containing protein 2	1.5	4.2E-02
GAS7	growth arrest-specific 7	1.5	4.3E-02
SERPINB6	serpin B6	1.4	4.4E-02
CABP7	calcium binding protein 7	1.6	4.5E-02
EMP2	epithelial membrane protein 2	1.3	4.5E-02
ENSGALG00000004676	Uncharacterized protein	1.4	4.5E-02
RNaseP_nuc	Nuclear RNase P	1.9	4.6E-02
KIF25	kinesin family member 25	1.5	4.6E-02
ACAP3	ArfGAP with coiled-coil, ankyrin repeat and PH domains 3	1.4	4.7E-02
EDN3	Endothelin 3	1.6	4.8E-02
DUSP8	dual specificity phosphatase 8	1.7	4.8E-02
gga-let-7b	*gga-let-7b* [Source:miRBase;Acc:MI0001172]	1.5	4.9E-02
ENSGALG00000028383	novel gene	1.7	5.0E-02
TMEM135	transmembrane protein 135	1.3	5.0E-02

*p-values are corrected for multiple testing by the false discovery rate method as utilized by cuffdiff (version 2.1.1).

Table S3 Detected EQ gene-specific transcripts statistically decreased in expression during EQ to FP transition.

Gene	Description	log2(Fold Change)	p-value*
CC2D2A	coiled-coil and C2 domain containing 2A	-2.0	1.2E-03
INSIG2	insulin induced gene 2	-2.0	1.2E-03
ODZ2	teneurin transmembrane protein 2	-2.0	1.2E-03
SEPHS1	selenophosphate synthetase 1	-2.0	1.2E-03
B4GALT6	UDP-Gal:betaGlcNAc beta 1,4- galactosyltransferase, polypeptide 6	-2.0	1.2E-03
CDC42SE2	CDC42 small effector 2	-2.0	1.2E-03
SLIT3	slit homolog 3 (Drosophila)	-2.1	1.2E-03
FKBP9	FK506 binding protein 9, 63 kDa	-2.1	1.2E-03
ATAD2	ATPase family, AAA domain containing 2	-2.1	1.2E-03
PURH	5-aminoimidazole-4-carboxamide ribonucleotide formyltransferase/IMP cyclohydrolase	-2.1	1.2E-03
PLXNA2	plexin A2	-2.1	1.2E-03
CSRNP1	cysteine-serine-rich nuclear protein 1	-2.1	1.2E-03
PER2	period circadian clock 2	-2.1	1.2E-03
CERK	ceramide kinase	-2.1	1.2E-03
NRSN1	neurensin 1	-2.1	1.2E-03
C1H21orf33	ES1 protein homolog, mitochondrial	-2.1	1.2E-03
REPS2	RALBP1 associated Eps domain containing 2	-2.2	1.2E-03
TPX2	*TPX2*, microtubule-associated, homolog (Xenopus laevis)	-2.2	1.2E-03
PPIC	peptidylprolyl isomerase C (cyclophilin C)	-2.2	1.2E-03
GNG10	guanine nucleotide binding protein (G protein), gamma 10	-2.2	1.2E-03
PHF16	PHD finger protein 16	-2.2	1.2E-03
TMEM108	transmembrane protein 108	-2.2	1.2E-03
MCAM	melanoma cell adhesion molecule	-2.2	1.2E-03
TLL1	tolloid-like 1	-2.2	1.2E-03
TMEM194B	transmembrane protein 194B	-2.2	1.2E-03
PIWIL1	piwi-like RNA-mediated gene silencing 1	-2.2	1.2E-03
SORCS1	sortilin-related VPS10 domain containing receptor 1	-2.2	1.2E-03
MKI67	antigen identified by monoclonal antibody Ki-67	-2.2	1.2E-03
C1H12ORF23	UPF0444 transmembrane protein C12orf23 homolog	-2.2	1.2E-03
CCDC108	coiled-coil domain containing 108	-2.2	1.2E-03
RYK	receptor-like tyrosine kinase	-2.2	1.2E-03
CMTM3	CKLF-like MARVEL transmembrane domain containing 3	-2.2	1.2E-03
LACTB2	lactamase, beta 2	-2.2	1.2E-03
KPNA2	karyopherin alpha 2 (RAG cohort 1, importin alpha 1)	-2.2	1.2E-03
ODZ3	teneurin-3	-2.2	1.2E-03
NDST2	N-deacetylase/N-sulfotransferase (heparan glucosaminyl) 2	-2.2	1.2E-03
TRAF4	TNF receptor-associated factor 4	-2.2	1.2E-03
FGF19	fibroblast growth factor 19	-2.2	1.2E-03
PLXNC1	plexin C1	-2.2	1.2E-03
CENPF	centromere protein F, 350/400kDa	-2.2	1.2E-03
ANKH	*ANKH* inorganic pyrophosphate transport regulator	-2.3	1.2E-03
SRP14	signal recognition particle 14kDa (homologous Alu RNA binding protein)	-2.3	1.2E-03
CHST14	carbohydrate (N-acetylgalactosamine 4-0) sulfotransferase 14	-2.3	1.2E-03
NBEAL2	neurobeachin-like 2	-2.3	1.2E-03
LAMA4	laminin, alpha 4	-2.3	1.2E-03
CABLES1	Cdk5 and Abl enzyme substrate 1	-2.3	1.2E-03
CBS	cystathionine-beta-synthase	-2.3	1.2E-03
OLFM3	olfactomedin 3	-2.3	1.2E-03
PAM	peptidylglycine alpha-amidating monooxygenase	-2.3	1.2E-03
P4HA3	prolyl 4-hydroxylase, alpha polypeptide III	-2.3	1.2E-03
HNRNPA0	heterogeneous nuclear ribonucleoprotein A0	-2.3	1.2E-03
BTG2	BTG family, member 2	-2.3	1.2E-03
SOBP	sine oculis binding protein homolog (Drosophila)	-2.3	1.2E-03
CCDC88C	coiled-coil domain containing 88C	-2.3	1.2E-03

SEC61A1	Sec61 alpha 1 subunit (S. cerevisiae)	-2.3	1.2E-03
METRNL	meteorin, glial cell differentiation regulator-like	-2.3	1.2E-03
SPINT1	serine peptidase inhibitor, Kunitz type 1	-2.3	1.2E-03
ROR1	receptor tyrosine kinase-like orphan receptor 1	-2.3	1.2E-03
GCHFR	GTP cyclohydrolase I feedback regulator	-2.3	1.2E-03
GM2A	GM2 ganglioside activator	-2.3	1.2E-03
SH3D19	SH3 domain containing 19	-2.3	1.2E-03
PRKX	protein kinase, X-linked	-2.4	1.2E-03
RND3	Rho family GTPase 3	-2.4	1.2E-03
TJP2	tight junction protein 2	-2.4	1.2E-03
ATP2B4	ATPase, Ca++ transporting, plasma membrane 4	-2.4	1.2E-03
NAB1	NGFI-A binding protein 1 (EGR1 binding protein 1)	-2.4	1.2E-03
TNS3	tensin 3	-2.4	1.2E-03
CCDC109B	coiled-coil domain containing 109B	-2.4	1.2E-03
TCTN3	tectonic family member 3	-2.4	1.2E-03
ZNF536	zinc finger protein 536	-2.4	1.2E-03
HKDC1	hexokinase domain containing 1	-2.4	1.2E-03
ANKRD50	ankyrin repeat domain 50	-2.4	1.2E-03
TMEM16E	anoctamin 5	-2.4	1.2E-03
SLC29A1	solute carrier family 29 (equilibrative nucleoside transporter), member 1	-2.4	1.2E-03
CASKIN2	CASK interacting protein 2	-2.4	1.2E-03
EXOG	endo/exonuclease (5,-3,), endonuclease G-like	-2.4	1.2E-03
PLOD2	procollagen-lysine, 2-oxoglutarate 5-dioxygenase 2	-2.4	1.2E-03
EGFR	epidermal growth factor receptor	-2.4	1.2E-03
C16ORF45	chromosome 16 open reading frame 45	-2.4	1.2E-03
DLG2	discs, large homolog 2 (Drosophila)	-2.4	1.2E-03
RRM2	ribonucleotide reductase M2	-2.5	1.2E-03
GPRIN2	G protein regulated inducer of neurite outgrowth 2	-2.5	1.2E-03
FAM69A	family with sequence similarity 69, member A	-2.5	1.2E-03
CTH	cystathionase (cystathionine gamma-lyase)	-2.5	1.2E-03
GLUR1/A	glutamate receptor 1 precursor	-2.5	1.2E-03
SHISA2	shisa homolog 2 (Xenopus laevis)	-2.5	1.2E-03
PLA2G10	phospholipase A2, group X	-2.5	1.2E-03
RBL1	retinoblastoma-like 1 (p107)	-2.5	1.2E-03
DUT	deoxyuridine 5,-triphosphate nucleotidohydrolase, mitochondrial	-2.5	1.2E-03
TP53INP1	tumor protein p53 inducible nuclear protein 1	-2.5	1.2E-03
FNDC3B	fibronectin type III domain containing 3B	-2.5	1.2E-03
CKAP2	cytoskeleton associated protein 2	-2.5	1.2E-03
ITPKA	inositol-trisphosphate 3-kinase A	-2.5	1.2E-03
GALNTL1	Uncharacterized protein	-2.5	1.2E-03
HNRPK	heterogeneous nuclear ribonucleoprotein K	-2.5	1.2E-03
EMILIN2	elastin microfibril interfacer 2	-2.5	1.2E-03
FAM43A	family with sequence similarity 43, member A	-2.5	1.2E-03
GCH1	GTP cyclohydrolase 1	-2.5	1.2E-03
GRM4	glutamate receptor, metabotropic 4	-2.5	1.2E-03
FAM171A1	family with sequence similarity 171, member A1	-2.5	1.2E-03
CASZ1	castor zinc finger 1	-2.5	1.2E-03
SCNN1A	amiloride-sensitive sodium channel subunit alpha	-2.5	1.2E-03
SMC2	structural maintenance of chromosomes protein 2	-2.6	1.2E-03
ENSGALG00000009516	transforming, acidic coiled-coil containing protein 2	-2.6	1.2E-03
FRMD4B	FERM domain containing 4B	-2.6	1.2E-03
SLC38A5	solute carrier family 38, member 5	-2.6	1.2E-03
EPB41L5	erythrocyte membrane protein band 4.1 like 5	-2.6	1.2E-03
MGST1	microsomal glutathione S-transferase 1	-2.6	1.2E-03
VPS37B	vacuolar protein sorting 37 homolog B (S. cerevisiae)	-2.6	1.2E-03
LAMA3	laminin, alpha 3	-2.6	1.2E-03
C2ORF40	Uncharacterized protein	-2.6	1.2E-03
KERA	Keratocan	-2.6	1.2E-03
PRR5	proline-rich protein 5	-2.6	1.2E-03
MID1	midline 1 (Opitz/BBB syndrome)	-2.6	1.2E-03
UGDH	UDP-glucose 6-dehydrogenase	-2.6	1.2E-03

PLVAP	plasmalemma vesicle associated protein	-2.6	1.2E-03
ENSGALG00000005727	solute carrier family 7 (cationic amino acid transporter, y+ system), member 3	-2.6	1.2E-03
ENSGALG00000028602	novel gene	-2.6	1.2E-03
DLGAP5	discs, large (Drosophila) homolog-associated protein 5	-2.6	1.2E-03
HES4	transcription factor HES-1 isoform 2	-2.6	1.2E-03
CXCR7	chemokine (C-X-C motif) receptor 7	-2.6	1.2E-03
RBP5	retinol binding protein 5, cellular	-2.6	1.2E-03
APAF1	apoptotic peptidase activating factor 1	-2.6	1.2E-03
UAP1L1	UDP-N-acteylglucosamine pyrophosphorylase 1-like 1	-2.6	1.2E-03
COL4A1	collagen alpha-1(IV) chain precursor	-2.6	1.2E-03
PLCL2	phospholipase C-like 2	-2.6	1.2E-03
PLCB4	1-phosphatidylinositol-4,5-bisphosphate phosphodiesterase beta-4	-2.7	1.2E-03
ADAMTS17	ADAM metallopeptidase with thrombospondin type 1 motif, 17	-2.7	1.2E-03
MYRF	myelin regulatory factor	-2.7	1.2E-03
ABCC9	ATP-binding cassette, sub-family C (CFTR/MRP), member 9	-2.7	1.2E-03
PLD1	phospholipase D1, phosphatidylcholine-specific	-2.7	1.2E-03
CNFI-A4	nuclear factor 1 A-type	-2.7	1.2E-03
RLBP1	retinaldehyde-binding protein 1	-2.7	1.2E-03
HMCN1	hemicentin 1	-2.7	1.2E-03
KAT6B	K(lysine) acetyltransferase 6B	-2.7	1.2E-03
ATP2B1	plasma membrane calcium-transporting ATPase 1	-2.7	1.2E-03
ENSGALG00000016322	glutathione S-transferase 3	-2.7	1.2E-03
PHLDB2	pleckstrin homology-like domain, family B, member 2	-2.7	1.2E-03
TENM4	teneurin transmembrane protein 4	-2.7	1.2E-03
LEF-1	lymphoid enhancer-binding factor *1*	-2.7	1.2E-03
SLC12A2	solute carrier family 12	-2.7	1.2E-03
ASTN1	astrotactin 1	-2.7	1.2E-03
RERG	RAS-like, estrogen-regulated, growth inhibitor	-2.7	1.2E-03
COL27A1,ENSGALG00000025797	Uncharacterized protein	-2.7	1.2E-03
MXRA8	Matrix-remodeling-associated protein 8	-2.7	1.2E-03
SCARB2	scavenger receptor class B, member 2	-2.7	1.2E-03
CDC2	cyclin-dependent kinase 1	-2.8	1.2E-03
RELL1	RELT-like protein 1	-2.8	1.2E-03
FOXP1	Forkhead box protein P1	-2.8	1.2E-03
ANXA8	Annexin	-2.8	1.2E-03
41528		-2.8	1.2E-03
PDK1	pyruvate dehydrogenase kinase, isozyme 1	-2.8	1.2E-03
HMGB2	high mobility group protein B2	-2.8	1.2E-03
LIPG	lipase, endothelial	-2.8	1.2E-03
SLC38A1	sodium-coupled neutral amino acid transporter 1	-2.8	1.2E-03
TNC	tenascin precursor	-2.8	1.2E-03
GXYLT2	glucoside xylosyltransferase 2	-2.8	1.2E-03
SLC38A11	solute carrier family 38, member 11	-2.8	1.2E-03
NMI	N-myc (and STAT) interactor	-2.8	1.2E-03
SLC16A12	solute carrier family 16, member 12 (monocarboxylic acid transporter 12)	-2.8	1.2E-03
NKD1	naked cuticle homolog 1 (Drosophila)	-2.8	1.2E-03
FGFR2	Fibroblast growth factor receptor 2	-2.8	1.2E-03
ISLR	immunoglobulin superfamily containing leucine-rich repeat	-2.8	1.2E-03
MAML2	mastermind-like 2 (Drosophila)	-2.8	1.2E-03
AADAC	arylacetamide deacetylase	-2.8	1.2E-03
RGMA	RGM domain family, member A	-2.8	1.2E-03
KIAA1644	*KIAA1644*	-2.8	1.2E-03
ABI3BP	ABI family, member 3 (NESH) binding protein	-2.9	1.2E-03
CITED3	Cbp/p300-interacting transactivator 3	-2.9	1.2E-03
C1orf198	chromosome 1 open reading frame 198	-2.9	1.2E-03
ARL4A	ADP-ribosylation factor-like 4A	-2.9	1.2E-03
PRPS2	phosphoribosyl pyrophosphate synthetase 2	-2.9	1.2E-03
NCKAP5	NCK-associated protein 5	-2.9	1.2E-03

TMEM200C	transmembrane protein 200C	-2.9	1.2E-03
ENSGALG00000000489	nascent polypeptide-associated complex alpha subunit 2	-2.9	1.2E-03
OPTC	opticin precursor	-2.9	1.2E-03
SEMA3C	semaphorin-3C precursor	-2.9	1.2E-03
SARDH	sarcosine dehydrogenase	-2.9	1.2E-03
CDK14	cyclin-dependent kinase 14	-2.9	1.2E-03
CPZ	carboxypeptidase Z	-2.9	1.2E-03
NFIB	nuclear factor I/B	-2.9	1.2E-03
MUC5B	mucin 5B, oligomeric mucus/gel-forming	-2.9	1.2E-03
FRMPD4	FERM and PDZ domain containing 4	-2.9	1.2E-03
PALLD	palladin, cytoskeletal associated protein	-3.0	1.2E-03
FAM207A	family with sequence similarity 207, member A	-3.0	1.2E-03
CD109	*CD109* molecule	-3.0	1.2E-03
MOV10L1	*Mov10l1*, Moloney leukemia virus 10-like 1, homolog (mouse)	-3.0	1.2E-03
FHL3	four and a half LIM domains 3	-3.0	1.2E-03
PDGFRL	platelet-derived growth factor receptor-like	-3.0	1.2E-03
NOTCH2	*notch 2*	-3.0	1.2E-03
SLC16A1	solute carrier family 16, member 1 (monocarboxylic acid transporter 1)	-3.0	1.2E-03
LTBP1	latent transforming growth factor beta binding protein 1	-3.0	1.2E-03
GABRB4	Gamma-aminobutyric acid receptor subunit beta-4	-3.0	1.2E-03
PLXNB2	plexin B2	-3.1	1.2E-03
PPP1R14C	protein phosphatase 1, regulatory (inhibitor) subunit 14C	-3.1	1.2E-03
C1QTNF6	C1q and tumor necrosis factor related protein 6	-3.1	1.2E-03
CSRP2	Cysteine and glycine-rich protein 2	-3.1	1.2E-03
CD81	*CD81* antigen	-3.1	1.2E-03
ADAMTS13	ADAM metallopeptidase with thrombospondin type 1 motif, 13	-3.1	1.2E-03
SOHO-1	sensory organ homeobox protein *SOHo*	-3.1	1.2E-03
TBC1D1	TBC1 (tre-2/USP6, BUB2, cdc16) domain family, member 1	-3.1	1.2E-03
ALPL	alkaline phosphatase, liver/bone/kidney	-3.1	1.2E-03
ANGPTL4	angiopoietin-like 4	-3.1	1.2E-03
TRPC3	transient receptor potential cation channel, subfamily C, member 3	-3.1	1.2E-03
ENSGALG00000027183	novel gene	-3.1	1.2E-03
SEC24D	SEC24 family, member D (S. cerevisiae)	-3.1	1.2E-03
EYA1	Eyes absent homolog 1	-3.1	1.2E-03
FREM1	FRAS1 related extracellular matrix 1	-3.1	1.2E-03
C7ORF63	Uncharacterized protein	-3.1	1.2E-03
ID2	DNA-binding protein inhibitor *ID-2*	-3.2	1.2E-03
SLC2A12	solute carrier family 2 (facilitated glucose transporter), member 12	-3.2	1.2E-03
OLFML3	Olfactomedin-like protein 3	-3.2	1.2E-03
CYP26A1	cytochrome P450 26A1	-3.2	1.2E-03
PLCXD1	PI-PLC X domain-containing protein 1	-3.2	1.2E-03
PSAT1	phosphoserine aminotransferase 1	-3.2	1.2E-03
WEE1	*wee1*-like protein kinase	-3.2	1.2E-03
NFKBIE	nuclear factor of kappa light polypeptide gene enhancer in B-cells inhibitor, epsilon	-3.2	1.2E-03
gga-let-7b	*gga-let-7b* [Source:miRBase;Acc:MI0001172]	-3.2	1.2E-03
ENPP6	ectonucleotide pyrophosphatase/phosphodiesterase 6	-3.2	1.2E-03
FAM110B	family with sequence similarity 110, member B	-3.3	1.2E-03
APOD	apolipoprotein D precursor	-3.3	1.2E-03
COL11A1	collagen, type XI, alpha 1	-3.3	1.2E-03
ENSGALG00000005344	Uncharacterized protein	-3.3	1.2E-03
PDGFRA	platelet-derived growth factor receptor alpha precursor	-3.3	1.2E-03
QPCT	glutaminyl-peptide cyclotransferase	-3.3	1.2E-03
SLC26A7	solute carrier family 26, member 7	-3.3	1.2E-03
ENSGALG00000028153	forkhead box E1 (thyroid transcription factor 2)	-3.3	1.2E-03
LRP1B	low density lipoprotein receptor-related protein 1	-3.3	1.2E-03
MAOA	monoamine oxidase A	-3.4	1.2E-03
GLUL	glutamine synthetase	-3.4	1.2E-03

SLC12A3	solute carrier family 12 (sodium/chloride transporters), member 3	-3.4	1.2E-03
DEGS1	Sphingolipid delta(4)-desaturase DES1	-3.4	1.2E-03
ADAMTSL3	ADAMTS-like 3	-3.4	1.2E-03
TDH	L-threonine dehydrogenase	-3.4	1.2E-03
PID1	PTB-containing, cubilin and LRP1-interacting protein	-3.4	1.2E-03
PCBP3	poly(rC) binding protein 3	-3.4	1.2E-03
TAC1	Tachykinin 1	-3.4	1.2E-03
LGALS3	Galectin-3	-3.4	1.2E-03
CLVS2	clavesin 2	-3.4	1.2E-03
TSKU	tsukushin precursor	-3.4	1.2E-03
CNTFR	Ciliary neurotrophic factor receptor subunit alpha	-3.4	1.2E-03
SLC6A6	sodium- and chloride-dependent taurine transporter	-3.4	1.2E-03
GRIN3A	glutamate receptor, ionotropic, N-methyl-D-aspartate 3A	-3.5	1.2E-03
SLC38A2	sodium-coupled neutral amino acid transporter 2	-3.5	1.2E-03
SOUL	*SOUL* protein	-3.5	1.2E-03
FSHB	follitropin subunit beta precursor	-3.6	1.2E-03
ABCA4	ATP-binding cassette, sub-family A (ABC1), member 4	-3.6	1.2E-03
COL24A1	collagen, type XXIV, alpha 1	-3.6	1.2E-03
RELN	reelin	-3.7	1.2E-03
SLC7A2	low affinity cationic amino acid transporter 2	-3.7	1.2E-03
ENSGALG00000027002	Uncharacterized protein	-3.7	1.2E-03
HEY1	hairy/enhancer-of-split related with YRPW motif 1	-3.7	1.2E-03
7SK	*7SK* RNA	-3.8	1.2E-03
NDNF	neuron-derived neurotrophic factor	-3.8	1.2E-03
KCNV1	potassium channel, subfamily V, member 1	-3.8	1.2E-03
NET1	neuroepithelial cell transforming 1	-3.9	1.2E-03
KCTD12	potassium channel tetramerization domain containing 12	-3.9	1.2E-03
ENSGALG00000004322	Uncharacterized protein	-3.9	1.2E-03
ENSGALG00000001136	Uncharacterized protein	-3.9	1.2E-03
STEAP1	six transmembrane epithelial antigen of the prostate 1	-4.0	1.2E-03
HEYL	hairy/enhancer-of-split related with YRPW motif-like	-4.0	1.2E-03
FAM198B	family with sequence similarity 198, member B	-4.2	1.2E-03
HGD	homogentisate 1,2-dioxygenase	-4.3	1.2E-03
HES5	transcription factor *HES-5*	-4.3	1.2E-03
CYP1B1	cytochrome P450, family 1, subfamily B, polypeptide 1	-4.3	1.2E-03
STARD8	StAR-related lipid transfer (START) domain containing 8	-5.4	1.2E-03
MIS12	*MIS12* kinetochore complex component	/0	1.2E-03
ADAMTS6	ADAM metallopeptidase with thrombospondin type 1 motif, 6	-1.8	2.0E-03
PGAP1	post-GPI attachment to proteins 1	-1.9	2.0E-03
TMX4	thioredoxin-related transmembrane protein 4 precursor	-2.0	2.0E-03
CDH11	cadherin-11 precursor	-2.0	2.0E-03
PLTP	phospholipid transfer protein precursor	-2.0	2.0E-03
JAZF1	juxtaposed with another zinc finger protein 1	-2.0	2.0E-03
ACSL1	long-chain-fatty-acid--CoA ligase 1	-2.0	2.0E-03
SNX25	sorting nexin 25	-2.0	2.0E-03
FRY	furry homolog (Drosophila)	-2.0	2.0E-03
TBC1D9	TBC1 domain family, member 9 (with GRAM domain)	-2.1	2.0E-03
SHF	Src homology 2 domain containing F	-2.1	2.0E-03
ENSGALG00000029174	novel gene	-2.1	2.0E-03
CRY1	cryptochrome-1	-2.1	2.0E-03
LGR4	leucine-rich repeat containing G protein-coupled receptor 4	-2.2	2.0E-03
ROR2	tyrosine-protein kinase transmembrane receptor *ROR2* precursor	-2.2	2.0E-03
CDCA7L	cell division cycle-associated 7-like protein	-2.2	2.0E-03
MMD	monocyte to macrophage differentiation protein [	-2.2	2.0E-03
MDK	midkine precursor	-2.2	2.0E-03
CHRDL1	chordin-like protein 1 precursor	-2.2	2.0E-03
ID3	inhibitor of DNA binding 3	-2.2	2.0E-03
IGSF3	immunoglobulin superfamily, member 3	-2.2	2.0E-03
DDOST	dolichyl-diphosphooligosaccharide--protein glycosyltransferase 48 kDa subunit	-2.2	2.0E-03

NAPRT1	nicotinate phosphoribosyltransferase domain containing 1	-2.2	2.0E-03
PLCG1	phospholipase C, gamma 1	-2.3	2.0E-03
ALDH18A1	aldehyde dehydrogenase 18 family, member A1	-2.3	2.0E-03
NOTCH1	*notch 1*	-2.3	2.0E-03
NFIL3	nuclear factor interleukin-3-regulated protein	-2.3	2.0E-03
MYD88	Myeloid differentiation primary response protein MyD88	-2.3	2.0E-03
DAB2	Dab, mitogen-responsive phosphoprotein, homolog 2	-2.3	2.0E-03
GPC4	glypican 4	-2.4	2.0E-03
TGIF2	TGFB-induced factor homeobox 2	-2.4	2.0E-03
ADORA2A	adenosine A2a receptor	-2.4	2.0E-03
CPM	carboxypeptidase M precursor	-2.4	2.0E-03
UHRF1BP1	UHRF1 binding protein 1	-2.4	2.0E-03
XRCC5	X-ray repair complementing defective repair in Chinese hamster cells 5 (double-strand-break rejoining)	-2.5	2.0E-03
TRAM2	translocation associated membrane protein 2	-2.5	2.0E-03
SH3BGRL	SH3 domain-binding glutamic acid-rich-like protein	-2.5	2.0E-03
ATP11A	ATPase, class VI, type 11A	-2.5	2.0E-03
GK5	Putative glycerol kinase 5	-2.5	2.0E-03
PAK1	serine/threonine-protein kinase *PAK 1*	-2.6	2.0E-03
EFHD1	EF-hand domain-containing protein D1	-2.6	2.0E-03
EPHB6	ephrin type-B receptor 5 precursor	-2.7	2.0E-03
MYO5A	myosin-Va	-3.0	2.0E-03
STYK1	serine/threonine/tyrosine kinase 1	-3.0	2.0E-03
AQP4	aquaporin-4	-3.4	2.0E-03
VAPA	vesicle-associated membrane protein-associated protein A	-1.8	2.8E-03
LNPEP	leucyl/cystinyl aminopeptidase	-2.0	2.8E-03
RP2	Protein XRP2	-2.0	2.8E-03
DPY19L3	dpy-19-like 3 (C. elegans)	-2.0	2.8E-03
GLT8D1	glycosyltransferase 8 domain-containing protein 1	-2.1	2.8E-03
TMPO	lamina-associated polypeptide 2, isoform beta	-2.1	2.8E-03
TXNDC5	thioredoxin domain-containing protein 5 precursor	-2.1	2.8E-03
ENSGALG00000004676	Uncharacterized protein	-2.2	2.8E-03
ARHGEF17	Rho guanine nucleotide exchange factor (GEF) 17	-2.2	2.8E-03
CDC45	cell division cycle 45	-2.2	2.8E-03
AP1AR	adaptor-related protein complex 1 associated regulatory protein	-2.2	2.8E-03
PYCR2	pyrroline-5-carboxylate reductase family, member 2	-2.3	2.8E-03
BOC	*BOC* cell adhesion associated, oncogene regulated	-2.3	2.8E-03
MANSC1	MANSC domain containing 1 precursor	-2.3	2.8E-03
ATP10D	ATPase, class V, type 10D	-2.5	2.8E-03
ENSGALG00000027352	novel gene	-2.5	2.8E-03
HSPB2	heat shock 27kDa protein 2	-2.6	2.8E-03
MAP4K4	mitogen-activated protein kinase kinase kinase kinase 4	-2.6	2.8E-03
ATP1A1	sodium/potassium-transporting ATPase subunit alpha-1 precursor	-2.8	2.8E-03
EMILIN3	elastin microfibril interfacer 3	-2.9	2.8E-03
ENSGALG00000005204	novel gene	-3.0	2.8E-03
TEF	transcription factor VBP	-3.4	2.8E-03
CER1	cerberus 1, DAN family BMP antagonist	-3.8	2.8E-03
gga-mir-1661	*gga-mir-1661* [Source:miRBase;Acc:MI0007395]	/0	2.8E-03
BCKDHB	2-oxoisovalerate dehydrogenase subunit beta, mitochondrial precursor	-1.8	3.5E-03
SPSB1	splA/ryanodine receptor domain and SOCS box containing 1	-1.9	3.5E-03
LRP2	low density lipoprotein receptor-related protein 2	-1.9	3.5E-03
GNPDA2	Glucosamine-6-phosphate deaminase 2 isoform 1	-1.9	3.5E-03
DOCK1	dedicator of cytokinesis 1	-1.9	3.5E-03
MFSD7	major facilitator superfamily domain containing 7	-2.0	3.5E-03
THSD7B	thrombospondin, type I, domain containing 7B	-2.0	3.5E-03
KIF15	Uncharacterized protein	-2.0	3.5E-03
PTBP1	polypyrimidine tract-binding protein 1	-2.0	3.5E-03
PODXL	podocalyxin-like	-2.1	3.5E-03
OMA1	*OMA1* zinc metallopeptidase	-2.1	3.5E-03
ARL5B	ADP-ribosylation factor-like 5B	-2.1	3.5E-03

DENND2A	DENN/MADD domain containing 2A	-2.1	3.5E-03
FRMD3	FERM domain containing 3	-2.1	3.5E-03
VAT1	vesicle amine transport protein 1 homolog (T. californica)	-2.2	3.5E-03
MTUS1	microtubule associated tumor suppressor 1	-2.2	3.5E-03
MCTP2	multiple C2 domains, transmembrane 2	-2.2	3.5E-03
BCL6	B-cell CLL/lymphoma 6	-2.2	3.5E-03
FAM102A	family with sequence similarity 102, member A	-2.2	3.5E-03
CYTH1	cytohesin-1	-2.2	3.5E-03
MYCN	N-myc proto-oncogene protein	-2.3	3.5E-03
TNFIP6	tumor necrosis factor-inducible gene 6 protein precursor	-2.3	3.5E-03
WNK2	WNK lysine deficient protein kinase 2	-2.4	3.5E-03
FBN1	fibrillin 1	-2.4	3.5E-03
ST3GAL6	ype 2 lactosamine alpha-2,3-sialyltransferase	-2.5	3.5E-03
PROCR	endothelial protein C receptor precursor	-2.5	3.5E-03
C12orf50	chromosome 12 open reading frame 50	-2.6	3.5E-03
PGM5	phosphoglucomutase 5	-2.6	3.5E-03
CENPE	centromere protein E, 312kDa	-2.6	3.5E-03
CHRAC1	chromatin accessibility complex 1	-2.7	3.5E-03
TACC3	transforming, acidic coiled-coil containing protein 3	-2.7	3.5E-03
TYMS	Thymidylate synthase	-2.8	3.5E-03
DAAM2	dishevelled associated activator of morphogenesis 2	-2.9	3.5E-03
CHST9	carbohydrate (N-acetylgalactosamine 4-0) sulfotransferase 9	-3.1	3.5E-03
C1R	complement component 1, r subcomponent	-3.1	3.5E-03
CDH6	Cadherin-6	-3.1	3.5E-03
CYP26C1	cytochrome P450, family 26, subfamily C, polypeptide 1	-3.5	3.5E-03
ZNF385D	zinc finger protein 385D	-3.5	3.5E-03
MXRA5	matrix-remodelling associated 5	-3.6	3.5E-03
CISH	Cytokine-inducible SH2-containing protein	-1.8	4.2E-03
FBXL2	F-box and leucine-rich repeat protein 2	-1.9	4.2E-03
BUB1	*BUB1* mitotic checkpoint serine/threonine kinase	-1.9	4.2E-03
SMCHD1	structural maintenance of chromosomes flexible hinge domain containing 1	-1.9	4.2E-03
PANK1	pantothenate kinase 1	-1.9	4.2E-03
FAM177A1	family with sequence similarity 177, member A1	-1.9	4.2E-03
CBX1	chromobox protein homolog 1	-1.9	4.2E-03
SETBP1	SET binding protein 1	-1.9	4.2E-03
ACTL6A	actin-like 6A	-1.9	4.2E-03
MPP6	membrane protein, palmitoylated 6 (MAGUK p55 subfamily member 6)	-2.0	4.2E-03
PIGA	N-acetylglucosaminyl-phosphatidylinositol biosynthetic protein	-2.0	4.2E-03
ABL1	c-abl oncogene 1, non-receptor tyrosine kinase	-2.0	4.2E-03
ZNHIT6	zinc finger, HIT-type containing 6	-2.0	4.2E-03
SLC31A1	solute carrier family 31 (copper transporter), member 1	-2.0	4.2E-03
SGIP1	SH3-domain GRB2-like (endophilin) interacting protein 1	-2.0	4.2E-03
BACE2	beta-site APP-cleaving enzyme 2	-2.1	4.2E-03
ATP2A2	ATPase, Ca++ transporting, cardiac muscle, slow twitch 2	-2.1	4.2E-03
ENSGALG00000014164	Uncharacterized protein	-2.2	4.2E-03
PHYHIPL	phytanoyl-CoA hydroxylase-interacting protein-like	-2.2	4.2E-03
ZNF395	zinc finger protein 395	-2.2	4.2E-03
CREB3L2	cAMP responsive element binding protein 3-like 2	-2.3	4.2E-03
GTF2H4	general transcription factor IIH, polypeptide 4, 52kDa	-2.4	4.2E-03
FAM114A1	family with sequence similarity 114, member A1	-2.4	4.2E-03
FKBP14	FK506 binding protein 14, 22 kDa	-2.5	4.2E-03
ADRA1B	adrenoceptor alpha 1B	-2.7	4.2E-03
SPOCK1	sparc/osteonectin, cwcv and kazal-like domains proteoglycan (testican) 1	-3.3	4.2E-03
FARS2	phenylalanyl-tRNA synthetase 2, mitochondrial	-1.7	4.8E-03
ZDHHC21	zinc finger, DHHC-type containing 21	-1.8	4.8E-03
NEK7	NIMA-related kinase 7	-1.9	4.8E-03
C20orf194	chromosome 20 open reading frame 194	-1.9	4.8E-03
KIF4A	chromosome-associated kinesin KIF4	-1.9	4.8E-03
ZNF521	zinc finger protein 521	-1.9	4.8E-03

RECK	reversion-inducing-cysteine-rich protein with kazal motifs	-1.9	4.8E-03
AS3MT	arsenic (+3 oxidation state) methyltransferase	-2.0	4.8E-03
ENSGALG00000007131	Non-histone chromosomal protein HMG-14A	-2.0	4.8E-03
ENSGALG00000028322	novel gene	-2.1	4.8E-03
SCAMP5	secretory carrier membrane protein 5	-2.1	4.8E-03
GPR27	G protein-coupled receptor 27	-2.2	4.8E-03
ICK	intestinal cell (MAK-like) kinase	-2.3	4.8E-03
ARHGAP39	Rho GTPase activating protein 39	-2.4	4.8E-03
BRCA1	breast cancer 1, early onset	-2.5	4.8E-03
CENPW	centromere protein W	-2.5	4.8E-03
TGFB2	transforming growth factor, beta 2	-2.5	4.8E-03
GJA1	gap junction protein, alpha 1, 43kDa	-3.1	4.8E-03
WDR35	WD repeat domain 35	-1.9	5.4E-03
RIF1	RAP1 interacting factor homolog (yeast)	-1.9	5.4E-03
FZD10	frizzled family receptor 10	-1.9	5.4E-03
LRCH3	leucine-rich repeats and calponin homology (CH) domain containing 3	-1.9	5.4E-03
BCL11A	B-cell CLL/lymphoma 11A (zinc finger protein)	-1.9	5.4E-03
DUSP19	dual specificity phosphatase 19	-2.0	5.4E-03
MCM2	minichromosome maintenance complex component 2	-2.0	5.4E-03
NUSAP1	nucleolar and spindle associated protein 1	-2.1	5.4E-03
PHACTR1	phosphatase and actin regulator 1	-2.2	5.4E-03
ENSGALG00000023464	Uncharacterized protein	-2.3	5.4E-03
HDAC10	histone deacetylase 10	-2.3	5.4E-03
SS3R	somatostatin receptor 3	-2.4	5.4E-03
IKBIP	IKBKB interacting protein	-2.5	5.4E-03
ENSGALG00000005969	novel gene	-2.5	5.4E-03
KCTD15	potassium channel tetramerization domain containing 15	-2.6	5.4E-03
DNAH17	dynein, axonemal, heavy chain 17	-2.7	5.4E-03
SEMA3G	sema domain, immunoglobulin domain (Ig), short basic domain, secreted, (semaphorin) 3G	-3.2	5.4E-03
ENSGALG00000016326	Uncharacterized protein	-3.5	5.4E-03
C1QTNF7	C1q and tumor necrosis factor related protein 7	-3.5	5.4E-03
ACTN1	actinin, alpha 1	-1.7	6.0E-03
DNMT3B	DNA (cytosine-5-)-methyltransferase 3 beta	-1.9	6.0E-03
NPR3	natriuretic peptide receptor C/guanylate cyclase C (atrionatriuretic peptide receptor C)	-1.9	6.0E-03
H2AFY2	H2A histone family, member Y2	-1.9	6.0E-03
KAZALD1	Kazal-type serine peptidase inhibitor domain 1	-1.9	6.0E-03
KANK1	KN motif and ankyrin repeat domains 1	-2.0	6.0E-03
AMPD2	adenosine monophosphate deaminase 2	-2.3	6.0E-03
EPB41L4B	erythrocyte membrane protein band 4.1 like 4B	-2.3	6.0E-03
RAB11FIP4	RAB11 family interacting protein 4 (class II)	-2.4	6.0E-03
EP3	prostaglandin E receptor 3 (subtype *EP3*)	-2.4	6.0E-03
LGALSL	lectin, galactoside-binding-like	-2.8	6.0E-03
gga-mir-454	*gga-mir-454* [Source:miRBase;Acc:MI0006984]	/0	6.0E-03
ENSGALG00000001604	novel gene	-1.8	6.5E-03
NALCN	sodium leak channel, non-selective	-1.8	6.5E-03
KIAA1211L	KIAA1211-like	-2.0	6.5E-03
CKAP4	cytoskeleton-associated protein 4	-2.2	6.5E-03
NR3C1	glucocorticoid receptor	-2.2	6.5E-03
BUB1B	mitotic checkpoint serine/threonine-protein kinase BUB1 beta	-2.2	6.5E-03
EPB41L2	erythrocyte membrane protein band 4.1-like 2	-2.3	6.5E-03
ADRBK2,MYO18B	adrenergic, beta, receptor kinase 2	-2.4	6.5E-03
TXNRD1	thioredoxin reductase 1	-2.7	6.5E-03
THBS1	thrombospondin 1	-3.0	6.5E-03
CTCF	CCCTC-binding factor (zinc finger protein)	-1.7	7.0E-03
E2F5	E2F transcription factor 5, p130-binding	-1.8	7.0E-03
RDH10	retinol dehydrogenase 10 (all-trans)	-1.8	7.0E-03
TRIL	TLR4 interactor with leucine-rich repeats	-1.8	7.0E-03
GRHPR	glyoxylate reductase/hydroxypyruvate reductase	-1.9	7.0E-03
SGK3	serum/glucocorticoid regulated kinase family, member 3	-1.9	7.0E-03

ARHGEF3	Rho guanine nucleotide exchange factor (GEF) 3	-1.9	7.0E-03
CTSO	cathepsin O	-1.9	7.0E-03
NEDD4L	neural precursor cell expressed, developmentally down-regulated 4-like, E3 ubiquitin protein ligase	-1.9	7.0E-03
FBLN7	fibulin 7	-2.0	7.0E-03
CBX3	chromobox homolog 3	-2.0	7.0E-03
HEBP1	heme binding protein 1	-2.0	7.0E-03
NPAS3	neuronal PAS domain protein 3	-2.1	7.0E-03
TSHZ3	teashirt zinc finger homeobox 3	-2.2	7.0E-03
CASKIN1	CASK interacting protein 1	-2.2	7.0E-03
TK1	thymidine kinase 1, soluble	-2.2	7.0E-03
KLHL3	kelch-like family member 3	-2.3	7.0E-03
ENSGALG00000010722	Schwann cell-specific EGF-like repeat autocrine factor precursor	-2.3	7.0E-03
PI15	Peptidase inhibitor 15	-1.8	7.5E-03
TFDP2	transcription factor Dp-2	-1.8	7.5E-03
N6AMT2	N(6)-adenine-specific DNA methyltransferase 2	-1.8	7.5E-03
FTD	Ferritoid; Uncharacterized protein	-1.8	7.5E-03
ASNS	asparagine synthetase (glutamine-hydrolyzing)	-1.8	7.5E-03
AMY1A	amylase, alpha 1A (salivary)	-1.8	7.5E-03
FGD3	FYVE, RhoGEF and PH domain containing 3	-1.8	7.5E-03
HMGN2	high mobility group nucleosomal binding domain 2	-1.9	7.5E-03
EXOC3L1	exocyst complex component 3-like 1	-1.9	7.5E-03
CCDC47	coiled-coil domain containing 47	-1.9	7.5E-03
CDCA7	cell division cycle associated 7	-1.9	7.5E-03
TGIF1	TGFB-induced factor homeobox 1	-2.0	7.5E-03
RFTN1	raftlin, lipid raft linker 1	-2.0	7.5E-03
TCF7L2	transcription factor 7-like 2 (T-cell specific, HMG-box)	-2.0	7.5E-03
MMP17	matrix metallopeptidase 17 (membrane-inserted)	-2.2	7.5E-03
HSP90B1	heat shock protein 90kDa beta (Grp94), member 1	-2.3	7.5E-03
LURAP1	leucine rich adaptor protein 1	-2.4	7.5E-03
PGM2	phosphoglucomutase 2	-2.5	7.5E-03
PRSS12	protease, serine, 12 (neurotrypsin, motopsin)	-2.7	7.5E-03
AKAP7	A kinase (PRKA) anchor protein 7	-3.0	7.5E-03
EDG7	lysophosphatidic acid receptor 3	-4.1	7.5E-03
POFUT1	GDP-fucose protein O-fucosyltransferase 1 precursor	-1.7	8.0E-03
ENSGALG00000012412	novel gene	-1.7	8.0E-03
LCLAT1	Lysocardiolipin acyltransferase 1	-1.7	8.0E-03
PHLPP1	PH domain and leucine rich repeat protein phosphatase 1	-1.7	8.0E-03
FARP1	FERM, RhoGEF (ARHGEF) and pleckstrin domain protein 1 (chondrocyte-derived)	-1.7	8.0E-03
PRKAA1	protein kinase, AMP-activated, alpha 1 catalytic subunit	-1.8	8.0E-03
GLI3	GLI family zinc finger 3	-1.8	8.0E-03
RALGAPA1	Ral GTPase activating protein, alpha subunit 1 (catalytic)	-1.9	8.0E-03
CCNB2	G2/mitotic-specific cyclin-B2	-1.9	8.0E-03
ADSS	Adenylosuccinate synthetase isozyme 2	-1.9	8.0E-03
IGDCC4	immunoglobulin superfamily, DCC subclass, member 4	-2.0	8.0E-03
CDK2AP1	cyclin-dependent kinase 2-associated protein 1	-2.2	8.0E-03
GUCY2F	guanylate cyclase 2F, retinal	-3.3	8.0E-03
KDELC1	KDEL (Lys-Asp-Glu-Leu) containing 1	-1.6	8.5E-03
UGP2	UDP-glucose pyrophosphorylase 2	-1.7	8.5E-03
ZBTB2	zinc finger and BTB domain containing 2	-1.8	8.5E-03
OSBPL6	oxysterol binding protein-like 6	-1.9	8.5E-03
GRAMD3	GRAM domain containing 3	-1.9	8.5E-03
ENSGALG00000016141	novel gene	-1.9	8.5E-03
FAM117B	family with sequence similarity 117, member B	-2.4	8.5E-03
PPP4R1	protein phosphatase 4, regulatory subunit 1	-1.7	9.0E-03
COMMD10	COMM domain-containing protein 10	-1.7	9.0E-03
RCN2	reticulocalbin 2, EF-hand calcium binding domain	-1.8	9.0E-03
DKC1	dyskeratosis congenita 1, dyskerin	-1.8	9.0E-03
TPGS2	tubulin polyglutamylase complex subunit 2	-1.9	9.0E-03
RBBP7	retinoblastoma binding protein 7	-2.0	9.0E-03
MLH1	mutL homolog 1, colon cancer, nonpolyposis type 2 (E. coli)	-2.1	9.0E-03

DCTD	dCMP deaminase	-2.1	9.0E-03
XYLB	xylulokinase homolog (H. influenzae)	-2.4	9.0E-03
PERP	*PERP*, TP53 apoptosis effector	-2.5	9.0E-03
CAB39L	calcium binding protein 39-like	-3.8	9.0E-03
PRKCA	protein kinase C, alpha	-1.7	9.5E-03
TBC1D2B	TBC1 domain family, member 2B	-1.7	9.5E-03
FAM188B	family with sequence similarity 188, member B	-1.7	9.5E-03
TMEM68	transmembrane protein 68	-1.7	9.5E-03
MAD2L1	MAD2 mitotic arrest deficient-like 1	-1.8	9.5E-03
FAM135A	family with sequence similarity 135, member A	-1.8	9.5E-03
CAMKK2	calcium/calmodulin-dependent protein kinase kinase 2, beta	-1.8	9.5E-03
SPRY2	sprouty homolog 2 (Drosophila)	-2.0	9.5E-03
CHST3	carbohydrate (chondroitin 6) sulfotransferase 3	-2.0	9.5E-03
KCNIP2	Kv channel interacting protein 2	-2.1	9.5E-03
CAMK2D	calcium/calmodulin-dependent protein kinase II delta	-2.2	9.5E-03
PRKAB1	protein kinase, AMP-activated, beta 1 non-catalytic subunit	-1.6	1.0E-02
ZNF518B	zinc finger protein 518B	-1.7	1.0E-02
CHN1	chimerin 1	-1.7	1.0E-02
EFR3A	EFR3 homolog A (S. cerevisiae)	-1.8	1.0E-02
LMNA	lamin A/C	-1.8	1.0E-02
PPP2R3A	protein phosphatase 2, regulatory subunit B,,, alpha	-1.8	1.0E-02
XBP1	X-box binding protein 1	-1.9	1.0E-02
OSBPL3	oxysterol binding protein-like 3	-2.0	1.0E-02
C5ORF13	neuronal protein 3.1	-2.6	1.0E-02
SEPP1	selenoprotein P, plasma, 1	-2.9	1.0E-02
AGXT2L1	alanine-glyoxylate aminotransferase 2-like 1	-2.9	1.0E-02
RNaseP_nuc	Nuclear RNase P	-4.8	1.0E-02
ZMYND11	zinc finger, MYND-type containing 11	-1.7	1.0E-02
SACS	spastic ataxia of Charlevoix-Saguenay (sacsin)	-1.8	1.0E-02
CPNE3	copine III	-1.9	1.0E-02
USP12	ubiquitin specific peptidase 12	-2.0	1.0E-02
SNED1	sushi, nidogen and EGF-like domains 1	-2.6	1.0E-02
STEAP2	STEAP family member 2, metalloreductase	-3.1	1.0E-02
PCDH1	protocadherin 1	-1.6	1.1E-02
ENSGALG00000002852	novel gene	-1.7	1.1E-02
GPR161,TTF2	transcription termination factor, RNA polymerase II	-1.7	1.1E-02
FOXM1	forkhead box M1	-1.8	1.1E-02
SIPA1L2	signal-induced proliferation-associated 1 like 2	-1.8	1.1E-02
IL11RA	interleukin 11 receptor, alpha	-1.8	1.1E-02
DAP	death-associated protein	-1.8	1.1E-02
POMT1	protein-O-mannosyltransferase 1	-1.8	1.1E-02
PLK1	polo-like kinase 1	-2.0	1.1E-02
POLA1	polymerase (DNA directed), alpha 1, catalytic subunit	-2.0	1.1E-02
CKI	beta-1,4-galactosyltransferase 1	-2.1	1.1E-02
GAS2	growth arrest-specific 2	-2.3	1.1E-02
U1	*U1* spliceosomal RNA	/0	1.1E-02
NEDD1	neural precursor cell expressed, developmentally down-regulated 1	-1.6	1.1E-02
B3GALTL	beta 1,3-galactosyltransferase-like	-1.8	1.1E-02
COL4A2	collagen, type IV, alpha 2	-1.9	1.1E-02
DYNC2LI1	dynein, cytoplasmic 2, light intermediate chain 1	-1.9	1.1E-02
DERL3	derlin 3	-1.9	1.1E-02
SNTA1	syntrophin, alpha 1	-2.0	1.1E-02
TAGLN	transgelin	-2.4	1.1E-02
gga-mir-30d	*gga-mir-30d* [Source:miRBase;Acc:MI0001198]	/0	1.1E-02
NCAPD2	non-SMC condensin I complex, subunit D2	-1.7	1.2E-02
ENSGALG00000027754	novel gene	-1.8	1.2E-02
CAMK1D	calcium/calmodulin-dependent protein kinase ID	-1.8	1.2E-02
LRIG3	leucine-rich repeats and immunoglobulin-like domains 3	-1.8	1.2E-02
PTGFRN	prostaglandin F2 receptor inhibitor	-1.8	1.2E-02
IKBIP	IKBKB interacting protein	-1.9	1.2E-02
GSTA	Glutathione S-transferase	-1.9	1.2E-02
SMAD1	SMAD family member 1	-1.9	1.2E-02

HTR7	5-hydroxytryptamine (serotonin) receptor 7, adenylate cyclase-coupled	-1.9	1.2E-02
VANGL2	VANGL planar cell polarity protein 2	-2.0	1.2E-02
NCAPG	non-SMC condensin I complex, subunit G	-2.0	1.2E-02
ENSGALG00000008755	novel gene	-2.3	1.2E-02
S1PR3	sphingosine-1-phosphate receptor 3	-2.5	1.2E-02
MKL2	MKL/myocardin-like 2	-1.7	1.3E-02
POLD3	polymerase (DNA-directed), delta 3, accessory subunit	-1.7	1.3E-02
IRF2BPL	interferon regulatory factor 2 binding protein-like	-1.8	1.3E-02
SRSF3	serine/arginine-rich splicing factor 3	-1.8	1.3E-02
BCL9L	B-cell CLL/lymphoma 9-like	-1.8	1.3E-02
ENSGALG00000015653	junctophilin 1	-1.8	1.3E-02
EPB41L3	erythrocyte membrane protein band 4.1-like 3	-2.0	1.3E-02
SEMA5B	sema domain, seven thrombospondin repeats (type 1 and type 1-like), transmembrane domain (TM) and short cytoplasmic domain, (semaphorin) 5B	-2.1	1.3E-02
PIGQ	phosphatidylinositol glycan anchor biosynthesis, class Q	-2.3	1.3E-02
PLXNA4	plexin A4	-4.6	1.3E-02
FUCA1	fucosidase, alpha-L- 1, tissue	-1.6	1.3E-02
ARPC5	actin related protein 2/3 complex, subunit 5, 16kDa	-1.7	1.3E-02
USP54	ubiquitin specific peptidase 54	-1.7	1.3E-02
CCNE1	cyclin E1	-1.8	1.3E-02
ACSF2	acyl-CoA synthetase family member 2	-1.9	1.3E-02
MCM3	minichromosome maintenance complex component 3	-1.9	1.3E-02
PPFIBP1	PTPRF interacting protein, binding protein 1 (liprin beta 1)	-1.9	1.3E-02
ANKS6	ankyrin repeat and sterile alpha motif domain containing 6	-2.5	1.3E-02
ARG2	arginase 2	-2.7	1.3E-02
MATN2	matrilin 2	-4.3	1.3E-02
CORTBP2	cortactin binding protein 2	-1.6	1.3E-02
ENSGALG00000008567	novel gene	-1.6	1.3E-02
C3ORF55	Uncharacterized protein	-1.7	1.3E-02
CCDC102A	coiled-coil domain containing 102A	-1.7	1.3E-02
GALNTL4	UDP-N-acetyl-alpha-D-galactosamine:polypeptide N-acetylgalactosaminyltransferase 18	-1.7	1.3E-02
ZMYND8	zinc finger, MYND-type containing 8	-1.8	1.3E-02
ADARB1	adenosine deaminase, RNA-specific, B1	-1.9	1.3E-02
C14ORF135	Uncharacterized protein	-1.9	1.3E-02
FBLN1	fibulin 1	-1.9	1.3E-02
ELOVL7	ELOVL fatty acid elongase 7	-2.1	1.3E-02
SPOCK3	sparc/osteonectin, cwcv and kazal-like domains proteoglycan (testican) 3	-2.2	1.3E-02
USP3	ubiquitin specific peptidase 3	-1.7	1.4E-02
PROM1	prominin 1	-1.9	1.4E-02
CCNB3	cyclin B3	-1.9	1.4E-02
TRPC6	transient receptor potential cation channel, subfamily C, member 6	-1.9	1.4E-02
WNT5A	wingless-type MMTV integration site family, member 5A	-2.0	1.4E-02
IMPA2	inositol(myo)-1(or 4)-monophosphatase 2	-2.3	1.4E-02
SHISA5	shisa homolog 5 (Xenopus laevis)	-1.7	1.4E-02
PQLC1	PQ loop repeat containing 1	-1.8	1.4E-02
P4HA2	prolyl 4-hydroxylase, alpha polypeptide II	-1.8	1.4E-02
EHHADH	enoyl-CoA, hydratase/3-hydroxyacyl CoA dehydrogenase	-1.8	1.4E-02
ENSGALG00000004132	novel gene	-1.9	1.4E-02
FBP1	fructose-1,6-bisphosphatase 1	-2.7	1.4E-02
ER81	ETS translocation variant 1	-1.7	1.5E-02
SLC13A3	solute carrier family 13 (sodium-dependent dicarboxylate transporter), member 3	-1.7	1.5E-02
CHAF1B	chromatin assembly factor 1, subunit B (p60)	-1.8	1.5E-02
NPM1	nucleophosmin (nucleolar phosphoprotein B23, numatrin)	-2.4	1.5E-02
TRPM1	transient receptor potential cation channel, subfamily M, member 1	-2.8	1.5E-02
SCRT2	scratch homolog 2, zinc finger protein (Drosophila)	-3.3	1.5E-02
LRRC16A	leucine rich repeat containing 16A	-1.6	1.5E-02

PTPRD	protein tyrosine phosphatase, receptor type, D	-1.6	1.5E-02
C28H19ORF10	UPF0556 protein C19orf10 homolog precursor	-1.7	1.5E-02
D2HGDH	D-2-hydroxyglutarate dehydrogenase	-1.7	1.5E-02
RREB1	ras responsive element binding protein 1	-1.7	1.5E-02
ENSGALG00000015577	novel gene	-1.9	1.5E-02
SDCBP	syndecan binding protein (syntenin)	-2.5	1.5E-02
MTHFR	methylenetetrahydrofolate reductase (NAD(P)H)	-1.6	1.6E-02
HSPA5	heat shock 70kDa protein 5 (glucose-regulated protein, 78kDa)	-1.9	1.6E-02
ENSGALG00000014513	novel gene	-2.0	1.6E-02
AMER2	APC membrane recruitment protein 2	-2.1	1.6E-02
TNFRSF1A	tumor necrosis factor receptor superfamily, member 1A	-1.6	1.6E-02
DNAL1	dynein, axonemal, light chain 1	-1.6	1.6E-02
RWDD3	RWD domain containing 3	-1.6	1.6E-02
MPP5	membrane protein, palmitoylated 5 (MAGUK p55 subfamily member 5)	-1.6	1.6E-02
CRELD2	cysteine-rich with EGF-like domains 2	-1.7	1.6E-02
ABHD6	abhydrolase domain containing 6	-1.8	1.6E-02
SUN2	Sad1 and UNC84 domain containing 2	-1.9	1.6E-02
ZDHHC12	zinc finger, DHHC-type containing 12	-2.1	1.6E-02
KIAA1147	*KIAA1147*	-2.1	1.6E-02
CASP6	caspase 6, apoptosis-related cysteine peptidase	-2.4	1.6E-02
CHEK1	checkpoint kinase 1	-2.5	1.6E-02
EML4	echinoderm microtubule associated protein like 4	-2.6	1.6E-02
FABP3	fatty acid binding protein 3, muscle and heart (mammary-derived growth inhibitor)	-4.1	1.6E-02
CD2AP	CD2-associated protein	-1.6	1.6E-02
TDP1	tyrosyl-DNA phosphodiesterase 1	-1.8	1.6E-02
RNFT1	ring finger protein, transmembrane 1	-1.8	1.6E-02
RCOR1	REST corepressor 1	-1.9	1.6E-02
ZNF488	zinc finger protein 488	-2.4	1.6E-02
ASB13	ankyrin repeat and SOCS box containing 13	-2.6	1.6E-02
HIF1A	hypoxia inducible factor 1, alpha subunit (basic helix-loop-helix transcription factor)	-2.6	1.6E-02
RAD51A	RAD51 recombinase	-2.7	1.6E-02
ENSGALG00000026521	novel gene	-3.0	1.6E-02
BRI3BP	BRI3 binding protein	-1.7	1.7E-02
FAM122A	family with sequence similarity 122A	-1.7	1.7E-02
SNAPC3	small nuclear RNA activating complex, polypeptide 3, 50kDa	-1.9	1.7E-02
N4BP2	NEDD4 binding protein 2	-1.6	1.7E-02
ASPM	asp (abnormal spindle) homolog, microcephaly associated (Drosophila)	-1.6	1.7E-02
ARSK	arylsulfatase family, member K	-1.6	1.7E-02
BAZ1A	bromodomain adjacent to zinc finger domain, 1A	-1.7	1.7E-02
SLC16A5	solute carrier family 16, member 5 (monocarboxylic acid transporter 6)	-1.7	1.7E-02
SLC6A15	solute carrier family 6 (neutral amino acid transporter), member 15	-1.8	1.7E-02
ENSGALG00000009432	Uncharacterized protein	-2.1	1.7E-02
UGT8	UDP glycosyltransferase 8	-2.5	1.7E-02
ABCG1	ATP-binding cassette, sub-family G (WHITE), member 1	-2.8	1.7E-02
ENSGALG00000009432	Uncharacterized protein	-1.6	1.7E-02
SPRED2	sprouty-related, EVH1 domain containing 2	-1.8	1.7E-02
DFNA5	deafness, autosomal dominant 5	-1.9	1.7E-02
SAE2	ubiquitin-like modifier activating enzyme 2	-1.5	1.8E-02
C3H6ORF72	uncharacterized protein C6orf72 homolog precursor	-1.6	1.8E-02
BAMBI	BMP and activin membrane-bound inhibitor homolog (Xenopus laevis)	-1.6	1.8E-02
EML1	echinoderm microtubule associated protein like 1	-1.6	1.8E-02
FAM98B	family with sequence similarity 98, member B	-1.7	1.8E-02
NFYB	nuclear transcription factor Y, beta	-1.7	1.8E-02
FRS2	fibroblast growth factor receptor substrate 2	-1.8	1.8E-02
PTPRF	protein tyrosine phosphatase, receptor type, F	-2.1	1.8E-02

BTF3	basic transcription factor 3	-1.6	1.8E-02
TOP2A	topoisomerase (DNA) II alpha 170kDa	-1.6	1.8E-02
FAM108C1	Abhydrolase domain-containing protein *FAM108C1*	-1.6	1.8E-02
IFT81	intraflagellar transport 81 homolog (Chlamydomonas)	-1.9	1.8E-02
TNPO1	transportin 1	-1.5	1.9E-02
PXMP3	peroxin 2	-1.6	1.9E-02
VAV2	*vav 2* guanine nucleotide exchange factor	-1.6	1.9E-02
MEIS1	Meis homeobox 1	-1.7	1.9E-02
GMPR	guanosine monophosphate reductase	-2.3	1.9E-02
SOWAHC	sosondowah ankyrin repeat domain family member C	-1.5	1.9E-02
C3ORF23	Uncharacterized protein	-1.5	1.9E-02
PDIA5	protein disulfide isomerase family A, member 5	-1.6	1.9E-02
RYBP	RING1 and YY1 binding protein	-1.7	1.9E-02
RCN1	reticulocalbin 1, EF-hand calcium binding domain	-1.8	1.9E-02
CMAS	cytidine monophosphate N-acetylneuraminic acid synthetase	-1.9	1.9E-02
IRF6	interferon regulatory factor 6	-2.1	1.9E-02
HSP90AB1	heat shock protein 90kDa alpha (cytosolic), class B member 1	-2.2	1.9E-02
CCNA1	cyclin A1	-2.2	1.9E-02
TSSC4	tumor suppressing subtransferable candidate 4	-1.5	1.9E-02
PARD6G	par-6 partitioning defective 6 homolog gamma (C. elegans)	-1.7	1.9E-02
PLCH1	phospholipase C, eta 1	-1.8	1.9E-02
EIF2C1	Uncharacterized protein	-1.9	1.9E-02
RXRA	retinoid X receptor, alpha	-1.6	2.0E-02
MT4	metallothionein 4	-1.6	2.0E-02
ALDH4A1	aldehyde dehydrogenase 4 family, member A1	-1.7	2.0E-02
GNE	glucosamine (UDP-N-acetyl)-2-epimerase/N-acetylmannosamine kinase	-1.7	2.0E-02
CAPN5	calpain 5	-1.9	2.0E-02
UGGT2	UDP-glucose glycoprotein glucosyltransferase 2	-1.5	2.0E-02
HACE1	HECT domain and ankyrin repeat containing E3 ubiquitin protein ligase 1	-1.5	2.0E-02
DCPS	decapping enzyme, scavenger	-1.6	2.0E-02
SLC25A12	solute carrier family 25 (aspartate/glutamate carrier), member 12	-1.6	2.0E-02
MCM5	minichromosome maintenance complex component 5	-1.9	2.0E-02
RALGPS1	Ral GEF with PH domain and SH3 binding motif 1	-2.0	2.0E-02
SNORD95	small nucleolar RNA, C/D box 95	/0	2.0E-02
LEPREL1	leprecan-like 1	-1.5	2.0E-02
POLE3	polymerase (DNA directed), epsilon 3, accessory subunit	-1.6	2.0E-02
ENSGALG00000012158	novel gene	-1.7	2.0E-02
C12orf5	chromosome 12 open reading frame 5	-1.7	2.0E-02
RNF130	ring finger protein 130	-1.9	2.0E-02
FSCN1	fascin homolog 1, actin-bundling protein (Strongylocentrotus purpuratus)	-2.0	2.0E-02
HUS1	*HUS1* checkpoint homolog (S. pombe)	-2.4	2.0E-02
DBT	dihydrolipoamide branched chain transacylase E2	-1.5	2.1E-02
TMEM164	transmembrane protein 164	-1.6	2.1E-02
C6orf70	chromosome 6 open reading frame 70	-1.6	2.1E-02
SMAD3	SMAD family member 3	-1.7	2.1E-02
DMD	dystrophin	-1.8	2.1E-02
LIMD2	LIM domain containing 2	-2.0	2.1E-02
ENSGALG00000006407	death domain-containing tumor necrosis factor receptor superfamily member 23 precursor	-2.9	2.1E-02
PTTG1IP	pituitary tumor-transforming 1 interacting protein	-1.6	2.1E-02
RNASEL	ribonuclease L (2,,5,-oligoisoadenylate synthetase-dependent)	-2.3	2.1E-02
TRIM9	tripartite motif containing 9	-1.6	2.2E-02
RACGAP1	Rac GTPase activating protein 1	-1.9	2.2E-02
THADA	thyroid adenoma associated	-1.5	2.2E-02
C9ORF4	Uncharacterized protein	-1.5	2.2E-02
GPR155	G protein-coupled receptor 155	-1.5	2.2E-02

ENSGALG00000014190	novel gene	-1.5	2.2E-02
SLC25A13	solute carrier family 25 (aspartate/glutamate carrier), member 13	-1.6	2.2E-02
ENSGALG00000013233	novel gene	-1.7	2.2E-02
SLC1A2	solute carrier family 1 (glial high affinity glutamate transporter), member 2	-1.8	2.2E-02
AP1S2	adaptor-related protein complex 1, sigma 2 subunit	-1.9	2.2E-02
NRP2	neuropilin 2	-2.0	2.2E-02
C13ORF7	Uncharacterized protein	-1.5	2.2E-02
BUB3	*BUB3* mitotic checkpoint protein	-1.5	2.2E-02
ARHGDIB	Rho GDP dissociation inhibitor (GDI) beta	-1.5	2.2E-02
SULT1E1	sulfotransferase family 1E, estrogen-preferring, member 1	-1.6	2.2E-02
CDK2	cyclin-dependent kinase 2	-2.3	2.2E-02
SMAD6	SMAD family member 6	-1.7	2.3E-02
CASK	calcium/calmodulin-dependent serine protein kinase (MAGUK family)	-1.9	2.3E-02
EFNB2	ephrin-B2	-2.0	2.3E-02
CXORF41	novel gene	-2.3	2.3E-02
NRARP	NOTCH-regulated ankyrin repeat protein	-1.6	2.3E-02
LBH	limb bud and heart development	-1.8	2.3E-02
RASL11A	RAS-like, family 11, member A	-2.9	2.3E-02
SMARCD3	SWI/SNF related, matrix associated, actin dependent regulator of chromatin, subfamily d, member 3	-1.6	2.3E-02
ACER3	alkaline ceramidase 3	-1.6	2.3E-02
SLC40A1	solute carrier family 40 (iron-regulated transporter), member 1	-1.6	2.3E-02
USP6NL	USP6 N-terminal like	-1.6	2.3E-02
SUSD1	sushi domain containing 1	-2.3	2.3E-02
KCNIP4	Kv channel interacting protein 4	-3.3	2.3E-02
SLC35C2	solute carrier family 35 (GDP-fucose transporter), member C2	-1.5	2.4E-02
PPDPF	pancreatic progenitor cell differentiation and proliferation factor homolog (zebrafish)	-1.6	2.4E-02
RHOBTB1	Rho-related BTB domain containing 1	-1.8	2.4E-02
ENSGALG00000002475,ENSGALG00000027637	Uncharacterized protein	-1.7	2.4E-02
B3GNT2	UDP-GlcNAc:betaGal beta-1,3-N-acetylglucosaminyltransferase 2	-2.1	2.4E-02
AKAP11	A kinase (PRKA) anchor protein 11	-1.5	2.4E-02
WTAP	Wilms tumor 1 associated protein	-1.5	2.4E-02
SWAP70	SWAP switching B-cell complex 70kDa subunit	-1.6	2.4E-02
FOXP2	forkhead box P2	-1.6	2.4E-02
NTN1	netrin 1	-1.8	2.4E-02
FAM19A3	family with sequence similarity 19 (chemokine (C-C motif)-like), member A3	-2.2	2.4E-02
HMG20A	high mobility group 20A	-1.5	2.5E-02
PPWD1	peptidylprolyl isomerase domain and WD repeat containing 1	-1.5	2.5E-02
CEP95	centrosomal protein 95kDa	-1.6	2.5E-02
SEMA6A	sema domain, transmembrane domain (TM), and cytoplasmic domain, (semaphorin) 6A	-1.6	2.5E-02
PPIB	peptidylprolyl isomerase B (cyclophilin B)	-1.7	2.5E-02
FLT4	fms-related tyrosine kinase 4	-1.7	2.5E-02
H2AFV	H2A histone family, member V	-1.4	2.5E-02
MAPKBP1	mitogen-activated protein kinase binding protein 1	-1.5	2.5E-02
SLC6A9	solute carrier family 6 (neurotransmitter transporter, glycine), member 9	-1.5	2.5E-02
SULT1B1	sulfotransferase family, cytosolic, 1B, member 1	-1.5	2.5E-02
INTS4	integrator complex subunit 4	-1.6	2.5E-02
NXPE3	neurexophilin and PC-esterase domain family, member 3	-2.0	2.5E-02
CCND1	cyclin D1	-2.3	2.5E-02
SNORD16	small nucleolar RNA, C/D box 16	/0	2.5E-02
NMNAT1	nicotinamide nucleotide adenylyltransferase 1	-1.5	2.5E-02

ZC3H12B	zinc finger CCCH-type containing 12B	-1.6	2.5E-02
TRIM13	tripartite motif containing 13	-1.6	2.5E-02
CHST11	carbohydrate (chondroitin 4) sulfotransferase 11	-1.6	2.5E-02
PPIP5K2	diphosphoinositol pentakisphosphate kinase 2	-1.6	2.5E-02
FAM110C	family with sequence similarity 110, member C	-2.2	2.5E-02
FAM54A	Uncharacterized protein	-2.3	2.5E-02
RPS6KA3	ribosomal protein S6 kinase, 90kDa, polypeptide 3	-1.4	2.6E-02
SGCD	sarcoglycan, delta (35kDa dystrophin-associated glycoprotein)	-2.5	2.6E-02
ITSN1	intersectin 1 (SH3 domain protein)	-1.6	2.6E-02
NUF2	*NUF2*, NDC80 kinetochore complex component	-1.9	2.6E-02
SDC1	syndecan 1	-2.0	2.6E-02
LMCD1	LIM and cysteine-rich domains 1	-2.0	2.6E-02
DIXDC1	DIX domain containing 1	-2.0	2.6E-02
LNX1	ligand of numb-protein X 1, E3 ubiquitin protein ligase	-3.3	2.6E-02
C18ORF22	ribosome binding factor A (putative)	-1.6	2.7E-02
LCAT	lecithin-cholesterol acyltransferase	-1.8	2.7E-02
AKR	aldo-keto reductase family 1 member B10	-2.0	2.7E-02
ENSGALG00000011233	novel gene	-3.2	2.7E-02
ZWILCH	*zwilch* kinetochore protein	-1.4	2.7E-02
FZD7	frizzled family receptor 7	-1.5	2.7E-02
SATB1	SATB homeobox 1	-1.6	2.7E-02
WBSCR17	Williams-Beuren syndrome chromosome region 17	-1.6	2.7E-02
SLC17A5	solute carrier family 17 (anion/sugar transporter), member 5	-1.6	2.7E-02
ITGB8	integrin, beta 8	-1.9	2.7E-02
ECT2	epithelial cell transforming sequence 2 oncogene	-2.1	2.7E-02
FAM83D	family with sequence similarity 83, member	-2.7	2.7E-02
SLC38A10	solute carrier family 38, member 10	-1.5	2.7E-02
KIAA1009	*KIAA1009*	-1.5	2.7E-02
MST1	macrophage stimulating 1 (hepatocyte growth factor-like)	-1.6	2.7E-02
RCOR3	REST corepressor 3	-1.5	2.7E-02
SUB1	*SUB1* homolog (S. cerevisiae)	-1.5	2.7E-02
SMARCD1	SWI/SNF related, matrix associated, actin dependent regulator of chromatin, subfamily d, member 1	-1.6	2.7E-02
ALG12	*ALG12*, alpha-1,6-mannosyltransferase	-1.6	2.7E-02
DOC2B	double C2-like domains, beta	-2.3	2.7E-02
ISCU	iron-sulfur cluster scaffold homolog (E. coli)	-1.4	2.8E-02
RNPEP	arginyl aminopeptidase (aminopeptidase B)	-1.5	2.8E-02
SLC30A7	solute carrier family 30 (zinc transporter), member 7	-1.8	2.8E-02
ANXA5	annexin A5	-1.5	2.8E-02
PPIF	peptidylprolyl isomerase F	-1.7	2.8E-02
FLRT2	fibronectin leucine rich transmembrane protein 2	-1.7	2.8E-02
BMP2K	BMP2 inducible kinase	-1.8	2.8E-02
NID1	nidogen 1	-2.3	2.8E-02
SLC9A3R2	solute carrier family 9, subfamily A (NHE3, cation proton antiporter 3), member 3 regulator 2	-1.5	2.8E-02
LSM7	*LSM7* homolog, U6 small nuclear RNA associated (S. cerevisiae)	-1.5	2.8E-02
FAM208A	family with sequence similarity 208, member A	-1.5	2.8E-02
KIAA1333	G2/M-phase specific E3 ubiquitin protein ligase	-1.7	2.8E-02
DHX33	DEAH (Asp-Glu-Ala-His) box polypeptide 33	-1.4	2.9E-02
GAS41	YEATS domain-containing protein 4	-1.4	2.9E-02
FZD2	frizzled family receptor 2	-1.6	2.9E-02
NAV2	neuron navigator 2	-1.7	2.9E-02
NCAPD3	non-SMC condensin II complex, subunit D3	-2.3	2.9E-02
GABPB2	GA binding protein transcription factor, beta subunit 2	-1.5	2.9E-02
PARN	poly(A)-specific ribonuclease	-1.5	2.9E-02
WWP1	WW domain containing E3 ubiquitin protein ligase 1	-1.5	2.9E-02
PODXL2	podocalyxin-like 2	-1.6	2.9E-02
STK38L	serine/threonine kinase 38 like	-1.7	2.9E-02
BBS7	Bardet-Biedl syndrome 7	-1.7	2.9E-02
TXNDC12	thioredoxin domain containing 12 (endoplasmic reticulum)	-1.4	2.9E-02
CHKA	choline kinase alpha	-1.5	2.9E-02

TUSC3	tumor suppressor candidate 3	-1.5	2.9E-02
DOCK10	dedicator of cytokinesis 10	-1.5	2.9E-02
HNRPLL	heterogeneous nuclear ribonucleoprotein L-like	-1.6	2.9E-02
PXDNL	peroxidasin homolog (Drosophila)-like	-1.6	2.9E-02
STARD5	StAR-related lipid transfer (START) domain containing 5	-1.6	2.9E-02
SNAPC5	small nuclear RNA activating complex, polypeptide 5, 19kDa	-1.4	3.0E-02
SPINZ	Spindlin-Z	-1.6	3.0E-02
ID1	inhibitor of DNA binding 1, dominant negative helix-loop-helix protein	-1.6	3.0E-02
RAD18	*RAD18* homolog (S. cerevisiae)	-1.8	3.0E-02
PPYR1	neuropeptide Y receptor type 4	-2.4	3.0E-02
SMARCA5	SWI/SNF related, matrix associated, actin dependent regulator of chromatin, subfamily a, member 5	-1.4	3.0E-02
MBTPS1	membrane-bound transcription factor peptidase, site 1	-1.4	3.0E-02
HIP1	huntingtin interacting protein 1	-1.5	3.0E-02
FAM69B	family with sequence similarity 69, member B	-1.6	3.0E-02
KLHL9	kelch-like family member 9	-1.4	3.1E-02
GGH	gamma-glutamyl hydrolase (conjugase, folylpolygammaglutamyl hydrolase)	-1.5	3.1E-02
MAEA	macrophage erythroblast attacher	-1.7	3.1E-02
TSC22D1	TSC22 domain family, member 1	-1.9	3.1E-02
GNB1	guanine nucleotide binding protein (G protein), beta polypeptide 1	-1.9	3.1E-02
PBLD	phenazine biosynthesis-like protein domain containing	-2.5	3.1E-02
VSIG10	V-set and immunoglobulin domain containing 10	-1.5	3.1E-02
STRADB	STE20-related kinase adaptor beta	-1.7	3.1E-02
ENSGALG00000007030	novel gene	-2.3	3.1E-02
KIRREL	kin of IRRE like (Drosophila)	-1.5	3.1E-02
WNT2B	wingless-type MMTV integration site family, member 2B	-1.9	3.1E-02
CZH5ORF42	Uncharacterized protein	-1.4	3.2E-02
EPHB2	EPH receptor B2	-1.5	3.2E-02
ANLN	anillin, actin binding protein	-1.6	3.2E-02
RFX3	regulatory factor X, 3 (influences HLA class II expression)	-1.8	3.2E-02
PANX1	pannexin 1	-1.9	3.2E-02
EIF3B	eukaryotic translation initiation factor 3, subunit B	-2.0	3.2E-02
HECW2	HECT, C2 and WW domain containing E3 ubiquitin protein ligase 2	-1.5	3.2E-02
ABCB11	ATP-binding cassette, sub-family B (MDR/TAP), member 11	-1.5	3.2E-02
CLCN4	chloride channel, voltage-sensitive 4	-1.7	3.2E-02
ANP32B	acidic (leucine-rich) nuclear phosphoprotein 32 family, member B	-1.7	3.2E-02
ENTPD2	ectonucleoside triphosphate diphosphohydrolase 2	-2.2	3.2E-02
GGA.4354	Uncharacterized protein	-2.3	3.2E-02
ENSGALG00000008518	Glutamine synthetase	-2.8	3.2E-02
MYC	v-*myc* avian myelocytomatosis viral oncogene homolog	-1.6	3.2E-02
PRRC1	proline-rich coiled-coil 1	-1.4	3.3E-02
PCDHA11	protocadherin alpha 11	-1.4	3.3E-02
OSBPL8	oxysterol binding protein-like 8	-1.5	3.3E-02
FRZB	frizzled-related protein	-1.6	3.3E-02
CTGF	connective tissue growth factor	-1.7	3.3E-02
MIPOL1	mirror-image polydactyly 1	-2.2	3.3E-02
IQGAP1	IQ motif containing GTPase activating protein 1	-1.7	3.3E-02
FBXO5	F-box protein 5	-1.7	3.3E-02
SPPL2A	signal peptide peptidase like 2A	-1.7	3.3E-02
CCDC3	coiled-coil domain containing 3	-1.7	3.3E-02
ENSGALG00000006724	novel gene	-2.3	3.3E-02
TSPAN12	tetraspanin 12	-1.5	3.3E-02
FAM171B	family with sequence similarity 171, member B	-1.5	3.3E-02
MTAP	methylthioadenosine phosphorylase	-1.7	3.3E-02
GEM	GTP binding protein overexpressed in skeletal muscle	-1.7	3.3E-02
PRG4	proteoglycan 4	-1.5	3.4E-02
ITFG2	integrin alpha FG-GAP repeat containing 2	-1.5	3.4E-02
HDDC2	HD domain containing 2	-1.5	3.4E-02

GMFB	glia maturation factor, beta	-1.7	3.4E-02
HSF3	Heat shock factor protein 3	-1.4	3.4E-02
SEC22A	SEC22 vesicle trafficking protein homolog A (S. cerevisiae)	-1.4	3.4E-02
CDCA4	cell division cycle associated 4	-1.4	3.4E-02
GPATCH2	G patch domain containing 2	-1.4	3.4E-02
AGL	amylo-alpha-1, 6-glucosidase, 4-alpha-glucanotransferase	-1.5	3.4E-02
SRD5A3	steroid 5 alpha-reductase 3	-1.5	3.4E-02
LRP8	low density lipoprotein receptor-related protein 8, apolipoprotein e receptor	-1.7	3.4E-02
PSPH	phosphoserine phosphatase	-1.9	3.4E-02
ARSH	arylsulfatase family, member H	-2.2	3.4E-02
ABCC10	ATP-binding cassette, sub-family C (CFTR/MRP), member 10	-1.4	3.4E-02
TOX	thymocyte selection-associated high mobility group box	-1.5	3.4E-02
CTSL2	cathepsin L1 precursor	-1.8	3.4E-02
NENF	neudesin neurotrophic factor	-1.4	3.4E-02
EPC2	enhancer of polycomb homolog 2 (Drosophila)	-1.4	3.4E-02
SCPEP1	serine carboxypeptidase 1	-1.4	3.4E-02
CHPKCI	histidine triad nucleotide-binding protein 1	-1.4	3.4E-02
RICTOR	RPTOR independent companion of MTOR, complex 2	-1.5	3.4E-02
FHOD3	formin homology 2 domain containing 3	-1.5	3.4E-02
POLG2	polymerase (DNA directed), gamma 2, accessory subunit	-1.5	3.4E-02
PAX-7	paired box protein *Pax-7*	-1.8	3.4E-02
C4ORF33	chromosome 4 open reading frame 33	-2.2	3.4E-02
SUPV3L1	suppressor of var1, 3-like 1 (S. cerevisiae)	-1.4	3.5E-02
ERH	enhancer of rudimentary homolog (Drosophila)	-1.4	3.5E-02
LMNB2	lamin B2	-1.5	3.5E-02
ECE-1	endothelin converting enzyme *1*	-1.4	3.5E-02
SEMA4G	sema domain, immunoglobulin domain (Ig), transmembrane domain (TM) and short cytoplasmic domain, (semaphorin) 4G	-1.4	3.5E-02
BFAR	bifunctional apoptosis regulator	-1.5	3.5E-02
IFT46	intraflagellar transport 46 homolog (Chlamydomonas)	-1.5	3.5E-02
CENPH	centromere protein H	-1.9	3.5E-02
INCENP	inner centromere protein antigens 135/155kDa	-2.2	3.5E-02
RAD21	*RAD21* homolog (S. pombe)	-1.5	3.6E-02
SLC7A6	solute carrier family 7 (amino acid transporter light chain, y+L system), member 6	-1.5	3.6E-02
KCTD18	potassium channel tetramerization domain containing 18	-1.8	3.6E-02
LEPRE1	leucine proline-enriched proteoglycan (leprecan) 1	-1.4	3.6E-02
GGA.42833	Uncharacterized protein	-1.4	3.6E-02
FDX1	ferredoxin 1	-1.5	3.6E-02
MGP	matrix Gla protein	-1.8	3.6E-02
DSCC1	DNA replication and sister chromatid cohesion 1	-2.6	3.6E-02
IRS1	insulin receptor substrate 1	-1.7	3.6E-02
KCNMA1	potassium large conductance calcium-activated channel, subfamily M, alpha member 1	-1.8	3.6E-02
SLC44A5	solute carrier family 44, member 5	-1.4	3.7E-02
ZNF827	zinc finger protein 827	-1.5	3.7E-02
ZNRF2	zinc and ring finger 2	-2.5	3.7E-02
ENSGALG00000017332	novel gene	-3.2	3.7E-02
KIF7	kinesin family member 7	-1.4	3.7E-02
ENSGALG00000028932	novel gene	-1.8	3.7E-02
NKTR	natural killer-tumor recognition sequence	-1.5	3.7E-02
UBP1	upstream binding protein 1 (LBP-1a)	-1.4	3.8E-02
SNORD14	Small nucleolar RNA *SNORD14*	/0	3.8E-02
ENSGALG00000004169	Uncharacterized protein	-1.4	3.8E-02
NUDT14	nudix (nucleoside diphosphate linked moiety X)-type motif 14	-1.4	3.8E-02
C4BPA	complement component 4 binding protein, alpha	-1.4	3.8E-02
TMEM38B	transmembrane protein 38B	-1.4	3.8E-02
TMEM132C	transmembrane protein 132C	-1.6	3.8E-02
MAK	male germ cell-associated kinase	-1.4	3.8E-02
ANKRD32	ankyrin repeat domain 32	-1.5	3.8E-02

CCNY	cyclin Y	-1.4	3.9E-02
ENSGALG00000029059	novel gene	-1.7	3.9E-02
NUP205	nucleoporin 205kDa	-1.5	3.9E-02
MKS1	Meckel syndrome, type 1	-1.5	3.9E-02
RRP8	ribosomal RNA processing 8, methyltransferase, homolog (yeast)	-2.5	3.9E-02
MSI2	musashi RNA-binding protein 2	-1.4	3.9E-02
RABL5	RAB, member RAS oncogene family-like 5	-1.5	3.9E-02
ZBTB47	zinc finger and BTB domain containing 47	-1.5	3.9E-02
ARNTL2	aryl hydrocarbon receptor nuclear translocator-like 2	-1.7	4.0E-02
WDFY1	WD repeat and FYVE domain containing 1	-1.4	4.0E-02
GALNT2	UDP-N-acetyl-alpha-D-galactosamine:polypeptide N-acetylgalactosaminyltransferase 2 (GalNAc-T2)	-1.5	4.0E-02
SYNE2	spectrin repeat containing, nuclear envelope 2	-1.5	4.0E-02
TMC6	transmembrane channel-like 6	-1.7	4.0E-02
BCL2	B-cell CLL/lymphoma 2	-1.6	4.0E-02
VSTM2L	V-set and transmembrane domain containing 2 like	-1.8	4.0E-02
ENSGALG00000016217	novel gene	-1.4	4.1E-02
PWP2	*PWP2* periodic tryptophan protein homolog (yeast)	-1.4	4.1E-02
GHOX-7	Homeobox protein *GHOX-7*	-1.5	4.1E-02
TLR21	Toll-like receptor 21 precursor	-1.6	4.1E-02
BBS5	Bardet-Biedl syndrome 5	-1.7	4.1E-02
DBR1	debranching RNA lariats 1	-1.4	4.1E-02
WIPF1	WAS/WASL interacting protein family, member 1	-1.5	4.1E-02
ENSGALG00000008803	Uncharacterized protein	-1.5	4.1E-02
TMEM18	transmembrane protein 18	-1.6	4.1E-02
MLLT3	myeloid/lymphoid or mixed-lineage leukemia (trithorax homolog, Drosophila); translocated to, 3	-1.6	4.1E-02
FIGNL1	fidgetin-like 1	-1.9	4.1E-02
HDAC2	histone deacetylase 2	-1.5	4.1E-02
CABP7	calcium binding protein 7	-1.6	4.1E-02
SH3RF3	SH3 domain containing ring finger 3	-1.7	4.1E-02
TLK1	tousled-like kinase 1	-1.4	4.2E-02
LIMS1	LIM and senescent cell antigen-like domains	-1.5	4.2E-02
TP53I11	tumor protein p53 inducible protein 11	-1.6	4.2E-02
USP43	ubiquitin specific peptidase 43	-2.8	4.2E-02
MAP4K4	mitogen-activated protein kinase kinase kinase kinase 4	-1.4	4.2E-02
ATP1B3	ATPase, Na+/K+ transporting, beta 3 polypeptide	-2.0	4.2E-02
NOC4L	nucleolar complex associated 4 homolog (S. cerevisiae)	-1.4	4.2E-02
TMEM201	transmembrane protein 201	-1.5	4.2E-02
RNASET2	ribonuclease T2	-1.5	4.2E-02
ENGASE	endo-beta-N-acetylglucosaminidase	-1.7	4.2E-02
PLK4	polo-like kinase 4	-2.0	4.2E-02
DEK	*DEK* oncogene	-1.3	4.3E-02
TOR2A	torsin family 2, member A	-1.5	4.3E-02
NSUN5	NOP2/Sun domain family, member 5	-1.5	4.3E-02
ENSGALG00000014551	Uncharacterized protein	-1.7	4.3E-02
ISM2	isthmin 2	-2.0	4.3E-02
POP1	processing of precursor 1, ribonuclease P/MRP subunit (S. cerevisiae)	-1.4	4.3E-02
HIPK1	homeodomain interacting protein kinase 1	-1.4	4.3E-02
C4H4ORF29	uncharacterized protein C4orf29 homolog	-1.5	4.3E-02
MAP4K3	mitogen-activated protein kinase kinase kinase kinase 3	-1.3	4.4E-02
ENSGALG00000027696	novel gene	-1.5	4.4E-02
SLC39A6	solute carrier family 39 (zinc transporter), member 6	-1.6	4.4E-02
CHSY1	chondroitin sulfate synthase 1	-1.3	4.4E-02
HADH	hydroxyacyl-CoA dehydrogenase	-1.5	4.4E-02
ACAD8	acyl-CoA dehydrogenase family, member 8	-1.5	4.4E-02
TNFRSF11B	tumor necrosis factor receptor superfamily, member 11b	-1.5	4.4E-02
GTSE1	G-2 and S-phase expressed 1	-2.3	4.4E-02
GPR56	G protein-coupled receptor 56	-1.5	4.4E-02
DPP6	dipeptidyl-peptidase 6	-1.4	4.5E-02
LPL	lipoprotein lipase	-1.9	4.5E-02

CALML4	calmodulin-like 4	-1.9	4.5E-02
ZCCHC8	zinc finger, CCHC domain containing 8	-1.3	4.5E-02
NASP	nuclear autoantigenic sperm protein (histone-binding)	-1.3	4.5E-02
LMF2	lipase maturation factor 2	-1.4	4.5E-02
ACSL4	acyl-CoA synthetase long-chain family member 4	-1.4	4.5E-02
ADPGK	ADP-dependent glucokinase	-1.5	4.5E-02
C5ORF30	Uncharacterized protein	-1.5	4.5E-02
FOXRED2	FAD-dependent oxidoreductase domain containing 2	-1.8	4.5E-02
SPC25	*SPC25*, NDC80 kinetochore complex component	-1.7	4.5E-02
NUDT5	nudix (nucleoside diphosphate linked moiety X)-type motif 5	-2.0	4.5E-02
NOX4	NADPH oxidase 4	-2.5	4.5E-02
QSER1	glutamine and serine rich 1	-1.3	4.6E-02
MYLK	myosin light chain kinase	-1.4	4.6E-02
LIFR	leukemia inhibitory factor receptor alpha	-1.4	4.6E-02
TMCO3	transmembrane and coiled-coil domains 3	-1.5	4.6E-02
CEP152	centrosomal protein 152kDa	-1.5	4.6E-02
GYLTL1B	glycosyltransferase-like 1B	-1.5	4.6E-02
LRIG1	leucine-rich repeats and immunoglobulin-like domains 1	-1.6	4.6E-02
NEK3	NIMA-related kinase 3	-1.8	4.6E-02
PAX-6	Paired box protein *Pax-6*	-1.9	4.6E-02
PDP2	pyruvate dehyrogenase phosphatase catalytic subunit 2	-1.6	4.6E-02
FN1	fibronectin 1	-1.5	4.6E-02
NFATC1	nuclear factor of activated T-cells, cytoplasmic, calcineurin-dependent 1	-3.4	4.6E-02
BCL10	B-cell CLL/lymphoma 10	-1.4	4.6E-02
COL4A3	collagen, type IV, alpha 3 (Goodpasture antigen)	-1.8	4.6E-02
FLVCR1	feline leukemia virus subgroup C cellular receptor 1	-2.1	4.6E-02
CREB1	cAMP responsive element binding protein 1	-1.3	4.7E-02
NUP43	nucleoporin 43kDa	-1.3	4.7E-02
ECI2	enoyl-CoA delta isomerase	-1.4	4.7E-02
MYNN	myoneurin	-1.4	4.7E-02
FBXO32	F-box protein 32	-1.6	4.7E-02
MBOAT1	membrane bound O-acyltransferase domain containing 1	-1.6	4.7E-02
ANKRD9	ankyrin repeat domain 9	-2.5	4.7E-02
TMEM237	transmembrane protein 237	-1.3	4.7E-02
EHMT1	euchromatic histone-lysine N-methyltransferase 1	-1.4	4.7E-02
PUS1	pseudouridylate synthase 1	-1.3	4.7E-02
P4HTM	prolyl 4-hydroxylase, transmembrane (endoplasmic reticulum)	-1.6	4.7E-02
KCTD14	potassium channel tetramerization domain containing 14	-1.8	4.7E-02
FEN1	flap structure-specific endonuclease 1	-1.4	4.7E-02
RASGEF1A	RasGEF domain family, member 1A	-1.6	4.7E-02
AGBL1	ATP/GTP binding protein-like 1	-1.3	4.8E-02
PRKD1	protein kinase D1	-1.5	4.8E-02
ENSGALG00000023742	novel gene	-1.7	4.8E-02
RRM1	ribonucleotide reductase M1	-1.3	4.8E-02
KIAA0556	*KIAA0556*	-1.4	4.8E-02
MTMR1	myotubularin related protein 1	-1.4	4.8E-02
CACNA2D2	calcium channel, voltage-dependent, alpha 2/delta subunit 2	-1.5	4.8E-02
CRYZL1	crystallin, zeta (quinone reductase)-like 1	-1.5	4.8E-02
CEP164	centrosomal protein 164kDa	-1.5	4.9E-02
SNORA62	small nucleolar RNA SNORA62/SNORA6 family	/0	4.9E-02
GPR162	G protein-coupled receptor 162	-1.5	4.9E-02
CHST6	carbohydrate (N-acetylglucosamine 6-O) sulfotransferase 6	-1.4	4.9E-02
DNA2	DNA replication helicase/nuclease 2	-1.6	4.9E-02
ATF4	activating transcription factor 4	-1.7	4.9E-02
ENSGALG00000015336	mature protein	-2.3	4.9E-02
MZT1	mitotic spindle organizing protein 1	-1.5	4.9E-02
QSOX1	quiescin Q6 sulfhydryl oxidase 1	-1.5	4.9E-02
METTL4	methyltransferase like 4	-2.3	4.9E-02
FBXO39	F-box protein 39	-2.7	4.9E-02
JDP2	Jun dimerization protein 2	-2.8	4.9E-02
TMEM175	transmembrane protein 175	-1.3	5.0E-02

ELMOD1	ELMO/CED-12 domain containing 1	-1.5	5.0E-02
EYA4	eyes absent homolog 4 (Drosophila)	-1.6	5.0E-02
C4ORF20	ufm1-specific protease 2	-1.4	5.0E-02
ENSGALG00000003492	smooth muscle protein phosphatase type 1-binding subunit	-1.4	5.0E-02
ST3GAL5	ST3 beta-galactoside alpha-2,3-sialyltransferase 5	-1.5	5.0E-02
MAP3K1	mitogen-activated protein kinase kinase kinase 1, E3 ubiquitin protein ligase	-1.9	5.0E-02

*p-values are corrected for multiple testing by the false discovery rate method as utilized by cuffdiff (version 2.1.1).

Table S4 Detected EQ gene-specific transcripts statistically increased in expression during EQ to FP transition.

Gene	Description	log2(Fold Change)	p-value*
RMST_8	Rhabdomyosarcoma 2 associated transcript conserved region 8	inf	1.2E-03
C7ORF53	Uncharacterized protein	inf	1.2E-03
PHF21B	PHD finger protein 21B	inf	1.2E-03
KCNE1	potassium voltage-gated channel, Isk-related family, member 1	inf	1.2E-03
RAB39A	*RAB39A*, member RAS oncogene family	inf	1.2E-03
ENSGALG00000027858	novel gene	inf	1.2E-03
ENSGALG00000005774	Na+/K+ transporting ATPase interacting 4	inf	1.2E-03
GPR160	Uncharacterized protein	inf	1.2E-03
ENSGALG00000025855	novel gene	inf	1.2E-03
PTPN5	protein tyrosine phosphatase, non-receptor type 5 (striatum-enriched)	7.8	1.2E-03
PNP	purine nucleoside phosphorylase	7.8	1.2E-03
HOPX	Homeodomain-only protein	7.4	1.2E-03
CLIC5	chloride intracellular channel 5	7.4	1.2E-03
LMO2	rhombotin-2	7.3	1.2E-03
RAB40B	Uncharacterized protein	7.2	1.2E-03
ENSGALG00000023818	novel gene	7.2	1.2E-03
BASP1	Brain acid soluble protein 1 homolog	6.9	1.2E-03
EPB49	dematin actin binding protein	6.7	1.2E-03
PTRF	polymerase I and transcript release factor	6.7	1.2E-03
IRAK4	interleukin-1 receptor-associated kinase 4	6.7	1.2E-03
FABP5	fatty acid binding protein 5 (psoriasis-associated)	6.7	1.2E-03
MFSD2B	major facilitator superfamily domain containing 2B	6.6	1.2E-03
ARHGAP20	Rho GTPase activating protein 20	6.5	1.2E-03
C12orf69	single-pass membrane protein with coiled-coil domains 3	6.5	1.2E-03
HSF4	heat shock factor protein 4	6.4	1.2E-03
ENSGALG00000028238	internexin neuronal intermediate filament protein, alpha	6.4	1.2E-03
PLEKHG4	pleckstrin homology domain containing, family G (with RhoGef domain) member 4**	6.3	1.2E-03
BFSP1	beaded filament structural protein 1, filensin	6.3	1.2E-03
PALM2	paralemmin 2	6.2	1.2E-03
PPP2R2C	protein phosphatase 2, regulatory subunit B, gamma	6.2	1.2E-03
SALL1	sal-like 1 (Drosophila)	6.2	1.2E-03
CASP7	caspase 7, apoptosis-related cysteine peptidase	6.1	1.2E-03
UPP1	uridine phosphorylase 1	6.1	1.2E-03
SASS6	spindle assembly abnormal protein 6 homolog	6.1	1.2E-03
GRIK3	glutamate receptor, ionotropic, kainate 3	6.1	1.2E-03
SPECC1	sperm antigen with calponin homology and coiled-coil domains 1	6.0	1.2E-03
GJA4	connexin 37	6.0	1.2E-03
ENSGALG00000012773	novel gene	5.9	1.2E-03
BIRC7	baculoviral IAP repeat containing 7	5.9	1.2E-03
FN3KRP	ketosamine-3-kinase	5.8	1.2E-03
TMEM47	transmembrane protein 47	5.7	1.2E-03
PKIB	protein kinase (cAMP-dependent, catalytic) inhibitor beta	5.7	1.2E-03
CTIF	CBP80/20-dependent translation initiation factor	5.7	1.2E-03
ABTB2	ankyrin repeat and BTB (POZ) domain containing 2	5.7	1.2E-03
PACSIN3	protein kinase C and casein kinase substrate in neurons 3	5.6	1.2E-03
FBLN5	fibulin 5	5.6	1.2E-03
CHRM4	muscarinic acetylcholine receptor M4	5.5	1.2E-03
STAMBPL1	STAM binding protein-like 1	5.4	1.2E-03
ATP8A2	ATPase, aminophospholipid transporter, class I, type 8A, member 2	5.4	1.2E-03
GDF10	growth differentiation factor 10	5.3	1.2E-03
HSPB8	heat shock 22kDa protein 8	5.3	1.2E-03
FAIM2	Fas apoptotic inhibitory molecule 2	5.3	1.2E-03

NT5C2	cytosolic purine 5,-nucleotidase	5.2	1.2E-03
ME1	NADP-dependent malic enzyme	5.2	1.2E-03
ESRRB	estrogen-related receptor beta	5.2	1.2E-03
PALM	paralemmin-1 isoform 2	5.2	1.2E-03
ADA	adenosine deaminase	5.2	1.2E-03
ARHGEF37	Rho guanine nucleotide exchange factor (GEF) 37	5.1	1.2E-03
ENSGALG00000001951	Uncharacterized protein	5.1	1.2E-03
NRCAM	neuronal cell adhesion molecule	5.0	1.2E-03
SCN3B	sodium channel, voltage-gated, type III, beta subunit	5.0	1.2E-03
ENSGALG00000004279	lectin, galactoside-binding, soluble, 12	5.0	1.2E-03
SCN5A	sodium channel, voltage-gated, type V, alpha subunit	4.9	1.2E-03
HEBP2	heme binding protein 2	4.9	1.2E-03
IGSF9B	immunoglobulin superfamily, member 9B	4.8	1.2E-03
LIMCH1	LIM and calponin homology domains 1	4.8	1.2E-03
UCH-L1	ubiquitin carboxyl-terminal hydrolase isozyme L1	4.7	1.2E-03
NEFL	neurofilament, light polypeptide	4.7	1.2E-03
ENC1	ectodermal-neural cortex 1 (with BTB domain)	4.7	1.2E-03
HRASLS	HRAS-like suppressor	4.7	1.2E-03
CRYBA4	beta-crystallin A4	4.7	1.2E-03
TNFAIP8	Tumor necrosis factor alpha-induced protein 8	4.7	1.2E-03
COL6A2	Collagen alpha-2(VI) chain	4.7	1.2E-03
CYYR1	cysteine/tyrosine-rich 1	4.7	1.2E-03
STARD9	StAR-related lipid transfer (START) domain containing 9	4.6	1.2E-03
RNF182	Uncharacterized protein	4.6	1.2E-03
TUBB6	Tubulin beta-5 chain	4.6	1.2E-03
NKAIN2	Na+/K+ transporting ATPase interacting 2	4.6	1.2E-03
DDHD1	DDHD domain containing 1	4.6	1.2E-03
TNFAIP8L3	tumor necrosis factor, alpha-induced protein 8-like 3	4.6	1.2E-03
MLIP	muscular LMNA-interacting protein	4.5	1.2E-03
DOK5	docking protein 5	4.5	1.2E-03
NT5DC1	5,-nucleotidase domain containing 1	4.5	1.2E-03
MYO1B	myosin IB	4.5	1.2E-03
GLRX	glutaredoxin-1	4.5	1.2E-03
CRYGN	gamma-crystallin N	4.5	1.2E-03
NPNT	nephronectin	4.5	1.2E-03
PPL	Periplakin; Uncharacterized protein	4.4	1.2E-03
ENSGALG00000022958	Uncharacterized protein	4.4	1.2E-03
MYOM2	M-protein, striated muscle	4.4	1.2E-03
PMP22	peripheral myelin protein 22	4.4	1.2E-03
HOMER2	homer homolog 2 (Drosophila)	4.4	1.2E-03
ADAMTS12	ADAM metallopeptidase with thrombospondin type 1 motif, 12	4.4	1.2E-03
SCG2	secretogranin II	4.3	1.2E-03
ACAP3	ArfGAP with coiled-coil, ankyrin repeat and PH domains 3	4.3	1.2E-03
LASP-2	nebulette non-muscle isoform	4.3	1.2E-03
CDH18	Cadherin; Uncharacterized protein	4.3	1.2E-03
WDR86	WD repeat domain 86	4.3	1.2E-03
NIF3L1	NIF3 NGG1 interacting factor 3-like 1 (S. cerevisiae)	4.3	1.2E-03
C9orf172	chromosome 9 open reading frame 172	4.3	1.2E-03
SORBS1	sorbin and SH3 domain containing 1**	4.3	1.2E-03
FAM69C	family with sequence similarity 69, member C	4.3	1.2E-03
CA10	carbonic anhydrase X	4.3	1.2E-03
RUNX2	runt-related transcription factor 2	4.3	1.2E-03
PDZD2	PDZ domain containing 2	4.2	1.2E-03
IFIH1	interferon-induced helicase C domain-containing protein 1	4.2	1.2E-03
TMEM246	transmembrane protein 246	4.2	1.2E-03
UBE2O	ubiquitin-conjugating enzyme E2O	4.2	1.2E-03
ENSGALG00000006003	Uncharacterized protein	4.2	1.2E-03
ENSGALG00000007710	Uncharacterized protein	4.2	1.2E-03
TUBB3	tubulin beta-4 chain	4.1	1.2E-03
NFKBIA	nuclear factor of kappa light polypeptide gene enhancer in B-cells inhibitor, alpha	4.0	1.2E-03
ASAP1	ArfGAP with SH3 domain, ankyrin repeat and PH domain 1	4.0	1.2E-03

LHCGR	Lutropin-choriogonadotropic hormone receptor	4.0	1.2E-03
DPYSL3	dihydropyrimidinase-related protein 3	3.9	1.2E-03
MRAS	ras-related protein *M-Ras*	3.9	1.2E-03
TRMT13	tRNA methyltransferase 13 homolog (S. cerevisiae)	3.9	1.2E-03
FIBIN	fin bud initiation factor homolog (zebrafish)	3.9	1.2E-03
MDFI	MyoD family inhibitor	3.9	1.2E-03
FAM46B	family with sequence similarity 46, member B	3.9	1.2E-03
GDPD5	glycerophosphodiester phosphodiesterase domain-containing protein 5	3.8	1.2E-03
FGF1	Fibroblast growth factor 1 Endothelial cell growth factor alpha	3.8	1.2E-03
NECAB1	N-terminal EF-hand calcium binding protein 1	3.8	1.2E-03
ENSGALG00000026188	novel gene	3.8	1.2E-03
SPHK1	sphingosine kinase 1	3.8	1.2E-03
SLC6A1	Transporter	3.8	1.2E-03
TTYH2	tweety homolog 2 (Drosophila)	3.8	1.2E-03
WDFY2	WD repeat and FYVE domain-containing protein 2	3.8	1.2E-03
DNAJB5	DnaJ (Hsp40) homolog, subfamily B, member 5	3.8	1.2E-03
RANBP3L	RAN binding protein 3-like	3.7	1.2E-03
SOX2	Transcription factor *SOX-2*	3.7	1.2E-03
SH3GL2	Endophilin-A1	3.7	1.2E-03
PIEZO2	piezo-type mechanosensitive ion channel component 2	3.7	1.2E-03
FAM159A	family with sequence similarity 159, member A	3.7	1.2E-03
DTNBP1	Dysbindin	3.7	1.2E-03
FAM222A	family with sequence similarity 222, member A	3.7	1.2E-03
NANP	N-acylneuraminate-9-phosphatase	3.6	1.2E-03
ELMO1	engulfment and cell motility protein 1	3.6	1.2E-03
DCLK2	doublecortin-like kinase 2	3.5	1.2E-03
B4GALT4	UDP-Gal:betaGlcNAc beta 1,4- galactosyltransferase, polypeptide 4	3.5	1.2E-03
NEDD9	neural precursor cell expressed, developmentally down-regulated 9	3.5	1.2E-03
PTN	pleiotrophin	3.5	1.2E-03
SDPR	serum deprivation response	3.4	1.2E-03
KALRN	kalirin, RhoGEF kinase	3.4	1.2E-03
CSDC2	cold shock domain containing C2, RNA binding	3.4	1.2E-03
HS3ST2	heparan sulfate (glucosamine) 3-O-sulfotransferase 2	3.4	1.2E-03
GRIK1	glutamate receptor, ionotropic, kainate 1	3.4	1.2E-03
TMCC3	transmembrane and coiled-coil domain family 3	3.4	1.2E-03
TSPAN15	tetraspanin-15	3.4	1.2E-03
SBDS	Ribosome maturation protein *SBDS*	3.4	1.2E-03
SBDS	Ribosome maturation protein *SBDS*	3.4	1.2E-03
ACSBG1	acyl-CoA synthetase bubblegum family member 1	3.4	1.2E-03
GSTA3	glutathione S-transferase alpha 3	3.3	1.2E-03
RFX2	regulatory factor X, 2 (influences HLA class II expression)	3.3	1.2E-03
TMCC2	transmembrane and coiled-coil domain family 2	3.3	1.2E-03
CMIP	c-Maf inducing protein	3.3	1.2E-03
PUS7L	pseudouridylate synthase 7 homolo	3.3	1.2E-03
FAM65B	protein *FAM65B*	3.3	1.2E-03
GPRC5B	G protein-coupled receptor, family C, group 5, member B	3.2	1.2E-03
FN3K	fructosamine 3 kinase	3.2	1.2E-03
LPHN3	latrophilin 3	3.2	1.2E-03
APBB1IP	amyloid beta A4 precursor protein-binding family B member 1-interacting protein	3.2	1.2E-03
RTN4IP1	reticulon 4 interacting protein 1	3.2	1.2E-03
C17ORF39	Uncharacterized protein	3.2	1.2E-03
EX-FABP	extracellular fatty acid-binding protein precursor	3.1	1.2E-03
CTNNA2	Catenin alpha-2	3.1	1.2E-03
PADI3	protein-arginine deiminase type-3	3.1	1.2E-03
NAA25	N-alpha-acetyltransferase 25, NatB auxiliary subunit	3.1	1.2E-03
ELOVL4	elongation of very long chain fatty acids protein 4	3.1	1.2E-03
CYP2H1	cytochrome P450 2H1 precursor	3.1	1.2E-03
SLC16A6	solute carrier family 16, member 6 (monocarboxylic acid	3.1	1.2E-03

	transporter 7)		
TBC1D8	TBC1 domain family, member 8 (with GRAM domain)	3.0	1.2E-03
MAPK10	mitogen-activated protein kinase 10	3.0	1.2E-03
ENSGALG00000005862	novel gene	3.0	1.2E-03
KIAA1324L	KIAA1324-like	3.0	1.2E-03
SPIRE1	spire homolog 1 (Drosophila)	3.0	1.2E-03
ENSGALG00000015366	Uncharacterized protein	2.9	1.2E-03
KIAA0226L	KIAA0226-like	2.9	1.2E-03
TMEM171	transmembrane protein 171	2.9	1.2E-03
RAI2	retinoic acid induced 2	2.9	1.2E-03
KTN1	kinectin	2.9	1.2E-03
RASGEF1B	RasGEF domain family, member 1B	2.9	1.2E-03
LRP11	low density lipoprotein receptor-related protein 11	2.9	1.2E-03
BIN1	bridging integrator 1	2.9	1.2E-03
ENSGALG00000027159	novel gene	2.9	1.2E-03
MASTL	microtubule associated serine/threonine kinase-like	2.8	1.2E-03
QRSL1	glutaminyl-tRNA synthase (glutamine-hydrolyzing)-like 1	2.8	1.2E-03
DOCK3	dedicator of cytokinesis 3	2.8	1.2E-03
MSRA	methionine sulfoxide reductase A	2.8	1.2E-03
ENSGALG00000005648	novel gene	2.8	1.2E-03
STK16	serine/threonine kinase 16	2.8	1.2E-03
GGT1	gamma-glutamyltransferase 1	2.8	1.2E-03
ME3	malic enzyme 3, NADP(+)-dependent, mitochondrial	2.8	1.2E-03
EDNRB	endothelin receptor type B precursor	2.8	1.2E-03
WNT5B	protein *Wnt-5b* precursor	2.8	1.2E-03
PCOLCE2	procollagen C-endopeptidase enhancer 2	2.7	1.2E-03
PTPN21	protein tyrosine phosphatase, non-receptor type 21	2.7	1.2E-03
ENSGALG00000020719	Uncharacterized protein	2.7	1.2E-03
ELOVL1	elongation of very long chain fatty acids protein 1	2.7	1.2E-03
TMTC2	transmembrane and tetratricopeptide repeat containing 2	2.7	1.2E-03
PDZRN4	PDZ domain containing ring finger 4	2.7	1.2E-03
ATG4B	cysteine protease *ATG4B*	2.7	1.2E-03
THSD4	thrombospondin, type I, domain containing 4	2.7	1.2E-03
HPRT1	hypoxanthine-guanine phosphoribosyltransferase	2.7	1.2E-03
WBP2	WW domain binding protein 2	2.6	1.2E-03
PAQR7	progestin and adipoQ receptor family member VII	2.6	1.2E-03
NCKIPSD	NCK interacting protein with SH3 domain	2.6	1.2E-03
ATG3	Autophagy-related protein 3	2.6	1.2E-03
ANKRD9	ankyrin repeat domain 9	2.6	1.2E-03
ENSGALG00000001166	novel gene	2.6	1.2E-03
CPEB3	cytoplasmic polyadenylation element binding protein 3	2.5	1.2E-03
ENSGALG00000005895	Uncharacterized protein	2.5	1.2E-03
MYEOV2	myeloma overexpressed 2	2.5	1.2E-03
ENSGALG00000005389	Uncharacterized protein	2.5	1.2E-03
PDE3B	cGMP-inhibited 3,,5,-cyclic phosphodiesterase B	2.5	1.2E-03
EAF2	ELL-associated factor 2	2.5	1.2E-03
PRRG3	proline rich Gla (G-carboxyglutamic acid) 3 (transmembrane)	2.5	1.2E-03
MAPK8IP2	mitogen-activated protein kinase 8 interacting protein 2	2.5	1.2E-03
EPS15L1	epidermal growth factor receptor pathway substrate 15-like 1	2.4	1.2E-03
PCSK2	proprotein convertase subtilisin/kexin type 2	2.4	1.2E-03
EZR	ezrin	2.4	1.2E-03
R3HDM2	R3H domain containing 2	2.3	1.2E-03
ENSGALG00000004032	Uncharacterized protein	2.3	1.2E-03
PIM3	*pim-3* oncogene	2.3	1.2E-03
CNP	2,,3,-cyclic nucleotide 3, phosphodiesterase	2.1	1.2E-03
PGGT1B	protein geranylgeranyltransferase type I, beta subunit	2.1	1.2E-03
LINGO1	Leucine-rich repeat and immunoglobulin-like domain-containing nogo receptor-interacting protein 1	2.1	1.2E-03
LRSAM1	leucine rich repeat and sterile alpha motif containing 1	2.1	1.2E-03
SPOCK2	sparc/osteonectin, cwcv and kazal-like domains proteoglycan (testican) 2	2.0	1.2E-03
IL2RG	interleukin 2 receptor, gamma	1.9	1.2E-03

GYPC	glycophorin C (Gerbich blood group)	5.3	2.0E-03
B3GNT5	UDP-GlcNAc:betaGal beta-1,3-N-acetylglucosaminyltransferase 5	5.2	2.0E-03
CGN	cingulin	5.2	2.0E-03
EPHB1	Ephrin type-B receptor 1	5.0	2.0E-03
RASGRP3	ras guanyl-releasing protein 3	3.6	2.0E-03
HUNK	hormonally up-regulated Neu-associated kinase	3.3	2.0E-03
SYN3	synapsin III	3.2	2.0E-03
ST8SIA4	CMP-N-acetylneuraminate-poly-alpha-2,8-sialyltransferase	3.1	2.0E-03
SLC24A3	solute carrier family 24 (sodium/potassium/calcium exchanger), member 3	2.9	2.0E-03
UBXD4	UBX domain-containing protein 2A	2.8	2.0E-03
HIGD1C	HIG1 domain family member 1A	2.8	2.0E-03
SBK1	Serine/threonine-protein kinase *SBK1*	2.6	2.0E-03
ENSGALG00000009458	novel gene	2.5	2.0E-03
RHOBTB3	Rho-related BTB domain containing 3	2.5	2.0E-03
HMX2	H6 family homeobox 2	2.4	2.0E-03
CDC20	cell division cycle protein 20 homolog	2.2	2.0E-03
SLC5A1	solute carrier family 5 (sodium/glucose cotransporter), member 1	2.2	2.0E-03
SDK1	protein sidekick-1 precursor	2.2	2.0E-03
FAR2	fatty acyl CoA reductase 2	2.2	2.0E-03
RNF146	ring finger protein 146	2.1	2.0E-03
ITGB1BP1	integrin beta 1 binding protein 1	2.1	2.0E-03
RNF151	ring finger protein 151	2.0	2.0E-03
MED10	mediator complex subunit 10	2.0	2.0E-03
FYCO1	FYVE and coiled-coil domain-containing protein 1	2.0	2.0E-03
PPRC1	peroxisome proliferator-activated receptor gamma, coactivator-related 1	1.9	2.0E-03
SAMD10	sterile alpha motif domain containing 10	6.5	2.8E-03
CP49	*CP49* protein; Uncharacterized protein	5.7	2.8E-03
GTF2A1L	TFIIA-alpha and beta-like factor	5.0	2.8E-03
PCMT1	Protein-L-isoaspartate(D-aspartate) O-methyltransferase	4.6	2.8E-03
SLC6A20	solute carrier family 6 (proline IMINO transporter), member 20	4.5	2.8E-03
ENSGALG00000025959	chromosome 3 open reading frame 83	3.4	2.8E-03
PIK3IP1	phosphoinositide-3-kinase interacting protein 1	3.1	2.8E-03
FAM189A1	family with sequence similarity 189, member A1	3.0	2.8E-03
MINPP1	multiple inositol polyphosphate phosphatase 1 precursor	3.0	2.8E-03
CAP2	Adenylyl cyclase-associated protein	2.8	2.8E-03
HSPB1	heat shock protein beta-1	2.6	2.8E-03
CHL1	cell adhesion molecule L1-like	2.6	2.8E-03
FAM126B	family with sequence similarity 126, member B	2.5	2.8E-03
INSIG1	insulin-induced gene 1 protein	2.5	2.8E-03
ASPR	substance-P receptor	2.5	2.8E-03
LIN54	*lin-54* homolog (C. elegans	2.5	2.8E-03
RPP38	ribonuclease P protein subunit p38	2.4	2.8E-03
GABRP	gamma-aminobutyric acid (GABA) A receptor, pi	2.4	2.8E-03
YWHAG	14-3-3 protein gamma	2.4	2.8E-03
BTBD3	BTB (POZ) domain containing 3	2.3	2.8E-03
GCC2	GRIP and coiled-coil domain containing 2	2.3	2.8E-03
TMEM64	transmembrane protein 64	2.3	2.8E-03
NUAK1	NUAK family, SNF1-like kinase, 1	2.2	2.8E-03
ENSGALG00000026539	novel gene	2.2	2.8E-03
UCKL1	Uridine kinase	2.1	2.8E-03
ZDHHC2	zinc finger, DHHC-type containing 2	2.0	2.8E-03
AHNAK	*AHNAK* nucleoprotein	1.9	2.8E-03
MKRN2	probable E3 ubiquitin-protein ligase makorin-2	1.9	2.8E-03
SLC39A8	solute carrier family 39 (zinc transporter), member 8	4.2	3.5E-03
DBC1	Deleted in bladder cancer protein 1 homolog	3.6	3.5E-03
GADD45	growth arrest and DNA-damage-inducible, alpha	3.2	3.5E-03
ABHD12	Monoacylglycerol lipase *ABHD12*	2.9	3.5E-03
ABLIM2	actin binding LIM protein family, member 2	2.8	3.5E-03

SBNO2	strawberry notch homolog 2 (Drosophila)	2.6	3.5E-03
MTOR	mechanistic target of rapamycin (serine/threonine kinase)	2.5	3.5E-03
ENSGALG00000002150	novel gene	2.3	3.5E-03
SDK2	protein sidekick-2	2.3	3.5E-03
WSCD1	WSC domain containing 1	2.3	3.5E-03
ZFYVE21	zinc finger, FYVE domain containing 21	2.1	3.5E-03
FAM129A	protein Niban	2.1	3.5E-03
DHRS11	Dehydrogenase/reductase SDR family member 11	2.0	3.5E-03
MLYCD	malonyl-CoA decarboxylase	2.0	3.5E-03
ENO2	gamma-enolase	2.0	3.5E-03
CEP41	Centrosomal protein of 41 kDa	2.0	3.5E-03
DHRS3	dehydrogenase/reductase (SDR family) member 3	1.9	3.5E-03
ASB1	ankyrin repeat and SOCS box containing 1	5.6	4.2E-03
CTNNA2	Catenin alpha-2	4.0	4.2E-03
CHANK1	Ankyrin 1; Uncharacterized protein	3.6	4.2E-03
BAI3	brain-specific angiogenesis inhibitor 3	3.4	4.2E-03
SCYL3	protein-associating with the carboxyl-terminal domain of ezrin	3.2	4.2E-03
KIT	Mast/stem cell growth factor receptor *Kit*	3.2	4.2E-03
RPIA	ribose-5-phosphate isomerase	2.9	4.2E-03
OTUD7B	OTU domain containing 7B	2.3	4.2E-03
PSEN2	presenilin-2	2.1	4.2E-03
SLC25A25	Uncharacterized protein	2.1	4.2E-03
MID2	Midline 2; Uncharacterized protein	1.9	4.2E-03
ST8SIA5	alpha-2,8-sialyltransferase 8E	3.7	4.8E-03
COBL	cordon-bleu WH2 repeat protein	3.4	4.8E-03
TTC9	tetratricopeptide repeat domain 9	3.2	4.8E-03
STMN4	Stathmin	2.8	4.8E-03
MAPK11	mitogen-activated protein kinase 11	2.5	4.8E-03
DYNC1I1	Uncharacterized protein	2.3	4.8E-03
DYNLL1	dynein, light chain, LC8-type 1	2.3	4.8E-03
CAV2	caveolin-2	2.2	4.8E-03
HCN2	hippocampus abundant transcript 1 protein	2.0	4.8E-03
HHLA2	HERV-H LTR-associating 2	1.9	4.8E-03
DNAH3	dynein, axonemal, heavy chain 3	1.8	4.8E-03
XIRP1	xin actin-binding repeat-containing protein 1	5.7	5.4E-03
BPGM	bisphosphoglycerate mutase	4.2	5.4E-03
PGAP2	post-GPI attachment to proteins 2	3.3	5.4E-03
FEZ1	fasciculation and elongation protein zeta 1 (zygin I)	3.2	5.4E-03
CDC34	Uncharacterized protein	3.2	5.4E-03
TLCD1	Calfacilitin	2.9	5.4E-03
SDCCAG3	serologically defined colon cancer antigen 3	2.4	5.4E-03
AMIGO2	amphoterin-induced protein 2 precursor	2.3	5.4E-03
MAP3K15	mitogen-activated protein kinase kinase kinase 15	2.3	5.4E-03
CLMN	calmin (calponin-like, transmembrane)	2.2	5.4E-03
SPON2	spondin 2, extracellular matrix protein	2.2	5.4E-03
RAD23A	RAD23 homolog A (S. cerevisiae)	2.1	5.4E-03
GGCT	gamma-glutamylcyclotransferase	2.0	5.4E-03
ENSGALG00000004078	novel gene	1.9	5.4E-03
IFRD1	interferon-related developmental regulator 1	4.4	6.0E-03
C10orf71	chromosome 10 open reading frame 71	4.0	6.0E-03
CEP104,LRRC47	leucine rich repeat containing 47	3.8	6.0E-03
KIF21A	kinesin family member 21A	2.2	6.0E-03
AGAP3	ArfGAP with GTPase domain, ankyrin repeat and PH domain 3	2.2	6.0E-03
FGFR1OP2	FGFR1 oncogene partner 2 homolog	2.0	6.0E-03
ECHDC1	enoyl CoA hydratase domain containing 1	2.0	6.0E-03
ENSGALG00000027680	novel gene	1.9	6.0E-03
STEAP3	STEAP family member 3, metalloreductase	1.8	6.0E-03
TMCO1	transmembrane and coiled-coil domains 1	1.8	6.0E-03
GLULD1	lengsin	7.5	6.5E-03
OVCH2	Uncharacterized protein	4.4	6.5E-03
LRTM2	leucine-rich repeats and transmembrane domains 2	3.3	6.5E-03

DGKB	diacylglycerol kinase, beta 90kDa	2.9	6.5E-03
KCTD20	potassium channel tetramerization domain containing 20	2.5	6.5E-03
TMEM117	transmembrane protein 117	2.4	6.5E-03
PITPNM2	phosphatidylinositol transfer protein, membrane-associated 2	2.4	6.5E-03
RTN4R	reticulon 4 receptor	2.3	6.5E-03
ARAP3	ArfGAP with RhoGAP domain, ankyrin repeat and PH domain 3	2.3	6.5E-03
AMD1	S-adenosylmethionine decarboxylase proenzyme	2.2	6.5E-03
TIPRL	TIP41, TOR signaling pathway regulator-like (S. cerevisiae)	2.2	6.5E-03
HSPH1	heat shock 105kDa	2.2	6.5E-03
THAP4	THAP domain containing 4	2.2	6.5E-03
IGBP1	immunoglobulin (CD79A) binding protein 1	2.1	6.5E-03
AKAP10	Uncharacterized protein	2.0	6.5E-03
ENSGALG00000011449	Uncharacterized protein	2.0	6.5E-03
NDUFA12	NADH dehydrogenase	1.8	6.5E-03
RANGAP1	Ran GTPase activating protein 1	4.0	7.0E-03
CXCL13L2	Chemokine	3.2	7.0E-03
NAT8L	Uncharacterized protein	3.0	7.0E-03
CAPRIN2	caprin family member 2	2.8	7.0E-03
BAG2	BCL2-associated athanogene 2	2.4	7.0E-03
GGA.46920	Uncharacterized protein	2.3	7.0E-03
LRR1	leucine rich repeat protein 1	2.3	7.0E-03
EPHA2	Uncharacterized protein	2.0	7.0E-03
GTDC2	Glycosyltransferase-like domain-containing protein 2	2.0	7.0E-03
MYO1E	myosin IE	1.7	7.0E-03
SRD5A2	steroid-5-alpha-reductase, alpha polypeptide 2 (3-oxo-5 alpha-steroid delta 4-dehydrogenase alpha 2)	3.6	7.5E-03
RBM38	RNA-binding protein 38 [	3.5	7.5E-03
ALS2CL	ALS2 C-terminal like	3.0	7.5E-03
C1QL1	complement component 1, q subcomponent-like 1	2.9	7.5E-03
FAM135B	family with sequence similarity 135, member B	2.3	7.5E-03
ENSGALG00000002955	novel gene	2.3	7.5E-03
ENSGALG00000004643	Protein-L-isoaspartate O-methyltransferase	1.9	7.5E-03
ENSGALG00000021692	novel gene	1.8	7.5E-03
ENSGALG00000026183	novel gene	1.8	7.5E-03
ZBTB17	zinc finger and BTB domain containing 17	1.7	7.5E-03
CRYBB1	Beta-crystallin B1	4.8	8.0E-03
DDB2	DNA damage-binding protein 2	4.6	8.0E-03
AHSG	alpha-2-HS-glycoprotein	4.5	8.0E-03
RANBP10	RAN binding protein 10	2.8	8.0E-03
LGALS1	16 kDa beta-galactoside-binding lectin	2.7	8.0E-03
ANKRD12	ankyrin repeat domain 12	1.8	8.0E-03
C2ORF18	Uncharacterized protein	1.7	8.0E-03
PSTPIP2	proline-serine-threonine phosphatase interacting protein 2	6.9	8.5E-03
PGM1	phosphoglucomutase-1	3.4	8.5E-03
KIF5C	kinesin family member 5C	2.2	8.5E-03
FAM129B	family with sequence similarity 129, member B	2.0	8.5E-03
SPTSSA	serine palmitoyltransferase, small subunit A	1.9	8.5E-03
SLC22A23	solute carrier family 22, member 23	1.9	8.5E-03
DPY30	protein *dpy-30* homolog	1.7	8.5E-03
CD3E	T-cell surface glycoprotein CD3 epsilon chain precursor	3.4	9.0E-03
MATN1	Cartilage matrix protein	3.0	9.0E-03
AFG3L2	AFG3 ATPase family member 3-like 2 (S. cerevisiae)	2.0	9.0E-03
EPS15	epidermal growth factor receptor substrate 15	3.4	9.5E-03
ENSGALG00000002128	Uncharacterized protein	2.8	9.5E-03
CHRNA7	neuronal acetylcholine receptor subunit alpha-7 precursor	2.8	1.0E-02
SLC25A22	mitochondrial glutamate carrier 1	5.4	1.0E-02
PENK	proenkephalin	4.7	1.0E-02
CARHSP1	calcium regulated heat stable protein 1, 24kDa	3.9	1.0E-02
FRMPD3	FERM and PDZ domain containing 3	2.7	1.0E-02
ANGIOPOIETIN-2	*angiopoietin-2*	2.5	1.0E-02
ENSGALG00000028363	AFG3 ATPase family member 3-like 2 (S. cerevisiae)	2.2	1.0E-02

SLC19A2	solute carrier family 19 (thiamine transporter), member 2	2.1	1.0E-02
RMI1	recQ-mediated genome instability protein 1	2.0	1.0E-02
ELL	RNA polymerase II elongation factor *ELL*	1.8	1.0E-02
WIPF2	WAS/WASL interacting protein family, member 2	1.6	1.0E-02
EMP2	epithelial membrane protein 2	3.0	1.1E-02
SLC28A3	solute carrier family 28 (concentrative nucleoside transporter), member 3	2.1	1.1E-02
NQO2	NAD(P)H dehydrogenase, quinone 2	1.7	1.1E-02
RFC2	Replication factor C subunit 2	1.7	1.1E-02
SH2D1B	SH2 domain containing 1B	4.9	1.1E-02
NTM	protein CEPU-1 precursor	4.4	1.1E-02
CRTAM	cytotoxic and regulatory T-cell molecule precursor	2.5	1.1E-02
EFR3B	EFR3 homolog B (S. cerevisiae)	2.5	1.1E-02
PACS2	phosphofurin acidic cluster sorting protein 2	2.3	1.1E-02
ENSGALG00000026460	Uncharacterized protein	2.2	1.1E-02
AKR1B10	aldo-keto reductase family 1, member B10 (aldose reductase)	1.9	1.1E-02
AKTIP	AKT-interacting protein	1.8	1.1E-02
ITA	Inhibitor of apoptosis protein	2.6	1.2E-02
ERAP1	endoplasmic reticulum aminopeptidase 1	2.3	1.2E-02
MAMDC4,PHPT1	phosphohistidine phosphatase 1	2.3	1.2E-02
SGMS2	sphingomyelin synthase 2	2.0	1.2E-02
TPST2	protein-tyrosine sulfotransferase 2 precursor	1.7	1.2E-02
PTTG1IP	pituitary tumor-transforming 1 interacting protein	1.6	1.2E-02
NPL	N-acetylneuraminate lyase	3.7	1.2E-02
APBA2	amyloid beta (A4) precursor protein-binding, family A, member 2	2.2	1.2E-02
SLC24A2	sodium/potassium/calcium exchanger 2	1.7	1.2E-02
TPST1	tyrosylprotein sulfotransferase 1	1.7	1.2E-02
LATS2	large tumor suppressor kinase 2	1.7	1.2E-02
TEKT3	tektin 3	3.5	1.3E-02
FGF16	fibroblast growth factor 16	3.5	1.3E-02
ART4	ecto-ADP-ribosyltransferase 4 precursor	3.4	1.3E-02
IRK1	inward rectifier potassium channel 2	3.0	1.3E-02
EPB41	Erythroid protein 4.1	2.9	1.3E-02
YWHAH	14-3-3 protein eta	2.6	1.3E-02
SYT12	synaptotagmin-12	2.3	1.3E-02
PROX2	prospero homeobox 2	2.2	1.3E-02
PSMG3	proteasome (prosome, macropain) assembly chaperone 3	1.7	1.3E-02
ESYT3	extended synaptotagmin-like protein 3	1.6	1.3E-02
SC5DL	sterol-C5-desaturase	2.8	1.3E-02
MTPN	myotrophin	2.6	1.3E-02
FAM190B	granule cell antiserum positive 14	2.3	1.3E-02
ATPAF2	ATP synthase mitochondrial F1 complex assembly factor 2	2.0	1.3E-02
DCLK1	doublecortin-like kinase 1	2.0	1.3E-02
TMEM86A	transmembrane protein 86A	3.2	1.4E-02
C10orf137	chromosome 10 open reading frame 137	2.1	1.4E-02
ATP6V0E2	ATPase, H+ transporting V0 subunit e2	1.8	1.4E-02
SLC24A6	solute carrier family 24 (sodium/lithium/calcium exchanger), member 6	1.7	1.4E-02
TP73	tumor protein p73	3.4	1.4E-02
FBXL20	F-box and leucine-rich repeat protein 20	2.8	1.4E-02
ASB9	ankyrin repeat and SOCS box protein 9	2.2	1.4E-02
RUSC1	RUN and SH3 domain containing 1	2.2	1.4E-02
SURF2	surfeit locus protein 2	1.7	1.4E-02
CORO6	coronin 6	1.6	1.4E-02
NIPA1	Uncharacterized protein	2.6	1.5E-02
GLS	glutaminase kidney isoform, mitochondrial precursor	2.3	1.5E-02
ENSGALG00000000549	urate (hydroxyiso-) hydrolase, pseudogene	2.3	1.5E-02
IGF2R	cation-independent mannose-6-phosphate receptor precursor	2.0	1.5E-02
FEM1A	Uncharacterized protein	1.9	1.5E-02
CAST	calpastatin	2.8	1.5E-02

ABCD2	ATP-binding cassette sub-family D member 2	2.3	1.5E-02
UNC13D	unc-13 homolog D (C. elegans)	2.1	1.6E-02
KIF1A	kinesin family member 1A	2.9	1.6E-02
RBM24	RNA-binding protein 24	3.5	1.6E-02
ANKIB1	ankyrin repeat and IBR domain containing 1	3.3	1.6E-02
VCL	Vinculin	1.9	1.6E-02
PAPLN	papilin, proteoglycan-like sulfated glycoprotein**	2.9	1.7E-02
METAP1	Methionine aminopeptidase 1	2.5	1.7E-02
BNIP3	BCL2/adenovirus E1B 19kDa interacting protein 3	2.1	1.7E-02
KANSL3	KAT8 regulatory NSL complex subunit 3	1.6	1.7E-02
C4ORF52	uncharacterized protein *C4orf52* homolog	1.6	1.7E-02
ENSGALG00000013848	novel gene	2.4	1.7E-02
MAP2K5	mitogen-activated protein kinase kinase 5	2.1	1.7E-02
DSG2	desmoglein 2	2.2	1.8E-02
MAP1B	Uncharacterized protein	1.8	1.8E-02
gga-mir-135a-2	*gga-mir-135a-2* [Source:miRBase;Acc:MI0001169]	inf	1.8E-02
TXNDC11	thioredoxin domain containing 11	2.9	1.8E-02
LSAMP	Limbic system-associated membrane protein	2.4	1.8E-02
SPATA2L	spermatogenesis associated 2-like	2.0	1.8E-02
STIL	SCL/TAL1 interrupting locus	1.8	1.8E-02
ST5	suppression of tumorigenicity 5	1.7	1.8E-02
SYNGR3	synaptogyrin-3	1.7	1.8E-02
ENSGALG00000023819	novel gene	6.9	1.9E-02
CELA2A	chymotrypsin-like elastase family member 2A precursor	3.0	1.9E-02
DIRAS1	DIRAS family, GTP-binding RAS-like 1	2.9	1.9E-02
ITGA4	integrin, alpha 4 (antigen CD49D, alpha 4 subunit of VLA-4 receptor)	2.2	1.9E-02
LMTK2	lemur tyrosine kinase 2	2.0	1.9E-02
C18ORF8	Uncharacterized protein	3.8	1.9E-02
FRYL	FRY-like	2.8	1.9E-02
KLF3	Kruppel-like factor 3 (basic)	2.0	1.9E-02
SYNPR	synaptoporin precursor	1.9	1.9E-02
STAT4	signal transducer and activator of transcription 4	4.1	1.9E-02
UBTD2	Uncharacterized protein	3.2	1.9E-02
ARFGAP3	ADP-ribosylation factor GTPase-activating protein 3	3.0	1.9E-02
RAPGEF6	Rap guanine nucleotide exchange factor (GEF) 6	2.4	1.9E-02
TWF1	twinfilin 1	2.0	1.9E-02
ROBO1	roundabout, axon guidance receptor, homolog 1	1.7	1.9E-02
GAB3	GRB2-associated binding protein 3	2.6	2.0E-02
CHODL	chondrolectin	4.2	2.0E-02
TBC1D14	TBC1 domain family member 14	1.8	2.0E-02
RAP2A	Uncharacterized protein	2.1	2.0E-02
F13A1	coagulation factor XIII A chain	1.9	2.0E-02
CACFD1	calcium channel flower domain containing 1	1.7	2.0E-02
RPE	ribulose-5-phosphate-3-epimerase	1.6	2.0E-02
PVALB	Parvalbumin, thymic	5.9	2.1E-02
C1orf43	chromosome 1 open reading frame 43	1.9	2.1E-02
ENSGALG00000003444	novel gene	1.8	2.1E-02
CZH18ORF25	Uncharacterized protein	1.8	2.1E-02
ENSGALG00000026110	SH3 and multiple ankyrin repeat domains 3	1.7	2.1E-02
NKIRAS1	NFKB inhibitor interacting Ras-like 1	1.5	2.1E-02
C2CD2	C2 calcium-dependent domain containing 2	2.0	2.1E-02
FAM100A	UBA-like domain containing 1	1.5	2.1E-02
GLI1	Zinc finger protein *GLI1*	2.5	2.2E-02
ENSGALG00000011324	novel gene	2.5	2.2E-02
CYP2D6	cytochrome P450, family 2, subfamily D, polypeptide 6	2.0	2.2E-02
ABCC3	ATP-binding cassette, sub-family C (CFTR/MRP), member 3	1.8	2.2E-02
CCM2	malcavernin	1.8	2.2E-02
FAM54B	Uncharacterized protein	1.6	2.2E-02
MED19	mediator complex subunit 19	1.5	2.2E-02
ENSGALG00000026793	novel gene	3.9	2.2E-02
ENSGALG00000025721	cytochrome P450, family 2, subfamily D, polypeptide 7 pseudogene 1	3.9	2.2E-02

UBE3B	Ubiquitin protein ligase; Uncharacterized protein	1.8	2.2E-02
OSBP2	oxysterol binding protein 2	1.5	2.2E-02
SLC38A7	solute carrier family 38, member 7	1.5	2.2E-02
FAM212B	family with sequence similarity 212, member B	3.5	2.2E-02
WDTC1	Uncharacterized protein	3.0	2.2E-02
41341	Translationally controlled tumor protein**	1.8	2.2E-02
ENSGALG00000015655	novel gene	5.9	2.3E-02
KCNK1	potassium channel, subfamily K, member 1	2.1	2.3E-02
CREBL2	cAMP responsive element binding protein-like 2	1.8	2.3E-02
ABTB1	ankyrin repeat and BTB/POZ domain-containing protein 1	1.8	2.3E-02
TRNT1	tRNA nucleotidyl transferase, CCA-adding, 1	1.7	2.3E-02
SNCG1	synuclein, gamma	2.5	2.3E-02
PBDC1	polysaccharide biosynthesis domain containing 1	2.0	2.3E-02
LRRK2	leucine-rich repeat kinase 2	1.8	2.3E-02
DOCK5	dedicator of cytokinesis 5	2.1	2.3E-02
GNPAT	glyceronephosphate O-acyltransferase	1.7	2.3E-02
TMEM123	transmembrane protein 123 precursor	1.7	2.3E-02
SIAH2	siah E3 ubiquitin protein ligase 2	1.6	2.4E-02
RPS6KA	Ribosomal protein S6 kinase 2 alpha	1.7	2.4E-02
MFSD6	Uncharacterized protein	1.6	2.4E-02
SEPX1	selenoprotein X, 1	1.4	2.4E-02
SPTAN1	spectrin alpha chain, brain	2.8	2.4E-02
PRKAB2	5,-AMP-activated protein kinase subunit beta-2	1.5	2.4E-02
UNC45B	unc-45 homolog B (C. elegans)	3.6	2.5E-02
ST18	suppression of tumorigenicity 18 (breast carcinoma) (zinc finger protein)	3.5	2.5E-02
ENSGALG00000009935	novel gene	2.1	2.5E-02
PHLDA2	pleckstrin homology-like domain family A member 2	1.9	2.5E-02
HSD17B4	peroxisomal multifunctional enzyme type 2	1.9	2.5E-02
ITSN2	intersectin 2	1.7	2.5E-02
CIT	citron (rho-interacting, serine/threonine kinase 21)	1.7	2.5E-02
NR2F1	nuclear receptor subfamily 2, group F, member 1	2.6	2.5E-02
CRCP	CGRP receptor component	2.4	2.5E-02
GPX1	glutathione peroxidase 1	2.3	2.5E-02
LDB3	LIM domain binding 3	2.1	2.5E-02
HECTD4	HECT domain containing E3 ubiquitin protein ligase 4	1.8	2.5E-02
OAF	Out at first protein homolog	1.8	2.5E-02
CYP39A1	cytochrome P450, family 39, subfamily A, polypeptide 1	1.5	2.5E-02
FBXW7	F-box and WD repeat domain containing 7, E3 ubiquitin protein ligase	1.4	2.5E-02
PRICKLE1	prickle homolog 1 (Drosophila)	1.9	2.6E-02
NDUFS5	NADH dehydrogenase (ubiquinone) Fe-S protein 5, 15kDa (NADH-coenzyme Q reductase)	1.7	2.6E-02
GGA.41926	Uncharacterized protein	1.7	2.6E-02
TIE1	tyrosine kinase with immunoglobulin-like and EGF-like domains 1	3.4	2.6E-02
FAM49B	family with sequence similarity 49, member B	2.1	2.6E-02
STAC	SH3 and cysteine rich domain	2.9	2.6E-02
EAF1	ELL associated factor 1	2.1	2.6E-02
ARHGEF10	Rho guanine nucleotide exchange factor (GEF) 10	2.1	2.7E-02
HSP70	heat shock 70 kDa protein	2.1	2.7E-02
CD5	T-cell surface glycoprotein *CD5* precursor	3.5	2.7E-02
CEPT1	choline/ethanolaminephosphotransferase 1	2.1	2.7E-02
SPG7	spastic paraplegia 7	1.8	2.7E-02
DENND4A	DENN/MADD domain containing 4A	1.6	2.7E-02
PTDSS1	Phosphatidylserine synthase 1	2.3	2.7E-02
ADCY5	adenylate cyclase type 5	2.1	2.7E-02
UBOX5	U-box domain containing 5	1.5	2.8E-02
ENSGALG00000023424	novel gene	2.0	2.8E-02
HABP4	Intracellular hyaluronan-binding protein 4	1.4	2.8E-02
SPTBN1	spectrin beta chain, brain 1	2.8	2.8E-02
PLEKHA1	pleckstrin homology domain containing, family A (phosphoinositide binding specific) member 1	1.9	2.9E-02

ZNF511	zinc finger protein 511	1.6	2.9E-02
KDM5A	lysine (K)-specific demethylase 5A	1.6	2.9E-02
HBS1L	HBS1-like (S. cerevisiae)	2.0	2.9E-02
CDK6	cell division protein kinase 6	1.5	3.0E-02
SLC37A2	solute carrier family 37 (glucose-6-phosphate transporter), member 2	1.5	3.0E-02
SNX20	sorting nexin 20	1.9	3.0E-02
SH3BGRL2	SH3 domain-binding glutamic acid-rich-like protein	5.4	3.0E-02
SUSD5	sushi domain containing 5	4.1	3.1E-02
ENSGALG00000027412	novel gene	2.3	3.1E-02
HINT3	histidine triad nucleotide binding protein 3	2.0	3.1E-02
TNRC6B	trinucleotide repeat containing 6B	2.6	3.1E-02
RAB7L1	RAB7, member RAS oncogene family-like 1	2.6	3.1E-02
RASSF2	ras association domain-containing protein 2	2.1	3.1E-02
HPSE	heparanase precursor	4.6	3.2E-02
TDRD7	Tudor domain-containing protein 7	2.4	3.2E-02
DCN	decorin precurso	2.3	3.2E-02
SCD	stearoyl-CoA desaturase 1	2.1	3.2E-02
FAM213A	Redox-regulatory protein *FAM213A*	1.9	3.2E-02
NT5C3	Cytosolic 5,-nucleotidase III	1.7	3.2E-02
SNTG1	syntrophin, gamma 1	1.5	3.2E-02
PDZD11	PDZ domain-containing protein 11	1.4	3.2E-02
CSDA	Y box binding protein 3	2.8	3.2E-02
GGA.17220,TOM1L2	Uncharacterized protein	2.0	3.2E-02
FDPS,NUP210L	nucleoporin 210kDa-like	1.7	3.2E-02
AKAP5	A kinase (PRKA) anchor protein 5	2.4	3.2E-02
DFFA	DNA fragmentation factor, 45kDa, alpha polypeptide	2.3	3.2E-02
C1ORF114	Uncharacterized protein	2.2	3.2E-02
KIAA0319L	KIAA0319-like	1.5	3.2E-02
PDHX	pyruvate dehydrogenase complex, component X	1.4	3.2E-02
UACA	uveal autoantigen with coiled-coil domains and ankyrin repeats	1.9	3.3E-02
ATP11C	Uncharacterized protein	1.7	3.3E-02
SFXN2	Uncharacterized protein	1.4	3.3E-02
ENSGALG00000015928	novel gene	2.8	3.4E-02
AANAT	Serotonin N-acetyltransferase	1.6	3.4E-02
MED8	Uncharacterized protein	1.5	3.4E-02
ATP6V0A1	V-type proton ATPase 116 kDa subunit a isoform 1	1.5	3.4E-02
HEATR5A	HEAT repeat containing 5A	1.5	3.4E-02
MED9	mediator complex subunit 9	1.4	3.4E-02
DGKQ	diacylglycerol kinase, theta 110kDa	1.5	3.4E-02
CDKL2	cyclin-dependent kinase-like 2 (CDC2-related kinase)	2.9	3.4E-02
SERPINE2	serpin peptidase inhibitor, clade E (nexin, plasminogen activator inhibitor type 1), member 2	1.7	3.4E-02
GATAD1	GATA zinc finger domain containing 1	1.5	3.4E-02
PRSS23	protease, serine, 23	3.3	3.5E-02
KIAA1522	*KIAA1522*	2.0	3.5E-02
SLC22A15	solute carrier family 22, member 15	2.0	3.5E-02
ZNF628	zinc finger protein 628	1.7	3.5E-02
PRPSAP1	phosphoribosyl pyrophosphate synthetase-associated protein 1	2.2	3.5E-02
UBE2G2	ubiquitin-conjugating enzyme E2G 2	2.2	3.6E-02
ENSGALG00000011528	novel gene	1.7	3.6E-02
ZDHHC8	zinc finger, DHHC-type containing 8	1.6	3.6E-02
CMC4	C-x(9)-C motif containing 4 homolog (S. cerevisiae)	1.4	3.6E-02
SNORD37	small nucleolar RNA, C/D box 37	0.2	3.6E-02
LCA5L	Leber congenital amaurosis 5-like	5.9	3.6E-02
EPB41L1	erythrocyte membrane protein band 4.1-like 1	2.1	3.6E-02
DDHD2	DDHD domain containing 2	2.0	3.6E-02
WNK1	WNK lysine deficient protein kinase 1	1.9	3.6E-02
PNRC1	proline-rich nuclear receptor coactivator 1	2.7	3.7E-02
DNAAF2	dynein, axonemal, assembly factor 2	1.7	3.7E-02
MRPS7	mitochondrial ribosomal protein S7	2.1	3.8E-02

CLTB	clathrin, light chain B	2.1	3.8E-02
TACC1	transforming, acidic coiled-coil containing protein 1	1.5	3.8E-02
C10ORF11	chromosome 10 open reading frame 11	1.7	3.9E-02
ZFYVE28	zinc finger, FYVE domain containing 28	1.5	4.0E-02
STAT5	signal transducer and activator of transcription 5A	2.1	4.0E-02
RARRES1	retinoic acid receptor responder (tazarotene induced) 1	2.3	4.0E-02
ENSGALG00000027645	novel gene	1.5	4.0E-02
SRSF4	serine/arginine-rich splicing factor 4	1.4	4.0E-02
ASTL	astacin-like metallo-endopeptidase (M12 family)	2.0	4.1E-02
RXRG	retinoid X receptor, gamma	1.8	4.1E-02
LSM12	*LSM12* homolog (S. cerevisiae)	1.4	4.1E-02
LYSMD2	LysM, putative peptidoglycan-binding, domain containing 2	1.4	4.1E-02
SEC31B	SEC31 homolog B (S. cerevisiae)	1.4	4.1E-02
PRKAR2B	protein kinase, cAMP-dependent, regulatory, type II, beta	3.3	4.1E-02
NELL2	NEL-like 2	1.7	4.1E-02
DNAJC15	DnaJ (Hsp40) homolog, subfamily C, member 15	1.4	4.1E-02
MRS2	*MRS2* magnesium transporter	1.3	4.1E-02
SNX18	sorting nexin 18	1.4	4.2E-02
IQGAP2	IQ motif containing GTPase activating protein 2	1.5	4.2E-02
ENSGALG00000006897	novel gene	1.7	4.2E-02
ANGEL1	angel homolog 1 (Drosophila)	1.8	4.3E-02
ATP6V1D	ATPase, H+ transporting, lysosomal 34kDa, V1 subunit D	1.8	4.3E-02
CISD3	CDGSH iron sulfur domain 3	1.6	4.3E-02
CCDC67	coiled-coil domain containing 67	6.8	4.3E-02
SNPH	syntaphilin	5.4	4.3E-02
COPS4	COP9 signalosome subunit 4	1.6	4.3E-02
BTAF1	*BTAF1* RNA polymerase II, B-TFIID transcription factor-associated, 170kDa	1.6	4.3E-02
BTBD10	BTB (POZ) domain containing 10	3.0	4.4E-02
ENSGALG00000026480	novel gene	1.6	4.4E-02
RPL3L	ribosomal protein L3-like	3.2	4.4E-02
TESC	tescalcin	2.2	4.4E-02
PYGL	phosphorylase, glycogen, liver	1.6	4.4E-02
RPS6KL1	ribosomal protein S6 kinase-like 1	1.4	4.4E-02
HCN3	hyperpolarization activated cyclic nucleotide-gated potassium channel 3	3.1	4.4E-02
FGF22	fibroblast growth factor 22	2.7	4.5E-02
CLIP1	CAP-GLY domain containing linker protein 1	1.8	4.5E-02
FAM188B2	family with sequence similarity 188, member B2	2.8	4.5E-02
NDUFB10	NADH dehydrogenase (ubiquinone) 1 beta subcomplex, 10, 22kDa	1.7	4.5E-02
SPG20	spastic paraplegia 20 (Troyer syndrome)	1.7	4.5E-02
SFXN1	sideroflexin 1	1.7	4.5E-02
SH3BGRL3	SH3 domain binding glutamic acid-rich protein like 3	1.4	4.5E-02
C8ORF22	chromosome 8 open reading frame 22	2.3	4.6E-02
FAM162A	family with sequence similarity 162, member A	2.0	4.6E-02
PNPLA7	patatin-like phospholipase domain containing 7	1.8	4.6E-02
ENSGALG00000020592	novel gene	3.6	4.6E-02
ENSGALG00000026680	novel gene	2.9	4.6E-02
RILPL1	Rab interacting lysosomal protein-like 1	1.9	4.6E-02
C7ORF25	chromosome 7 open reading frame 25	1.4	4.7E-02
DEPDC7,TCP11L1	t-complex 11, testis-specific-like 1	2.5	4.7E-02
FAM20A	family with sequence similarity 20, member A	2.0	4.7E-02
IGSF21	immunoglobin superfamily, member 21	1.7	4.7E-02
RCAN2	regulator of calcineurin 2	2.0	4.8E-02
ETV4	ets variant 4	1.6	4.8E-02
F5	coagulation factor V (proaccelerin, labile factor)	1.3	4.8E-02
GABRG4	Gamma-aminobutyric acid receptor subunit gamma-4	1.7	4.8E-02
CLPX	*ClpX* caseinolytic peptidase X homolog (E. coli)	1.5	4.8E-02
ALAS1	aminolevulinate, delta-, synthase 1	1.5	4.9E-02
SPTY2D1	SPT2, Suppressor of Ty, domain containing 1 (S. cerevisiae)	1.4	4.9E-02
MPV17L2	MPV17 mitochondrial membrane protein-like 2	1.3	4.9E-02
PPIL6	peptidylprolyl isomerase (cyclophilin)-like 6	3.3	5.0E-02

ZNF503	zinc finger protein 503	3.1	5.0E-02
HMOX1	heme oxygenase (decycling) 1	2.8	5.0E-02

*p-values are corrected for multiple testing by the false discovery rate method as utilized by cuffdiff (version 2.1.1).

Table S5 Detected FP gene-specific transcripts statistically decreased in expression during FP to FC transition.

Gene	Description	log2(Fold Change)	p-value*
ENSGALG00000007692	Uncharacterized protein	/0	1.2E-03
CGN	cingulin	-5.0	6.0E-03
ENSGALG00000026793	novel gene	-3.1	6.5E-03
R3HDML	R3H domain containing-like	-2.0	8.0E-03
SNORD37	Small nucleolar RNA *SNORD37*	/0	1.6E-02
ENSGALG00000025721	cytochrome P450, family 2, subfamily D, polypeptide 7 pseudogene 1	-2.0	2.2E-02
PENK	proenkephalin	-1.9	2.7E-02
ENSGALG00000025721	cytochrome P450, family 2, subfamily D, polypeptide 7 pseudogene 1	/0	3.1E-02
snoU83B	Small nucleolar RNA U83B	/0	4.3E-02
ENSGALG00000026680	novel gene	-2.0	4.8E-02

*p-values are corrected for multiple testing by the false discovery rate method as utilized by cuffdiff (version 2.1.1).

Table S6 Detected FP gene-specific transcripts statistically increased in expression during FP to FC transition.

Gene	Description	log2(Fold Change)	p-value*
gga-mir-10a	*gga-mir-10a* [Source:miRBase;Acc:MI0007559]	inf	1.2E-03
C10ORF47	Uncharacterized protein	3.4	4.2E-03
SLC35E4	solute carrier family 35, member E4	inf	4.8E-03
TRAIL-LIKE	TNF-related apoptosis inducing ligand-*like* protein precursor	2.7	4.8E-03
ENSGALG00000010944	novel gene	2.3	1.8E-02
ENSGALG00000028414	novel gene	2.8	1.8E-02
LONRF2	LON peptidase N-terminal domain and ring finger 2	2.8	2.6E-02
ENSGALG00000026665	novel gene	1.1	4.2E-02
NUPL2	nucleoporin-like protein 2	2.0	4.6E-02
gga-mir-1618	*gga-mir-1618* [Source:miRBase;Acc:MI0007347]	inf	4.7E-02
ENSGALG00000011233	novel gene	2.6	4.9E-02

*p-values are corrected for multiple testing by the false discovery rate method as utilized by cuffdiff (version 2.1.1).

Table S7 Nuclear encoded mitochondrial protein transcript that demonstrated a two-fold decrease in expression or greater during EC to EQ transition. Detected FPKM and fold change (Δ) shown.

Symbol	EC	EQ	Δ	Description
**LDHA**	4469.11	771.07	-5.80	lactate dehydrogenase A
**HK2**	21.34	5.45	-3.92	hexokinase 2
**ACSL4**	50.55	13.05	-3.87	acyl-CoA synthetase long-chain family member 4
**GATM**	0.36	0.09	-3.78	glycine amidinotransferase (L-arginine:glycine amidinotransferase)
**PRSS35**	12.87	3.41	-3.77	protease, serine, 35
**TAP1**	1.36	0.39	-3.52	transporter 1, ATP-binding cassette, sub-family B (MDR/TAP)
**SDSL**	1.80	0.52	-3.46	serine dehydratase-like
**HAO2**	0.56	0.18	-3.22	hydroxyacid oxidase 2 (long chain)
**OGG1**	10.88	3.39	-3.20	8-oxoguanine DNA glycosylase
**CKMT1A**	1.37	0.44	-3.14	creatine kinase, mitochondrial 1A
**TDRKH**	0.48	0.16	-3.09	tudor and KH domain containing
**AIFM3**	1.96	0.67	-2.92	apoptosis-inducing factor, mitochondrion-associated, 3
**ARG2**	159.12	58.90	-2.70	arginase, type II
**ELN**	0.75	0.31	-2.46	elastin (supravalvular aortic stenosis, Williams-Beuren syndrome)
**CYP11A1**	1.53	0.62	-2.44	cytochrome P450, family 11, subfamily A, polypeptide 1
**SLC25A29**	6.60	2.80	-2.36	solute carrier family 25, member 29
**ARMC4**	0.26	0.12	-2.23	armadillo repeat containing 4
**SLC25A37**	2.82	1.35	-2.10	solute carrier family 25, member 37
**SNPH**	0.75	0.36	-2.09	syntaphilin
**COQ9**	9.49	4.66	-2.04	coenzyme Q9 homolog (S. cerevisiae)
**MUTYH**	2.93	1.45	-2.03	mutY homolog (E. coli)

Table S8 Nuclear encoded mitochondrial protein transcript that demonstrated a two-fold increase in expression or greater during EC to EQ transition. Detected FPKM and fold change (Δ) shown.

Symbol	EC	EQ	Δ	Description
**NME4**	0.22	1.19	5.34	non-metastatic cells 4, protein expressed in
**GPAM**	2.81	9.54	3.40	glycerol-3-phosphate acyltransferase, mitochondrial
**MRPL39**	10.44	34.55	3.31	mitochondrial ribosomal protein L39
**BCKDHB**	4.73	14.00	2.96	branched chain keto acid dehydrogenase E1, beta polypeptide (maple syrup urine disease)
**BBOX1**	1.50	3.92	2.61	butyrobetaine (gamma), 2-oxoglutarate dioxygenase (gamma-butyrobetaine hydroxylase) 1
**AGR2**	0.23	0.60	2.59	anterior gradient 2 homolog (Xenopus laevis)
**TSHZ3**	0.74	1.89	2.56	teashirt family zinc finger 3
**ATP10D**	1.54	3.92	2.54	ATPase, Class V, type 10D
**IDH1**	53.10	130.93	2.47	isocitrate dehydrogenase 1 (NADP+), soluble
**NCOA4**	21.37	52.42	2.45	nuclear receptor coactivator 4
**SQRDL**	1.76	4.24	2.41	sulfide quinone reductase-like (yeast)
**TOMM70A**	23.57	56.63	2.40	translocase of outer mitochondrial membrane 70 homolog A (S. cerevisiae)
**LYRM1**	10.58	25.30	2.39	LYR motif containing 1
**AK2**	23.62	56.27	2.38	adenylate kinase 2
**MAOA**	2.63	6.20	2.35	monoamine oxidase A
**SLC25A38**	1.46	3.36	2.30	solute carrier family 25, member 38
**IDE**	10.66	24.40	2.29	insulin-degrading enzyme
**ALDH2**	22.08	49.66	2.25	aldehyde dehydrogenase 2 family (mitochondrial)
**MRPS14**	8.95	19.85	2.22	mitochondrial ribosomal protein S14
**GLRX**	9.47	20.81	2.20	glutaredoxin (thioltransferase)
**GTPBP5**	4.41	9.55	2.16	GTP binding protein 5 (putative)
**ABHD10**	2.65	5.71	2.16	abhydrolase domain containing 10
**KYNU**	0.46	0.98	2.15	kynureninase (L-kynurenine hydrolase)
**MCAT**	14.46	30.59	2.12	malonyl CoA:ACP acyltransferase (mitochondrial)
**COMTD1**	2.64	5.45	2.06	catechol-O-methyltransferase domain containing 1
**TIMM23**	13.74	28.27	2.06	translocase of inner mitochondrial membrane 23 homolog (yeast)
**DUSP26**	0.74	1.51	2.04	dual specificity phosphatase 26 (putative)
**HMGCS2**	0.65	1.33	2.04	3-hydroxy-3-methylglutaryl-Coenzyme A synthase 2 (mitochondrial)
**MTX2**	26.46	53.22	2.01	metaxin 2
**NDUFA10**	40.07	80.42	2.01	NADH dehydrogenase (ubiquinone) 1 alpha subcomplex, 10, 42kDa
**NDUFA4**	93.79	187.91	2.00	NADH dehydrogenase (ubiquinone) 1 alpha subcomplex, 4, 9kDa

Table S9 Nuclear encoded mitochondrial protein transcript that demonstrated a two-fold decrease in expression or greater during EQ to FP transition. Detected FPKM and fold change (Δ) shown.

Symbol	EQ	FP	Δ	Description
**MAOA**	6.2	0.6	-10.2	monoamine oxidase A
**SLC16A1**	210.8	25.8	-8.2	solute carrier family 16, member 1 (monocarboxylic acid transporter 1)
**SARDH**	15.9	2.1	-7.5	sarcosine dehydrogenase
**AGMAT**	0.8	0.1	-7.1	agmatine ureohydrolase (agmatinase)
**PDK1**	12.6	1.8	-6.9	pyruvate dehydrogenase kinase, isozyme 1
**TXNRD1**	2.8	0.4	-6.6	thioredoxin reductase 1
**ARG2**	58.9	9.0	-6.5	arginase, type II
**EFHD1**	5.9	1.0	-6.2	EF-hand domain family, member D1
**ATP10D**	3.9	0.7	-5.6	ATPase, Class V, type 10D
**DUT**	10.4	1.9	-5.6	dUTP pyrophosphatase
**ALDH18A1**	12.9	2.6	-5.0	aldehyde dehydrogenase 18 family, member A1
**PHYHIPL**	3.9	0.8	-4.7	phytanoyl-CoA 2-hydroxylase interacting protein-like
**LACTB2**	5.2	1.1	-4.6	lactamase, beta 2
**TSHZ3**	1.9	0.4	-4.5	teashirt family zinc finger 3
**CERK**	11.1	2.5	-4.4	ceramide kinase
**CRY1**	22.8	5.3	-4.3	cryptochrome 1 (photolyase-like)
**OMA1**	5.4	1.3	-4.2	OMA1 homolog, zinc metallopeptidase (S. cerevisiae)
**ACSL1**	9.0	2.2	-4.0	acyl-CoA synthetase long-chain family member 1
**AS3MT**	19.8	5.0	-3.9	arsenic (+3 oxidation state) methyltransferase
**NME4**	1.2	0.3	-3.8	non-metastatic cells 4, protein expressed in
**SLC25A3**	395.5	104.9	-3.8	solute carrier family 25 (mitochondrial carrier; phosphate carrier), member 3
**UNG**	10.7	2.9	-3.7	uracil-DNA glycosylase
**POLG**	4.3	1.2	-3.7	polymerase (DNA directed), gamma
**BCKDHB**	14.0	3.9	-3.6	branched chain keto acid dehydrogenase E1, beta polypeptide (maple syrup urine disease)
**CYP27A1**	6.2	1.8	-3.4	cytochrome P450, family 27, subfamily A, polypeptide 1
**EHHADH**	7.7	2.2	-3.4	enoyl-Coenzyme A, hydratase/3-hydroxyacyl Coenzyme A dehydrogenase
**EARS2**	2.4	0.7	-3.4	glutamyl-tRNA synthetase 2 (mitochondrial)(putative)
**DDAH1**	54.9	16.4	-3.3	dimethylarginine dimethylaminohydrolase 1
**BID**	9.7	2.9	-3.3	BH3 interacting domain death agonist
**FARS2**	20.7	6.4	-3.2	phenylalanine-tRNA synthetase 2 (mitochondrial)
**ALDH4A1**	7.4	2.3	-3.2	aldehyde dehydrogenase 4 family, member A1
**ALDH5A1**	7.4	2.3	-3.2	aldehyde dehydrogenase 5 family, member A1 (succinate-semialdehyde dehydrogenase)
**PPIF**	5.3	1.7	-3.1	peptidylprolyl isomerase F (cyclophilin F)
**GALC**	1.7	0.5	-3.1	galactosylceramidase
**SLC25A6**	294.3	94.2	-3.1	solute carrier family 25 (mitochondrial carrier; adenine nucleotide translocator), member 6
**PPM1K**	8.7	2.8	-3.1	protein phosphatase 1K (PP2C domain containing)
**SLC25A12**	9.9	3.3	-3.0	solute carrier family 25 (mitochondrial carrier, Aralar), member 12
**PDP2**	6.6	2.2	-3.0	pyruvate dehydrogenase phosphatase isoenzyme 2

**SLC25A13**	14.0	4.7	-3.0	solute carrier family 25, member 13 (citrin)
**BCL2**	6.2	2.1	-3.0	B-cell CLL/lymphoma 2
**POLG2**	13.5	4.7	-2.9	polymerase (DNA directed), gamma 2, accessory subunit
**DBT**	15.1	5.3	-2.9	dihydrolipoamide branched chain transacylase E2
**ACAD8**	8.7	3.1	-2.9	acyl-Coenzyme A dehydrogenase family, member 8
**HDDC2**	32.3	11.3	-2.8	HD domain containing 2
**PISD**	4.3	1.5	-2.8	phosphatidylserine decarboxylase
**TIMM44**	93.0	33.0	-2.8	translocase of inner mitochondrial membrane 44 homolog (yeast)
**GCAT**	8.2	2.9	-2.8	glycine C-acetyltransferase (2-amino-3-ketobutyrate coenzyme A ligase)
**KYNU**	1.0	0.4	-2.8	kynureninase (L-kynurenine hydrolase)
**ACOT9**	5.4	1.9	-2.8	acyl-CoA thioesterase 9
**FDX1**	5.5	2.0	-2.8	ferredoxin 1
**ACSM3**	0.8	0.3	-2.8	acyl-CoA synthetase medium-chain family member 3
**HADH**	58.0	21.2	-2.7	hydroxyacyl-Coenzyme A dehydrogenase
**ACSL4**	13.1	4.8	-2.7	acyl-CoA synthetase long-chain family member 4
**ISCU**	31.4	11.6	-2.7	IscU iron-sulfur cluster scaffold homolog (E. coli)
**NT5M**	11.4	4.2	-2.7	5',3'-nucleotidase, mitochondrial
**AGXT2**	1.2	0.4	-2.7	alanine-glyoxylate aminotransferase 2
**COMT**	88.3	33.7	-2.6	catechol-O-methyltransferase
**ECHDC3**	35.4	13.6	-2.6	enoyl Coenzyme A hydratase domain containing 3
**NUDT2**	9.0	3.5	-2.6	nudix (nucleoside diphosphate linked moiety X)-type motif 2
**SUPV3L1**	8.1	3.2	-2.6	suppressor of var1, 3-like 1 (S. cerevisiae)
**MPST**	35.0	13.7	-2.6	mercaptopyruvate sulfurtransferase
**MDH1**	112.7	44.2	-2.5	malate dehydrogenase 1, NAD (soluble)
**ALDH7A1**	33.3	13.2	-2.5	aldehyde dehydrogenase 7 family, member A1
**DHTKD1**	7.8	3.1	-2.5	dehydrogenase E1 and transketolase domain containing 1
**CPOX**	5.2	2.1	-2.5	coproporphyrinogen oxidase
**CYP24A1**	0.4	0.2	-2.5	cytochrome P450, family 24, subfamily A, polypeptide 1
**ADCK2**	6.5	2.6	-2.5	aarF domain containing kinase 2
**SLC25A15**	7.9	3.2	-2.5	solute carrier family 25 (mitochondrial carrier; ornithine transporter) member 15
**TST**	31.7	13.0	-2.4	thiosulfate sulfurtransferase (rhodanese)
**RILP**	2.1	0.9	-2.4	Rab interacting lysosomal protein
**SLC25A29**	2.8	1.2	-2.4	solute carrier family 25, member 29
**COX18**	2.4	1.0	-2.4	COX18 cytochrome c oxidase assembly homolog (S. cerevisiae)
**QDPR**	13.8	5.9	-2.3	quinoid dihydropteridine reductase
**NUDT19**	8.2	3.5	-2.3	nudix (nucleoside diphosphate linked moiety X)-type motif 19
**PCK2**	4.4	1.9	-2.3	phosphoenolpyruvate carboxykinase 2 (mitochondrial)
**SLC25A4**	24.7	10.7	-2.3	solute carrier family 25 (mitochondrial carrier; adenine nucleotide translocator), member 4
**MRPL15**	7.9	3.5	-2.3	mitochondrial ribosomal protein L15
**GNG5**	24.0	10.5	-2.3	guanine nucleotide binding protein (G protein), gamma 5
**IDH1**	130.9	57.3	-2.3	isocitrate dehydrogenase 1 (NADP+), soluble
**ABCB10**	4.3	1.9	-2.3	ATP-binding cassette, sub-family B (MDR/TAP), member 10
**THG1L**	6.9	3.1	-2.3	tRNA-histidine guanylyltransferase 1-like (S. cerevisiae)
**PARL**	15.2	6.8	-2.2	presenilin associated, rhomboid-like
**CA5B**	6.3	2.8	-2.2	carbonic anhydrase VB, mitochondrial

**RFK**	3.2	1.4	-2.2	riboflavin kinase
**FAHD1**	4.2	1.9	-2.2	fumarylacetoacetate hydrolase domain containing 1
**SERHL2**	28.1	12.8	-2.2	serine hydrolase-like 2
**NFS1**	9.9	4.5	-2.2	NFS1 nitrogen fixation 1 homolog (S. cerevisiae)
**GLRX5**	11.1	5.0	-2.2	glutaredoxin 5 homolog (S. cerevisiae)
**ZADH2**	9.0	4.1	-2.2	zinc binding alcohol dehydrogenase, domain containing 2
**ACADSB**	4.9	2.3	-2.2	acyl-Coenzyme A dehydrogenase, short/branched chain
**HSDL2**	26.5	12.2	-2.2	hydroxysteroid dehydrogenase like 2
**RAB8B**	10.2	4.7	-2.1	RAB8B, member RAS oncogene family
**NME2**	1960.6	912.7	-2.1	non-metastatic cells 2, protein (NM23B) expressed in
**CLIC4**	76.0	35.4	-2.1	chloride intracellular channel 4
**MRPL51**	14.6	6.8	-2.1	mitochondrial ribosomal protein L51
**BRP44L**	18.5	8.7	-2.1	brain protein 44-like
**SUCLG2**	5.4	2.5	-2.1	succinate-CoA ligase, GDP-forming, beta subunit
**ARMC1**	8.8	4.1	-2.1	armadillo repeat containing 1
**GFM2**	6.2	3.0	-2.1	G elongation factor, mitochondrial 2
**NME1**	4.6	2.2	-2.1	non-metastatic cells 1, protein (NM23A) expressed in
**GLDC**	23.3	11.1	-2.1	glycine dehydrogenase (decarboxylating)
**SLC25A36**	9.2	4.4	-2.1	solute carrier family 25, member 36
**PANK2**	16.2	7.8	-2.1	pantothenate kinase 2 (Hallervorden-Spatz syndrome)
**TOMM70A**	56.6	27.5	-2.1	translocase of outer mitochondrial membrane 70 homolog A (S. cerevisiae)
**MRPS6**	66.3	32.3	-2.1	mitochondrial ribosomal protein S6
**SLC30A6**	8.6	4.2	-2.0	solute carrier family 30 (zinc transporter), member 6
**LYRM2**	44.5	21.8	-2.0	LYR motif containing 2
**LDHA**	771.1	381.7	-2.0	lactate dehydrogenase A
**GLUD1**	25.0	12.4	-2.0	glutamate dehydrogenase 1
**RPL35A**	315.6	157.2	-2.0	ribosomal protein L35a
**CYP11A1**	0.6	0.3	-2.0	cytochrome P450, family 11, subfamily A, polypeptide 1

Table S10 Nuclear encoded mitochondrial protein transcript that demonstrated a two-fold increase in expression or greater during EQ to FP transition. Detected FPKM and fold change (Δ) shown.

Symbol	EQ	FP	Δ	Description
**SLC25A22**	0.8	32.5	42.5	solute carrier family 25 (mitochondrial carrier: glutamate), member 22
**SNPH**	0.4	14.6	40.8	syntaphilin
**ME1**	1.1	41.5	38.0	malic enzyme 1, NADP(+)-dependent, cytosolic
**GLRX**	20.8	469.8	22.6	glutaredoxin (thioltransferase)
**NIF3L1**	43.5	847.2	19.5	NIF3 NGG1 interacting factor 3-like 1 (S. pombe)
**RTN4IP1**	15.5	141.3	9.1	reticulon 4 interacting protein 1
**QRSL1**	7.5	53.8	7.2	glutaminyl-tRNA synthase (glutamine-hydrolyzing)-like 1
**MSRA**	10.1	71.2	7.1	methionine sulfoxide reductase A
**ME3**	8.1	55.3	6.9	malic enzyme 3, NADP(+)-dependent, mitochondrial
**TDRKH**	0.2	0.9	6.0	tudor and KH domain containing
**GLS**	12.7	63.9	5.0	glutaminase
**GPX1**	109.2	549.7	5.0	glutathione peroxidase 1
**ABCD2**	2.8	14.1	5.0	ATP-binding cassette, sub-family D (ALD), member 2
**MRPS7**	17.7	78.1	4.4	mitochondrial ribosomal protein S7
**SLC25A25**	3.9	16.5	4.3	solute carrier family 25 (mitochondrial carrier; phosphate carrier), member 25
**DMGDH**	0.1	0.6	4.2	dimethylglycine dehydrogenase
**DHRS1**	3.3	13.8	4.1	dehydrogenase/reductase (SDR family) member 1
**MLYCD**	3.4	13.9	4.1	malonyl-CoA decarboxylase
**AKAP10**	11.7	47.0	4.0	A kinase (PRKA) anchor protein 10
**ATPAF2**	10.5	42.0	4.0	ATP synthase mitochondrial F1 complex assembly factor 2
**AFG3L2**	11.4	45.6	4.0	AFG3 ATPase family gene 3-like 2 (yeast)
**DUSP26**	1.5	5.7	3.8	dual specificity phosphatase 26 (putative)
**HSD17B4**	9.1	33.8	3.7	hydroxysteroid (17-beta) dehydrogenase 4
**TMTC1**	0.4	1.5	3.6	transmembrane and tetratricopeptide repeat containing 1
**NDUFA12**	70.5	247.6	3.5	NADH dehydrogenase (ubiquinone) 1 alpha subcomplex, 12
**MRPS18C**	8.2	27.6	3.4	mitochondrial ribosomal protein S18C
**NDUFS5**	54.4	182.0	3.3	NADH dehydrogenase (ubiquinone) Fe-S protein 5, 15kDa (NADH-coenzyme Q reductase)
**NDUFB10**	126.7	424.3	3.3	NADH dehydrogenase (ubiquinone) 1 beta subcomplex, 10, 22kDa
**TRNT1**	37.6	125.4	3.3	tRNA nucleotidyl transferase, CCA-adding, 1
**NT5C3**	13.4	44.4	3.3	5'-nucleotidase, cytosolic III
**FDPS**	74.8	241.1	3.2	farnesyl diphosphate synthase (farnesyl pyrophosphate synthetase, dimethylallyltranstransferase, geranyltranstransferase)
**SFXN1**	58.0	184.2	3.2	sideroflexin 1
**ASAH2**	0.2	0.8	3.1	N-acylsphingosine amidohydrolase (non-lysosomal ceramidase) 2
**GATM**	0.1	0.3	3.1	glycine amidinotransferase (L-arginine:glycine amidinotransferase)
**RDH14**	3.5	10.4	3.0	retinol dehydrogenase 14 (all-trans/9-cis/11-cis)
**MTIF3**	27.1	77.9	2.9	mitochondrial translational initiation factor 3
**CLPX**	17.5	49.2	2.8	ClpX caseinolytic peptidase X homolog (E. coli)
**ALAS1**	11.8	33.2	2.8	aminolevulinate, delta-, synthase 1

**PDHX**	3.4	9.3	2.7	pyruvate dehydrogenase complex, component X
**SOD1**	108.1	282.2	2.6	superoxide dismutase 1, soluble (amyotrophic lateral sclerosis 1 (adult))
**SFXN2**	9.9	25.7	2.6	sideroflexin 2
**RAB11A**	24.6	63.4	2.6	RAB11A, member RAS oncogene family
**DNAJC15**	11.3	29.3	2.6	DnaJ (Hsp40) homolog, subfamily C, member 15
**MRPL2**	23.8	60.7	2.5	mitochondrial ribosomal protein L2
**TFB1M**	6.2	15.8	2.5	transcription factor B1, mitochondrial
**TIMM23**	28.3	70.0	2.5	translocase of inner mitochondrial membrane 23 homolog (yeast)
**SLC25A43**	13.1	32.3	2.5	solute carrier family 25, member 43
**PMPCA**	29.1	69.4	2.4	peptidase (mitochondrial processing) alpha
**HADHA**	27.2	63.4	2.3	hydroxyacyl-Coenzyme A dehydrogenase/3-ketoacyl-Coenzyme A thiolase/enoyl-Coenzyme A hydratase (trifunctional protein), alpha subunit
**NEU4**	5.2	11.9	2.3	sialidase 4
**COQ2**	2.1	4.7	2.3	coenzyme Q2 homolog, prenyltransferase (yeast)
**SLC25A10**	63.9	143.2	2.2	solute carrier family 25 (mitochondrial carrier; dicarboxylate transporter), member 10
**NDUFA2**	116.0	256.3	2.2	NADH dehydrogenase (ubiquinone) 1 alpha subcomplex, 2, 8kDa
**LARS2**	9.7	21.4	2.2	leucyl-tRNA synthetase 2, mitochondrial
**TRIAP1**	16.4	35.7	2.2	TP53 regulated inhibitor of apoptosis 1
**GARS**	28.2	60.4	2.1	glycyl-tRNA synthetase
**DACT2**	11.3	24.1	2.1	dapper, antagonist of beta-catenin, homolog 2 (Xenopus laevis)
**DNAJA3**	20.8	44.3	2.1	DnaJ (Hsp40) homolog, subfamily A, member 3
**CPS1**	0.5	1.1	2.1	carbamoyl-phosphate synthetase 1, mitochondrial
**TBRG4**	14.6	30.4	2.1	transforming growth factor beta regulator 4
**COQ4**	15.6	32.5	2.1	coenzyme Q4 homolog (S. cerevisiae)
**NDUFS8**	104.8	210.3	2.0	NADH dehydrogenase (ubiquinone) Fe-S protein 8, 23kDa (NADH-coenzyme Q reductase)
**SLC25A38**	3.4	6.7	2.0	solute carrier family 25, member 37
**ATPAF1**	10.0	20.0	2.0	ATP synthase mitochondrial F1 complex assembly factor 1
