# Supplementary material for: Differentiation State-Specific Mitochondrial Dynamic Regulatory Networks Are Revealed by Global Transcriptional Analysis of the Developing Chicken Lens
Source: G3 (Bethesda). 2014 Jun 13;4(8):1515–27. doi: 10.1534/g3.114.012120 (PMC4132181; doi:10.1534/g3.114.012120)
Supplement: Supporting Information [file supp_g3.114.012120_TableS1.pdf]

Table S1 Detected EC gene-specific transcripts statistically decreased in expression during EC to EQ transition.

Gene	Description	log2(Fold Change)	p-value*
SERPINI1	Neuroserpin	-2.1	1.2E-03
NANOS1	nanos homolog 1 (Drosophila)	-2.1	1.2E-03
APOA1	Apolipoprotein A-I	-2.2	1.2E-03
FABP7	Fatty acid-binding protein, brain	-2.2	1.2E-03
MAPK10	mitogen-activated protein kinase 10	-2.2	1.2E-03
MGP	Matrix Gla protein	-2.2	1.2E-03
FAM169A	family with sequence similarity 169, member A	-2.4	1.2E-03
DPP6	dipeptidyl-peptidase 6	-2.4	1.2E-03
PCDH1	protocadherin-1 precursor	-2.4	1.2E-03
SLC6A1	Transporter	-2.4	1.2E-03
DCP1A	decapping mRNA 1A	-2.4	1.2E-03
SOD3	Superoxide dismutase [Cu-Zn]	-2.4	1.2E-03
KIAA1239	*KIAA1239*	-2.4	1.2E-03
SCUBE3	signal peptide, CUB domain, EGF-like 3	-2.5	1.2E-03
CHMP4C	chromatin modifying protein 4C	-2.6	1.2E-03
HBAD	Hemoglobin subunit alpha-D	-2.6	1.2E-03
HBAA	Hemoglobin subunit alpha-A	-2.6	1.2E-03
ALDH6	aldehyde dehydrogenase family 1 member A3	-2.6	1.2E-03
TFRC	transferrin receptor protein 1	-2.7	1.2E-03
CLEC19A	C-type lectin domain family 19, member A	-2.7	1.2E-03
SIX6	Homeobox protein *SIX6*	-2.7	1.2E-03
CRABP-I	Cellular retinoic acid-binding protein 1	-2.7	1.2E-03
ENSGALG00000005470	novel gene	-2.7	1.2E-03
CNTN3	contactin 3 (plasmacytoma associated)	-2.7	1.2E-03
SLCO4A1	solute carrier organic anion transporter family member 4A1	-2.7	1.2E-03
HBB	Hemoglobin subunit beta	-2.7	1.2E-03
EFEMP1	EGF containing fibulin-like extracellular matrix protein 1	-2.8	1.2E-03
RDH10	retinol dehydrogenase 10 (all-trans)	-2.8	1.2E-03
SLC45A4	solute carrier family 45, member 4	-2.9	1.2E-03
MYLK	myosin light chain kinase, smooth muscle	-2.9	1.2E-03
GPR20	Uncharacterized protein	-2.9	1.2E-03
NPR3	natriuretic peptide receptor C/guanylate cyclase C (atrionatriuretic peptide receptor C)	-2.9	1.2E-03
LRRC38	leucine rich repeat containing 38	-2.9	1.2E-03
ENSGALG00000013280	novel gene	-2.9	1.2E-03
LPL	Lipoprotein lipase	-2.9	1.2E-03
AXDND1	axonemal dynein light chain domain containing 1	-2.9	1.2E-03
AKR	aldo-keto reductase family 1 member B10	-3.0	1.2E-03
TSPAN4	tetraspanin 4	-3.0	1.2E-03
SLC5A7	high affinity choline transporter 1	-3.0	1.2E-03
GALNT5	UDP-N-acetyl-alpha-D-galactosamine:polypeptide N-acetylgalactosaminyltransferase 5 (GalNAc-T5)	-3.1	1.2E-03
PCDHA11	protocadherin alpha 11 precursor	-3.1	1.2E-03
COL9A2	collagen, type IX, alpha 2	-3.1	1.2E-03
WBSCR17	Williams-Beuren syndrome chromosome region 17	-3.2	1.2E-03
FAM5B	family with sequence similarity 5, member B	-3.2	1.2E-03
PARM1	prostate androgen-regulated mucin-like protein 1	-3.3	1.2E-03
LGR5	leucine-rich repeat containing G protein-coupled receptor 5	-3.3	1.2E-03
TMEM72	transmembrane protein 72	-3.3	1.2E-03
NXPH2	neurexophilin 2	-3.3	1.2E-03
VIPR1	vasoactive intestinal polypeptide receptor 1 precursor	-3.4	1.2E-03
KCNT1	Potassium channel subfamily T member 1	-3.5	1.2E-03
FAR-2	contactin-5 precursor	-3.5	1.2E-03
ADPRHL1	ADP-ribosylhydrolase like 1	-3.6	1.2E-03
GEM	GTP-binding protein *GEM*	-3.6	1.2E-03
CPXM2	carboxypeptidase X (M14 family), member 2	-3.8	1.2E-03
SLC29A4	solute carrier family 29 (equilibrative nucleoside transporter), member 4	-3.9	1.2E-03

ADAMTS3	ADAM metallopeptidase with thrombospondin type 1 motif, 3	-4.0	1.2E-03
PTPRO	receptor-type tyrosine-protein phosphatase O	-4.3	1.2E-03
CYTL1	cytokine-like 1	-4.3	1.2E-03
RGS2	regulator of G-protein signaling 2	-4.4	1.2E-03
R3HDML	R3H domain containing-like	-4.4	1.2E-03
CHGB	chromogranin B (secretogranin 1)	-4.6	1.2E-03
PLEKHG4	pleckstrin homology domain containing, family G (with RhoGef domain) member 4 **	-4.7	1.2E-03
CGN	cingulin	-5.2	1.2E-03
DKK-1	*Dkk-1*; Uncharacterized protein	-5.6	1.2E-03
RASD1	dexamethasone-induced Ras-related protein 1	-5.7	1.2E-03
SIVA1	*SIVA1*, apoptosis-inducing factor	-1.9	2.0E-03
COL5A1	collagen alpha-1(V) chain precursor	-1.9	2.0E-03
FSTL4	follistatin-related protein 4 precursor	-2.2	2.0E-03
CLSTN2	calsyntenin 2	-2.3	2.0E-03
ZIC1	zinc finger protein *ZIC 1*	-2.4	2.0E-03
LRFN5	leucine rich repeat and fibronectin type III domain containing 5	-2.6	2.0E-03
FMO6P	flavin containing monooxygenase 6 pseudogene	-2.7	2.0E-03
STK17A	serine/threonine-protein kinase 17A	-2.8	2.0E-03
PTRF	polymerase I and transcript release factor	-3.0	2.0E-03
ST3GAL1	CMP-N-acetylneuraminate-beta-galactosamide-alpha-2,3-sialyltransferase 1	-3.1	2.0E-03
VWF	von Willebrand factor	-3.1	2.0E-03
COL6A1	collagen alpha-1(VI) chain precursor	-3.5	2.0E-03
PLCD1	Uncharacterized protein	-2.0	2.8E-03
MGAT4A	Alpha-1,3-mannosyl-glycoprotein 4-beta-N-acetylglucosaminyltransferase A	-2.1	2.8E-03
UBE2C	ubiquitin-conjugating enzyme E2C	-2.2	2.8E-03
ENSGALG00000023581	Uncharacterized protein	-2.3	2.8E-03
COL12A1	collagen alpha-1(XII) chain precursor	-2.4	2.8E-03
NEFM	neurofilament medium polypeptide	-3.5	2.8E-03
CYP26B1	cytochrome P450, family 26, subfamily B, polypeptide 1	-3.7	2.8E-03
CACNG5	calcium channel, voltage-dependent, gamma subunit 5	-1.9	3.5E-03
SOCS2	suppressor of cytokine signaling 2	-2.0	3.5E-03
CDH11	cadherin-11 precursor	-2.4	3.5E-03
GAS1	growth arrest-specific 1	-2.8	3.5E-03
SPAG5	sperm associated antigen 5	-1.8	4.2E-03
AGRN	agrin	-2.0	4.2E-03
THBS4	thrombospondin 4	-2.1	4.2E-03
PDLIM3	PDZ and LIM domain protein 3	-2.2	4.2E-03
CPA6	carboxypeptidase A6	-2.3	4.2E-03
GRIK3	glutamate receptor, ionotropic, kainate 3	-2.4	4.2E-03
KCNS1	potassium voltage-gated channel, delayed-rectifier, subfamily S, member 1	-2.6	4.2E-03
LY6E	lymphocyte antigen 6E precursor	-1.9	4.8E-03
CAMK1D	calcium/calmodulin-dependent protein kinase ID	-2.2	4.8E-03
TLL1	tolloid-like protein 1 precursor	-2.4	4.8E-03
IGSF21	immunoglobin superfamily, member 21	-2.6	4.8E-03
CYR61	Protein *CYR61*	-2.8	4.8E-03
HTRA3	HtrA serine peptidase 3	-2.1	5.4E-03
GGA.4981	epsilon globin	-2.1	5.4E-03
MKP3	dual specificity protein phosphatase 6	-2.2	5.4E-03
NRN1	neuritin precursor	-2.7	5.4E-03
VEGFA	Vascular endothelial growth factor A	-2.0	6.0E-03
SHISA2	protein *shisa-2* homolog precursor	-2.1	6.0E-03
VSX2	Visual system homeobox 2	-2.7	6.0E-03
FAM110C	family with sequence similarity 110, member C	-1.8	6.5E-03
PDE4D	phosphodiesterase 4D, cAMP-specific	-2.0	6.5E-03
MDGA2	MAM domain containing glycosylphosphatidylinositol anchor 2	-2.2	6.5E-03
RASSF9	Ras association (RalGDS/AF-6) domain family (N-terminal)	-2.2	6.5E-03

	member 9		
PCDH8	protocadherin-8 precursor	-2.3	6.5E-03
ENSGALG00000028527	novel gene	-3.0	6.5E-03
C1QTNF4	C1q and tumor necrosis factor related protein 4	-3.3	6.5E-03
SLITRK6	SLIT and NTRK-like family, member 6	-2.4	7.0E-03
LAMC2	laminin, gamma 2	-2.7	7.0E-03
CCDC3	coiled-coil domain containing 3	-3.0	7.0E-03
ENSGALG00000006325	Uncharacterized protein	-3.1	7.0E-03
SEZ6L	seizure related 6 homolog (mouse)-like	-3.6	7.0E-03
LEPREL4	leprecan-like 4	-1.7	8.0E-03
GCH1	GTP cyclohydrolase 1	-1.8	8.0E-03
LOXL3	lysyl oxidase-like 3	-1.9	8.0E-03
PI15	Peptidase inhibitor 15	-2.0	8.0E-03
HSPB1	heat shock protein beta-1	-2.1	8.0E-03
ALDH3B1,ENSGALG00000003490	Aldehyde dehydrogenase	-2.4	8.0E-03
PCDH18	protocadherin 18	-3.0	8.0E-03
FBN2	Fibrillin-3; Uncharacterized protein	-1.8	9.5E-03
CACNA1G	calcium channel, voltage-dependent, T type, alpha 1G subunit	-1.8	1.0E-02
LINGO3	leucine rich repeat and Ig domain containing 3	-3.4	1.0E-02
VAV3	guanine nucleotide exchange factor *VAV3*	-1.7	1.1E-02
TFAP2C	transcription factor AP-2 gamma (activating enhancer binding protein 2 gamma)	-1.8	1.1E-02
ACSL4	acyl-CoA synthetase long-chain family member 4	-2.0	1.1E-02
IRK1	inward rectifier potassium channel 2	-3.2	1.1E-02
R3HCC1L	R3H domain and coiled-coil containing 1-like	-1.7	1.1E-02
RAX1	retinal homeobox protein Rx2	-2.2	1.1E-02
PAPLN	papilin, proteoglycan-like sulfated glycoprotein**	-3.0	1.1E-02
PIK3C2G	phosphatidylinositol-4-phosphate 3-kinase, catalytic subunit type 2 gamma	-2.3	1.2E-02
CSDC2	cold shock domain containing C2, RNA binding	-1.6	1.3E-02
ANLN	anillin, actin binding protein	-1.7	1.3E-02
LTF	ovotransferrin precursor	-2.1	1.3E-02
COL9A3	collagen alpha-3(IX) chain precursor	-2.2	1.3E-02
ZIC3	Uncharacterized protein	-2.6	1.3E-02
COL1A2	collagen alpha-2(I) chain precursor	-1.6	1.4E-02
NR2F1	nuclear receptor subfamily 2, group F, member 1	-3.0	1.4E-02
PODXL	podocalyxin-like	-2.0	1.4E-02
ENO2	gamma-enolase	-1.6	1.5E-02
NTN1	Netrin-1	-2.6	1.5E-02
ENSGALG00000012847	novel gene	-1.7	1.6E-02
CPN1	Uncharacterized protein	-2.2	1.6E-02
CXCR4	C-X-C chemokine receptor type 4	-2.6	1.6E-02
NIN	ninein (GSK3B interacting protein)	-1.6	1.6E-02
PLA2G10	phospholipase A2, group X	-2.2	1.6E-02
ISLR2	uncharacterized protein LOC429941 precursor	-2.4	1.6E-02
HEXB	hexosaminidase B (beta polypeptide)	-1.5	1.7E-02
SPATA5	protein sprouty homolog 1	-1.6	1.7E-02
GPX8	Glutathione peroxidase	-2.0	1.7E-02
SH3TC2	SH3 domain and tetratricopeptide repeats 2	-2.0	1.7E-02
ENSGALG00000005747	Uncharacterized protein	-2.2	1.8E-02
CTNND2	catenin (cadherin-associated protein), delta 2	-1.5	1.8E-02
ARHGAP19	rho GTPase-activating protein 19	-1.6	1.9E-02
FAIM2	Fas apoptotic inhibitory molecule 2	-1.9	1.9E-02
PALB2	partner and localizer of BRCA2	-1.9	1.9E-02
ENSGALG00000023973	Uncharacterized protein	-2.3	1.9E-02
KIAA1324L	KIAA1324-like	-1.7	2.0E-02
WFDC1	WAP four-disulfide core domain protein 1	-1.6	2.0E-02
CKS1B	CDC28 protein kinase regulatory subunit 1B	-1.6	2.0E-02
PDIA5	protein disulfide isomerase family A, member 5	-1.7	2.0E-02
NEIL1	endonuclease VIII-like 1	-1.9	2.1E-02
ATP8A2	ATPase, aminophospholipid transporter, class I, type 8A,	-1.6	2.2E-02

	member 2		
SLC39A12	solute carrier family 39 (zinc transporter), member 12	-2.0	2.2E-02
CHRD	chordin precursor	-2.3	2.2E-02
ZIC2	Zic family member 2	-2.4	2.3E-02
ENSGALG00000015363	Uncharacterized protein	-1.7	2.3E-02
VEPH1	ventricular zone expressed PH domain-containing 1	-2.3	2.3E-02
ENSGALG00000014513	novel gene	-1.6	2.3E-02
PLA2G15	phospholipase A2, group XV	-1.8	2.3E-02
SNX16	sorting nexin-16	-1.5	2.4E-02
SH3RF2	SH3 domain containing ring finger 2	-2.2	2.4E-02
PLK2	polo-like kinase 2	-1.5	2.4E-02
MMP11	matrix metallopeptidase 11 (stromelysin 3)	-1.9	2.5E-02
PLOD1	procollagen-lysine,2-oxoglutarate 5-dioxygenase 1 precursor	-2.3	2.5E-02
NBL1	Neuroblastoma suppressor of tumorigenicity 1	-1.8	2.5E-02
CDC45	cell division cycle 45	-1.5	2.6E-02
COL9A1	collagen alpha-1(IX) chain precursor	-2.0	2.6E-02
CHRDL1	chordin-like protein 1 precursor	-2.0	2.6E-02
BMP4	Bone morphogenetic protein 4	-2.1	2.6E-02
LECT1	leukocyte cell-derived chemotaxin 1	-2.2	2.6E-02
PAM	peptidylglycine alpha-amidating monooxygenase	-1.6	2.6E-02
CLDN19	Uncharacterized protein	-2.0	2.6E-02
OLFM1	Noelin	-2.1	2.6E-02
HK2	hexokinase-2	-2.0	2.7E-02
GPM6A	neuronal membrane glycoprotein M6-a	-1.6	2.8E-02
SYT10	synaptotagmin X	-2.1	2.8E-02
CITED2	cbp/p300-interacting transactivator 2	-1.6	2.8E-02
KIF23	kinesin-like protein *KIF23*	-1.4	2.9E-02
PLK1	serine/threonine-protein kinase *PLK1*	-1.4	2.9E-02
LATS2	large tumor suppressor kinase 2	-1.5	2.9E-02
OLFML2B	olfactomedin-like 2B	-1.9	3.0E-02
FZD7	frizzled-7 precursor	-1.5	3.0E-02
MYOC	myocilin, trabecular meshwork inducible glucocorticoid response	-1.7	3.0E-02
RASGRP1	RAS guanyl releasing protein 1 (calcium and DAG-regulated)	-1.8	3.1E-02
EDNRB	endothelin receptor type B precursor	-1.5	3.2E-02
NDC80	Kinetochore protein *NDC80* homolog	-1.5	3.2E-02
PRSS55	protease, serine, 55	-1.9	3.2E-02
PASK	PAS domain containing serine/threonine kinase	-1.5	3.2E-02
ANKRD29	ankyrin repeat domain 29	-1.8	3.2E-02
DEPDC1B	DEP domain-containing protein 1B	-1.6	3.3E-02
INCENP	inner centromere protein	-1.5	3.3E-02
S100A11	Protein *S100-A11*	-2.3	3.3E-02
CLIC6	Uncharacterized protein	-2.2	3.4E-02
AMH	muellerian-inhibiting factor precursor	-2.2	3.4E-02
PCDH19	protocadherin-19 precursor	-1.9	3.4E-02
TIFA	TRAF-interacting protein with forkhead-associated domain	-2.1	3.5E-02
U6	*U6* spliceosomal RNA	/0	3.6E-02
SLCO1C1	solute carrier organic anion transporter family member 1C1	-2.0	3.6E-02
BOC	*BOC* cell adhesion associated, oncogene regulated	-1.5	3.6E-02
LAPTM4B	lysosomal protein transmembrane 4 beta	-1.5	3.7E-02
SERPINB10	Heterochromatin-associated protein MENT [	-2.1	3.7E-02
KCNQ4	potassium voltage-gated channel, KQT-like subfamily, member 4	-1.4	3.8E-02
F10	Coagulation factor X Factor X light chain Factor X heavy chain Activated factor Xa heavy chain	-1.7	3.8E-02
FOXI2	forkhead box I2	-1.7	3.8E-02
TERT	telomerase reverse transcriptase	-1.4	3.8E-02
RPIA	ribose-5-phosphate isomerase	-1.4	3.8E-02
RNASET2	ribonuclease T2 precursor	-1.5	3.8E-02
FN1	fibronectin precursor	-1.5	3.9E-02
CLDN5	claudin 5	-3.1	3.9E-02
STRA6	stimulated by retinoic acid 6	-2.0	4.0E-02

UTS2D	urotensin-2B precursor	-2.1	4.0E-02
EMCN	endomucin	-1.9	4.0E-02
PDE4C	phosphodiesterase 4C, cAMP-specific	-1.4	4.1E-02
ENSGALG00000002749	novel gene	-1.4	4.1E-02
RBP3	retinol-binding protein 3 precursor	-1.4	4.1E-02
ENTPD2	ectonucleoside triphosphate diphosphohydrolase 2 precursor	-1.5	4.1E-02
BARD1	BRCA1-associated RING domain protein 1	-1.5	4.1E-02
TMEM132D	transmembrane protein 132D	-1.5	4.1E-02
HGF/SF	hepatocyte growth factor precursor	-1.4	4.1E-02
OSBPL3	oxysterol binding protein-like 3	-1.4	4.1E-02
ENSGALG00000002955	novel gene	-1.7	4.1E-02
SLC39A8	solute carrier family 39 (zinc transporter), member 8	-2.2	4.1E-02
PDE9A	phosphodiesterase 9A	-2.2	4.1E-02
KIF4A	chromosome-associated kinesin KIF4	-1.4	4.2E-02
GPC4	glypican 4	-2.1	4.2E-02
ENSGALG00000020899	Uncharacterized protein	-2.3	4.2E-02
TSC22D3	TSC22 domain family, member 3	-1.5	4.3E-02
GPX7	glutathione peroxidase 7	-1.7	4.3E-02
EGLN3	egl nine homolog 3 (C. elegans)	-1.9	4.4E-02
MXD3	MAX dimerization protein 3	-1.9	4.4E-02
DISP1	dispatched homolog 1 (Drosophila)	-1.3	4.4E-02
CCNB3	G2/mitotic-specific cyclin-B3	-1.4	4.5E-02
PRX	paired mesoderm homeobox protein 1	-1.8	4.5E-02
ENSGALG00000005114	novel gene	-1.8	4.5E-02
DOCK11	dedicator of cytokinesis 11	-1.4	4.6E-02
BRCA1	breast cancer 1, early onset	-1.3	4.8E-02
MCM3	DNA replication licensing factor *MCM3*	-1.4	4.9E-02
snoZ196	Small nucleolar RNA Z196/R39/R59 family	/0	5.0E-02

*p-values are corrected for multiple testing by the false discovery rate method as utilized by cuffdiff (version 2.1.1).
