# Supplementary material for: Differentiation State-Specific Mitochondrial Dynamic Regulatory Networks Are Revealed by Global Transcriptional Analysis of the Developing Chicken Lens
Source: G3 (Bethesda). 2014 Jun 13;4(8):1515–27. doi: 10.1534/g3.114.012120 (PMC4132181; doi:10.1534/g3.114.012120)
Supplement: Supporting Information [file supp_g3.114.012120_TableS2.pdf]

Table S2 Detected EC gene-specific transcripts statistically decreased in expression during EC to EQ transition.

Gene	Description	log2(Fold Change)	p-value*
ANXA1	annexin A1	4.9	1.2E-03
CER1	cerberus 1, DAN family BMP antagonist	4.4	1.2E-03
BMPER	BMP-binding endothelial regulator protein precursor	4.2	1.2E-03
HRH3	histamine receptor H3	3.9	1.2E-03
STEAP1	six transmembrane epithelial antigen of the prostate 1	3.9	1.2E-03
GATA3	GATA-binding factor 3	3.5	1.2E-03
CRYBB3	Beta-crystallin B3	3.5	1.2E-03
GCHFR	GTP cyclohydrolase 1 feedback regulatory protein	3.5	1.2E-03
CILP	cartilage intermediate layer protein, nucleotide pyrophosphohydrolase	3.4	1.2E-03
GABRA3	gamma-aminobutyric acid (GABA) A receptor, alpha 3	3.3	1.2E-03
ARSH	arylsulfatase H precursor	3.3	1.2E-03
PAG1	phosphoprotein associated with glycosphingolipid microdomains 1	3.3	1.2E-03
HGD	homogentisate 1,2-dioxygenase	3.3	1.2E-03
CYP1B1	cytochrome P450, family 1, subfamily B, polypeptide 1	3.2	1.2E-03
KCNK1	potassium channel, subfamily K, member 1	3.2	1.2E-03
WNT7A	wingless-type MMTV integration site family, member 7A	3.2	1.2E-03
CYP1A4	cytochrome P450 1A4	3.2	1.2E-03
VIT	vitrin precursor	3.2	1.2E-03
RCAN2	regulator of calcineurin 2	3.1	1.2E-03
SCIN	Adseverin	3.1	1.2E-03
CCDC80	coiled-coil domain-containing protein 80 precursor	3.1	1.2E-03
FAM46A	uncharacterized protein LOC421845	3.1	1.2E-03
WNT7B	Protein *Wnt-7b*	3.1	1.2E-03
CDK14	cyclin-dependent kinase 14	3.0	1.2E-03
HEYL	hairy/enhancer-of-split related with YRPW motif-like	3.0	1.2E-03
DLG2	discs, large homolog 2 (Drosophila)	3.0	1.2E-03
MEGF10	multiple EGF-like-domains 10	3.0	1.2E-03
SLC12A3	solute carrier family 12 (sodium/chloride transporters), member 3	2.9	1.2E-03
GLUR1/A	glutamate receptor 1 precursor	2.9	1.2E-03
ADAMTSL3	ADAMTS-like 3	2.9	1.2E-03
RSPO3	R-spondin 3	2.9	1.2E-03
ENSGALG00000015653	junctophilin 1	2.9	1.2E-03
LONRF3	LON peptidase N-terminal domain and ring finger 3	2.9	1.2E-03
ID1	DNA-binding protein inhibitor *ID-1*	2.9	1.2E-03
RELN	reelin	2.9	1.2E-03
KIAA1107	*KIAA1107*	2.8	1.2E-03
JAG1	Delta-like protein	2.7	1.2E-03
FZD10	frizzled-10 precursor	2.7	1.2E-03
SEMA5A	sema domain, seven thrombospondin repeats (type 1 and type 1-like), transmembrane domain (TM) and short cytoplasmic domain, (semaphorin) 5A	2.7	1.2E-03
TAPP2	pleckstrin homology domain-containing family A member 2	2.7	1.2E-03
ENSGALG00000005344	Uncharacterized protein	2.7	1.2E-03
SLC35A5	solute carrier family 35, member A5	2.6	1.2E-03
PIK3CB	phosphatidylinositol-4,5-bisphosphate 3-kinase catalytic subunit beta isoform	2.6	1.2E-03
JAM2	junctional adhesion molecule B precursor	2.6	1.2E-03
ABI3BP	ABI family, member 3 (NESH) binding protein	2.5	1.2E-03
EDC3	enhancer of mRNA-decapping protein 3	2.5	1.2E-03
PIWIL1	Piwi-like protein 1	2.5	1.2E-03
PLCL1	phospholipase C-like 1	2.5	1.2E-03
RHOBTB3	Rho-related BTB domain containing 3	2.5	1.2E-03
AGL	amylo-alpha-1, 6-glucosidase, 4-alpha-glucanotransferase	2.5	1.2E-03
CPM	carboxypeptidase M precursor	2.4	1.2E-03
EPS8	epidermal growth factor receptor pathway substrate 8	2.3	1.2E-03

ETV4	ets variant 4	2.3	1.2E-03
NDRG1	N-myc downstream regulated 1	2.3	1.2E-03
ST6GAL2	beta-galactoside alpha-2,6-sialyltransferase 2	2.3	1.2E-03
ADCY8	adenylate cyclase 8 (brain)	2.3	1.2E-03
TRAK1	trafficking protein, kinesin binding 1	2.3	1.2E-03
TBCB	Tubulin-folding cofactor B**	2.2	1.2E-03
SLC26A5	prestin	2.2	1.2E-03
EFR3B	EFR3 homolog B (S. cerevisiae)	2.2	1.2E-03
KCNV1	potassium channel, subfamily V, member 1	2.2	1.2E-03
PRTG	protogenin precursor	2.2	1.2E-03
CRYGS	beta-crystallin S	2.2	1.2E-03
TAC1	Tachykinin 1	2.2	1.2E-03
MYO3B	myosin IIIB	2.2	1.2E-03
MUC5B	Mucin-5B	2.2	1.2E-03
FRAS1	Fraser syndrome 1	2.2	1.2E-03
POLH	polymerase (DNA directed), eta	2.1	1.2E-03
EGFR	Epidermal growth factor receptor	2.1	1.2E-03
PARP16	poly (ADP-ribose) polymerase family, member 16	2.1	1.2E-03
KIAA1644	Uncharacterized protein	2.1	1.2E-03
AGBL1	ATP/GTP binding protein-like 1	2.1	1.2E-03
NRIP1	nuclear receptor interacting protein 1	2.0	1.2E-03
LAMA5	laminin, alpha 5	2.0	1.2E-03
C7ORF63	Uncharacterized protein	2.0	1.2E-03
SLC16A5	solute carrier family 16, member 5 (monocarboxylic acid transporter 6)	2.0	1.2E-03
RSPO1	R-spondin 1	3.9	2.0E-03
FSHB	follitropin subunit beta precursor	2.9	2.0E-03
MOV10L1	*Mov10l1*, Moloney leukemia virus 10-like 1, homolog (mouse)	2.9	2.0E-03
ID2	DNA-binding protein inhibitor *ID-2*	2.5	2.0E-03
FSIP1	fibrous sheath interacting protein 1	2.4	2.0E-03
CAMK1G	calcium/calmodulin-dependent protein kinase IG	2.4	2.0E-03
GRK5	G protein-coupled receptor kinase 5	2.4	2.0E-03
KCTD12	Uncharacterized protein	2.2	2.0E-03
SPSB4	splA/ryanodine receptor domain and SOCS box containing 4	2.1	2.0E-03
HPCAL1	Hippocalcin-like protein 1	2.0	2.0E-03
ENSGALG00000005204	novel gene	2.7	2.8E-03
DNER	delta/notch-like EGF repeat containing	2.6	2.8E-03
CHST9	Uncharacterized protein	2.5	2.8E-03
OSBPL2	oxysterol-binding protein-related protein 2	2.4	2.8E-03
SCRN1	secernin 1	2.3	2.8E-03
C3ORF64	uncharacterized glycosyltransferase AER61	2.1	2.8E-03
S1PR1	sphingosine-1-phosphate receptor 1	2.0	2.8E-03
PRR5	proline-rich protein 5	2.0	2.8E-03
TRPC3	Uncharacterized protein	2.4	3.5E-03
HOMER3	homer homolog 3 (Drosophila)	2.1	3.5E-03
SPON2	spondin 2, extracellular matrix protein	2.0	3.5E-03
NDNF	neuron-derived neurotrophic factor	2.0	3.5E-03
ENSGALG00000027002	Uncharacterized protein	1.9	3.5E-03
MXRA5	matrix-remodelling associated 5	3.2	4.2E-03
ENSGALG00000022857	Uncharacterized protein	3.0	4.2E-03
FAM198B	family with sequence similarity 198, member B	3.0	4.2E-03
SCNN1B	sodium channel, non-voltage-gated 1, beta subunit	2.7	4.2E-03
PDGFRA	platelet-derived growth factor receptor alpha precursor	2.3	4.2E-03
THSD7A	thrombospondin, type I, domain containing 7A	2.2	4.2E-03
ENSGALG00000004078	novel gene	2.2	4.2E-03
CXCR7	chemokine (C-X-C motif) receptor 7	2.0	4.2E-03
ENSGALG00000004322	Uncharacterized protein	2.0	4.2E-03
KLHL36	kelch-like family member 36	1.8	4.2E-03
PTPRD	protein tyrosine phosphatase, receptor type, D	1.8	4.2E-03
GPAM	glycerol-3-phosphate acyltransferase, mitochondrial	1.8	4.2E-03
MRPL39	mitochondrial ribosomal protein L39	1.7	4.2E-03
EDG7	lysophosphatidic acid receptor 3	3.3	4.8E-03

EPHX4	epoxide hydrolase 4	2.6	4.8E-03
SEMA3G	sema domain, immunoglobulin domain (Ig), short basic domain, secreted, (semaphorin) 3G	2.5	4.8E-03
RGS6	regulator of G-protein signaling 6	2.0	4.8E-03
CDS1	Phosphatidate cytidylyltransferase	1.9	4.8E-03
PLCL2	phospholipase C-like 2	1.9	4.8E-03
STARD8	StAR-related lipid transfer (START) domain containing 8	4.2	5.4E-03
NRARP	NOTCH-regulated ankyrin repeat protein	3.2	5.4E-03
RBM24	RNA-binding protein 24	2.4	5.4E-03
NHS	Nance-Horan syndrome (congenital cataracts and dental anomalies)	2.2	5.4E-03
MYPN	myopalladin	2.1	5.4E-03
DCLK2	doublecortin-like kinase 2	1.9	5.4E-03
VTN	vitronectin precursor	1.8	5.4E-03
ENSGALG00000014164	Uncharacterized protein	1.8	5.4E-03
DGKB	diacylglycerol kinase, beta 90kDa	1.7	5.4E-03
DRAXIN	*Draxin*	3.0	6.0E-03
SARM1	sterile alpha and TIR motif containing 1	2.4	6.0E-03
KIF26B	kinesin family member 26B	2.0	6.0E-03
CRYBA4	beta-crystallin A4	2.0	6.0E-03
HES5	transcription factor *HES-5*	1.9	6.0E-03
DUSP16	dual specificity phosphatase 16	1.8	6.0E-03
CYP26A1	cytochrome P450 26A1	1.7	6.0E-03
PERP	*PERP*, TP53 apoptosis effector	2.7	6.5E-03
CRYGN	gamma-crystallin N	2.0	6.5E-03
MAP4K4	mitogen-activated protein kinase kinase kinase kinase 4	1.9	6.5E-03
OSBPL6	oxysterol binding protein-like 6	1.8	6.5E-03
TMEM56	transmembrane protein 56	1.6	6.5E-03
WNK2	WNK lysine deficient protein kinase 2	2.2	7.0E-03
HTR7	5-hydroxytryptamine (serotonin) receptor 7, adenylate cyclase-coupled	2.2	7.0E-03
SULF2	sulfatase 2	1.8	7.0E-03
CAPRIN2	caprin family member 2	1.8	7.0E-03
PLCXD1	PI-PLC X domain-containing protein 1	1.8	7.0E-03
CUX2	cut-like homeobox 2	1.7	7.0E-03
MYO3A	myosin IIIA	1.9	7.5E-03
TMCC1	transmembrane and coiled-coil domain family 1	1.7	7.5E-03
NTNG1	netrin G1	1.6	7.5E-03
ARSD	arylsulfatase D	2.7	8.0E-03
ELOVL7	elongation of very long chain fatty acids protein 7	2.1	8.0E-03
TBXAS1	Uncharacterized protein	2.5	8.5E-03
RUFY2	RUN and FYVE domain containing 2	2.2	8.5E-03
CARHSP1	Uncharacterized protein	1.8	8.5E-03
FABP3	fatty acid-binding protein, heart	3.4	9.0E-03
PID1	PTB-containing, cubilin and LRP1-interacting protein	2.0	9.0E-03
SAT1	Diamine acetyltransferase 1	1.7	9.0E-03
SMAD6	Mothers against decapentaplegic homolog 6	1.7	9.0E-03
gga-mir-1661	*gga-mir-1661* [Source:miRBase;Acc:MI0007395]	1.9	9.5E-03
CRCP	CGRP receptor component	1.9	9.5E-03
FREM1	FRAS1 related extracellular matrix 1	1.8	9.5E-03
COTL1	coactosin-like protein	2.8	1.0E-02
DKK3	dickkopf-related protein 3 precursor	2.6	1.0E-02
ATP2B2	ATPase, Ca++ transporting, plasma membrane 2	1.6	1.0E-02
COL27A1,ENSGALG00000025797	Uncharacterized protein	1.7	1.1E-02
TOX3	TOX high mobility group box family member 3	2.5	1.1E-02
PCNXL2	pecanex-like 2 (Drosophila)	1.8	1.1E-02
SCARB1	scavenger receptor class B, member 1	1.7	1.1E-02
TPGS2	tubulin polyglutamylase complex subunit 2	1.9	1.2E-02
RAPGEF1	Rap guanine nucleotide exchange factor (GEF) 1	1.8	1.2E-02
PARVB	parvin, beta	1.8	1.2E-02
THBS1	thrombospondin-1 precursor	2.6	1.2E-02
EPB41L2	erythrocyte membrane protein band 4.1-like 2	1.9	1.2E-02

PKDCC	protein kinase domain containing, cytoplasmic	1.8	1.2E-02
ENSGALG00000002150	novel gene	1.7	1.2E-02
EMILIN3	elastin microfibril interfacer 3	2.1	1.3E-02
ENSGALG00000026183	novel gene	1.7	1.3E-02
GAS2	growth arrest-specific protein 2	2.2	1.3E-02
GRAMD4	GRAM domain containing 4	1.8	1.3E-02
SESN1	sestrin 1	1.6	1.3E-02
ENSGALG00000027182	novel gene	1.6	1.3E-02
SASH1	SAM and SH3 domain containing 1	1.6	1.4E-02
ACTR3B	ARP3 actin-related protein 3 homolog B (yeast)	1.5	1.4E-02
PTGDS	prostaglandin D2 synthase, brain precursor	1.8	1.5E-02
VCPIP1	deubiquitinating protein VCIP135	1.7	1.5E-02
CCDC108	coiled-coil domain containing 108	1.6	1.5E-02
SLC4A7	solute carrier family 4, sodium bicarbonate cotransporter, member 7	1.6	1.5E-02
ENSGALG00000006407	death domain-containing tumor necrosis factor receptor superfamily member 23 precursor	2.5	1.5E-02
ENSGALG00000003644	novel gene	2.0	1.5E-02
NCKAP5	NCK-associated protein 5	1.9	1.5E-02
SHC2	SHC (Src homology 2 domain containing) transforming protein 2	1.7	1.5E-02
UGCG	UDP-glucose ceramide glucosyltransferase	1.6	1.5E-02
FBP1	fructose-1,6-bisphosphatase 1	2.1	1.6E-02
CRYBB1	Beta-crystallin B1	2.1	1.6E-02
BEND4	BEN domain containing 4	1.9	1.6E-02
UBE2Q2	ubiquitin-conjugating enzyme E2Q family member 2	1.9	1.6E-02
GYPC	glycophorin C (Gerbich blood group)	1.7	1.6E-02
ENSGALG00000005895	Uncharacterized protein	1.6	1.6E-02
MATN2	matrilin 2	1.9	1.6E-02
PPIC	peptidylprolyl isomerase C (cyclophilin C)	1.5	1.6E-02
DHX32	DEAH (Asp-Glu-Ala-His) box polypeptide 32	3.4	1.7E-02
FGFR2	Fibroblast growth factor receptor 2	1.9	1.7E-02
SESN3	sestrin 3	1.9	1.7E-02
RCBTB1	regulator of chromosome condensation (RCC1) and BTB (POZ) domain containing protein 1	1.7	1.7E-02
AGTPBP1	Cytosolic carboxypeptidase 1	1.6	1.7E-02
CDC14A	CDC14 cell division cycle 14 homolog A	1.6	1.7E-02
LHFP	Uncharacterized protein	1.6	1.7E-02
FXYD6	FXYD domain-containing ion transport regulator 6 precursor	1.5	1.7E-02
GPR137C	G protein-coupled receptor 137C	1.5	1.7E-02
TAGLN	Transgelin	1.7	1.8E-02
BCKDHB	2-oxoisovalerate dehydrogenase subunit beta, mitochondrial precursor	1.6	1.8E-02
SRP14	signal recognition particle 14 kDa protein	1.6	1.8E-02
JUN	transcription factor AP-1	1.5	1.9E-02
SMAD9	mothers against decapentaplegic homolog 9	2.6	1.9E-02
ZP4	zona pellucida sperm-binding protein 4	2.0	1.9E-02
NAV2	neuron navigator 2	1.8	1.9E-02
PROX2	prospero homeobox 2	2.1	1.9E-02
SYBU	syntabulin (syntaxin-interacting)	1.8	1.9E-02
CCNA1	cyclin A1	2.3	2.0E-02
KCNIP2	Kv channel-interacting protein 2	1.9	2.0E-02
NDRG4	NDRG family member 4	1.8	2.0E-02
PKP4	plakophilin-4	1.8	2.0E-02
PMP22	peripheral myelin protein 22	1.6	2.0E-02
GAMT	guanidinoacetate N-methyltransferase	1.5	2.0E-02
ENTPD3	ectonucleoside triphosphate diphosphohydrolase 3	2.2	2.0E-02
SH3RF3	SH3 domain containing ring finger 3	1.7	2.1E-02
CSK	tyrosine-protein kinase *CSK*	1.7	2.2E-02
GPRIN2	GRIN2-like protein	1.5	2.2E-02
SLC44A3	solute carrier family 44, member 3	1.4	2.2E-02
ENSGALG00000004279	lectin, galactoside-binding, soluble, 12	1.9	2.2E-02
STEAP2	STEAP family member 2, metalloreductase	1.9	2.2E-02

LMNA	lamin A/C	1.5	2.2E-02
FAM110B	family with sequence similarity 110, member B	1.7	2.3E-02
RPS6KL1	ribosomal protein S6 kinase-like 1	1.7	2.3E-02
TNNC1	Troponin C, slow skeletal and cardiac muscles	1.8	2.3E-02
MAP1B	microtubule-associated protein 1B	1.6	2.3E-02
SLC24A2	sodium/potassium/calcium exchanger 2	1.5	2.4E-02
MBOAT1	membrane bound O-acyltransferase domain containing 1	1.8	2.4E-02
TPH2	tryptophan 5-hydroxylase 2	1.6	2.4E-02
CAP2	Adenylyl cyclase-associated protein	1.6	2.4E-02
PLA2G10	phospholipase A2, group X	1.5	2.4E-02
GSTT1	Glutathione S-transferase theta-1	2.2	2.5E-02
DMN	synemin	1.8	2.5E-02
ENSGALG00000010722	Schwann cell-specific EGF-like repeat autocrine factor precursor	1.7	2.5E-02
CPEB4	cytoplasmic polyadenylation element binding protein 4	1.7	2.6E-02
TBC1D1	TBC1 (tre-2/USP6, BUB2, cdc16) domain family, member 1	1.7	2.6E-02
ZNF704	zinc finger protein 704	1.4	2.7E-02
NACAD	NAC alpha domain containing	1.9	2.7E-02
LURAP1	leucine rich adaptor protein 1	2.7	2.7E-02
MICALL2	MICAL-like 2	2.5	2.7E-02
SLC38A2	sodium-coupled neutral amino acid transporter 2	2.2	2.7E-02
AGPAT4	1-acylglycerol-3-phosphate O-acyltransferase 4	1.7	2.8E-02
NRCAM	neuronal cell adhesion molecule	1.4	2.8E-02
STARD5	StAR-related lipid transfer (START) domain containing 5	1.7	2.9E-02
FAM102A	early estrogen-induced gene 1 protein	1.7	2.9E-02
GLUL	glutamine synthetase	1.4	3.0E-02
HECW1	HECT, C2 and WW domain containing E3 ubiquitin protein ligase 1	1.4	3.0E-02
PABPC4	poly(A) binding protein, cytoplasmic 4 (inducible form)	1.4	3.0E-02
ARHGAP39	Rho GTPase activating protein 39	1.6	3.1E-02
PNPLA2	patatin-like phospholipase domain-containing protein 2	1.5	3.1E-02
PLTP	phospholipid transfer protein precursor	1.5	3.1E-02
NPY	Pro-neuropeptide Y Neuropeptide Y C-flanking peptide of *NPY*	3.9	3.1E-02
ARHGAP22	Rho GTPase activating protein 22	2.4	3.2E-02
TEF	transcription factor VBP	2.4	3.2E-02
SSFA2	sperm specific antigen 2	1.4	3.2E-02
LRRC1	leucine rich repeat containing 1	1.5	3.2E-02
MAP7	ensconsin	1.4	3.2E-02
AMER3	APC membrane recruitment protein 3	2.8	3.2E-02
EPHA6	EPH receptor A6	1.7	3.2E-02
NFKBIE	nuclear factor of kappa light polypeptide gene enhancer in B-cells inhibitor, epsilon	1.5	3.3E-02
SNORD37	Small nucleolar RNA *SNORD37*	1.4	3.3E-02
MID2	Midline 2; Uncharacterized protein	2.1	3.4E-02
ASS1	argininosuccinate synthase	2.2	3.4E-02
MAEA	Macrophage erythroblast attacher	1.6	3.4E-02
TP53INP1	tumor protein p53-inducible nuclear protein 1	1.4	3.4E-02
METTL7A	methyltransferase like 7A	2.0	3.4E-02
FAM124A	family with sequence similarity 124A	1.9	3.4E-02
CD99	*CD99* antigen precursor	1.8	3.4E-02
CYP26C1	cytochrome P450, family 26, subfamily C, polypeptide 1	1.5	3.5E-02
ENSGALG00000026539	novel gene	1.4	3.5E-02
FHOD3	formin homology 2 domain containing 3	1.5	3.5E-02
SEMA6A	sema domain, transmembrane domain (TM), and cytoplasmic domain, (semaphorin) 6A	1.5	3.5E-02
LRRC48	leucine rich repeat containing 48	1.7	3.5E-02
ENSGALG00000017332	novel gene	2.0	3.6E-02
NFIL3	nuclear factor interleukin-3-regulated protein	1.4	3.6E-02
SEMA4G	sema domain, immunoglobulin domain (Ig), transmembrane domain (TM) and short cytoplasmic domain, (semaphorin) 4G	1.4	3.6E-02
RBM38	RNA-binding protein 38	1.4	3.6E-02

TTC7B	tetratricopeptide repeat protein 7B	1.4	3.6E-02
OSGIN2	oxidative stress induced growth inhibitor family member 2	1.5	3.7E-02
OTUD7A	OTU domain containing 7A	1.4	3.7E-02
SGK3	serine/threonine-protein kinase Sgk3	1.4	3.7E-02
CNN3	calponin 3, acidic	1.7	3.8E-02
PHKA2	phosphorylase kinase, alpha 2 (liver)	1.5	3.8E-02
APAF1	apoptotic peptidase activating factor 1	1.4	3.8E-02
ARHGAP24	Rho GTPase activating protein 24	1.5	3.8E-02
SORCS1	sortilin-related VPS10 domain containing receptor 1	1.4	3.8E-02
DAAM2	dishevelled associated activator of morphogenesis 2	1.6	3.9E-02
MSANTD3	Myb/SANT-like DNA-binding domain containing 3	1.4	3.9E-02
PIK3CA	phosphatidylinositol-4,5-bisphosphate 3-kinase catalytic subunit alpha isoform	1.3	3.9E-02
TUBB6	Tubulin beta-5 chain	1.4	3.9E-02
ENSGALG00000021838	novel gene	1.6	4.0E-02
ARHGAP10	Rho GTPase activating protein 10	1.4	4.1E-02
CAB39L	calcium binding protein 39-like	1.9	4.1E-02
FSD1L	fibronectin type III and SPRY domain containing 1-like	1.5	4.2E-02
TRIB1	tribbles homolog 1 (Drosophila)	1.5	4.2E-02
KCTD1	potassium channel tetramerization domain containing 1	1.5	4.2E-02
RASSF2	ras association domain-containing protein 2	1.5	4.2E-02
GAS7	growth arrest-specific 7	1.5	4.3E-02
SERPINB6	serpin B6	1.4	4.4E-02
CABP7	calcium binding protein 7	1.6	4.5E-02
EMP2	epithelial membrane protein 2	1.3	4.5E-02
ENSGALG00000004676	Uncharacterized protein	1.4	4.5E-02
RNaseP_nuc	Nuclear RNase P	1.9	4.6E-02
KIF25	kinesin family member 25	1.5	4.6E-02
ACAP3	ArfGAP with coiled-coil, ankyrin repeat and PH domains 3	1.4	4.7E-02
EDN3	Endothelin 3	1.6	4.8E-02
DUSP8	dual specificity phosphatase 8	1.7	4.8E-02
gga-let-7b	*gga-let-7b* [Source:miRBase;Acc:MI0001172]	1.5	4.9E-02
ENSGALG00000028383	novel gene	1.7	5.0E-02
TMEM135	transmembrane protein 135	1.3	5.0E-02

*p-values are corrected for multiple testing by the false discovery rate method as utilized by cuffdiff (version 2.1.1).
