# Supplementary material for: Differentiation State-Specific Mitochondrial Dynamic Regulatory Networks Are Revealed by Global Transcriptional Analysis of the Developing Chicken Lens
Source: G3 (Bethesda). 2014 Jun 13;4(8):1515–27. doi: 10.1534/g3.114.012120 (PMC4132181; doi:10.1534/g3.114.012120)
Supplement: Supporting Information [file supp_g3.114.012120_TableS3.pdf]

Table S3   Detected EQ gene-specific transcripts statistically decreased in expression during EQ to FP transition.

Gene	Description	log2(Fold Change)	p-value*
CC2D2A	coiled-coil and C2 domain containing 2A	-2.0	1.2E-03
INSIG2	insulin induced gene 2	-2.0	1.2E-03
ODZ2	teneurin transmembrane protein 2	-2.0	1.2E-03
SEPHS1	selenophosphate synthetase 1	-2.0	1.2E-03
B4GALT6	UDP-Gal:betaGlcNAc beta 1,4- galactosyltransferase, polypeptide 6	-2.0	1.2E-03
CDC42SE2	CDC42 small effector 2	-2.0	1.2E-03
SLIT3	slit homolog 3 (Drosophila)	-2.1	1.2E-03
FKBP9	FK506 binding protein 9, 63 kDa	-2.1	1.2E-03
ATAD2	ATPase family, AAA domain containing 2	-2.1	1.2E-03
PURH	5-aminoimidazole-4-carboxamide ribonucleotide formyltransferase/IMP cyclohydrolase	-2.1	1.2E-03
PLXNA2	plexin A2	-2.1	1.2E-03
CSRNP1	cysteine-serine-rich nuclear protein 1	-2.1	1.2E-03
PER2	period circadian clock 2	-2.1	1.2E-03
CERK	ceramide kinase	-2.1	1.2E-03
NRSN1	neurensin 1	-2.1	1.2E-03
C1H21orf33	ES1 protein homolog, mitochondrial	-2.1	1.2E-03
REPS2	RALBP1 associated Eps domain containing 2	-2.2	1.2E-03
TPX2	*TPX2*, microtubule-associated, homolog (Xenopus laevis)	-2.2	1.2E-03
PPIC	peptidylprolyl isomerase C (cyclophilin C)	-2.2	1.2E-03
GNG10	guanine nucleotide binding protein (G protein), gamma 10	-2.2	1.2E-03
PHF16	PHD finger protein 16	-2.2	1.2E-03
TMEM108	transmembrane protein 108	-2.2	1.2E-03
MCAM	melanoma cell adhesion molecule	-2.2	1.2E-03
TLL1	tolloid-like 1	-2.2	1.2E-03
TMEM194B	transmembrane protein 194B	-2.2	1.2E-03
PIWIL1	piwi-like RNA-mediated gene silencing 1	-2.2	1.2E-03
SORCS1	sortilin-related VPS10 domain containing receptor 1	-2.2	1.2E-03
MKI67	antigen identified by monoclonal antibody Ki-67	-2.2	1.2E-03
C1H12ORF23	UPF0444 transmembrane protein C12orf23 homolog	-2.2	1.2E-03
CCDC108	coiled-coil domain containing 108	-2.2	1.2E-03
RYK	receptor-like tyrosine kinase	-2.2	1.2E-03
CMTM3	CKLF-like MARVEL transmembrane domain containing 3	-2.2	1.2E-03
LACTB2	lactamase, beta 2	-2.2	1.2E-03
KPNA2	karyopherin alpha 2 (RAG cohort 1, importin alpha 1)	-2.2	1.2E-03
ODZ3	teneurin-3	-2.2	1.2E-03
NDST2	N-deacetylase/N-sulfotransferase (heparan glucosaminyl) 2	-2.2	1.2E-03
TRAF4	TNF receptor-associated factor 4	-2.2	1.2E-03
FGF19	fibroblast growth factor 19	-2.2	1.2E-03
PLXNC1	plexin C1	-2.2	1.2E-03
CENPF	centromere protein F, 350/400kDa	-2.2	1.2E-03
ANKH	*ANKH* inorganic pyrophosphate transport regulator	-2.3	1.2E-03
SRP14	signal recognition particle 14kDa (homologous Alu RNA binding protein)	-2.3	1.2E-03
CHST14	carbohydrate (N-acetylgalactosamine 4-0) sulfotransferase 14	-2.3	1.2E-03
NBEAL2	neurobeachin-like 2	-2.3	1.2E-03
LAMA4	laminin, alpha 4	-2.3	1.2E-03
CABLES1	Cdk5 and Abl enzyme substrate 1	-2.3	1.2E-03
CBS	cystathionine-beta-synthase	-2.3	1.2E-03
OLFM3	olfactomedin 3	-2.3	1.2E-03
PAM	peptidylglycine alpha-amidating monooxygenase	-2.3	1.2E-03
P4HA3	prolyl 4-hydroxylase, alpha polypeptide III	-2.3	1.2E-03
HNRNPA0	heterogeneous nuclear ribonucleoprotein A0	-2.3	1.2E-03
BTG2	BTG family, member 2	-2.3	1.2E-03
SOBP	sine oculis binding protein homolog (Drosophila)	-2.3	1.2E-03
CCDC88C	coiled-coil domain containing 88C	-2.3	1.2E-03

SEC61A1	Sec61 alpha 1 subunit (S. cerevisiae)	-2.3	1.2E-03
METRNL	meteorin, glial cell differentiation regulator-like	-2.3	1.2E-03
SPINT1	serine peptidase inhibitor, Kunitz type 1	-2.3	1.2E-03
ROR1	receptor tyrosine kinase-like orphan receptor 1	-2.3	1.2E-03
GCHFR	GTP cyclohydrolase I feedback regulator	-2.3	1.2E-03
GM2A	GM2 ganglioside activator	-2.3	1.2E-03
SH3D19	SH3 domain containing 19	-2.3	1.2E-03
PRKX	protein kinase, X-linked	-2.4	1.2E-03
RND3	Rho family GTPase 3	-2.4	1.2E-03
TJP2	tight junction protein 2	-2.4	1.2E-03
ATP2B4	ATPase, Ca++ transporting, plasma membrane 4	-2.4	1.2E-03
NAB1	NGFI-A binding protein 1 (EGR1 binding protein 1)	-2.4	1.2E-03
TNS3	tensin 3	-2.4	1.2E-03
CCDC109B	coiled-coil domain containing 109B	-2.4	1.2E-03
TCTN3	tectonic family member 3	-2.4	1.2E-03
ZNF536	zinc finger protein 536	-2.4	1.2E-03
HKDC1	hexokinase domain containing 1	-2.4	1.2E-03
ANKRD50	ankyrin repeat domain 50	-2.4	1.2E-03
TMEM16E	anoctamin 5	-2.4	1.2E-03
SLC29A1	solute carrier family 29 (equilibrative nucleoside transporter), member 1	-2.4	1.2E-03
CASKIN2	CASK interacting protein 2	-2.4	1.2E-03
EXOG	endo/exonuclease (5,-3,), endonuclease G-like	-2.4	1.2E-03
PLOD2	procollagen-lysine, 2-oxoglutarate 5-dioxygenase 2	-2.4	1.2E-03
EGFR	epidermal growth factor receptor	-2.4	1.2E-03
C16ORF45	chromosome 16 open reading frame 45	-2.4	1.2E-03
DLG2	discs, large homolog 2 (Drosophila)	-2.4	1.2E-03
RRM2	ribonucleotide reductase M2	-2.5	1.2E-03
GPRIN2	G protein regulated inducer of neurite outgrowth 2	-2.5	1.2E-03
FAM69A	family with sequence similarity 69, member A	-2.5	1.2E-03
CTH	cystathionase (cystathionine gamma-lyase)	-2.5	1.2E-03
GLUR1/A	glutamate receptor 1 precursor	-2.5	1.2E-03
SHISA2	shisa homolog 2 (Xenopus laevis)	-2.5	1.2E-03
PLA2G10	phospholipase A2, group X	-2.5	1.2E-03
RBL1	retinoblastoma-like 1 (p107)	-2.5	1.2E-03
DUT	deoxyuridine 5,-triphosphate nucleotidohydrolase, mitochondrial	-2.5	1.2E-03
TP53INP1	tumor protein p53 inducible nuclear protein 1	-2.5	1.2E-03
FNDC3B	fibronectin type III domain containing 3B	-2.5	1.2E-03
CKAP2	cytoskeleton associated protein 2	-2.5	1.2E-03
ITPKA	inositol-trisphosphate 3-kinase A	-2.5	1.2E-03
GALNTL1	Uncharacterized protein	-2.5	1.2E-03
HNRPK	heterogeneous nuclear ribonucleoprotein K	-2.5	1.2E-03
EMILIN2	elastin microfibril interfacer 2	-2.5	1.2E-03
FAM43A	family with sequence similarity 43, member A	-2.5	1.2E-03
GCH1	GTP cyclohydrolase 1	-2.5	1.2E-03
GRM4	glutamate receptor, metabotropic 4	-2.5	1.2E-03
FAM171A1	family with sequence similarity 171, member A1	-2.5	1.2E-03
CASZ1	castor zinc finger 1	-2.5	1.2E-03
SCNN1A	amiloride-sensitive sodium channel subunit alpha	-2.5	1.2E-03
SMC2	structural maintenance of chromosomes protein 2	-2.6	1.2E-03
ENSGALG00000009516	transforming, acidic coiled-coil containing protein 2	-2.6	1.2E-03
FRMD4B	FERM domain containing 4B	-2.6	1.2E-03
SLC38A5	solute carrier family 38, member 5	-2.6	1.2E-03
EPB41L5	erythrocyte membrane protein band 4.1 like 5	-2.6	1.2E-03
MGST1	microsomal glutathione S-transferase 1	-2.6	1.2E-03
VPS37B	vacuolar protein sorting 37 homolog B (S. cerevisiae)	-2.6	1.2E-03
LAMA3	laminin, alpha 3	-2.6	1.2E-03
C2ORF40	Uncharacterized protein	-2.6	1.2E-03
KERA	Keratocan	-2.6	1.2E-03
PRR5	proline-rich protein 5	-2.6	1.2E-03
MID1	midline 1 (Opitz/BBB syndrome)	-2.6	1.2E-03
UGDH	UDP-glucose 6-dehydrogenase	-2.6	1.2E-03

PLVAP	plasmalemma vesicle associated protein	-2.6	1.2E-03
ENSGALG00000005727	solute carrier family 7 (cationic amino acid transporter, y+ system), member 3	-2.6	1.2E-03
ENSGALG00000028602	novel gene	-2.6	1.2E-03
DLGAP5	discs, large (Drosophila) homolog-associated protein 5	-2.6	1.2E-03
HES4	transcription factor HES-1 isoform 2	-2.6	1.2E-03
CXCR7	chemokine (C-X-C motif) receptor 7	-2.6	1.2E-03
RBP5	retinol binding protein 5, cellular	-2.6	1.2E-03
APAF1	apoptotic peptidase activating factor 1	-2.6	1.2E-03
UAP1L1	UDP-N-acteylglucosamine pyrophosphorylase 1-like 1	-2.6	1.2E-03
COL4A1	collagen alpha-1(IV) chain precursor	-2.6	1.2E-03
PLCL2	phospholipase C-like 2	-2.6	1.2E-03
PLCB4	1-phosphatidylinositol-4,5-bisphosphate phosphodiesterase beta-4	-2.7	1.2E-03
ADAMTS17	ADAM metallopeptidase with thrombospondin type 1 motif, 17	-2.7	1.2E-03
MYRF	myelin regulatory factor	-2.7	1.2E-03
ABCC9	ATP-binding cassette, sub-family C (CFTR/MRP), member 9	-2.7	1.2E-03
PLD1	phospholipase D1, phosphatidylcholine-specific	-2.7	1.2E-03
CNFI-A4	nuclear factor 1 A-type	-2.7	1.2E-03
RLBP1	retinaldehyde-binding protein 1	-2.7	1.2E-03
HMCN1	hemicentin 1	-2.7	1.2E-03
KAT6B	K(lysine) acetyltransferase 6B	-2.7	1.2E-03
ATP2B1	plasma membrane calcium-transporting ATPase 1	-2.7	1.2E-03
ENSGALG00000016322	glutathione S-transferase 3	-2.7	1.2E-03
PHLDB2	pleckstrin homology-like domain, family B, member 2	-2.7	1.2E-03
TENM4	teneurin transmembrane protein 4	-2.7	1.2E-03
LEF-1	lymphoid enhancer-binding factor *1*	-2.7	1.2E-03
SLC12A2	solute carrier family 12	-2.7	1.2E-03
ASTN1	astrotactin 1	-2.7	1.2E-03
RERG	RAS-like, estrogen-regulated, growth inhibitor	-2.7	1.2E-03
COL27A1,ENSGALG00000025797	Uncharacterized protein	-2.7	1.2E-03
MXRA8	Matrix-remodeling-associated protein 8	-2.7	1.2E-03
SCARB2	scavenger receptor class B, member 2	-2.7	1.2E-03
CDC2	cyclin-dependent kinase 1	-2.8	1.2E-03
RELL1	RELT-like protein 1	-2.8	1.2E-03
FOXP1	Forkhead box protein P1	-2.8	1.2E-03
ANXA8	Annexin	-2.8	1.2E-03
41528		-2.8	1.2E-03
PDK1	pyruvate dehydrogenase kinase, isozyme 1	-2.8	1.2E-03
HMGB2	high mobility group protein B2	-2.8	1.2E-03
LIPG	lipase, endothelial	-2.8	1.2E-03
SLC38A1	sodium-coupled neutral amino acid transporter 1	-2.8	1.2E-03
TNC	tenascin precursor	-2.8	1.2E-03
GXYLT2	glucoside xylosyltransferase 2	-2.8	1.2E-03
SLC38A11	solute carrier family 38, member 11	-2.8	1.2E-03
NMI	N-myc (and STAT) interactor	-2.8	1.2E-03
SLC16A12	solute carrier family 16, member 12 (monocarboxylic acid transporter 12)	-2.8	1.2E-03
NKD1	naked cuticle homolog 1 (Drosophila)	-2.8	1.2E-03
FGFR2	Fibroblast growth factor receptor 2	-2.8	1.2E-03
ISLR	immunoglobulin superfamily containing leucine-rich repeat	-2.8	1.2E-03
MAML2	mastermind-like 2 (Drosophila)	-2.8	1.2E-03
AADAC	arylacetamide deacetylase	-2.8	1.2E-03
RGMA	RGM domain family, member A	-2.8	1.2E-03
KIAA1644	*KIAA1644*	-2.8	1.2E-03
ABI3BP	ABI family, member 3 (NESH) binding protein	-2.9	1.2E-03
CITED3	Cbp/p300-interacting transactivator 3	-2.9	1.2E-03
C1orf198	chromosome 1 open reading frame 198	-2.9	1.2E-03
ARL4A	ADP-ribosylation factor-like 4A	-2.9	1.2E-03
PRPS2	phosphoribosyl pyrophosphate synthetase 2	-2.9	1.2E-03
NCKAP5	NCK-associated protein 5	-2.9	1.2E-03

TMEM200C	transmembrane protein 200C	-2.9	1.2E-03
ENSGALG00000000489	nascent polypeptide-associated complex alpha subunit 2	-2.9	1.2E-03
OPTC	opticin precursor	-2.9	1.2E-03
SEMA3C	semaphorin-3C precursor	-2.9	1.2E-03
SARDH	sarcosine dehydrogenase	-2.9	1.2E-03
CDK14	cyclin-dependent kinase 14	-2.9	1.2E-03
CPZ	carboxypeptidase Z	-2.9	1.2E-03
NFIB	nuclear factor I/B	-2.9	1.2E-03
MUC5B	mucin 5B, oligomeric mucus/gel-forming	-2.9	1.2E-03
FRMPD4	FERM and PDZ domain containing 4	-2.9	1.2E-03
PALLD	palladin, cytoskeletal associated protein	-3.0	1.2E-03
FAM207A	family with sequence similarity 207, member A	-3.0	1.2E-03
CD109	*CD109* molecule	-3.0	1.2E-03
MOV10L1	*Mov10l1*, Moloney leukemia virus 10-like 1, homolog (mouse)	-3.0	1.2E-03
FHL3	four and a half LIM domains 3	-3.0	1.2E-03
PDGFRL	platelet-derived growth factor receptor-like	-3.0	1.2E-03
NOTCH2	*notch 2*	-3.0	1.2E-03
SLC16A1	solute carrier family 16, member 1 (monocarboxylic acid transporter 1)	-3.0	1.2E-03
LTBP1	latent transforming growth factor beta binding protein 1	-3.0	1.2E-03
GABRB4	Gamma-aminobutyric acid receptor subunit beta-4	-3.0	1.2E-03
PLXNB2	plexin B2	-3.1	1.2E-03
PPP1R14C	protein phosphatase 1, regulatory (inhibitor) subunit 14C	-3.1	1.2E-03
C1QTNF6	C1q and tumor necrosis factor related protein 6	-3.1	1.2E-03
CSRP2	Cysteine and glycine-rich protein 2	-3.1	1.2E-03
CD81	*CD81* antigen	-3.1	1.2E-03
ADAMTS13	ADAM metallopeptidase with thrombospondin type 1 motif, 13	-3.1	1.2E-03
SOHO-1	sensory organ homeobox protein *SOHo*	-3.1	1.2E-03
TBC1D1	TBC1 (tre-2/USP6, BUB2, cdc16) domain family, member 1	-3.1	1.2E-03
ALPL	alkaline phosphatase, liver/bone/kidney	-3.1	1.2E-03
ANGPTL4	angiopoietin-like 4	-3.1	1.2E-03
TRPC3	transient receptor potential cation channel, subfamily C, member 3	-3.1	1.2E-03
ENSGALG00000027183	novel gene	-3.1	1.2E-03
SEC24D	SEC24 family, member D (S. cerevisiae)	-3.1	1.2E-03
EYA1	Eyes absent homolog 1	-3.1	1.2E-03
FREM1	FRAS1 related extracellular matrix 1	-3.1	1.2E-03
C7ORF63	Uncharacterized protein	-3.1	1.2E-03
ID2	DNA-binding protein inhibitor *ID-2*	-3.2	1.2E-03
SLC2A12	solute carrier family 2 (facilitated glucose transporter), member 12	-3.2	1.2E-03
OLFML3	Olfactomedin-like protein 3	-3.2	1.2E-03
CYP26A1	cytochrome P450 26A1	-3.2	1.2E-03
PLCXD1	PI-PLC X domain-containing protein 1	-3.2	1.2E-03
PSAT1	phosphoserine aminotransferase 1	-3.2	1.2E-03
WEE1	*wee1*-like protein kinase	-3.2	1.2E-03
NFKBIE	nuclear factor of kappa light polypeptide gene enhancer in B-cells inhibitor, epsilon	-3.2	1.2E-03
gga-let-7b	*gga-let-7b* [Source:miRBase;Acc:MI0001172]	-3.2	1.2E-03
ENPP6	ectonucleotide pyrophosphatase/phosphodiesterase 6	-3.2	1.2E-03
FAM110B	family with sequence similarity 110, member B	-3.3	1.2E-03
APOD	apolipoprotein D precursor	-3.3	1.2E-03
COL11A1	collagen, type XI, alpha 1	-3.3	1.2E-03
ENSGALG00000005344	Uncharacterized protein	-3.3	1.2E-03
PDGFRA	platelet-derived growth factor receptor alpha precursor	-3.3	1.2E-03
QPCT	glutaminyl-peptide cyclotransferase	-3.3	1.2E-03
SLC26A7	solute carrier family 26, member 7	-3.3	1.2E-03
ENSGALG00000028153	forkhead box E1 (thyroid transcription factor 2)	-3.3	1.2E-03
LRP1B	low density lipoprotein receptor-related protein 1	-3.3	1.2E-03
MAOA	monoamine oxidase A	-3.4	1.2E-03
GLUL	glutamine synthetase	-3.4	1.2E-03

SLC12A3	solute carrier family 12 (sodium/chloride transporters), member 3	-3.4	1.2E-03
DEGS1	Sphingolipid delta(4)-desaturase DES1	-3.4	1.2E-03
ADAMTSL3	ADAMTS-like 3	-3.4	1.2E-03
TDH	L-threonine dehydrogenase	-3.4	1.2E-03
PID1	PTB-containing, cubilin and LRP1-interacting protein	-3.4	1.2E-03
PCBP3	poly(rC) binding protein 3	-3.4	1.2E-03
TAC1	Tachykinin 1	-3.4	1.2E-03
LGALS3	Galectin-3	-3.4	1.2E-03
CLVS2	clavesin 2	-3.4	1.2E-03
TSKU	tsukushin precursor	-3.4	1.2E-03
CNTFR	Ciliary neurotrophic factor receptor subunit alpha	-3.4	1.2E-03
SLC6A6	sodium- and chloride-dependent taurine transporter	-3.4	1.2E-03
GRIN3A	glutamate receptor, ionotropic, N-methyl-D-aspartate 3A	-3.5	1.2E-03
SLC38A2	sodium-coupled neutral amino acid transporter 2	-3.5	1.2E-03
SOUL	*SOUL* protein	-3.5	1.2E-03
FSHB	follitropin subunit beta precursor	-3.6	1.2E-03
ABCA4	ATP-binding cassette, sub-family A (ABC1), member 4	-3.6	1.2E-03
COL24A1	collagen, type XXIV, alpha 1	-3.6	1.2E-03
RELN	reelin	-3.7	1.2E-03
SLC7A2	low affinity cationic amino acid transporter 2	-3.7	1.2E-03
ENSGALG00000027002	Uncharacterized protein	-3.7	1.2E-03
HEY1	hairy/enhancer-of-split related with YRPW motif 1	-3.7	1.2E-03
7SK	*7SK* RNA	-3.8	1.2E-03
NDNF	neuron-derived neurotrophic factor	-3.8	1.2E-03
KCNV1	potassium channel, subfamily V, member 1	-3.8	1.2E-03
NET1	neuroepithelial cell transforming 1	-3.9	1.2E-03
KCTD12	potassium channel tetramerization domain containing 12	-3.9	1.2E-03
ENSGALG00000004322	Uncharacterized protein	-3.9	1.2E-03
ENSGALG00000001136	Uncharacterized protein	-3.9	1.2E-03
STEAP1	six transmembrane epithelial antigen of the prostate 1	-4.0	1.2E-03
HEYL	hairy/enhancer-of-split related with YRPW motif-like	-4.0	1.2E-03
FAM198B	family with sequence similarity 198, member B	-4.2	1.2E-03
HGD	homogentisate 1,2-dioxygenase	-4.3	1.2E-03
HES5	transcription factor *HES-5*	-4.3	1.2E-03
CYP1B1	cytochrome P450, family 1, subfamily B, polypeptide 1	-4.3	1.2E-03
STARD8	StAR-related lipid transfer (START) domain containing 8	-5.4	1.2E-03
MIS12	*MIS12* kinetochore complex component	/0	1.2E-03
ADAMTS6	ADAM metallopeptidase with thrombospondin type 1 motif, 6	-1.8	2.0E-03
PGAP1	post-GPI attachment to proteins 1	-1.9	2.0E-03
TMX4	thioredoxin-related transmembrane protein 4 precursor	-2.0	2.0E-03
CDH11	cadherin-11 precursor	-2.0	2.0E-03
PLTP	phospholipid transfer protein precursor	-2.0	2.0E-03
JAZF1	juxtaposed with another zinc finger protein 1	-2.0	2.0E-03
ACSL1	long-chain-fatty-acid--CoA ligase 1	-2.0	2.0E-03
SNX25	sorting nexin 25	-2.0	2.0E-03
FRY	furry homolog (Drosophila)	-2.0	2.0E-03
TBC1D9	TBC1 domain family, member 9 (with GRAM domain)	-2.1	2.0E-03
SHF	Src homology 2 domain containing F	-2.1	2.0E-03
ENSGALG00000029174	novel gene	-2.1	2.0E-03
CRY1	cryptochrome-1	-2.1	2.0E-03
LGR4	leucine-rich repeat containing G protein-coupled receptor 4	-2.2	2.0E-03
ROR2	tyrosine-protein kinase transmembrane receptor *ROR2* precursor	-2.2	2.0E-03
CDCA7L	cell division cycle-associated 7-like protein	-2.2	2.0E-03
MMD	monocyte to macrophage differentiation protein [	-2.2	2.0E-03
MDK	midkine precursor	-2.2	2.0E-03
CHRDL1	chordin-like protein 1 precursor	-2.2	2.0E-03
ID3	inhibitor of DNA binding 3	-2.2	2.0E-03
IGSF3	immunoglobulin superfamily, member 3	-2.2	2.0E-03
DDOST	dolichyl-diphosphooligosaccharide--protein glycosyltransferase 48 kDa subunit	-2.2	2.0E-03

NAPRT1	nicotinate phosphoribosyltransferase domain containing 1	-2.2	2.0E-03
PLCG1	phospholipase C, gamma 1	-2.3	2.0E-03
ALDH18A1	aldehyde dehydrogenase 18 family, member A1	-2.3	2.0E-03
NOTCH1	*notch 1*	-2.3	2.0E-03
NFIL3	nuclear factor interleukin-3-regulated protein	-2.3	2.0E-03
MYD88	Myeloid differentiation primary response protein MyD88	-2.3	2.0E-03
DAB2	Dab, mitogen-responsive phosphoprotein, homolog 2	-2.3	2.0E-03
GPC4	glypican 4	-2.4	2.0E-03
TGIF2	TGFB-induced factor homeobox 2	-2.4	2.0E-03
ADORA2A	adenosine A2a receptor	-2.4	2.0E-03
CPM	carboxypeptidase M precursor	-2.4	2.0E-03
UHRF1BP1	UHRF1 binding protein 1	-2.4	2.0E-03
XRCC5	X-ray repair complementing defective repair in Chinese hamster cells 5 (double-strand-break rejoining)	-2.5	2.0E-03
TRAM2	translocation associated membrane protein 2	-2.5	2.0E-03
SH3BGRL	SH3 domain-binding glutamic acid-rich-like protein	-2.5	2.0E-03
ATP11A	ATPase, class VI, type 11A	-2.5	2.0E-03
GK5	Putative glycerol kinase 5	-2.5	2.0E-03
PAK1	serine/threonine-protein kinase *PAK 1*	-2.6	2.0E-03
EFHD1	EF-hand domain-containing protein D1	-2.6	2.0E-03
EPHB6	ephrin type-B receptor 5 precursor	-2.7	2.0E-03
MYO5A	myosin-Va	-3.0	2.0E-03
STYK1	serine/threonine/tyrosine kinase 1	-3.0	2.0E-03
AQP4	aquaporin-4	-3.4	2.0E-03
VAPA	vesicle-associated membrane protein-associated protein A	-1.8	2.8E-03
LNPEP	leucyl/cystinyl aminopeptidase	-2.0	2.8E-03
RP2	Protein XRP2	-2.0	2.8E-03
DPY19L3	dpy-19-like 3 (C. elegans)	-2.0	2.8E-03
GLT8D1	glycosyltransferase 8 domain-containing protein 1	-2.1	2.8E-03
TMPO	lamina-associated polypeptide 2, isoform beta	-2.1	2.8E-03
TXNDC5	thioredoxin domain-containing protein 5 precursor	-2.1	2.8E-03
ENSGALG00000004676	Uncharacterized protein	-2.2	2.8E-03
ARHGEF17	Rho guanine nucleotide exchange factor (GEF) 17	-2.2	2.8E-03
CDC45	cell division cycle 45	-2.2	2.8E-03
AP1AR	adaptor-related protein complex 1 associated regulatory protein	-2.2	2.8E-03
PYCR2	pyrroline-5-carboxylate reductase family, member 2	-2.3	2.8E-03
BOC	*BOC* cell adhesion associated, oncogene regulated	-2.3	2.8E-03
MANSC1	MANSC domain containing 1 precursor	-2.3	2.8E-03
ATP10D	ATPase, class V, type 10D	-2.5	2.8E-03
ENSGALG00000027352	novel gene	-2.5	2.8E-03
HSPB2	heat shock 27kDa protein 2	-2.6	2.8E-03
MAP4K4	mitogen-activated protein kinase kinase kinase kinase 4	-2.6	2.8E-03
ATP1A1	sodium/potassium-transporting ATPase subunit alpha-1 precursor	-2.8	2.8E-03
EMILIN3	elastin microfibril interfacer 3	-2.9	2.8E-03
ENSGALG00000005204	novel gene	-3.0	2.8E-03
TEF	transcription factor VBP	-3.4	2.8E-03
CER1	cerberus 1, DAN family BMP antagonist	-3.8	2.8E-03
gga-mir-1661	*gga-mir-1661* [Source:miRBase;Acc:MI0007395]	/0	2.8E-03
BCKDHB	2-oxoisovalerate dehydrogenase subunit beta, mitochondrial precursor	-1.8	3.5E-03
SPSB1	splA/ryanodine receptor domain and SOCS box containing 1	-1.9	3.5E-03
LRP2	low density lipoprotein receptor-related protein 2	-1.9	3.5E-03
GNPDA2	Glucosamine-6-phosphate deaminase 2 isoform 1	-1.9	3.5E-03
DOCK1	dedicator of cytokinesis 1	-1.9	3.5E-03
MFSD7	major facilitator superfamily domain containing 7	-2.0	3.5E-03
THSD7B	thrombospondin, type I, domain containing 7B	-2.0	3.5E-03
KIF15	Uncharacterized protein	-2.0	3.5E-03
PTBP1	polypyrimidine tract-binding protein 1	-2.0	3.5E-03
PODXL	podocalyxin-like	-2.1	3.5E-03
OMA1	*OMA1* zinc metallopeptidase	-2.1	3.5E-03
ARL5B	ADP-ribosylation factor-like 5B	-2.1	3.5E-03

DENND2A	DENN/MADD domain containing 2A	-2.1	3.5E-03
FRMD3	FERM domain containing 3	-2.1	3.5E-03
VAT1	vesicle amine transport protein 1 homolog (T. californica)	-2.2	3.5E-03
MTUS1	microtubule associated tumor suppressor 1	-2.2	3.5E-03
MCTP2	multiple C2 domains, transmembrane 2	-2.2	3.5E-03
BCL6	B-cell CLL/lymphoma 6	-2.2	3.5E-03
FAM102A	family with sequence similarity 102, member A	-2.2	3.5E-03
CYTH1	cytohesin-1	-2.2	3.5E-03
MYCN	N-myc proto-oncogene protein	-2.3	3.5E-03
TNFIP6	tumor necrosis factor-inducible gene 6 protein precursor	-2.3	3.5E-03
WNK2	WNK lysine deficient protein kinase 2	-2.4	3.5E-03
FBN1	fibrillin 1	-2.4	3.5E-03
ST3GAL6	ype 2 lactosamine alpha-2,3-sialyltransferase	-2.5	3.5E-03
PROCR	endothelial protein C receptor precursor	-2.5	3.5E-03
C12orf50	chromosome 12 open reading frame 50	-2.6	3.5E-03
PGM5	phosphoglucomutase 5	-2.6	3.5E-03
CENPE	centromere protein E, 312kDa	-2.6	3.5E-03
CHRAC1	chromatin accessibility complex 1	-2.7	3.5E-03
TACC3	transforming, acidic coiled-coil containing protein 3	-2.7	3.5E-03
TYMS	Thymidylate synthase	-2.8	3.5E-03
DAAM2	dishevelled associated activator of morphogenesis 2	-2.9	3.5E-03
CHST9	carbohydrate (N-acetylgalactosamine 4-0) sulfotransferase 9	-3.1	3.5E-03
C1R	complement component 1, r subcomponent	-3.1	3.5E-03
CDH6	Cadherin-6	-3.1	3.5E-03
CYP26C1	cytochrome P450, family 26, subfamily C, polypeptide 1	-3.5	3.5E-03
ZNF385D	zinc finger protein 385D	-3.5	3.5E-03
MXRA5	matrix-remodelling associated 5	-3.6	3.5E-03
CISH	Cytokine-inducible SH2-containing protein	-1.8	4.2E-03
FBXL2	F-box and leucine-rich repeat protein 2	-1.9	4.2E-03
BUB1	*BUB1* mitotic checkpoint serine/threonine kinase	-1.9	4.2E-03
SMCHD1	structural maintenance of chromosomes flexible hinge domain containing 1	-1.9	4.2E-03
PANK1	pantothenate kinase 1	-1.9	4.2E-03
FAM177A1	family with sequence similarity 177, member A1	-1.9	4.2E-03
CBX1	chromobox protein homolog 1	-1.9	4.2E-03
SETBP1	SET binding protein 1	-1.9	4.2E-03
ACTL6A	actin-like 6A	-1.9	4.2E-03
MPP6	membrane protein, palmitoylated 6 (MAGUK p55 subfamily member 6)	-2.0	4.2E-03
PIGA	N-acetylglucosaminyl-phosphatidylinositol biosynthetic protein	-2.0	4.2E-03
ABL1	c-abl oncogene 1, non-receptor tyrosine kinase	-2.0	4.2E-03
ZNHIT6	zinc finger, HIT-type containing 6	-2.0	4.2E-03
SLC31A1	solute carrier family 31 (copper transporter), member 1	-2.0	4.2E-03
SGIP1	SH3-domain GRB2-like (endophilin) interacting protein 1	-2.0	4.2E-03
BACE2	beta-site APP-cleaving enzyme 2	-2.1	4.2E-03
ATP2A2	ATPase, Ca++ transporting, cardiac muscle, slow twitch 2	-2.1	4.2E-03
ENSGALG00000014164	Uncharacterized protein	-2.2	4.2E-03
PHYHIPL	phytanoyl-CoA hydroxylase-interacting protein-like	-2.2	4.2E-03
ZNF395	zinc finger protein 395	-2.2	4.2E-03
CREB3L2	cAMP responsive element binding protein 3-like 2	-2.3	4.2E-03
GTF2H4	general transcription factor IIH, polypeptide 4, 52kDa	-2.4	4.2E-03
FAM114A1	family with sequence similarity 114, member A1	-2.4	4.2E-03
FKBP14	FK506 binding protein 14, 22 kDa	-2.5	4.2E-03
ADRA1B	adrenoceptor alpha 1B	-2.7	4.2E-03
SPOCK1	sparc/osteonectin, cwcv and kazal-like domains proteoglycan (testican) 1	-3.3	4.2E-03
FARS2	phenylalanyl-tRNA synthetase 2, mitochondrial	-1.7	4.8E-03
ZDHHC21	zinc finger, DHHC-type containing 21	-1.8	4.8E-03
NEK7	NIMA-related kinase 7	-1.9	4.8E-03
C20orf194	chromosome 20 open reading frame 194	-1.9	4.8E-03
KIF4A	chromosome-associated kinesin KIF4	-1.9	4.8E-03
ZNF521	zinc finger protein 521	-1.9	4.8E-03

RECK	reversion-inducing-cysteine-rich protein with kazal motifs	-1.9	4.8E-03
AS3MT	arsenic (+3 oxidation state) methyltransferase	-2.0	4.8E-03
ENSGALG00000007131	Non-histone chromosomal protein HMG-14A	-2.0	4.8E-03
ENSGALG00000028322	novel gene	-2.1	4.8E-03
SCAMP5	secretory carrier membrane protein 5	-2.1	4.8E-03
GPR27	G protein-coupled receptor 27	-2.2	4.8E-03
ICK	intestinal cell (MAK-like) kinase	-2.3	4.8E-03
ARHGAP39	Rho GTPase activating protein 39	-2.4	4.8E-03
BRCA1	breast cancer 1, early onset	-2.5	4.8E-03
CENPW	centromere protein W	-2.5	4.8E-03
TGFB2	transforming growth factor, beta 2	-2.5	4.8E-03
GJA1	gap junction protein, alpha 1, 43kDa	-3.1	4.8E-03
WDR35	WD repeat domain 35	-1.9	5.4E-03
RIF1	RAP1 interacting factor homolog (yeast)	-1.9	5.4E-03
FZD10	frizzled family receptor 10	-1.9	5.4E-03
LRCH3	leucine-rich repeats and calponin homology (CH) domain containing 3	-1.9	5.4E-03
BCL11A	B-cell CLL/lymphoma 11A (zinc finger protein)	-1.9	5.4E-03
DUSP19	dual specificity phosphatase 19	-2.0	5.4E-03
MCM2	minichromosome maintenance complex component 2	-2.0	5.4E-03
NUSAP1	nucleolar and spindle associated protein 1	-2.1	5.4E-03
PHACTR1	phosphatase and actin regulator 1	-2.2	5.4E-03
ENSGALG00000023464	Uncharacterized protein	-2.3	5.4E-03
HDAC10	histone deacetylase 10	-2.3	5.4E-03
SS3R	somatostatin receptor 3	-2.4	5.4E-03
IKBIP	IKBKB interacting protein	-2.5	5.4E-03
ENSGALG00000005969	novel gene	-2.5	5.4E-03
KCTD15	potassium channel tetramerization domain containing 15	-2.6	5.4E-03
DNAH17	dynein, axonemal, heavy chain 17	-2.7	5.4E-03
SEMA3G	sema domain, immunoglobulin domain (Ig), short basic domain, secreted, (semaphorin) 3G	-3.2	5.4E-03
ENSGALG00000016326	Uncharacterized protein	-3.5	5.4E-03
C1QTNF7	C1q and tumor necrosis factor related protein 7	-3.5	5.4E-03
ACTN1	actinin, alpha 1	-1.7	6.0E-03
DNMT3B	DNA (cytosine-5-)-methyltransferase 3 beta	-1.9	6.0E-03
NPR3	natriuretic peptide receptor C/guanylate cyclase C (atrionatriuretic peptide receptor C)	-1.9	6.0E-03
H2AFY2	H2A histone family, member Y2	-1.9	6.0E-03
KAZALD1	Kazal-type serine peptidase inhibitor domain 1	-1.9	6.0E-03
KANK1	KN motif and ankyrin repeat domains 1	-2.0	6.0E-03
AMPD2	adenosine monophosphate deaminase 2	-2.3	6.0E-03
EPB41L4B	erythrocyte membrane protein band 4.1 like 4B	-2.3	6.0E-03
RAB11FIP4	RAB11 family interacting protein 4 (class II)	-2.4	6.0E-03
EP3	prostaglandin E receptor 3 (subtype *EP3*)	-2.4	6.0E-03
LGALSL	lectin, galactoside-binding-like	-2.8	6.0E-03
gga-mir-454	*gga-mir-454* [Source:miRBase;Acc:MI0006984]	/0	6.0E-03
ENSGALG00000001604	novel gene	-1.8	6.5E-03
NALCN	sodium leak channel, non-selective	-1.8	6.5E-03
KIAA1211L	KIAA1211-like	-2.0	6.5E-03
CKAP4	cytoskeleton-associated protein 4	-2.2	6.5E-03
NR3C1	glucocorticoid receptor	-2.2	6.5E-03
BUB1B	mitotic checkpoint serine/threonine-protein kinase BUB1 beta	-2.2	6.5E-03
EPB41L2	erythrocyte membrane protein band 4.1-like 2	-2.3	6.5E-03
ADRBK2,MYO18B	adrenergic, beta, receptor kinase 2	-2.4	6.5E-03
TXNRD1	thioredoxin reductase 1	-2.7	6.5E-03
THBS1	thrombospondin 1	-3.0	6.5E-03
CTCF	CCCTC-binding factor (zinc finger protein)	-1.7	7.0E-03
E2F5	E2F transcription factor 5, p130-binding	-1.8	7.0E-03
RDH10	retinol dehydrogenase 10 (all-trans)	-1.8	7.0E-03
TRIL	TLR4 interactor with leucine-rich repeats	-1.8	7.0E-03
GRHPR	glyoxylate reductase/hydroxypyruvate reductase	-1.9	7.0E-03
SGK3	serum/glucocorticoid regulated kinase family, member 3	-1.9	7.0E-03

ARHGEF3	Rho guanine nucleotide exchange factor (GEF) 3	-1.9	7.0E-03
CTSO	cathepsin O	-1.9	7.0E-03
NEDD4L	neural precursor cell expressed, developmentally down-regulated 4-like, E3 ubiquitin protein ligase	-1.9	7.0E-03
FBLN7	fibulin 7	-2.0	7.0E-03
CBX3	chromobox homolog 3	-2.0	7.0E-03
HEBP1	heme binding protein 1	-2.0	7.0E-03
NPAS3	neuronal PAS domain protein 3	-2.1	7.0E-03
TSHZ3	teashirt zinc finger homeobox 3	-2.2	7.0E-03
CASKIN1	CASK interacting protein 1	-2.2	7.0E-03
TK1	thymidine kinase 1, soluble	-2.2	7.0E-03
KLHL3	kelch-like family member 3	-2.3	7.0E-03
ENSGALG00000010722	Schwann cell-specific EGF-like repeat autocrine factor precursor	-2.3	7.0E-03
PI15	Peptidase inhibitor 15	-1.8	7.5E-03
TFDP2	transcription factor Dp-2	-1.8	7.5E-03
N6AMT2	N(6)-adenine-specific DNA methyltransferase 2	-1.8	7.5E-03
FTD	Ferritoid; Uncharacterized protein	-1.8	7.5E-03
ASNS	asparagine synthetase (glutamine-hydrolyzing)	-1.8	7.5E-03
AMY1A	amylase, alpha 1A (salivary)	-1.8	7.5E-03
FGD3	FYVE, RhoGEF and PH domain containing 3	-1.8	7.5E-03
HMGN2	high mobility group nucleosomal binding domain 2	-1.9	7.5E-03
EXOC3L1	exocyst complex component 3-like 1	-1.9	7.5E-03
CCDC47	coiled-coil domain containing 47	-1.9	7.5E-03
CDCA7	cell division cycle associated 7	-1.9	7.5E-03
TGIF1	TGFB-induced factor homeobox 1	-2.0	7.5E-03
RFTN1	raftlin, lipid raft linker 1	-2.0	7.5E-03
TCF7L2	transcription factor 7-like 2 (T-cell specific, HMG-box)	-2.0	7.5E-03
MMP17	matrix metallopeptidase 17 (membrane-inserted)	-2.2	7.5E-03
HSP90B1	heat shock protein 90kDa beta (Grp94), member 1	-2.3	7.5E-03
LURAP1	leucine rich adaptor protein 1	-2.4	7.5E-03
PGM2	phosphoglucomutase 2	-2.5	7.5E-03
PRSS12	protease, serine, 12 (neurotrypsin, motopsin)	-2.7	7.5E-03
AKAP7	A kinase (PRKA) anchor protein 7	-3.0	7.5E-03
EDG7	lysophosphatidic acid receptor 3	-4.1	7.5E-03
POFUT1	GDP-fucose protein O-fucosyltransferase 1 precursor	-1.7	8.0E-03
ENSGALG00000012412	novel gene	-1.7	8.0E-03
LCLAT1	Lysocardiolipin acyltransferase 1	-1.7	8.0E-03
PHLPP1	PH domain and leucine rich repeat protein phosphatase 1	-1.7	8.0E-03
FARP1	FERM, RhoGEF (ARHGEF) and pleckstrin domain protein 1 (chondrocyte-derived)	-1.7	8.0E-03
PRKAA1	protein kinase, AMP-activated, alpha 1 catalytic subunit	-1.8	8.0E-03
GLI3	GLI family zinc finger 3	-1.8	8.0E-03
RALGAPA1	Ral GTPase activating protein, alpha subunit 1 (catalytic)	-1.9	8.0E-03
CCNB2	G2/mitotic-specific cyclin-B2	-1.9	8.0E-03
ADSS	Adenylosuccinate synthetase isozyme 2	-1.9	8.0E-03
IGDCC4	immunoglobulin superfamily, DCC subclass, member 4	-2.0	8.0E-03
CDK2AP1	cyclin-dependent kinase 2-associated protein 1	-2.2	8.0E-03
GUCY2F	guanylate cyclase 2F, retinal	-3.3	8.0E-03
KDELC1	KDEL (Lys-Asp-Glu-Leu) containing 1	-1.6	8.5E-03
UGP2	UDP-glucose pyrophosphorylase 2	-1.7	8.5E-03
ZBTB2	zinc finger and BTB domain containing 2	-1.8	8.5E-03
OSBPL6	oxysterol binding protein-like 6	-1.9	8.5E-03
GRAMD3	GRAM domain containing 3	-1.9	8.5E-03
ENSGALG00000016141	novel gene	-1.9	8.5E-03
FAM117B	family with sequence similarity 117, member B	-2.4	8.5E-03
PPP4R1	protein phosphatase 4, regulatory subunit 1	-1.7	9.0E-03
COMMD10	COMM domain-containing protein 10	-1.7	9.0E-03
RCN2	reticulocalbin 2, EF-hand calcium binding domain	-1.8	9.0E-03
DKC1	dyskeratosis congenita 1, dyskerin	-1.8	9.0E-03
TPGS2	tubulin polyglutamylase complex subunit 2	-1.9	9.0E-03
RBBP7	retinoblastoma binding protein 7	-2.0	9.0E-03
MLH1	mutL homolog 1, colon cancer, nonpolyposis type 2 (E. coli)	-2.1	9.0E-03

DCTD	dCMP deaminase	-2.1	9.0E-03
XYLB	xylulokinase homolog (H. influenzae)	-2.4	9.0E-03
PERP	*PERP*, TP53 apoptosis effector	-2.5	9.0E-03
CAB39L	calcium binding protein 39-like	-3.8	9.0E-03
PRKCA	protein kinase C, alpha	-1.7	9.5E-03
TBC1D2B	TBC1 domain family, member 2B	-1.7	9.5E-03
FAM188B	family with sequence similarity 188, member B	-1.7	9.5E-03
TMEM68	transmembrane protein 68	-1.7	9.5E-03
MAD2L1	MAD2 mitotic arrest deficient-like 1	-1.8	9.5E-03
FAM135A	family with sequence similarity 135, member A	-1.8	9.5E-03
CAMKK2	calcium/calmodulin-dependent protein kinase kinase 2, beta	-1.8	9.5E-03
SPRY2	sprouty homolog 2 (Drosophila)	-2.0	9.5E-03
CHST3	carbohydrate (chondroitin 6) sulfotransferase 3	-2.0	9.5E-03
KCNIP2	Kv channel interacting protein 2	-2.1	9.5E-03
CAMK2D	calcium/calmodulin-dependent protein kinase II delta	-2.2	9.5E-03
PRKAB1	protein kinase, AMP-activated, beta 1 non-catalytic subunit	-1.6	1.0E-02
ZNF518B	zinc finger protein 518B	-1.7	1.0E-02
CHN1	chimerin 1	-1.7	1.0E-02
EFR3A	EFR3 homolog A (S. cerevisiae)	-1.8	1.0E-02
LMNA	lamin A/C	-1.8	1.0E-02
PPP2R3A	protein phosphatase 2, regulatory subunit B,,, alpha	-1.8	1.0E-02
XBP1	X-box binding protein 1	-1.9	1.0E-02
OSBPL3	oxysterol binding protein-like 3	-2.0	1.0E-02
C5ORF13	neuronal protein 3.1	-2.6	1.0E-02
SEPP1	selenoprotein P, plasma, 1	-2.9	1.0E-02
AGXT2L1	alanine-glyoxylate aminotransferase 2-like 1	-2.9	1.0E-02
RNaseP_nuc	Nuclear RNase P	-4.8	1.0E-02
ZMYND11	zinc finger, MYND-type containing 11	-1.7	1.0E-02
SACS	spastic ataxia of Charlevoix-Saguenay (sacsin)	-1.8	1.0E-02
CPNE3	copine III	-1.9	1.0E-02
USP12	ubiquitin specific peptidase 12	-2.0	1.0E-02
SNED1	sushi, nidogen and EGF-like domains 1	-2.6	1.0E-02
STEAP2	STEAP family member 2, metalloreductase	-3.1	1.0E-02
PCDH1	protocadherin 1	-1.6	1.1E-02
ENSGALG00000002852	novel gene	-1.7	1.1E-02
GPR161,TTF2	transcription termination factor, RNA polymerase II	-1.7	1.1E-02
FOXM1	forkhead box M1	-1.8	1.1E-02
SIPA1L2	signal-induced proliferation-associated 1 like 2	-1.8	1.1E-02
IL11RA	interleukin 11 receptor, alpha	-1.8	1.1E-02
DAP	death-associated protein	-1.8	1.1E-02
POMT1	protein-O-mannosyltransferase 1	-1.8	1.1E-02
PLK1	polo-like kinase 1	-2.0	1.1E-02
POLA1	polymerase (DNA directed), alpha 1, catalytic subunit	-2.0	1.1E-02
CKI	beta-1,4-galactosyltransferase 1	-2.1	1.1E-02
GAS2	growth arrest-specific 2	-2.3	1.1E-02
U1	*U1* spliceosomal RNA	/0	1.1E-02
NEDD1	neural precursor cell expressed, developmentally down-regulated 1	-1.6	1.1E-02
B3GALTL	beta 1,3-galactosyltransferase-like	-1.8	1.1E-02
COL4A2	collagen, type IV, alpha 2	-1.9	1.1E-02
DYNC2LI1	dynein, cytoplasmic 2, light intermediate chain 1	-1.9	1.1E-02
DERL3	derlin 3	-1.9	1.1E-02
SNTA1	syntrophin, alpha 1	-2.0	1.1E-02
TAGLN	transgelin	-2.4	1.1E-02
gga-mir-30d	*gga-mir-30d* [Source:miRBase;Acc:MI0001198]	/0	1.1E-02
NCAPD2	non-SMC condensin I complex, subunit D2	-1.7	1.2E-02
ENSGALG00000027754	novel gene	-1.8	1.2E-02
CAMK1D	calcium/calmodulin-dependent protein kinase ID	-1.8	1.2E-02
LRIG3	leucine-rich repeats and immunoglobulin-like domains 3	-1.8	1.2E-02
PTGFRN	prostaglandin F2 receptor inhibitor	-1.8	1.2E-02
IKBIP	IKBKB interacting protein	-1.9	1.2E-02
GSTA	Glutathione S-transferase	-1.9	1.2E-02
SMAD1	SMAD family member 1	-1.9	1.2E-02

HTR7	5-hydroxytryptamine (serotonin) receptor 7, adenylate cyclase-coupled	-1.9	1.2E-02
VANGL2	VANGL planar cell polarity protein 2	-2.0	1.2E-02
NCAPG	non-SMC condensin I complex, subunit G	-2.0	1.2E-02
ENSGALG00000008755	novel gene	-2.3	1.2E-02
S1PR3	sphingosine-1-phosphate receptor 3	-2.5	1.2E-02
MKL2	MKL/myocardin-like 2	-1.7	1.3E-02
POLD3	polymerase (DNA-directed), delta 3, accessory subunit	-1.7	1.3E-02
IRF2BPL	interferon regulatory factor 2 binding protein-like	-1.8	1.3E-02
SRSF3	serine/arginine-rich splicing factor 3	-1.8	1.3E-02
BCL9L	B-cell CLL/lymphoma 9-like	-1.8	1.3E-02
ENSGALG00000015653	junctophilin 1	-1.8	1.3E-02
EPB41L3	erythrocyte membrane protein band 4.1-like 3	-2.0	1.3E-02
SEMA5B	sema domain, seven thrombospondin repeats (type 1 and type 1-like), transmembrane domain (TM) and short cytoplasmic domain, (semaphorin) 5B	-2.1	1.3E-02
PIGQ	phosphatidylinositol glycan anchor biosynthesis, class Q	-2.3	1.3E-02
PLXNA4	plexin A4	-4.6	1.3E-02
FUCA1	fucosidase, alpha-L- 1, tissue	-1.6	1.3E-02
ARPC5	actin related protein 2/3 complex, subunit 5, 16kDa	-1.7	1.3E-02
USP54	ubiquitin specific peptidase 54	-1.7	1.3E-02
CCNE1	cyclin E1	-1.8	1.3E-02
ACSF2	acyl-CoA synthetase family member 2	-1.9	1.3E-02
MCM3	minichromosome maintenance complex component 3	-1.9	1.3E-02
PPFIBP1	PTPRF interacting protein, binding protein 1 (liprin beta 1)	-1.9	1.3E-02
ANKS6	ankyrin repeat and sterile alpha motif domain containing 6	-2.5	1.3E-02
ARG2	arginase 2	-2.7	1.3E-02
MATN2	matrilin 2	-4.3	1.3E-02
CORTBP2	cortactin binding protein 2	-1.6	1.3E-02
ENSGALG00000008567	novel gene	-1.6	1.3E-02
C3ORF55	Uncharacterized protein	-1.7	1.3E-02
CCDC102A	coiled-coil domain containing 102A	-1.7	1.3E-02
GALNTL4	UDP-N-acetyl-alpha-D-galactosamine:polypeptide N-acetylgalactosaminyltransferase 18	-1.7	1.3E-02
ZMYND8	zinc finger, MYND-type containing 8	-1.8	1.3E-02
ADARB1	adenosine deaminase, RNA-specific, B1	-1.9	1.3E-02
C14ORF135	Uncharacterized protein	-1.9	1.3E-02
FBLN1	fibulin 1	-1.9	1.3E-02
ELOVL7	ELOVL fatty acid elongase 7	-2.1	1.3E-02
SPOCK3	sparc/osteonectin, cwcv and kazal-like domains proteoglycan (testican) 3	-2.2	1.3E-02
USP3	ubiquitin specific peptidase 3	-1.7	1.4E-02
PROM1	prominin 1	-1.9	1.4E-02
CCNB3	cyclin B3	-1.9	1.4E-02
TRPC6	transient receptor potential cation channel, subfamily C, member 6	-1.9	1.4E-02
WNT5A	wingless-type MMTV integration site family, member 5A	-2.0	1.4E-02
IMPA2	inositol(myo)-1(or 4)-monophosphatase 2	-2.3	1.4E-02
SHISA5	shisa homolog 5 (Xenopus laevis)	-1.7	1.4E-02
PQLC1	PQ loop repeat containing 1	-1.8	1.4E-02
P4HA2	prolyl 4-hydroxylase, alpha polypeptide II	-1.8	1.4E-02
EHHADH	enoyl-CoA, hydratase/3-hydroxyacyl CoA dehydrogenase	-1.8	1.4E-02
ENSGALG00000004132	novel gene	-1.9	1.4E-02
FBP1	fructose-1,6-bisphosphatase 1	-2.7	1.4E-02
ER81	ETS translocation variant 1	-1.7	1.5E-02
SLC13A3	solute carrier family 13 (sodium-dependent dicarboxylate transporter), member 3	-1.7	1.5E-02
CHAF1B	chromatin assembly factor 1, subunit B (p60)	-1.8	1.5E-02
NPM1	nucleophosmin (nucleolar phosphoprotein B23, numatrin)	-2.4	1.5E-02
TRPM1	transient receptor potential cation channel, subfamily M, member 1	-2.8	1.5E-02
SCRT2	scratch homolog 2, zinc finger protein (Drosophila)	-3.3	1.5E-02
LRRC16A	leucine rich repeat containing 16A	-1.6	1.5E-02

PTPRD	protein tyrosine phosphatase, receptor type, D	-1.6	1.5E-02
C28H19ORF10	UPF0556 protein C19orf10 homolog precursor	-1.7	1.5E-02
D2HGDH	D-2-hydroxyglutarate dehydrogenase	-1.7	1.5E-02
RREB1	ras responsive element binding protein 1	-1.7	1.5E-02
ENSGALG00000015577	novel gene	-1.9	1.5E-02
SDCBP	syndecan binding protein (syntenin)	-2.5	1.5E-02
MTHFR	methylenetetrahydrofolate reductase (NAD(P)H)	-1.6	1.6E-02
HSPA5	heat shock 70kDa protein 5 (glucose-regulated protein, 78kDa)	-1.9	1.6E-02
ENSGALG00000014513	novel gene	-2.0	1.6E-02
AMER2	APC membrane recruitment protein 2	-2.1	1.6E-02
TNFRSF1A	tumor necrosis factor receptor superfamily, member 1A	-1.6	1.6E-02
DNAL1	dynein, axonemal, light chain 1	-1.6	1.6E-02
RWDD3	RWD domain containing 3	-1.6	1.6E-02
MPP5	membrane protein, palmitoylated 5 (MAGUK p55 subfamily member 5)	-1.6	1.6E-02
CRELD2	cysteine-rich with EGF-like domains 2	-1.7	1.6E-02
ABHD6	abhydrolase domain containing 6	-1.8	1.6E-02
SUN2	Sad1 and UNC84 domain containing 2	-1.9	1.6E-02
ZDHHC12	zinc finger, DHHC-type containing 12	-2.1	1.6E-02
KIAA1147	*KIAA1147*	-2.1	1.6E-02
CASP6	caspase 6, apoptosis-related cysteine peptidase	-2.4	1.6E-02
CHEK1	checkpoint kinase 1	-2.5	1.6E-02
EML4	echinoderm microtubule associated protein like 4	-2.6	1.6E-02
FABP3	fatty acid binding protein 3, muscle and heart (mammary-derived growth inhibitor)	-4.1	1.6E-02
CD2AP	CD2-associated protein	-1.6	1.6E-02
TDP1	tyrosyl-DNA phosphodiesterase 1	-1.8	1.6E-02
RNFT1	ring finger protein, transmembrane 1	-1.8	1.6E-02
RCOR1	REST corepressor 1	-1.9	1.6E-02
ZNF488	zinc finger protein 488	-2.4	1.6E-02
ASB13	ankyrin repeat and SOCS box containing 13	-2.6	1.6E-02
HIF1A	hypoxia inducible factor 1, alpha subunit (basic helix-loop-helix transcription factor)	-2.6	1.6E-02
RAD51A	RAD51 recombinase	-2.7	1.6E-02
ENSGALG00000026521	novel gene	-3.0	1.6E-02
BRI3BP	BRI3 binding protein	-1.7	1.7E-02
FAM122A	family with sequence similarity 122A	-1.7	1.7E-02
SNAPC3	small nuclear RNA activating complex, polypeptide 3, 50kDa	-1.9	1.7E-02
N4BP2	NEDD4 binding protein 2	-1.6	1.7E-02
ASPM	asp (abnormal spindle) homolog, microcephaly associated (Drosophila)	-1.6	1.7E-02
ARSK	arylsulfatase family, member K	-1.6	1.7E-02
BAZ1A	bromodomain adjacent to zinc finger domain, 1A	-1.7	1.7E-02
SLC16A5	solute carrier family 16, member 5 (monocarboxylic acid transporter 6)	-1.7	1.7E-02
SLC6A15	solute carrier family 6 (neutral amino acid transporter), member 15	-1.8	1.7E-02
ENSGALG00000009432	Uncharacterized protein	-2.1	1.7E-02
UGT8	UDP glycosyltransferase 8	-2.5	1.7E-02
ABCG1	ATP-binding cassette, sub-family G (WHITE), member 1	-2.8	1.7E-02
ENSGALG00000009432	Uncharacterized protein	-1.6	1.7E-02
SPRED2	sprouty-related, EVH1 domain containing 2	-1.8	1.7E-02
DFNA5	deafness, autosomal dominant 5	-1.9	1.7E-02
SAE2	ubiquitin-like modifier activating enzyme 2	-1.5	1.8E-02
C3H6ORF72	uncharacterized protein C6orf72 homolog precursor	-1.6	1.8E-02
BAMBI	BMP and activin membrane-bound inhibitor homolog (Xenopus laevis)	-1.6	1.8E-02
EML1	echinoderm microtubule associated protein like 1	-1.6	1.8E-02
FAM98B	family with sequence similarity 98, member B	-1.7	1.8E-02
NFYB	nuclear transcription factor Y, beta	-1.7	1.8E-02
FRS2	fibroblast growth factor receptor substrate 2	-1.8	1.8E-02
PTPRF	protein tyrosine phosphatase, receptor type, F	-2.1	1.8E-02

BTF3	basic transcription factor 3	-1.6	1.8E-02
TOP2A	topoisomerase (DNA) II alpha 170kDa	-1.6	1.8E-02
FAM108C1	Abhydrolase domain-containing protein *FAM108C1*	-1.6	1.8E-02
IFT81	intraflagellar transport 81 homolog (Chlamydomonas)	-1.9	1.8E-02
TNPO1	transportin 1	-1.5	1.9E-02
PXMP3	peroxin 2	-1.6	1.9E-02
VAV2	*vav 2* guanine nucleotide exchange factor	-1.6	1.9E-02
MEIS1	Meis homeobox 1	-1.7	1.9E-02
GMPR	guanosine monophosphate reductase	-2.3	1.9E-02
SOWAHC	sosondowah ankyrin repeat domain family member C	-1.5	1.9E-02
C3ORF23	Uncharacterized protein	-1.5	1.9E-02
PDIA5	protein disulfide isomerase family A, member 5	-1.6	1.9E-02
RYBP	RING1 and YY1 binding protein	-1.7	1.9E-02
RCN1	reticulocalbin 1, EF-hand calcium binding domain	-1.8	1.9E-02
CMAS	cytidine monophosphate N-acetylneuraminic acid synthetase	-1.9	1.9E-02
IRF6	interferon regulatory factor 6	-2.1	1.9E-02
HSP90AB1	heat shock protein 90kDa alpha (cytosolic), class B member 1	-2.2	1.9E-02
CCNA1	cyclin A1	-2.2	1.9E-02
TSSC4	tumor suppressing subtransferable candidate 4	-1.5	1.9E-02
PARD6G	par-6 partitioning defective 6 homolog gamma (C. elegans)	-1.7	1.9E-02
PLCH1	phospholipase C, eta 1	-1.8	1.9E-02
EIF2C1	Uncharacterized protein	-1.9	1.9E-02
RXRA	retinoid X receptor, alpha	-1.6	2.0E-02
MT4	metallothionein 4	-1.6	2.0E-02
ALDH4A1	aldehyde dehydrogenase 4 family, member A1	-1.7	2.0E-02
GNE	glucosamine (UDP-N-acetyl)-2-epimerase/N-acetylmannosamine kinase	-1.7	2.0E-02
CAPN5	calpain 5	-1.9	2.0E-02
UGGT2	UDP-glucose glycoprotein glucosyltransferase 2	-1.5	2.0E-02
HACE1	HECT domain and ankyrin repeat containing E3 ubiquitin protein ligase 1	-1.5	2.0E-02
DCPS	decapping enzyme, scavenger	-1.6	2.0E-02
SLC25A12	solute carrier family 25 (aspartate/glutamate carrier), member 12	-1.6	2.0E-02
MCM5	minichromosome maintenance complex component 5	-1.9	2.0E-02
RALGPS1	Ral GEF with PH domain and SH3 binding motif 1	-2.0	2.0E-02
SNORD95	small nucleolar RNA, C/D box 95	/0	2.0E-02
LEPREL1	leprecan-like 1	-1.5	2.0E-02
POLE3	polymerase (DNA directed), epsilon 3, accessory subunit	-1.6	2.0E-02
ENSGALG00000012158	novel gene	-1.7	2.0E-02
C12orf5	chromosome 12 open reading frame 5	-1.7	2.0E-02
RNF130	ring finger protein 130	-1.9	2.0E-02
FSCN1	fascin homolog 1, actin-bundling protein (Strongylocentrotus purpuratus)	-2.0	2.0E-02
HUS1	*HUS1* checkpoint homolog (S. pombe)	-2.4	2.0E-02
DBT	dihydrolipoamide branched chain transacylase E2	-1.5	2.1E-02
TMEM164	transmembrane protein 164	-1.6	2.1E-02
C6orf70	chromosome 6 open reading frame 70	-1.6	2.1E-02
SMAD3	SMAD family member 3	-1.7	2.1E-02
DMD	dystrophin	-1.8	2.1E-02
LIMD2	LIM domain containing 2	-2.0	2.1E-02
ENSGALG00000006407	death domain-containing tumor necrosis factor receptor superfamily member 23 precursor	-2.9	2.1E-02
PTTG1IP	pituitary tumor-transforming 1 interacting protein	-1.6	2.1E-02
RNASEL	ribonuclease L (2,,5,-oligoisoadenylate synthetase-dependent)	-2.3	2.1E-02
TRIM9	tripartite motif containing 9	-1.6	2.2E-02
RACGAP1	Rac GTPase activating protein 1	-1.9	2.2E-02
THADA	thyroid adenoma associated	-1.5	2.2E-02
C9ORF4	Uncharacterized protein	-1.5	2.2E-02
GPR155	G protein-coupled receptor 155	-1.5	2.2E-02

ENSGALG00000014190	novel gene	-1.5	2.2E-02
SLC25A13	solute carrier family 25 (aspartate/glutamate carrier), member 13	-1.6	2.2E-02
ENSGALG00000013233	novel gene	-1.7	2.2E-02
SLC1A2	solute carrier family 1 (glial high affinity glutamate transporter), member 2	-1.8	2.2E-02
AP1S2	adaptor-related protein complex 1, sigma 2 subunit	-1.9	2.2E-02
NRP2	neuropilin 2	-2.0	2.2E-02
C13ORF7	Uncharacterized protein	-1.5	2.2E-02
BUB3	*BUB3* mitotic checkpoint protein	-1.5	2.2E-02
ARHGDIB	Rho GDP dissociation inhibitor (GDI) beta	-1.5	2.2E-02
SULT1E1	sulfotransferase family 1E, estrogen-preferring, member 1	-1.6	2.2E-02
CDK2	cyclin-dependent kinase 2	-2.3	2.2E-02
SMAD6	SMAD family member 6	-1.7	2.3E-02
CASK	calcium/calmodulin-dependent serine protein kinase (MAGUK family)	-1.9	2.3E-02
EFNB2	ephrin-B2	-2.0	2.3E-02
CXORF41	novel gene	-2.3	2.3E-02
NRARP	NOTCH-regulated ankyrin repeat protein	-1.6	2.3E-02
LBH	limb bud and heart development	-1.8	2.3E-02
RASL11A	RAS-like, family 11, member A	-2.9	2.3E-02
SMARCD3	SWI/SNF related, matrix associated, actin dependent regulator of chromatin, subfamily d, member 3	-1.6	2.3E-02
ACER3	alkaline ceramidase 3	-1.6	2.3E-02
SLC40A1	solute carrier family 40 (iron-regulated transporter), member 1	-1.6	2.3E-02
USP6NL	USP6 N-terminal like	-1.6	2.3E-02
SUSD1	sushi domain containing 1	-2.3	2.3E-02
KCNIP4	Kv channel interacting protein 4	-3.3	2.3E-02
SLC35C2	solute carrier family 35 (GDP-fucose transporter), member C2	-1.5	2.4E-02
PPDPF	pancreatic progenitor cell differentiation and proliferation factor homolog (zebrafish)	-1.6	2.4E-02
RHOBTB1	Rho-related BTB domain containing 1	-1.8	2.4E-02
ENSGALG00000002475,ENSGALG00000027637	Uncharacterized protein	-1.7	2.4E-02
B3GNT2	UDP-GlcNAc:betaGal beta-1,3-N-acetylglucosaminyltransferase 2	-2.1	2.4E-02
AKAP11	A kinase (PRKA) anchor protein 11	-1.5	2.4E-02
WTAP	Wilms tumor 1 associated protein	-1.5	2.4E-02
SWAP70	SWAP switching B-cell complex 70kDa subunit	-1.6	2.4E-02
FOXP2	forkhead box P2	-1.6	2.4E-02
NTN1	netrin 1	-1.8	2.4E-02
FAM19A3	family with sequence similarity 19 (chemokine (C-C motif)-like), member A3	-2.2	2.4E-02
HMG20A	high mobility group 20A	-1.5	2.5E-02
PPWD1	peptidylprolyl isomerase domain and WD repeat containing 1	-1.5	2.5E-02
CEP95	centrosomal protein 95kDa	-1.6	2.5E-02
SEMA6A	sema domain, transmembrane domain (TM), and cytoplasmic domain, (semaphorin) 6A	-1.6	2.5E-02
PPIB	peptidylprolyl isomerase B (cyclophilin B)	-1.7	2.5E-02
FLT4	fms-related tyrosine kinase 4	-1.7	2.5E-02
H2AFV	H2A histone family, member V	-1.4	2.5E-02
MAPKBP1	mitogen-activated protein kinase binding protein 1	-1.5	2.5E-02
SLC6A9	solute carrier family 6 (neurotransmitter transporter, glycine), member 9	-1.5	2.5E-02
SULT1B1	sulfotransferase family, cytosolic, 1B, member 1	-1.5	2.5E-02
INTS4	integrator complex subunit 4	-1.6	2.5E-02
NXPE3	neurexophilin and PC-esterase domain family, member 3	-2.0	2.5E-02
CCND1	cyclin D1	-2.3	2.5E-02
SNORD16	small nucleolar RNA, C/D box 16	/0	2.5E-02
NMNAT1	nicotinamide nucleotide adenylyltransferase 1	-1.5	2.5E-02

ZC3H12B	zinc finger CCCH-type containing 12B	-1.6	2.5E-02
TRIM13	tripartite motif containing 13	-1.6	2.5E-02
CHST11	carbohydrate (chondroitin 4) sulfotransferase 11	-1.6	2.5E-02
PPIP5K2	diphosphoinositol pentakisphosphate kinase 2	-1.6	2.5E-02
FAM110C	family with sequence similarity 110, member C	-2.2	2.5E-02
FAM54A	Uncharacterized protein	-2.3	2.5E-02
RPS6KA3	ribosomal protein S6 kinase, 90kDa, polypeptide 3	-1.4	2.6E-02
SGCD	sarcoglycan, delta (35kDa dystrophin-associated glycoprotein)	-2.5	2.6E-02
ITSN1	intersectin 1 (SH3 domain protein)	-1.6	2.6E-02
NUF2	*NUF2*, NDC80 kinetochore complex component	-1.9	2.6E-02
SDC1	syndecan 1	-2.0	2.6E-02
LMCD1	LIM and cysteine-rich domains 1	-2.0	2.6E-02
DIXDC1	DIX domain containing 1	-2.0	2.6E-02
LNX1	ligand of numb-protein X 1, E3 ubiquitin protein ligase	-3.3	2.6E-02
C18ORF22	ribosome binding factor A (putative)	-1.6	2.7E-02
LCAT	lecithin-cholesterol acyltransferase	-1.8	2.7E-02
AKR	aldo-keto reductase family 1 member B10	-2.0	2.7E-02
ENSGALG00000011233	novel gene	-3.2	2.7E-02
ZWILCH	*zwilch* kinetochore protein	-1.4	2.7E-02
FZD7	frizzled family receptor 7	-1.5	2.7E-02
SATB1	SATB homeobox 1	-1.6	2.7E-02
WBSCR17	Williams-Beuren syndrome chromosome region 17	-1.6	2.7E-02
SLC17A5	solute carrier family 17 (anion/sugar transporter), member 5	-1.6	2.7E-02
ITGB8	integrin, beta 8	-1.9	2.7E-02
ECT2	epithelial cell transforming sequence 2 oncogene	-2.1	2.7E-02
FAM83D	family with sequence similarity 83, member	-2.7	2.7E-02
SLC38A10	solute carrier family 38, member 10	-1.5	2.7E-02
KIAA1009	*KIAA1009*	-1.5	2.7E-02
MST1	macrophage stimulating 1 (hepatocyte growth factor-like)	-1.6	2.7E-02
RCOR3	REST corepressor 3	-1.5	2.7E-02
SUB1	*SUB1* homolog (S. cerevisiae)	-1.5	2.7E-02
SMARCD1	SWI/SNF related, matrix associated, actin dependent regulator of chromatin, subfamily d, member 1	-1.6	2.7E-02
ALG12	*ALG12*, alpha-1,6-mannosyltransferase	-1.6	2.7E-02
DOC2B	double C2-like domains, beta	-2.3	2.7E-02
ISCU	iron-sulfur cluster scaffold homolog (E. coli)	-1.4	2.8E-02
RNPEP	arginyl aminopeptidase (aminopeptidase B)	-1.5	2.8E-02
SLC30A7	solute carrier family 30 (zinc transporter), member 7	-1.8	2.8E-02
ANXA5	annexin A5	-1.5	2.8E-02
PPIF	peptidylprolyl isomerase F	-1.7	2.8E-02
FLRT2	fibronectin leucine rich transmembrane protein 2	-1.7	2.8E-02
BMP2K	BMP2 inducible kinase	-1.8	2.8E-02
NID1	nidogen 1	-2.3	2.8E-02
SLC9A3R2	solute carrier family 9, subfamily A (NHE3, cation proton antiporter 3), member 3 regulator 2	-1.5	2.8E-02
LSM7	*LSM7* homolog, U6 small nuclear RNA associated (S. cerevisiae)	-1.5	2.8E-02
FAM208A	family with sequence similarity 208, member A	-1.5	2.8E-02
KIAA1333	G2/M-phase specific E3 ubiquitin protein ligase	-1.7	2.8E-02
DHX33	DEAH (Asp-Glu-Ala-His) box polypeptide 33	-1.4	2.9E-02
GAS41	YEATS domain-containing protein 4	-1.4	2.9E-02
FZD2	frizzled family receptor 2	-1.6	2.9E-02
NAV2	neuron navigator 2	-1.7	2.9E-02
NCAPD3	non-SMC condensin II complex, subunit D3	-2.3	2.9E-02
GABPB2	GA binding protein transcription factor, beta subunit 2	-1.5	2.9E-02
PARN	poly(A)-specific ribonuclease	-1.5	2.9E-02
WWP1	WW domain containing E3 ubiquitin protein ligase 1	-1.5	2.9E-02
PODXL2	podocalyxin-like 2	-1.6	2.9E-02
STK38L	serine/threonine kinase 38 like	-1.7	2.9E-02
BBS7	Bardet-Biedl syndrome 7	-1.7	2.9E-02
TXNDC12	thioredoxin domain containing 12 (endoplasmic reticulum)	-1.4	2.9E-02
CHKA	choline kinase alpha	-1.5	2.9E-02

TUSC3	tumor suppressor candidate 3	-1.5	2.9E-02
DOCK10	dedicator of cytokinesis 10	-1.5	2.9E-02
HNRPLL	heterogeneous nuclear ribonucleoprotein L-like	-1.6	2.9E-02
PXDNL	peroxidasin homolog (Drosophila)-like	-1.6	2.9E-02
STARD5	StAR-related lipid transfer (START) domain containing 5	-1.6	2.9E-02
SNAPC5	small nuclear RNA activating complex, polypeptide 5, 19kDa	-1.4	3.0E-02
SPINZ	Spindlin-Z	-1.6	3.0E-02
ID1	inhibitor of DNA binding 1, dominant negative helix-loop-helix protein	-1.6	3.0E-02
RAD18	*RAD18* homolog (S. cerevisiae)	-1.8	3.0E-02
PPYR1	neuropeptide Y receptor type 4	-2.4	3.0E-02
SMARCA5	SWI/SNF related, matrix associated, actin dependent regulator of chromatin, subfamily a, member 5	-1.4	3.0E-02
MBTPS1	membrane-bound transcription factor peptidase, site 1	-1.4	3.0E-02
HIP1	huntingtin interacting protein 1	-1.5	3.0E-02
FAM69B	family with sequence similarity 69, member B	-1.6	3.0E-02
KLHL9	kelch-like family member 9	-1.4	3.1E-02
GGH	gamma-glutamyl hydrolase (conjugase, folylpolygammaglutamyl hydrolase)	-1.5	3.1E-02
MAEA	macrophage erythroblast attacher	-1.7	3.1E-02
TSC22D1	TSC22 domain family, member 1	-1.9	3.1E-02
GNB1	guanine nucleotide binding protein (G protein), beta polypeptide 1	-1.9	3.1E-02
PBLD	phenazine biosynthesis-like protein domain containing	-2.5	3.1E-02
VSIG10	V-set and immunoglobulin domain containing 10	-1.5	3.1E-02
STRADB	STE20-related kinase adaptor beta	-1.7	3.1E-02
ENSGALG00000007030	novel gene	-2.3	3.1E-02
KIRREL	kin of IRRE like (Drosophila)	-1.5	3.1E-02
WNT2B	wingless-type MMTV integration site family, member 2B	-1.9	3.1E-02
CZH5ORF42	Uncharacterized protein	-1.4	3.2E-02
EPHB2	EPH receptor B2	-1.5	3.2E-02
ANLN	anillin, actin binding protein	-1.6	3.2E-02
RFX3	regulatory factor X, 3 (influences HLA class II expression)	-1.8	3.2E-02
PANX1	pannexin 1	-1.9	3.2E-02
EIF3B	eukaryotic translation initiation factor 3, subunit B	-2.0	3.2E-02
HECW2	HECT, C2 and WW domain containing E3 ubiquitin protein ligase 2	-1.5	3.2E-02
ABCB11	ATP-binding cassette, sub-family B (MDR/TAP), member 11	-1.5	3.2E-02
CLCN4	chloride channel, voltage-sensitive 4	-1.7	3.2E-02
ANP32B	acidic (leucine-rich) nuclear phosphoprotein 32 family, member B	-1.7	3.2E-02
ENTPD2	ectonucleoside triphosphate diphosphohydrolase 2	-2.2	3.2E-02
GGA.4354	Uncharacterized protein	-2.3	3.2E-02
ENSGALG00000008518	Glutamine synthetase	-2.8	3.2E-02
MYC	v-*myc* avian myelocytomatosis viral oncogene homolog	-1.6	3.2E-02
PRRC1	proline-rich coiled-coil 1	-1.4	3.3E-02
PCDHA11	protocadherin alpha 11	-1.4	3.3E-02
OSBPL8	oxysterol binding protein-like 8	-1.5	3.3E-02
FRZB	frizzled-related protein	-1.6	3.3E-02
CTGF	connective tissue growth factor	-1.7	3.3E-02
MIPOL1	mirror-image polydactyly 1	-2.2	3.3E-02
IQGAP1	IQ motif containing GTPase activating protein 1	-1.7	3.3E-02
FBXO5	F-box protein 5	-1.7	3.3E-02
SPPL2A	signal peptide peptidase like 2A	-1.7	3.3E-02
CCDC3	coiled-coil domain containing 3	-1.7	3.3E-02
ENSGALG00000006724	novel gene	-2.3	3.3E-02
TSPAN12	tetraspanin 12	-1.5	3.3E-02
FAM171B	family with sequence similarity 171, member B	-1.5	3.3E-02
MTAP	methylthioadenosine phosphorylase	-1.7	3.3E-02
GEM	GTP binding protein overexpressed in skeletal muscle	-1.7	3.3E-02
PRG4	proteoglycan 4	-1.5	3.4E-02
ITFG2	integrin alpha FG-GAP repeat containing 2	-1.5	3.4E-02
HDDC2	HD domain containing 2	-1.5	3.4E-02

GMFB	glia maturation factor, beta	-1.7	3.4E-02
HSF3	Heat shock factor protein 3	-1.4	3.4E-02
SEC22A	SEC22 vesicle trafficking protein homolog A (S. cerevisiae)	-1.4	3.4E-02
CDCA4	cell division cycle associated 4	-1.4	3.4E-02
GPATCH2	G patch domain containing 2	-1.4	3.4E-02
AGL	amylo-alpha-1, 6-glucosidase, 4-alpha-glucanotransferase	-1.5	3.4E-02
SRD5A3	steroid 5 alpha-reductase 3	-1.5	3.4E-02
LRP8	low density lipoprotein receptor-related protein 8, apolipoprotein e receptor	-1.7	3.4E-02
PSPH	phosphoserine phosphatase	-1.9	3.4E-02
ARSH	arylsulfatase family, member H	-2.2	3.4E-02
ABCC10	ATP-binding cassette, sub-family C (CFTR/MRP), member 10	-1.4	3.4E-02
TOX	thymocyte selection-associated high mobility group box	-1.5	3.4E-02
CTSL2	cathepsin L1 precursor	-1.8	3.4E-02
NENF	neudesin neurotrophic factor	-1.4	3.4E-02
EPC2	enhancer of polycomb homolog 2 (Drosophila)	-1.4	3.4E-02
SCPEP1	serine carboxypeptidase 1	-1.4	3.4E-02
CHPKCI	histidine triad nucleotide-binding protein 1	-1.4	3.4E-02
RICTOR	RPTOR independent companion of MTOR, complex 2	-1.5	3.4E-02
FHOD3	formin homology 2 domain containing 3	-1.5	3.4E-02
POLG2	polymerase (DNA directed), gamma 2, accessory subunit	-1.5	3.4E-02
PAX-7	paired box protein *Pax-7*	-1.8	3.4E-02
C4ORF33	chromosome 4 open reading frame 33	-2.2	3.4E-02
SUPV3L1	suppressor of var1, 3-like 1 (S. cerevisiae)	-1.4	3.5E-02
ERH	enhancer of rudimentary homolog (Drosophila)	-1.4	3.5E-02
LMNB2	lamin B2	-1.5	3.5E-02
ECE-1	endothelin converting enzyme *1*	-1.4	3.5E-02
SEMA4G	sema domain, immunoglobulin domain (Ig), transmembrane domain (TM) and short cytoplasmic domain, (semaphorin) 4G	-1.4	3.5E-02
BFAR	bifunctional apoptosis regulator	-1.5	3.5E-02
IFT46	intraflagellar transport 46 homolog (Chlamydomonas)	-1.5	3.5E-02
CENPH	centromere protein H	-1.9	3.5E-02
INCENP	inner centromere protein antigens 135/155kDa	-2.2	3.5E-02
RAD21	*RAD21* homolog (S. pombe)	-1.5	3.6E-02
SLC7A6	solute carrier family 7 (amino acid transporter light chain, y+L system), member 6	-1.5	3.6E-02
KCTD18	potassium channel tetramerization domain containing 18	-1.8	3.6E-02
LEPRE1	leucine proline-enriched proteoglycan (leprecan) 1	-1.4	3.6E-02
GGA.42833	Uncharacterized protein	-1.4	3.6E-02
FDX1	ferredoxin 1	-1.5	3.6E-02
MGP	matrix Gla protein	-1.8	3.6E-02
DSCC1	DNA replication and sister chromatid cohesion 1	-2.6	3.6E-02
IRS1	insulin receptor substrate 1	-1.7	3.6E-02
KCNMA1	potassium large conductance calcium-activated channel, subfamily M, alpha member 1	-1.8	3.6E-02
SLC44A5	solute carrier family 44, member 5	-1.4	3.7E-02
ZNF827	zinc finger protein 827	-1.5	3.7E-02
ZNRF2	zinc and ring finger 2	-2.5	3.7E-02
ENSGALG00000017332	novel gene	-3.2	3.7E-02
KIF7	kinesin family member 7	-1.4	3.7E-02
ENSGALG00000028932	novel gene	-1.8	3.7E-02
NKTR	natural killer-tumor recognition sequence	-1.5	3.7E-02
UBP1	upstream binding protein 1 (LBP-1a)	-1.4	3.8E-02
SNORD14	Small nucleolar RNA *SNORD14*	/0	3.8E-02
ENSGALG00000004169	Uncharacterized protein	-1.4	3.8E-02
NUDT14	nudix (nucleoside diphosphate linked moiety X)-type motif 14	-1.4	3.8E-02
C4BPA	complement component 4 binding protein, alpha	-1.4	3.8E-02
TMEM38B	transmembrane protein 38B	-1.4	3.8E-02
TMEM132C	transmembrane protein 132C	-1.6	3.8E-02
MAK	male germ cell-associated kinase	-1.4	3.8E-02
ANKRD32	ankyrin repeat domain 32	-1.5	3.8E-02

CCNY	cyclin Y	-1.4	3.9E-02
ENSGALG00000029059	novel gene	-1.7	3.9E-02
NUP205	nucleoporin 205kDa	-1.5	3.9E-02
MKS1	Meckel syndrome, type 1	-1.5	3.9E-02
RRP8	ribosomal RNA processing 8, methyltransferase, homolog (yeast)	-2.5	3.9E-02
MSI2	musashi RNA-binding protein 2	-1.4	3.9E-02
RABL5	RAB, member RAS oncogene family-like 5	-1.5	3.9E-02
ZBTB47	zinc finger and BTB domain containing 47	-1.5	3.9E-02
ARNTL2	aryl hydrocarbon receptor nuclear translocator-like 2	-1.7	4.0E-02
WDFY1	WD repeat and FYVE domain containing 1	-1.4	4.0E-02
GALNT2	UDP-N-acetyl-alpha-D-galactosamine:polypeptide N-acetylgalactosaminyltransferase 2 (GalNAc-T2)	-1.5	4.0E-02
SYNE2	spectrin repeat containing, nuclear envelope 2	-1.5	4.0E-02
TMC6	transmembrane channel-like 6	-1.7	4.0E-02
BCL2	B-cell CLL/lymphoma 2	-1.6	4.0E-02
VSTM2L	V-set and transmembrane domain containing 2 like	-1.8	4.0E-02
ENSGALG00000016217	novel gene	-1.4	4.1E-02
PWP2	*PWP2* periodic tryptophan protein homolog (yeast)	-1.4	4.1E-02
GHOX-7	Homeobox protein *GHOX-7*	-1.5	4.1E-02
TLR21	Toll-like receptor 21 precursor	-1.6	4.1E-02
BBS5	Bardet-Biedl syndrome 5	-1.7	4.1E-02
DBR1	debranching RNA lariats 1	-1.4	4.1E-02
WIPF1	WAS/WASL interacting protein family, member 1	-1.5	4.1E-02
ENSGALG00000008803	Uncharacterized protein	-1.5	4.1E-02
TMEM18	transmembrane protein 18	-1.6	4.1E-02
MLLT3	myeloid/lymphoid or mixed-lineage leukemia (trithorax homolog, Drosophila); translocated to, 3	-1.6	4.1E-02
FIGNL1	fidgetin-like 1	-1.9	4.1E-02
HDAC2	histone deacetylase 2	-1.5	4.1E-02
CABP7	calcium binding protein 7	-1.6	4.1E-02
SH3RF3	SH3 domain containing ring finger 3	-1.7	4.1E-02
TLK1	tousled-like kinase 1	-1.4	4.2E-02
LIMS1	LIM and senescent cell antigen-like domains	-1.5	4.2E-02
TP53I11	tumor protein p53 inducible protein 11	-1.6	4.2E-02
USP43	ubiquitin specific peptidase 43	-2.8	4.2E-02
MAP4K4	mitogen-activated protein kinase kinase kinase kinase 4	-1.4	4.2E-02
ATP1B3	ATPase, Na+/K+ transporting, beta 3 polypeptide	-2.0	4.2E-02
NOC4L	nucleolar complex associated 4 homolog (S. cerevisiae)	-1.4	4.2E-02
TMEM201	transmembrane protein 201	-1.5	4.2E-02
RNASET2	ribonuclease T2	-1.5	4.2E-02
ENGASE	endo-beta-N-acetylglucosaminidase	-1.7	4.2E-02
PLK4	polo-like kinase 4	-2.0	4.2E-02
DEK	*DEK* oncogene	-1.3	4.3E-02
TOR2A	torsin family 2, member A	-1.5	4.3E-02
NSUN5	NOP2/Sun domain family, member 5	-1.5	4.3E-02
ENSGALG00000014551	Uncharacterized protein	-1.7	4.3E-02
ISM2	isthmin 2	-2.0	4.3E-02
POP1	processing of precursor 1, ribonuclease P/MRP subunit (S. cerevisiae)	-1.4	4.3E-02
HIPK1	homeodomain interacting protein kinase 1	-1.4	4.3E-02
C4H4ORF29	uncharacterized protein C4orf29 homolog	-1.5	4.3E-02
MAP4K3	mitogen-activated protein kinase kinase kinase kinase 3	-1.3	4.4E-02
ENSGALG00000027696	novel gene	-1.5	4.4E-02
SLC39A6	solute carrier family 39 (zinc transporter), member 6	-1.6	4.4E-02
CHSY1	chondroitin sulfate synthase 1	-1.3	4.4E-02
HADH	hydroxyacyl-CoA dehydrogenase	-1.5	4.4E-02
ACAD8	acyl-CoA dehydrogenase family, member 8	-1.5	4.4E-02
TNFRSF11B	tumor necrosis factor receptor superfamily, member 11b	-1.5	4.4E-02
GTSE1	G-2 and S-phase expressed 1	-2.3	4.4E-02
GPR56	G protein-coupled receptor 56	-1.5	4.4E-02
DPP6	dipeptidyl-peptidase 6	-1.4	4.5E-02
LPL	lipoprotein lipase	-1.9	4.5E-02

CALML4	calmodulin-like 4	-1.9	4.5E-02
ZCCHC8	zinc finger, CCHC domain containing 8	-1.3	4.5E-02
NASP	nuclear autoantigenic sperm protein (histone-binding)	-1.3	4.5E-02
LMF2	lipase maturation factor 2	-1.4	4.5E-02
ACSL4	acyl-CoA synthetase long-chain family member 4	-1.4	4.5E-02
ADPGK	ADP-dependent glucokinase	-1.5	4.5E-02
C5ORF30	Uncharacterized protein	-1.5	4.5E-02
FOXRED2	FAD-dependent oxidoreductase domain containing 2	-1.8	4.5E-02
SPC25	*SPC25*, NDC80 kinetochore complex component	-1.7	4.5E-02
NUDT5	nudix (nucleoside diphosphate linked moiety X)-type motif 5	-2.0	4.5E-02
NOX4	NADPH oxidase 4	-2.5	4.5E-02
QSER1	glutamine and serine rich 1	-1.3	4.6E-02
MYLK	myosin light chain kinase	-1.4	4.6E-02
LIFR	leukemia inhibitory factor receptor alpha	-1.4	4.6E-02
TMCO3	transmembrane and coiled-coil domains 3	-1.5	4.6E-02
CEP152	centrosomal protein 152kDa	-1.5	4.6E-02
GYLTL1B	glycosyltransferase-like 1B	-1.5	4.6E-02
LRIG1	leucine-rich repeats and immunoglobulin-like domains 1	-1.6	4.6E-02
NEK3	NIMA-related kinase 3	-1.8	4.6E-02
PAX-6	Paired box protein *Pax-6*	-1.9	4.6E-02
PDP2	pyruvate dehyrogenase phosphatase catalytic subunit 2	-1.6	4.6E-02
FN1	fibronectin 1	-1.5	4.6E-02
NFATC1	nuclear factor of activated T-cells, cytoplasmic, calcineurin-dependent 1	-3.4	4.6E-02
BCL10	B-cell CLL/lymphoma 10	-1.4	4.6E-02
COL4A3	collagen, type IV, alpha 3 (Goodpasture antigen)	-1.8	4.6E-02
FLVCR1	feline leukemia virus subgroup C cellular receptor 1	-2.1	4.6E-02
CREB1	cAMP responsive element binding protein 1	-1.3	4.7E-02
NUP43	nucleoporin 43kDa	-1.3	4.7E-02
ECI2	enoyl-CoA delta isomerase	-1.4	4.7E-02
MYNN	myoneurin	-1.4	4.7E-02
FBXO32	F-box protein 32	-1.6	4.7E-02
MBOAT1	membrane bound O-acyltransferase domain containing 1	-1.6	4.7E-02
ANKRD9	ankyrin repeat domain 9	-2.5	4.7E-02
TMEM237	transmembrane protein 237	-1.3	4.7E-02
EHMT1	euchromatic histone-lysine N-methyltransferase 1	-1.4	4.7E-02
PUS1	pseudouridylate synthase 1	-1.3	4.7E-02
P4HTM	prolyl 4-hydroxylase, transmembrane (endoplasmic reticulum)	-1.6	4.7E-02
KCTD14	potassium channel tetramerization domain containing 14	-1.8	4.7E-02
FEN1	flap structure-specific endonuclease 1	-1.4	4.7E-02
RASGEF1A	RasGEF domain family, member 1A	-1.6	4.7E-02
AGBL1	ATP/GTP binding protein-like 1	-1.3	4.8E-02
PRKD1	protein kinase D1	-1.5	4.8E-02
ENSGALG00000023742	novel gene	-1.7	4.8E-02
RRM1	ribonucleotide reductase M1	-1.3	4.8E-02
KIAA0556	*KIAA0556*	-1.4	4.8E-02
MTMR1	myotubularin related protein 1	-1.4	4.8E-02
CACNA2D2	calcium channel, voltage-dependent, alpha 2/delta subunit 2	-1.5	4.8E-02
CRYZL1	crystallin, zeta (quinone reductase)-like 1	-1.5	4.8E-02
CEP164	centrosomal protein 164kDa	-1.5	4.9E-02
SNORA62	small nucleolar RNA SNORA62/SNORA6 family	/0	4.9E-02
GPR162	G protein-coupled receptor 162	-1.5	4.9E-02
CHST6	carbohydrate (N-acetylglucosamine 6-O) sulfotransferase 6	-1.4	4.9E-02
DNA2	DNA replication helicase/nuclease 2	-1.6	4.9E-02
ATF4	activating transcription factor 4	-1.7	4.9E-02
ENSGALG00000015336	mature protein	-2.3	4.9E-02
MZT1	mitotic spindle organizing protein 1	-1.5	4.9E-02
QSOX1	quiescin Q6 sulfhydryl oxidase 1	-1.5	4.9E-02
METTL4	methyltransferase like 4	-2.3	4.9E-02
FBXO39	F-box protein 39	-2.7	4.9E-02
JDP2	Jun dimerization protein 2	-2.8	4.9E-02
TMEM175	transmembrane protein 175	-1.3	5.0E-02

ELMOD1	ELMO/CED-12 domain containing 1	-1.5	5.0E-02
EYA4	eyes absent homolog 4 (Drosophila)	-1.6	5.0E-02
C4ORF20	ufm1-specific protease 2	-1.4	5.0E-02
ENSGALG00000003492	smooth muscle protein phosphatase type 1-binding subunit	-1.4	5.0E-02
ST3GAL5	ST3 beta-galactoside alpha-2,3-sialyltransferase 5	-1.5	5.0E-02
MAP3K1	mitogen-activated protein kinase kinase kinase 1, E3 ubiquitin protein ligase	-1.9	5.0E-02

*p-values are corrected for multiple testing by the false discovery rate method as utilized by cuffdiff (version 2.1.1).
