# Supplementary material for: Differentiation State-Specific Mitochondrial Dynamic Regulatory Networks Are Revealed by Global Transcriptional Analysis of the Developing Chicken Lens
Source: G3 (Bethesda). 2014 Jun 13;4(8):1515–27. doi: 10.1534/g3.114.012120 (PMC4132181; doi:10.1534/g3.114.012120)
Supplement: Supporting Information [file supp_g3.114.012120_TableS4.pdf]

Table S4 Detected EQ gene-specific transcripts statistically increased in expression during EQ to FP transition.

Gene	Description	log2(Fold Change)	p-value*
RMST_8	Rhabdomyosarcoma 2 associated transcript conserved region 8	inf	1.2E-03
C7ORF53	Uncharacterized protein	inf	1.2E-03
PHF21B	PHD finger protein 21B	inf	1.2E-03
KCNE1	potassium voltage-gated channel, Isk-related family, member 1	inf	1.2E-03
RAB39A	*RAB39A*, member RAS oncogene family	inf	1.2E-03
ENSGALG00000027858	novel gene	inf	1.2E-03
ENSGALG00000005774	Na+/K+ transporting ATPase interacting 4	inf	1.2E-03
GPR160	Uncharacterized protein	inf	1.2E-03
ENSGALG00000025855	novel gene	inf	1.2E-03
PTPN5	protein tyrosine phosphatase, non-receptor type 5 (striatum-enriched)	7.8	1.2E-03
PNP	purine nucleoside phosphorylase	7.8	1.2E-03
HOPX	Homeodomain-only protein	7.4	1.2E-03
CLIC5	chloride intracellular channel 5	7.4	1.2E-03
LMO2	rhombotin-2	7.3	1.2E-03
RAB40B	Uncharacterized protein	7.2	1.2E-03
ENSGALG00000023818	novel gene	7.2	1.2E-03
BASP1	Brain acid soluble protein 1 homolog	6.9	1.2E-03
EPB49	dematin actin binding protein	6.7	1.2E-03
PTRF	polymerase I and transcript release factor	6.7	1.2E-03
IRAK4	interleukin-1 receptor-associated kinase 4	6.7	1.2E-03
FABP5	fatty acid binding protein 5 (psoriasis-associated)	6.7	1.2E-03
MFSD2B	major facilitator superfamily domain containing 2B	6.6	1.2E-03
ARHGAP20	Rho GTPase activating protein 20	6.5	1.2E-03
C12orf69	single-pass membrane protein with coiled-coil domains 3	6.5	1.2E-03
HSF4	heat shock factor protein 4	6.4	1.2E-03
ENSGALG00000028238	internexin neuronal intermediate filament protein, alpha	6.4	1.2E-03
PLEKHG4	pleckstrin homology domain containing, family G (with RhoGef domain) member 4**	6.3	1.2E-03
BFSP1	beaded filament structural protein 1, filensin	6.3	1.2E-03
PALM2	paralemmin 2	6.2	1.2E-03
PPP2R2C	protein phosphatase 2, regulatory subunit B, gamma	6.2	1.2E-03
SALL1	sal-like 1 (Drosophila)	6.2	1.2E-03
CASP7	caspase 7, apoptosis-related cysteine peptidase	6.1	1.2E-03
UPP1	uridine phosphorylase 1	6.1	1.2E-03
SASS6	spindle assembly abnormal protein 6 homolog	6.1	1.2E-03
GRIK3	glutamate receptor, ionotropic, kainate 3	6.1	1.2E-03
SPECC1	sperm antigen with calponin homology and coiled-coil domains 1	6.0	1.2E-03
GJA4	connexin 37	6.0	1.2E-03
ENSGALG00000012773	novel gene	5.9	1.2E-03
BIRC7	baculoviral IAP repeat containing 7	5.9	1.2E-03
FN3KRP	ketosamine-3-kinase	5.8	1.2E-03
TMEM47	transmembrane protein 47	5.7	1.2E-03
PKIB	protein kinase (cAMP-dependent, catalytic) inhibitor beta	5.7	1.2E-03
CTIF	CBP80/20-dependent translation initiation factor	5.7	1.2E-03
ABTB2	ankyrin repeat and BTB (POZ) domain containing 2	5.7	1.2E-03
PACSIN3	protein kinase C and casein kinase substrate in neurons 3	5.6	1.2E-03
FBLN5	fibulin 5	5.6	1.2E-03
CHRM4	muscarinic acetylcholine receptor M4	5.5	1.2E-03
STAMBPL1	STAM binding protein-like 1	5.4	1.2E-03
ATP8A2	ATPase, aminophospholipid transporter, class I, type 8A, member 2	5.4	1.2E-03
GDF10	growth differentiation factor 10	5.3	1.2E-03
HSPB8	heat shock 22kDa protein 8	5.3	1.2E-03
FAIM2	Fas apoptotic inhibitory molecule 2	5.3	1.2E-03

NT5C2	cytosolic purine 5,-nucleotidase	5.2	1.2E-03
ME1	NADP-dependent malic enzyme	5.2	1.2E-03
ESRRB	estrogen-related receptor beta	5.2	1.2E-03
PALM	paralemmin-1 isoform 2	5.2	1.2E-03
ADA	adenosine deaminase	5.2	1.2E-03
ARHGEF37	Rho guanine nucleotide exchange factor (GEF) 37	5.1	1.2E-03
ENSGALG00000001951	Uncharacterized protein	5.1	1.2E-03
NRCAM	neuronal cell adhesion molecule	5.0	1.2E-03
SCN3B	sodium channel, voltage-gated, type III, beta subunit	5.0	1.2E-03
ENSGALG00000004279	lectin, galactoside-binding, soluble, 12	5.0	1.2E-03
SCN5A	sodium channel, voltage-gated, type V, alpha subunit	4.9	1.2E-03
HEBP2	heme binding protein 2	4.9	1.2E-03
IGSF9B	immunoglobulin superfamily, member 9B	4.8	1.2E-03
LIMCH1	LIM and calponin homology domains 1	4.8	1.2E-03
UCH-L1	ubiquitin carboxyl-terminal hydrolase isozyme L1	4.7	1.2E-03
NEFL	neurofilament, light polypeptide	4.7	1.2E-03
ENC1	ectodermal-neural cortex 1 (with BTB domain)	4.7	1.2E-03
HRASLS	HRAS-like suppressor	4.7	1.2E-03
CRYBA4	beta-crystallin A4	4.7	1.2E-03
TNFAIP8	Tumor necrosis factor alpha-induced protein 8	4.7	1.2E-03
COL6A2	Collagen alpha-2(VI) chain	4.7	1.2E-03
CYYR1	cysteine/tyrosine-rich 1	4.7	1.2E-03
STARD9	StAR-related lipid transfer (START) domain containing 9	4.6	1.2E-03
RNF182	Uncharacterized protein	4.6	1.2E-03
TUBB6	Tubulin beta-5 chain	4.6	1.2E-03
NKAIN2	Na+/K+ transporting ATPase interacting 2	4.6	1.2E-03
DDHD1	DDHD domain containing 1	4.6	1.2E-03
TNFAIP8L3	tumor necrosis factor, alpha-induced protein 8-like 3	4.6	1.2E-03
MLIP	muscular LMNA-interacting protein	4.5	1.2E-03
DOK5	docking protein 5	4.5	1.2E-03
NT5DC1	5,-nucleotidase domain containing 1	4.5	1.2E-03
MYO1B	myosin IB	4.5	1.2E-03
GLRX	glutaredoxin-1	4.5	1.2E-03
CRYGN	gamma-crystallin N	4.5	1.2E-03
NPNT	nephronectin	4.5	1.2E-03
PPL	Periplakin; Uncharacterized protein	4.4	1.2E-03
ENSGALG00000022958	Uncharacterized protein	4.4	1.2E-03
MYOM2	M-protein, striated muscle	4.4	1.2E-03
PMP22	peripheral myelin protein 22	4.4	1.2E-03
HOMER2	homer homolog 2 (Drosophila)	4.4	1.2E-03
ADAMTS12	ADAM metallopeptidase with thrombospondin type 1 motif, 12	4.4	1.2E-03
SCG2	secretogranin II	4.3	1.2E-03
ACAP3	ArfGAP with coiled-coil, ankyrin repeat and PH domains 3	4.3	1.2E-03
LASP-2	nebulette non-muscle isoform	4.3	1.2E-03
CDH18	Cadherin; Uncharacterized protein	4.3	1.2E-03
WDR86	WD repeat domain 86	4.3	1.2E-03
NIF3L1	NIF3 NGG1 interacting factor 3-like 1 (S. cerevisiae)	4.3	1.2E-03
C9orf172	chromosome 9 open reading frame 172	4.3	1.2E-03
SORBS1	sorbin and SH3 domain containing 1**	4.3	1.2E-03
FAM69C	family with sequence similarity 69, member C	4.3	1.2E-03
CA10	carbonic anhydrase X	4.3	1.2E-03
RUNX2	runt-related transcription factor 2	4.3	1.2E-03
PDZD2	PDZ domain containing 2	4.2	1.2E-03
IFIH1	interferon-induced helicase C domain-containing protein 1	4.2	1.2E-03
TMEM246	transmembrane protein 246	4.2	1.2E-03
UBE2O	ubiquitin-conjugating enzyme E2O	4.2	1.2E-03
ENSGALG00000006003	Uncharacterized protein	4.2	1.2E-03
ENSGALG00000007710	Uncharacterized protein	4.2	1.2E-03
TUBB3	tubulin beta-4 chain	4.1	1.2E-03
NFKBIA	nuclear factor of kappa light polypeptide gene enhancer in B-cells inhibitor, alpha	4.0	1.2E-03
ASAP1	ArfGAP with SH3 domain, ankyrin repeat and PH domain 1	4.0	1.2E-03

LHCGR	Lutropin-choriogonadotropic hormone receptor	4.0	1.2E-03
DPYSL3	dihydropyrimidinase-related protein 3	3.9	1.2E-03
MRAS	ras-related protein *M-Ras*	3.9	1.2E-03
TRMT13	tRNA methyltransferase 13 homolog (S. cerevisiae)	3.9	1.2E-03
FIBIN	fin bud initiation factor homolog (zebrafish)	3.9	1.2E-03
MDFI	MyoD family inhibitor	3.9	1.2E-03
FAM46B	family with sequence similarity 46, member B	3.9	1.2E-03
GDPD5	glycerophosphodiester phosphodiesterase domain-containing protein 5	3.8	1.2E-03
FGF1	Fibroblast growth factor 1 Endothelial cell growth factor alpha	3.8	1.2E-03
NECAB1	N-terminal EF-hand calcium binding protein 1	3.8	1.2E-03
ENSGALG00000026188	novel gene	3.8	1.2E-03
SPHK1	sphingosine kinase 1	3.8	1.2E-03
SLC6A1	Transporter	3.8	1.2E-03
TTYH2	tweety homolog 2 (Drosophila)	3.8	1.2E-03
WDFY2	WD repeat and FYVE domain-containing protein 2	3.8	1.2E-03
DNAJB5	DnaJ (Hsp40) homolog, subfamily B, member 5	3.8	1.2E-03
RANBP3L	RAN binding protein 3-like	3.7	1.2E-03
SOX2	Transcription factor *SOX-2*	3.7	1.2E-03
SH3GL2	Endophilin-A1	3.7	1.2E-03
PIEZO2	piezo-type mechanosensitive ion channel component 2	3.7	1.2E-03
FAM159A	family with sequence similarity 159, member A	3.7	1.2E-03
DTNBP1	Dysbindin	3.7	1.2E-03
FAM222A	family with sequence similarity 222, member A	3.7	1.2E-03
NANP	N-acylneuraminate-9-phosphatase	3.6	1.2E-03
ELMO1	engulfment and cell motility protein 1	3.6	1.2E-03
DCLK2	doublecortin-like kinase 2	3.5	1.2E-03
B4GALT4	UDP-Gal:betaGlcNAc beta 1,4- galactosyltransferase, polypeptide 4	3.5	1.2E-03
NEDD9	neural precursor cell expressed, developmentally down-regulated 9	3.5	1.2E-03
PTN	pleiotrophin	3.5	1.2E-03
SDPR	serum deprivation response	3.4	1.2E-03
KALRN	kalirin, RhoGEF kinase	3.4	1.2E-03
CSDC2	cold shock domain containing C2, RNA binding	3.4	1.2E-03
HS3ST2	heparan sulfate (glucosamine) 3-O-sulfotransferase 2	3.4	1.2E-03
GRIK1	glutamate receptor, ionotropic, kainate 1	3.4	1.2E-03
TMCC3	transmembrane and coiled-coil domain family 3	3.4	1.2E-03
TSPAN15	tetraspanin-15	3.4	1.2E-03
SBDS	Ribosome maturation protein *SBDS*	3.4	1.2E-03
SBDS	Ribosome maturation protein *SBDS*	3.4	1.2E-03
ACSBG1	acyl-CoA synthetase bubblegum family member 1	3.4	1.2E-03
GSTA3	glutathione S-transferase alpha 3	3.3	1.2E-03
RFX2	regulatory factor X, 2 (influences HLA class II expression)	3.3	1.2E-03
TMCC2	transmembrane and coiled-coil domain family 2	3.3	1.2E-03
CMIP	c-Maf inducing protein	3.3	1.2E-03
PUS7L	pseudouridylate synthase 7 homolo	3.3	1.2E-03
FAM65B	protein *FAM65B*	3.3	1.2E-03
GPRC5B	G protein-coupled receptor, family C, group 5, member B	3.2	1.2E-03
FN3K	fructosamine 3 kinase	3.2	1.2E-03
LPHN3	latrophilin 3	3.2	1.2E-03
APBB1IP	amyloid beta A4 precursor protein-binding family B member 1-interacting protein	3.2	1.2E-03
RTN4IP1	reticulon 4 interacting protein 1	3.2	1.2E-03
C17ORF39	Uncharacterized protein	3.2	1.2E-03
EX-FABP	extracellular fatty acid-binding protein precursor	3.1	1.2E-03
CTNNA2	Catenin alpha-2	3.1	1.2E-03
PADI3	protein-arginine deiminase type-3	3.1	1.2E-03
NAA25	N-alpha-acetyltransferase 25, NatB auxiliary subunit	3.1	1.2E-03
ELOVL4	elongation of very long chain fatty acids protein 4	3.1	1.2E-03
CYP2H1	cytochrome P450 2H1 precursor	3.1	1.2E-03
SLC16A6	solute carrier family 16, member 6 (monocarboxylic acid	3.1	1.2E-03

	transporter 7)		
TBC1D8	TBC1 domain family, member 8 (with GRAM domain)	3.0	1.2E-03
MAPK10	mitogen-activated protein kinase 10	3.0	1.2E-03
ENSGALG00000005862	novel gene	3.0	1.2E-03
KIAA1324L	KIAA1324-like	3.0	1.2E-03
SPIRE1	spire homolog 1 (Drosophila)	3.0	1.2E-03
ENSGALG00000015366	Uncharacterized protein	2.9	1.2E-03
KIAA0226L	KIAA0226-like	2.9	1.2E-03
TMEM171	transmembrane protein 171	2.9	1.2E-03
RAI2	retinoic acid induced 2	2.9	1.2E-03
KTN1	kinectin	2.9	1.2E-03
RASGEF1B	RasGEF domain family, member 1B	2.9	1.2E-03
LRP11	low density lipoprotein receptor-related protein 11	2.9	1.2E-03
BIN1	bridging integrator 1	2.9	1.2E-03
ENSGALG00000027159	novel gene	2.9	1.2E-03
MASTL	microtubule associated serine/threonine kinase-like	2.8	1.2E-03
QRSL1	glutaminyl-tRNA synthase (glutamine-hydrolyzing)-like 1	2.8	1.2E-03
DOCK3	dedicator of cytokinesis 3	2.8	1.2E-03
MSRA	methionine sulfoxide reductase A	2.8	1.2E-03
ENSGALG00000005648	novel gene	2.8	1.2E-03
STK16	serine/threonine kinase 16	2.8	1.2E-03
GGT1	gamma-glutamyltransferase 1	2.8	1.2E-03
ME3	malic enzyme 3, NADP(+)-dependent, mitochondrial	2.8	1.2E-03
EDNRB	endothelin receptor type B precursor	2.8	1.2E-03
WNT5B	protein *Wnt-5b* precursor	2.8	1.2E-03
PCOLCE2	procollagen C-endopeptidase enhancer 2	2.7	1.2E-03
PTPN21	protein tyrosine phosphatase, non-receptor type 21	2.7	1.2E-03
ENSGALG00000020719	Uncharacterized protein	2.7	1.2E-03
ELOVL1	elongation of very long chain fatty acids protein 1	2.7	1.2E-03
TMTC2	transmembrane and tetratricopeptide repeat containing 2	2.7	1.2E-03
PDZRN4	PDZ domain containing ring finger 4	2.7	1.2E-03
ATG4B	cysteine protease *ATG4B*	2.7	1.2E-03
THSD4	thrombospondin, type I, domain containing 4	2.7	1.2E-03
HPRT1	hypoxanthine-guanine phosphoribosyltransferase	2.7	1.2E-03
WBP2	WW domain binding protein 2	2.6	1.2E-03
PAQR7	progestin and adipoQ receptor family member VII	2.6	1.2E-03
NCKIPSD	NCK interacting protein with SH3 domain	2.6	1.2E-03
ATG3	Autophagy-related protein 3	2.6	1.2E-03
ANKRD9	ankyrin repeat domain 9	2.6	1.2E-03
ENSGALG00000001166	novel gene	2.6	1.2E-03
CPEB3	cytoplasmic polyadenylation element binding protein 3	2.5	1.2E-03
ENSGALG00000005895	Uncharacterized protein	2.5	1.2E-03
MYEOV2	myeloma overexpressed 2	2.5	1.2E-03
ENSGALG00000005389	Uncharacterized protein	2.5	1.2E-03
PDE3B	cGMP-inhibited 3,,5,-cyclic phosphodiesterase B	2.5	1.2E-03
EAF2	ELL-associated factor 2	2.5	1.2E-03
PRRG3	proline rich Gla (G-carboxyglutamic acid) 3 (transmembrane)	2.5	1.2E-03
MAPK8IP2	mitogen-activated protein kinase 8 interacting protein 2	2.5	1.2E-03
EPS15L1	epidermal growth factor receptor pathway substrate 15-like 1	2.4	1.2E-03
PCSK2	proprotein convertase subtilisin/kexin type 2	2.4	1.2E-03
EZR	ezrin	2.4	1.2E-03
R3HDM2	R3H domain containing 2	2.3	1.2E-03
ENSGALG00000004032	Uncharacterized protein	2.3	1.2E-03
PIM3	*pim-3* oncogene	2.3	1.2E-03
CNP	2,,3,-cyclic nucleotide 3, phosphodiesterase	2.1	1.2E-03
PGGT1B	protein geranylgeranyltransferase type I, beta subunit	2.1	1.2E-03
LINGO1	Leucine-rich repeat and immunoglobulin-like domain-containing nogo receptor-interacting protein 1	2.1	1.2E-03
LRSAM1	leucine rich repeat and sterile alpha motif containing 1	2.1	1.2E-03
SPOCK2	sparc/osteonectin, cwcv and kazal-like domains proteoglycan (testican) 2	2.0	1.2E-03
IL2RG	interleukin 2 receptor, gamma	1.9	1.2E-03

GYPC	glycophorin C (Gerbich blood group)	5.3	2.0E-03
B3GNT5	UDP-GlcNAc:betaGal beta-1,3-N-acetylglucosaminyltransferase 5	5.2	2.0E-03
CGN	cingulin	5.2	2.0E-03
EPHB1	Ephrin type-B receptor 1	5.0	2.0E-03
RASGRP3	ras guanyl-releasing protein 3	3.6	2.0E-03
HUNK	hormonally up-regulated Neu-associated kinase	3.3	2.0E-03
SYN3	synapsin III	3.2	2.0E-03
ST8SIA4	CMP-N-acetylneuraminate-poly-alpha-2,8-sialyltransferase	3.1	2.0E-03
SLC24A3	solute carrier family 24 (sodium/potassium/calcium exchanger), member 3	2.9	2.0E-03
UBXD4	UBX domain-containing protein 2A	2.8	2.0E-03
HIGD1C	HIG1 domain family member 1A	2.8	2.0E-03
SBK1	Serine/threonine-protein kinase *SBK1*	2.6	2.0E-03
ENSGALG00000009458	novel gene	2.5	2.0E-03
RHOBTB3	Rho-related BTB domain containing 3	2.5	2.0E-03
HMX2	H6 family homeobox 2	2.4	2.0E-03
CDC20	cell division cycle protein 20 homolog	2.2	2.0E-03
SLC5A1	solute carrier family 5 (sodium/glucose cotransporter), member 1	2.2	2.0E-03
SDK1	protein sidekick-1 precursor	2.2	2.0E-03
FAR2	fatty acyl CoA reductase 2	2.2	2.0E-03
RNF146	ring finger protein 146	2.1	2.0E-03
ITGB1BP1	integrin beta 1 binding protein 1	2.1	2.0E-03
RNF151	ring finger protein 151	2.0	2.0E-03
MED10	mediator complex subunit 10	2.0	2.0E-03
FYCO1	FYVE and coiled-coil domain-containing protein 1	2.0	2.0E-03
PPRC1	peroxisome proliferator-activated receptor gamma, coactivator-related 1	1.9	2.0E-03
SAMD10	sterile alpha motif domain containing 10	6.5	2.8E-03
CP49	*CP49* protein; Uncharacterized protein	5.7	2.8E-03
GTF2A1L	TFIIA-alpha and beta-like factor	5.0	2.8E-03
PCMT1	Protein-L-isoaspartate(D-aspartate) O-methyltransferase	4.6	2.8E-03
SLC6A20	solute carrier family 6 (proline IMINO transporter), member 20	4.5	2.8E-03
ENSGALG00000025959	chromosome 3 open reading frame 83	3.4	2.8E-03
PIK3IP1	phosphoinositide-3-kinase interacting protein 1	3.1	2.8E-03
FAM189A1	family with sequence similarity 189, member A1	3.0	2.8E-03
MINPP1	multiple inositol polyphosphate phosphatase 1 precursor	3.0	2.8E-03
CAP2	Adenylyl cyclase-associated protein	2.8	2.8E-03
HSPB1	heat shock protein beta-1	2.6	2.8E-03
CHL1	cell adhesion molecule L1-like	2.6	2.8E-03
FAM126B	family with sequence similarity 126, member B	2.5	2.8E-03
INSIG1	insulin-induced gene 1 protein	2.5	2.8E-03
ASPR	substance-P receptor	2.5	2.8E-03
LIN54	*lin-54* homolog (C. elegans	2.5	2.8E-03
RPP38	ribonuclease P protein subunit p38	2.4	2.8E-03
GABRP	gamma-aminobutyric acid (GABA) A receptor, pi	2.4	2.8E-03
YWHAG	14-3-3 protein gamma	2.4	2.8E-03
BTBD3	BTB (POZ) domain containing 3	2.3	2.8E-03
GCC2	GRIP and coiled-coil domain containing 2	2.3	2.8E-03
TMEM64	transmembrane protein 64	2.3	2.8E-03
NUAK1	NUAK family, SNF1-like kinase, 1	2.2	2.8E-03
ENSGALG00000026539	novel gene	2.2	2.8E-03
UCKL1	Uridine kinase	2.1	2.8E-03
ZDHHC2	zinc finger, DHHC-type containing 2	2.0	2.8E-03
AHNAK	*AHNAK* nucleoprotein	1.9	2.8E-03
MKRN2	probable E3 ubiquitin-protein ligase makorin-2	1.9	2.8E-03
SLC39A8	solute carrier family 39 (zinc transporter), member 8	4.2	3.5E-03
DBC1	Deleted in bladder cancer protein 1 homolog	3.6	3.5E-03
GADD45	growth arrest and DNA-damage-inducible, alpha	3.2	3.5E-03
ABHD12	Monoacylglycerol lipase *ABHD12*	2.9	3.5E-03
ABLIM2	actin binding LIM protein family, member 2	2.8	3.5E-03

SBNO2	strawberry notch homolog 2 (Drosophila)	2.6	3.5E-03
MTOR	mechanistic target of rapamycin (serine/threonine kinase)	2.5	3.5E-03
ENSGALG00000002150	novel gene	2.3	3.5E-03
SDK2	protein sidekick-2	2.3	3.5E-03
WSCD1	WSC domain containing 1	2.3	3.5E-03
ZFYVE21	zinc finger, FYVE domain containing 21	2.1	3.5E-03
FAM129A	protein Niban	2.1	3.5E-03
DHRS11	Dehydrogenase/reductase SDR family member 11	2.0	3.5E-03
MLYCD	malonyl-CoA decarboxylase	2.0	3.5E-03
ENO2	gamma-enolase	2.0	3.5E-03
CEP41	Centrosomal protein of 41 kDa	2.0	3.5E-03
DHRS3	dehydrogenase/reductase (SDR family) member 3	1.9	3.5E-03
ASB1	ankyrin repeat and SOCS box containing 1	5.6	4.2E-03
CTNNA2	Catenin alpha-2	4.0	4.2E-03
CHANK1	Ankyrin 1; Uncharacterized protein	3.6	4.2E-03
BAI3	brain-specific angiogenesis inhibitor 3	3.4	4.2E-03
SCYL3	protein-associating with the carboxyl-terminal domain of ezrin	3.2	4.2E-03
KIT	Mast/stem cell growth factor receptor *Kit*	3.2	4.2E-03
RPIA	ribose-5-phosphate isomerase	2.9	4.2E-03
OTUD7B	OTU domain containing 7B	2.3	4.2E-03
PSEN2	presenilin-2	2.1	4.2E-03
SLC25A25	Uncharacterized protein	2.1	4.2E-03
MID2	Midline 2; Uncharacterized protein	1.9	4.2E-03
ST8SIA5	alpha-2,8-sialyltransferase 8E	3.7	4.8E-03
COBL	cordon-bleu WH2 repeat protein	3.4	4.8E-03
TTC9	tetratricopeptide repeat domain 9	3.2	4.8E-03
STMN4	Stathmin	2.8	4.8E-03
MAPK11	mitogen-activated protein kinase 11	2.5	4.8E-03
DYNC1I1	Uncharacterized protein	2.3	4.8E-03
DYNLL1	dynein, light chain, LC8-type 1	2.3	4.8E-03
CAV2	caveolin-2	2.2	4.8E-03
HCN2	hippocampus abundant transcript 1 protein	2.0	4.8E-03
HHLA2	HERV-H LTR-associating 2	1.9	4.8E-03
DNAH3	dynein, axonemal, heavy chain 3	1.8	4.8E-03
XIRP1	xin actin-binding repeat-containing protein 1	5.7	5.4E-03
BPGM	bisphosphoglycerate mutase	4.2	5.4E-03
PGAP2	post-GPI attachment to proteins 2	3.3	5.4E-03
FEZ1	fasciculation and elongation protein zeta 1 (zygin I)	3.2	5.4E-03
CDC34	Uncharacterized protein	3.2	5.4E-03
TLCD1	Calfacilitin	2.9	5.4E-03
SDCCAG3	serologically defined colon cancer antigen 3	2.4	5.4E-03
AMIGO2	amphoterin-induced protein 2 precursor	2.3	5.4E-03
MAP3K15	mitogen-activated protein kinase kinase kinase 15	2.3	5.4E-03
CLMN	calmin (calponin-like, transmembrane)	2.2	5.4E-03
SPON2	spondin 2, extracellular matrix protein	2.2	5.4E-03
RAD23A	RAD23 homolog A (S. cerevisiae)	2.1	5.4E-03
GGCT	gamma-glutamylcyclotransferase	2.0	5.4E-03
ENSGALG00000004078	novel gene	1.9	5.4E-03
IFRD1	interferon-related developmental regulator 1	4.4	6.0E-03
C10orf71	chromosome 10 open reading frame 71	4.0	6.0E-03
CEP104,LRRC47	leucine rich repeat containing 47	3.8	6.0E-03
KIF21A	kinesin family member 21A	2.2	6.0E-03
AGAP3	ArfGAP with GTPase domain, ankyrin repeat and PH domain 3	2.2	6.0E-03
FGFR1OP2	FGFR1 oncogene partner 2 homolog	2.0	6.0E-03
ECHDC1	enoyl CoA hydratase domain containing 1	2.0	6.0E-03
ENSGALG00000027680	novel gene	1.9	6.0E-03
STEAP3	STEAP family member 3, metalloreductase	1.8	6.0E-03
TMCO1	transmembrane and coiled-coil domains 1	1.8	6.0E-03
GLULD1	lengsin	7.5	6.5E-03
OVCH2	Uncharacterized protein	4.4	6.5E-03
LRTM2	leucine-rich repeats and transmembrane domains 2	3.3	6.5E-03

DGKB	diacylglycerol kinase, beta 90kDa	2.9	6.5E-03
KCTD20	potassium channel tetramerization domain containing 20	2.5	6.5E-03
TMEM117	transmembrane protein 117	2.4	6.5E-03
PITPNM2	phosphatidylinositol transfer protein, membrane-associated 2	2.4	6.5E-03
RTN4R	reticulon 4 receptor	2.3	6.5E-03
ARAP3	ArfGAP with RhoGAP domain, ankyrin repeat and PH domain 3	2.3	6.5E-03
AMD1	S-adenosylmethionine decarboxylase proenzyme	2.2	6.5E-03
TIPRL	TIP41, TOR signaling pathway regulator-like (S. cerevisiae)	2.2	6.5E-03
HSPH1	heat shock 105kDa	2.2	6.5E-03
THAP4	THAP domain containing 4	2.2	6.5E-03
IGBP1	immunoglobulin (CD79A) binding protein 1	2.1	6.5E-03
AKAP10	Uncharacterized protein	2.0	6.5E-03
ENSGALG00000011449	Uncharacterized protein	2.0	6.5E-03
NDUFA12	NADH dehydrogenase	1.8	6.5E-03
RANGAP1	Ran GTPase activating protein 1	4.0	7.0E-03
CXCL13L2	Chemokine	3.2	7.0E-03
NAT8L	Uncharacterized protein	3.0	7.0E-03
CAPRIN2	caprin family member 2	2.8	7.0E-03
BAG2	BCL2-associated athanogene 2	2.4	7.0E-03
GGA.46920	Uncharacterized protein	2.3	7.0E-03
LRR1	leucine rich repeat protein 1	2.3	7.0E-03
EPHA2	Uncharacterized protein	2.0	7.0E-03
GTDC2	Glycosyltransferase-like domain-containing protein 2	2.0	7.0E-03
MYO1E	myosin IE	1.7	7.0E-03
SRD5A2	steroid-5-alpha-reductase, alpha polypeptide 2 (3-oxo-5 alpha-steroid delta 4-dehydrogenase alpha 2)	3.6	7.5E-03
RBM38	RNA-binding protein 38 [	3.5	7.5E-03
ALS2CL	ALS2 C-terminal like	3.0	7.5E-03
C1QL1	complement component 1, q subcomponent-like 1	2.9	7.5E-03
FAM135B	family with sequence similarity 135, member B	2.3	7.5E-03
ENSGALG00000002955	novel gene	2.3	7.5E-03
ENSGALG00000004643	Protein-L-isoaspartate O-methyltransferase	1.9	7.5E-03
ENSGALG00000021692	novel gene	1.8	7.5E-03
ENSGALG00000026183	novel gene	1.8	7.5E-03
ZBTB17	zinc finger and BTB domain containing 17	1.7	7.5E-03
CRYBB1	Beta-crystallin B1	4.8	8.0E-03
DDB2	DNA damage-binding protein 2	4.6	8.0E-03
AHSG	alpha-2-HS-glycoprotein	4.5	8.0E-03
RANBP10	RAN binding protein 10	2.8	8.0E-03
LGALS1	16 kDa beta-galactoside-binding lectin	2.7	8.0E-03
ANKRD12	ankyrin repeat domain 12	1.8	8.0E-03
C2ORF18	Uncharacterized protein	1.7	8.0E-03
PSTPIP2	proline-serine-threonine phosphatase interacting protein 2	6.9	8.5E-03
PGM1	phosphoglucomutase-1	3.4	8.5E-03
KIF5C	kinesin family member 5C	2.2	8.5E-03
FAM129B	family with sequence similarity 129, member B	2.0	8.5E-03
SPTSSA	serine palmitoyltransferase, small subunit A	1.9	8.5E-03
SLC22A23	solute carrier family 22, member 23	1.9	8.5E-03
DPY30	protein *dpy-30* homolog	1.7	8.5E-03
CD3E	T-cell surface glycoprotein CD3 epsilon chain precursor	3.4	9.0E-03
MATN1	Cartilage matrix protein	3.0	9.0E-03
AFG3L2	AFG3 ATPase family member 3-like 2 (S. cerevisiae)	2.0	9.0E-03
EPS15	epidermal growth factor receptor substrate 15	3.4	9.5E-03
ENSGALG00000002128	Uncharacterized protein	2.8	9.5E-03
CHRNA7	neuronal acetylcholine receptor subunit alpha-7 precursor	2.8	1.0E-02
SLC25A22	mitochondrial glutamate carrier 1	5.4	1.0E-02
PENK	proenkephalin	4.7	1.0E-02
CARHSP1	calcium regulated heat stable protein 1, 24kDa	3.9	1.0E-02
FRMPD3	FERM and PDZ domain containing 3	2.7	1.0E-02
ANGIOPOIETIN-2	*angiopoietin-2*	2.5	1.0E-02
ENSGALG00000028363	AFG3 ATPase family member 3-like 2 (S. cerevisiae)	2.2	1.0E-02

SLC19A2	solute carrier family 19 (thiamine transporter), member 2	2.1	1.0E-02
RMI1	recQ-mediated genome instability protein 1	2.0	1.0E-02
ELL	RNA polymerase II elongation factor *ELL*	1.8	1.0E-02
WIPF2	WAS/WASL interacting protein family, member 2	1.6	1.0E-02
EMP2	epithelial membrane protein 2	3.0	1.1E-02
SLC28A3	solute carrier family 28 (concentrative nucleoside transporter), member 3	2.1	1.1E-02
NQO2	NAD(P)H dehydrogenase, quinone 2	1.7	1.1E-02
RFC2	Replication factor C subunit 2	1.7	1.1E-02
SH2D1B	SH2 domain containing 1B	4.9	1.1E-02
NTM	protein CEPU-1 precursor	4.4	1.1E-02
CRTAM	cytotoxic and regulatory T-cell molecule precursor	2.5	1.1E-02
EFR3B	EFR3 homolog B (S. cerevisiae)	2.5	1.1E-02
PACS2	phosphofurin acidic cluster sorting protein 2	2.3	1.1E-02
ENSGALG00000026460	Uncharacterized protein	2.2	1.1E-02
AKR1B10	aldo-keto reductase family 1, member B10 (aldose reductase)	1.9	1.1E-02
AKTIP	AKT-interacting protein	1.8	1.1E-02
ITA	Inhibitor of apoptosis protein	2.6	1.2E-02
ERAP1	endoplasmic reticulum aminopeptidase 1	2.3	1.2E-02
MAMDC4,PHPT1	phosphohistidine phosphatase 1	2.3	1.2E-02
SGMS2	sphingomyelin synthase 2	2.0	1.2E-02
TPST2	protein-tyrosine sulfotransferase 2 precursor	1.7	1.2E-02
PTTG1IP	pituitary tumor-transforming 1 interacting protein	1.6	1.2E-02
NPL	N-acetylneuraminate lyase	3.7	1.2E-02
APBA2	amyloid beta (A4) precursor protein-binding, family A, member 2	2.2	1.2E-02
SLC24A2	sodium/potassium/calcium exchanger 2	1.7	1.2E-02
TPST1	tyrosylprotein sulfotransferase 1	1.7	1.2E-02
LATS2	large tumor suppressor kinase 2	1.7	1.2E-02
TEKT3	tektin 3	3.5	1.3E-02
FGF16	fibroblast growth factor 16	3.5	1.3E-02
ART4	ecto-ADP-ribosyltransferase 4 precursor	3.4	1.3E-02
IRK1	inward rectifier potassium channel 2	3.0	1.3E-02
EPB41	Erythroid protein 4.1	2.9	1.3E-02
YWHAH	14-3-3 protein eta	2.6	1.3E-02
SYT12	synaptotagmin-12	2.3	1.3E-02
PROX2	prospero homeobox 2	2.2	1.3E-02
PSMG3	proteasome (prosome, macropain) assembly chaperone 3	1.7	1.3E-02
ESYT3	extended synaptotagmin-like protein 3	1.6	1.3E-02
SC5DL	sterol-C5-desaturase	2.8	1.3E-02
MTPN	myotrophin	2.6	1.3E-02
FAM190B	granule cell antiserum positive 14	2.3	1.3E-02
ATPAF2	ATP synthase mitochondrial F1 complex assembly factor 2	2.0	1.3E-02
DCLK1	doublecortin-like kinase 1	2.0	1.3E-02
TMEM86A	transmembrane protein 86A	3.2	1.4E-02
C10orf137	chromosome 10 open reading frame 137	2.1	1.4E-02
ATP6V0E2	ATPase, H+ transporting V0 subunit e2	1.8	1.4E-02
SLC24A6	solute carrier family 24 (sodium/lithium/calcium exchanger), member 6	1.7	1.4E-02
TP73	tumor protein p73	3.4	1.4E-02
FBXL20	F-box and leucine-rich repeat protein 20	2.8	1.4E-02
ASB9	ankyrin repeat and SOCS box protein 9	2.2	1.4E-02
RUSC1	RUN and SH3 domain containing 1	2.2	1.4E-02
SURF2	surfeit locus protein 2	1.7	1.4E-02
CORO6	coronin 6	1.6	1.4E-02
NIPA1	Uncharacterized protein	2.6	1.5E-02
GLS	glutaminase kidney isoform, mitochondrial precursor	2.3	1.5E-02
ENSGALG00000000549	urate (hydroxyiso-) hydrolase, pseudogene	2.3	1.5E-02
IGF2R	cation-independent mannose-6-phosphate receptor precursor	2.0	1.5E-02
FEM1A	Uncharacterized protein	1.9	1.5E-02
CAST	calpastatin	2.8	1.5E-02

ABCD2	ATP-binding cassette sub-family D member 2	2.3	1.5E-02
UNC13D	unc-13 homolog D (C. elegans)	2.1	1.6E-02
KIF1A	kinesin family member 1A	2.9	1.6E-02
RBM24	RNA-binding protein 24	3.5	1.6E-02
ANKIB1	ankyrin repeat and IBR domain containing 1	3.3	1.6E-02
VCL	Vinculin	1.9	1.6E-02
PAPLN	papilin, proteoglycan-like sulfated glycoprotein**	2.9	1.7E-02
METAP1	Methionine aminopeptidase 1	2.5	1.7E-02
BNIP3	BCL2/adenovirus E1B 19kDa interacting protein 3	2.1	1.7E-02
KANSL3	KAT8 regulatory NSL complex subunit 3	1.6	1.7E-02
C4ORF52	uncharacterized protein *C4orf52* homolog	1.6	1.7E-02
ENSGALG00000013848	novel gene	2.4	1.7E-02
MAP2K5	mitogen-activated protein kinase kinase 5	2.1	1.7E-02
DSG2	desmoglein 2	2.2	1.8E-02
MAP1B	Uncharacterized protein	1.8	1.8E-02
gga-mir-135a-2	*gga-mir-135a-2* [Source:miRBase;Acc:MI0001169]	inf	1.8E-02
TXNDC11	thioredoxin domain containing 11	2.9	1.8E-02
LSAMP	Limbic system-associated membrane protein	2.4	1.8E-02
SPATA2L	spermatogenesis associated 2-like	2.0	1.8E-02
STIL	SCL/TAL1 interrupting locus	1.8	1.8E-02
ST5	suppression of tumorigenicity 5	1.7	1.8E-02
SYNGR3	synaptogyrin-3	1.7	1.8E-02
ENSGALG00000023819	novel gene	6.9	1.9E-02
CELA2A	chymotrypsin-like elastase family member 2A precursor	3.0	1.9E-02
DIRAS1	DIRAS family, GTP-binding RAS-like 1	2.9	1.9E-02
ITGA4	integrin, alpha 4 (antigen CD49D, alpha 4 subunit of VLA-4 receptor)	2.2	1.9E-02
LMTK2	lemur tyrosine kinase 2	2.0	1.9E-02
C18ORF8	Uncharacterized protein	3.8	1.9E-02
FRYL	FRY-like	2.8	1.9E-02
KLF3	Kruppel-like factor 3 (basic)	2.0	1.9E-02
SYNPR	synaptoporin precursor	1.9	1.9E-02
STAT4	signal transducer and activator of transcription 4	4.1	1.9E-02
UBTD2	Uncharacterized protein	3.2	1.9E-02
ARFGAP3	ADP-ribosylation factor GTPase-activating protein 3	3.0	1.9E-02
RAPGEF6	Rap guanine nucleotide exchange factor (GEF) 6	2.4	1.9E-02
TWF1	twinfilin 1	2.0	1.9E-02
ROBO1	roundabout, axon guidance receptor, homolog 1	1.7	1.9E-02
GAB3	GRB2-associated binding protein 3	2.6	2.0E-02
CHODL	chondrolectin	4.2	2.0E-02
TBC1D14	TBC1 domain family member 14	1.8	2.0E-02
RAP2A	Uncharacterized protein	2.1	2.0E-02
F13A1	coagulation factor XIII A chain	1.9	2.0E-02
CACFD1	calcium channel flower domain containing 1	1.7	2.0E-02
RPE	ribulose-5-phosphate-3-epimerase	1.6	2.0E-02
PVALB	Parvalbumin, thymic	5.9	2.1E-02
C1orf43	chromosome 1 open reading frame 43	1.9	2.1E-02
ENSGALG00000003444	novel gene	1.8	2.1E-02
CZH18ORF25	Uncharacterized protein	1.8	2.1E-02
ENSGALG00000026110	SH3 and multiple ankyrin repeat domains 3	1.7	2.1E-02
NKIRAS1	NFKB inhibitor interacting Ras-like 1	1.5	2.1E-02
C2CD2	C2 calcium-dependent domain containing 2	2.0	2.1E-02
FAM100A	UBA-like domain containing 1	1.5	2.1E-02
GLI1	Zinc finger protein *GLI1*	2.5	2.2E-02
ENSGALG00000011324	novel gene	2.5	2.2E-02
CYP2D6	cytochrome P450, family 2, subfamily D, polypeptide 6	2.0	2.2E-02
ABCC3	ATP-binding cassette, sub-family C (CFTR/MRP), member 3	1.8	2.2E-02
CCM2	malcavernin	1.8	2.2E-02
FAM54B	Uncharacterized protein	1.6	2.2E-02
MED19	mediator complex subunit 19	1.5	2.2E-02
ENSGALG00000026793	novel gene	3.9	2.2E-02
ENSGALG00000025721	cytochrome P450, family 2, subfamily D, polypeptide 7 pseudogene 1	3.9	2.2E-02

UBE3B	Ubiquitin protein ligase; Uncharacterized protein	1.8	2.2E-02
OSBP2	oxysterol binding protein 2	1.5	2.2E-02
SLC38A7	solute carrier family 38, member 7	1.5	2.2E-02
FAM212B	family with sequence similarity 212, member B	3.5	2.2E-02
WDTC1	Uncharacterized protein	3.0	2.2E-02
41341	Translationally controlled tumor protein**	1.8	2.2E-02
ENSGALG00000015655	novel gene	5.9	2.3E-02
KCNK1	potassium channel, subfamily K, member 1	2.1	2.3E-02
CREBL2	cAMP responsive element binding protein-like 2	1.8	2.3E-02
ABTB1	ankyrin repeat and BTB/POZ domain-containing protein 1	1.8	2.3E-02
TRNT1	tRNA nucleotidyl transferase, CCA-adding, 1	1.7	2.3E-02
SNCG1	synuclein, gamma	2.5	2.3E-02
PBDC1	polysaccharide biosynthesis domain containing 1	2.0	2.3E-02
LRRK2	leucine-rich repeat kinase 2	1.8	2.3E-02
DOCK5	dedicator of cytokinesis 5	2.1	2.3E-02
GNPAT	glyceronephosphate O-acyltransferase	1.7	2.3E-02
TMEM123	transmembrane protein 123 precursor	1.7	2.3E-02
SIAH2	siah E3 ubiquitin protein ligase 2	1.6	2.4E-02
RPS6KA	Ribosomal protein S6 kinase 2 alpha	1.7	2.4E-02
MFSD6	Uncharacterized protein	1.6	2.4E-02
SEPX1	selenoprotein X, 1	1.4	2.4E-02
SPTAN1	spectrin alpha chain, brain	2.8	2.4E-02
PRKAB2	5,-AMP-activated protein kinase subunit beta-2	1.5	2.4E-02
UNC45B	unc-45 homolog B (C. elegans)	3.6	2.5E-02
ST18	suppression of tumorigenicity 18 (breast carcinoma) (zinc finger protein)	3.5	2.5E-02
ENSGALG00000009935	novel gene	2.1	2.5E-02
PHLDA2	pleckstrin homology-like domain family A member 2	1.9	2.5E-02
HSD17B4	peroxisomal multifunctional enzyme type 2	1.9	2.5E-02
ITSN2	intersectin 2	1.7	2.5E-02
CIT	citron (rho-interacting, serine/threonine kinase 21)	1.7	2.5E-02
NR2F1	nuclear receptor subfamily 2, group F, member 1	2.6	2.5E-02
CRCP	CGRP receptor component	2.4	2.5E-02
GPX1	glutathione peroxidase 1	2.3	2.5E-02
LDB3	LIM domain binding 3	2.1	2.5E-02
HECTD4	HECT domain containing E3 ubiquitin protein ligase 4	1.8	2.5E-02
OAF	Out at first protein homolog	1.8	2.5E-02
CYP39A1	cytochrome P450, family 39, subfamily A, polypeptide 1	1.5	2.5E-02
FBXW7	F-box and WD repeat domain containing 7, E3 ubiquitin protein ligase	1.4	2.5E-02
PRICKLE1	prickle homolog 1 (Drosophila)	1.9	2.6E-02
NDUFS5	NADH dehydrogenase (ubiquinone) Fe-S protein 5, 15kDa (NADH-coenzyme Q reductase)	1.7	2.6E-02
GGA.41926	Uncharacterized protein	1.7	2.6E-02
TIE1	tyrosine kinase with immunoglobulin-like and EGF-like domains 1	3.4	2.6E-02
FAM49B	family with sequence similarity 49, member B	2.1	2.6E-02
STAC	SH3 and cysteine rich domain	2.9	2.6E-02
EAF1	ELL associated factor 1	2.1	2.6E-02
ARHGEF10	Rho guanine nucleotide exchange factor (GEF) 10	2.1	2.7E-02
HSP70	heat shock 70 kDa protein	2.1	2.7E-02
CD5	T-cell surface glycoprotein *CD5* precursor	3.5	2.7E-02
CEPT1	choline/ethanolaminephosphotransferase 1	2.1	2.7E-02
SPG7	spastic paraplegia 7	1.8	2.7E-02
DENND4A	DENN/MADD domain containing 4A	1.6	2.7E-02
PTDSS1	Phosphatidylserine synthase 1	2.3	2.7E-02
ADCY5	adenylate cyclase type 5	2.1	2.7E-02
UBOX5	U-box domain containing 5	1.5	2.8E-02
ENSGALG00000023424	novel gene	2.0	2.8E-02
HABP4	Intracellular hyaluronan-binding protein 4	1.4	2.8E-02
SPTBN1	spectrin beta chain, brain 1	2.8	2.8E-02
PLEKHA1	pleckstrin homology domain containing, family A (phosphoinositide binding specific) member 1	1.9	2.9E-02

ZNF511	zinc finger protein 511	1.6	2.9E-02
KDM5A	lysine (K)-specific demethylase 5A	1.6	2.9E-02
HBS1L	HBS1-like (S. cerevisiae)	2.0	2.9E-02
CDK6	cell division protein kinase 6	1.5	3.0E-02
SLC37A2	solute carrier family 37 (glucose-6-phosphate transporter), member 2	1.5	3.0E-02
SNX20	sorting nexin 20	1.9	3.0E-02
SH3BGRL2	SH3 domain-binding glutamic acid-rich-like protein	5.4	3.0E-02
SUSD5	sushi domain containing 5	4.1	3.1E-02
ENSGALG00000027412	novel gene	2.3	3.1E-02
HINT3	histidine triad nucleotide binding protein 3	2.0	3.1E-02
TNRC6B	trinucleotide repeat containing 6B	2.6	3.1E-02
RAB7L1	RAB7, member RAS oncogene family-like 1	2.6	3.1E-02
RASSF2	ras association domain-containing protein 2	2.1	3.1E-02
HPSE	heparanase precursor	4.6	3.2E-02
TDRD7	Tudor domain-containing protein 7	2.4	3.2E-02
DCN	decorin precurso	2.3	3.2E-02
SCD	stearoyl-CoA desaturase 1	2.1	3.2E-02
FAM213A	Redox-regulatory protein *FAM213A*	1.9	3.2E-02
NT5C3	Cytosolic 5,-nucleotidase III	1.7	3.2E-02
SNTG1	syntrophin, gamma 1	1.5	3.2E-02
PDZD11	PDZ domain-containing protein 11	1.4	3.2E-02
CSDA	Y box binding protein 3	2.8	3.2E-02
GGA.17220,TOM1L2	Uncharacterized protein	2.0	3.2E-02
FDPS,NUP210L	nucleoporin 210kDa-like	1.7	3.2E-02
AKAP5	A kinase (PRKA) anchor protein 5	2.4	3.2E-02
DFFA	DNA fragmentation factor, 45kDa, alpha polypeptide	2.3	3.2E-02
C1ORF114	Uncharacterized protein	2.2	3.2E-02
KIAA0319L	KIAA0319-like	1.5	3.2E-02
PDHX	pyruvate dehydrogenase complex, component X	1.4	3.2E-02
UACA	uveal autoantigen with coiled-coil domains and ankyrin repeats	1.9	3.3E-02
ATP11C	Uncharacterized protein	1.7	3.3E-02
SFXN2	Uncharacterized protein	1.4	3.3E-02
ENSGALG00000015928	novel gene	2.8	3.4E-02
AANAT	Serotonin N-acetyltransferase	1.6	3.4E-02
MED8	Uncharacterized protein	1.5	3.4E-02
ATP6V0A1	V-type proton ATPase 116 kDa subunit a isoform 1	1.5	3.4E-02
HEATR5A	HEAT repeat containing 5A	1.5	3.4E-02
MED9	mediator complex subunit 9	1.4	3.4E-02
DGKQ	diacylglycerol kinase, theta 110kDa	1.5	3.4E-02
CDKL2	cyclin-dependent kinase-like 2 (CDC2-related kinase)	2.9	3.4E-02
SERPINE2	serpin peptidase inhibitor, clade E (nexin, plasminogen activator inhibitor type 1), member 2	1.7	3.4E-02
GATAD1	GATA zinc finger domain containing 1	1.5	3.4E-02
PRSS23	protease, serine, 23	3.3	3.5E-02
KIAA1522	*KIAA1522*	2.0	3.5E-02
SLC22A15	solute carrier family 22, member 15	2.0	3.5E-02
ZNF628	zinc finger protein 628	1.7	3.5E-02
PRPSAP1	phosphoribosyl pyrophosphate synthetase-associated protein 1	2.2	3.5E-02
UBE2G2	ubiquitin-conjugating enzyme E2G 2	2.2	3.6E-02
ENSGALG00000011528	novel gene	1.7	3.6E-02
ZDHHC8	zinc finger, DHHC-type containing 8	1.6	3.6E-02
CMC4	C-x(9)-C motif containing 4 homolog (S. cerevisiae)	1.4	3.6E-02
SNORD37	small nucleolar RNA, C/D box 37	0.2	3.6E-02
LCA5L	Leber congenital amaurosis 5-like	5.9	3.6E-02
EPB41L1	erythrocyte membrane protein band 4.1-like 1	2.1	3.6E-02
DDHD2	DDHD domain containing 2	2.0	3.6E-02
WNK1	WNK lysine deficient protein kinase 1	1.9	3.6E-02
PNRC1	proline-rich nuclear receptor coactivator 1	2.7	3.7E-02
DNAAF2	dynein, axonemal, assembly factor 2	1.7	3.7E-02
MRPS7	mitochondrial ribosomal protein S7	2.1	3.8E-02

CLTB	clathrin, light chain B	2.1	3.8E-02
TACC1	transforming, acidic coiled-coil containing protein 1	1.5	3.8E-02
C10ORF11	chromosome 10 open reading frame 11	1.7	3.9E-02
ZFYVE28	zinc finger, FYVE domain containing 28	1.5	4.0E-02
STAT5	signal transducer and activator of transcription 5A	2.1	4.0E-02
RARRES1	retinoic acid receptor responder (tazarotene induced) 1	2.3	4.0E-02
ENSGALG00000027645	novel gene	1.5	4.0E-02
SRSF4	serine/arginine-rich splicing factor 4	1.4	4.0E-02
ASTL	astacin-like metallo-endopeptidase (M12 family)	2.0	4.1E-02
RXRG	retinoid X receptor, gamma	1.8	4.1E-02
LSM12	*LSM12* homolog (S. cerevisiae)	1.4	4.1E-02
LYSMD2	LysM, putative peptidoglycan-binding, domain containing 2	1.4	4.1E-02
SEC31B	SEC31 homolog B (S. cerevisiae)	1.4	4.1E-02
PRKAR2B	protein kinase, cAMP-dependent, regulatory, type II, beta	3.3	4.1E-02
NELL2	NEL-like 2	1.7	4.1E-02
DNAJC15	DnaJ (Hsp40) homolog, subfamily C, member 15	1.4	4.1E-02
MRS2	*MRS2* magnesium transporter	1.3	4.1E-02
SNX18	sorting nexin 18	1.4	4.2E-02
IQGAP2	IQ motif containing GTPase activating protein 2	1.5	4.2E-02
ENSGALG00000006897	novel gene	1.7	4.2E-02
ANGEL1	angel homolog 1 (Drosophila)	1.8	4.3E-02
ATP6V1D	ATPase, H+ transporting, lysosomal 34kDa, V1 subunit D	1.8	4.3E-02
CISD3	CDGSH iron sulfur domain 3	1.6	4.3E-02
CCDC67	coiled-coil domain containing 67	6.8	4.3E-02
SNPH	syntaphilin	5.4	4.3E-02
COPS4	COP9 signalosome subunit 4	1.6	4.3E-02
BTAF1	*BTAF1* RNA polymerase II, B-TFIID transcription factor-associated, 170kDa	1.6	4.3E-02
BTBD10	BTB (POZ) domain containing 10	3.0	4.4E-02
ENSGALG00000026480	novel gene	1.6	4.4E-02
RPL3L	ribosomal protein L3-like	3.2	4.4E-02
TESC	tescalcin	2.2	4.4E-02
PYGL	phosphorylase, glycogen, liver	1.6	4.4E-02
RPS6KL1	ribosomal protein S6 kinase-like 1	1.4	4.4E-02
HCN3	hyperpolarization activated cyclic nucleotide-gated potassium channel 3	3.1	4.4E-02
FGF22	fibroblast growth factor 22	2.7	4.5E-02
CLIP1	CAP-GLY domain containing linker protein 1	1.8	4.5E-02
FAM188B2	family with sequence similarity 188, member B2	2.8	4.5E-02
NDUFB10	NADH dehydrogenase (ubiquinone) 1 beta subcomplex, 10, 22kDa	1.7	4.5E-02
SPG20	spastic paraplegia 20 (Troyer syndrome)	1.7	4.5E-02
SFXN1	sideroflexin 1	1.7	4.5E-02
SH3BGRL3	SH3 domain binding glutamic acid-rich protein like 3	1.4	4.5E-02
C8ORF22	chromosome 8 open reading frame 22	2.3	4.6E-02
FAM162A	family with sequence similarity 162, member A	2.0	4.6E-02
PNPLA7	patatin-like phospholipase domain containing 7	1.8	4.6E-02
ENSGALG00000020592	novel gene	3.6	4.6E-02
ENSGALG00000026680	novel gene	2.9	4.6E-02
RILPL1	Rab interacting lysosomal protein-like 1	1.9	4.6E-02
C7ORF25	chromosome 7 open reading frame 25	1.4	4.7E-02
DEPDC7,TCP11L1	t-complex 11, testis-specific-like 1	2.5	4.7E-02
FAM20A	family with sequence similarity 20, member A	2.0	4.7E-02
IGSF21	immunoglobin superfamily, member 21	1.7	4.7E-02
RCAN2	regulator of calcineurin 2	2.0	4.8E-02
ETV4	ets variant 4	1.6	4.8E-02
F5	coagulation factor V (proaccelerin, labile factor)	1.3	4.8E-02
GABRG4	Gamma-aminobutyric acid receptor subunit gamma-4	1.7	4.8E-02
CLPX	*ClpX* caseinolytic peptidase X homolog (E. coli)	1.5	4.8E-02
ALAS1	aminolevulinate, delta-, synthase 1	1.5	4.9E-02
SPTY2D1	SPT2, Suppressor of Ty, domain containing 1 (S. cerevisiae)	1.4	4.9E-02
MPV17L2	MPV17 mitochondrial membrane protein-like 2	1.3	4.9E-02
PPIL6	peptidylprolyl isomerase (cyclophilin)-like 6	3.3	5.0E-02

ZNF503	zinc finger protein 503	3.1	5.0E-02
HMOX1	heme oxygenase (decycling) 1	2.8	5.0E-02

*p-values are corrected for multiple testing by the false discovery rate method as utilized by cuffdiff (version 2.1.1).
