# Supplementary material for: Differentiation State-Specific Mitochondrial Dynamic Regulatory Networks Are Revealed by Global Transcriptional Analysis of the Developing Chicken Lens
Source: G3 (Bethesda). 2014 Jun 13;4(8):1515–27. doi: 10.1534/g3.114.012120 (PMC4132181; doi:10.1534/g3.114.012120)
Supplement: Supporting Information [file supp_g3.114.012120_TableS5.pdf]

Table S5 Detected FP gene-specific transcripts statistically decreased in expression during FP to FC transition.

Gene	Description	log2(Fold Change)	p-value*
ENSGALG00000007692	Uncharacterized protein	/0	1.2E-03
CGN	cingulin	-5.0	6.0E-03
ENSGALG00000026793	novel gene	-3.1	6.5E-03
R3HDML	R3H domain containing-like	-2.0	8.0E-03
SNORD37	Small nucleolar RNA *SNORD37*	/0	1.6E-02
ENSGALG00000025721	cytochrome P450, family 2, subfamily D, polypeptide 7 pseudogene 1	-2.0	2.2E-02
PENK	proenkephalin	-1.9	2.7E-02
ENSGALG00000025721	cytochrome P450, family 2, subfamily D, polypeptide 7 pseudogene 1	/0	3.1E-02
snoU83B	Small nucleolar RNA U83B	/0	4.3E-02
ENSGALG00000026680	novel gene	-2.0	4.8E-02

*p-values are corrected for multiple testing by the false discovery rate method as utilized by cuffdiff (version 2.1.1).
