# Supplementary material for: Differentiation State-Specific Mitochondrial Dynamic Regulatory Networks Are Revealed by Global Transcriptional Analysis of the Developing Chicken Lens
Source: G3 (Bethesda). 2014 Jun 13;4(8):1515–27. doi: 10.1534/g3.114.012120 (PMC4132181; doi:10.1534/g3.114.012120)
Supplement: Supporting Information [file supp_g3.114.012120_TableS6.pdf]

Table S6 Detected FP gene-specific transcripts statistically increased in expression during FP to FC transition.

Gene	Description	log2(Fold Change)	p-value*
gga-mir-10a	*gga-mir-10a* [Source:miRBase;Acc:MI0007559]	inf	1.2E-03
C10ORF47	Uncharacterized protein	3.4	4.2E-03
SLC35E4	solute carrier family 35, member E4	inf	4.8E-03
TRAIL-LIKE	TNF-related apoptosis inducing ligand-*like* protein precursor	2.7	4.8E-03
ENSGALG00000010944	novel gene	2.3	1.8E-02
ENSGALG00000028414	novel gene	2.8	1.8E-02
LONRF2	LON peptidase N-terminal domain and ring finger 2	2.8	2.6E-02
ENSGALG00000026665	novel gene	1.1	4.2E-02
NUPL2	nucleoporin-like protein 2	2.0	4.6E-02
gga-mir-1618	*gga-mir-1618* [Source:miRBase;Acc:MI0007347]	inf	4.7E-02
ENSGALG00000011233	novel gene	2.6	4.9E-02

*p-values are corrected for multiple testing by the false discovery rate method as utilized by cuffdiff (version 2.1.1).
