# Supplementary material for: Differentiation State-Specific Mitochondrial Dynamic Regulatory Networks Are Revealed by Global Transcriptional Analysis of the Developing Chicken Lens
Source: G3 (Bethesda). 2014 Jun 13;4(8):1515–27. doi: 10.1534/g3.114.012120 (PMC4132181; doi:10.1534/g3.114.012120)
Supplement: Supporting Information [file supp_g3.114.012120_TableS7.pdf]

Table S7 Nuclear encoded mitochondrial protein transcript that demonstrated a two-fold decrease in expression or greater during EC to EQ transition. Detected FPKM and fold change (Δ) shown.

Symbol	EC	EQ	Δ	Description
**LDHA**	4469.11	771.07	-5.80	lactate dehydrogenase A
**HK2**	21.34	5.45	-3.92	hexokinase 2
**ACSL4**	50.55	13.05	-3.87	acyl-CoA synthetase long-chain family member 4
**GATM**	0.36	0.09	-3.78	glycine amidinotransferase (L-arginine:glycine amidinotransferase)
**PRSS35**	12.87	3.41	-3.77	protease, serine, 35
**TAP1**	1.36	0.39	-3.52	transporter 1, ATP-binding cassette, sub-family B (MDR/TAP)
**SDSL**	1.80	0.52	-3.46	serine dehydratase-like
**HAO2**	0.56	0.18	-3.22	hydroxyacid oxidase 2 (long chain)
**OGG1**	10.88	3.39	-3.20	8-oxoguanine DNA glycosylase
**CKMT1A**	1.37	0.44	-3.14	creatine kinase, mitochondrial 1A
**TDRKH**	0.48	0.16	-3.09	tudor and KH domain containing
**AIFM3**	1.96	0.67	-2.92	apoptosis-inducing factor, mitochondrion-associated, 3
**ARG2**	159.12	58.90	-2.70	arginase, type II
**ELN**	0.75	0.31	-2.46	elastin (supravalvular aortic stenosis, Williams-Beuren syndrome)
**CYP11A1**	1.53	0.62	-2.44	cytochrome P450, family 11, subfamily A, polypeptide 1
**SLC25A29**	6.60	2.80	-2.36	solute carrier family 25, member 29
**ARMC4**	0.26	0.12	-2.23	armadillo repeat containing 4
**SLC25A37**	2.82	1.35	-2.10	solute carrier family 25, member 37
**SNPH**	0.75	0.36	-2.09	syntaphilin
**COQ9**	9.49	4.66	-2.04	coenzyme Q9 homolog (S. cerevisiae)
**MUTYH**	2.93	1.45	-2.03	mutY homolog (E. coli)
