# Supplementary material for: Differentiation State-Specific Mitochondrial Dynamic Regulatory Networks Are Revealed by Global Transcriptional Analysis of the Developing Chicken Lens
Source: G3 (Bethesda). 2014 Jun 13;4(8):1515–27. doi: 10.1534/g3.114.012120 (PMC4132181; doi:10.1534/g3.114.012120)
Supplement: Supporting Information [file supp_g3.114.012120_TableS8.pdf]

Table S8 Nuclear encoded mitochondrial protein transcript that demonstrated a two-fold increase in expression or greater during EC to EQ transition. Detected FPKM and fold change (Δ) shown.

Symbol	EC	EQ	Δ	Description
**NME4**	0.22	1.19	5.34	non-metastatic cells 4, protein expressed in
**GPAM**	2.81	9.54	3.40	glycerol-3-phosphate acyltransferase, mitochondrial
**MRPL39**	10.44	34.55	3.31	mitochondrial ribosomal protein L39
**BCKDHB**	4.73	14.00	2.96	branched chain keto acid dehydrogenase E1, beta polypeptide (maple syrup urine disease)
**BBOX1**	1.50	3.92	2.61	butyrobetaine (gamma), 2-oxoglutarate dioxygenase (gamma-butyrobetaine hydroxylase) 1
**AGR2**	0.23	0.60	2.59	anterior gradient 2 homolog (Xenopus laevis)
**TSHZ3**	0.74	1.89	2.56	teashirt family zinc finger 3
**ATP10D**	1.54	3.92	2.54	ATPase, Class V, type 10D
**IDH1**	53.10	130.93	2.47	isocitrate dehydrogenase 1 (NADP+), soluble
**NCOA4**	21.37	52.42	2.45	nuclear receptor coactivator 4
**SQRDL**	1.76	4.24	2.41	sulfide quinone reductase-like (yeast)
**TOMM70A**	23.57	56.63	2.40	translocase of outer mitochondrial membrane 70 homolog A (S. cerevisiae)
**LYRM1**	10.58	25.30	2.39	LYR motif containing 1
**AK2**	23.62	56.27	2.38	adenylate kinase 2
**MAOA**	2.63	6.20	2.35	monoamine oxidase A
**SLC25A38**	1.46	3.36	2.30	solute carrier family 25, member 38
**IDE**	10.66	24.40	2.29	insulin-degrading enzyme
**ALDH2**	22.08	49.66	2.25	aldehyde dehydrogenase 2 family (mitochondrial)
**MRPS14**	8.95	19.85	2.22	mitochondrial ribosomal protein S14
**GLRX**	9.47	20.81	2.20	glutaredoxin (thioltransferase)
**GTPBP5**	4.41	9.55	2.16	GTP binding protein 5 (putative)
**ABHD10**	2.65	5.71	2.16	abhydrolase domain containing 10
**KYNU**	0.46	0.98	2.15	kynureninase (L-kynurenine hydrolase)
**MCAT**	14.46	30.59	2.12	malonyl CoA:ACP acyltransferase (mitochondrial)
**COMTD1**	2.64	5.45	2.06	catechol-O-methyltransferase domain containing 1
**TIMM23**	13.74	28.27	2.06	translocase of inner mitochondrial membrane 23 homolog (yeast)
**DUSP26**	0.74	1.51	2.04	dual specificity phosphatase 26 (putative)
**HMGCS2**	0.65	1.33	2.04	3-hydroxy-3-methylglutaryl-Coenzyme A synthase 2 (mitochondrial)
**MTX2**	26.46	53.22	2.01	metaxin 2
**NDUFA10**	40.07	80.42	2.01	NADH dehydrogenase (ubiquinone) 1 alpha subcomplex, 10, 42kDa
**NDUFA4**	93.79	187.91	2.00	NADH dehydrogenase (ubiquinone) 1 alpha subcomplex, 4, 9kDa
