# Supplementary material for: Differentiation State-Specific Mitochondrial Dynamic Regulatory Networks Are Revealed by Global Transcriptional Analysis of the Developing Chicken Lens
Source: G3 (Bethesda). 2014 Jun 13;4(8):1515–27. doi: 10.1534/g3.114.012120 (PMC4132181; doi:10.1534/g3.114.012120)
Supplement: Supporting Information [file supp_g3.114.012120_TableS9.pdf]

Table S9 Nuclear encoded mitochondrial protein transcript that demonstrated a two-fold decrease in expression or greater during EQ to FP transition. Detected FPKM and fold change (Δ) shown.

Symbol	EQ	FP	Δ	Description
**MAOA**	6.2	0.6	-10.2	monoamine oxidase A
**SLC16A1**	210.8	25.8	-8.2	solute carrier family 16, member 1 (monocarboxylic acid transporter 1)
**SARDH**	15.9	2.1	-7.5	sarcosine dehydrogenase
**AGMAT**	0.8	0.1	-7.1	agmatine ureohydrolase (agmatinase)
**PDK1**	12.6	1.8	-6.9	pyruvate dehydrogenase kinase, isozyme 1
**TXNRD1**	2.8	0.4	-6.6	thioredoxin reductase 1
**ARG2**	58.9	9.0	-6.5	arginase, type II
**EFHD1**	5.9	1.0	-6.2	EF-hand domain family, member D1
**ATP10D**	3.9	0.7	-5.6	ATPase, Class V, type 10D
**DUT**	10.4	1.9	-5.6	dUTP pyrophosphatase
**ALDH18A1**	12.9	2.6	-5.0	aldehyde dehydrogenase 18 family, member A1
**PHYHIPL**	3.9	0.8	-4.7	phytanoyl-CoA 2-hydroxylase interacting protein-like
**LACTB2**	5.2	1.1	-4.6	lactamase, beta 2
**TSHZ3**	1.9	0.4	-4.5	teashirt family zinc finger 3
**CERK**	11.1	2.5	-4.4	ceramide kinase
**CRY1**	22.8	5.3	-4.3	cryptochrome 1 (photolyase-like)
**OMA1**	5.4	1.3	-4.2	OMA1 homolog, zinc metallopeptidase (S. cerevisiae)
**ACSL1**	9.0	2.2	-4.0	acyl-CoA synthetase long-chain family member 1
**AS3MT**	19.8	5.0	-3.9	arsenic (+3 oxidation state) methyltransferase
**NME4**	1.2	0.3	-3.8	non-metastatic cells 4, protein expressed in
**SLC25A3**	395.5	104.9	-3.8	solute carrier family 25 (mitochondrial carrier; phosphate carrier), member 3
**UNG**	10.7	2.9	-3.7	uracil-DNA glycosylase
**POLG**	4.3	1.2	-3.7	polymerase (DNA directed), gamma
**BCKDHB**	14.0	3.9	-3.6	branched chain keto acid dehydrogenase E1, beta polypeptide (maple syrup urine disease)
**CYP27A1**	6.2	1.8	-3.4	cytochrome P450, family 27, subfamily A, polypeptide 1
**EHHADH**	7.7	2.2	-3.4	enoyl-Coenzyme A, hydratase/3-hydroxyacyl Coenzyme A dehydrogenase
**EARS2**	2.4	0.7	-3.4	glutamyl-tRNA synthetase 2 (mitochondrial)(putative)
**DDAH1**	54.9	16.4	-3.3	dimethylarginine dimethylaminohydrolase 1
**BID**	9.7	2.9	-3.3	BH3 interacting domain death agonist
**FARS2**	20.7	6.4	-3.2	phenylalanine-tRNA synthetase 2 (mitochondrial)
**ALDH4A1**	7.4	2.3	-3.2	aldehyde dehydrogenase 4 family, member A1
**ALDH5A1**	7.4	2.3	-3.2	aldehyde dehydrogenase 5 family, member A1 (succinate-semialdehyde dehydrogenase)
**PPIF**	5.3	1.7	-3.1	peptidylprolyl isomerase F (cyclophilin F)
**GALC**	1.7	0.5	-3.1	galactosylceramidase
**SLC25A6**	294.3	94.2	-3.1	solute carrier family 25 (mitochondrial carrier; adenine nucleotide translocator), member 6
**PPM1K**	8.7	2.8	-3.1	protein phosphatase 1K (PP2C domain containing)
**SLC25A12**	9.9	3.3	-3.0	solute carrier family 25 (mitochondrial carrier, Aralar), member 12
**PDP2**	6.6	2.2	-3.0	pyruvate dehydrogenase phosphatase isoenzyme 2

**SLC25A13**	14.0	4.7	-3.0	solute carrier family 25, member 13 (citrin)
**BCL2**	6.2	2.1	-3.0	B-cell CLL/lymphoma 2
**POLG2**	13.5	4.7	-2.9	polymerase (DNA directed), gamma 2, accessory subunit
**DBT**	15.1	5.3	-2.9	dihydrolipoamide branched chain transacylase E2
**ACAD8**	8.7	3.1	-2.9	acyl-Coenzyme A dehydrogenase family, member 8
**HDDC2**	32.3	11.3	-2.8	HD domain containing 2
**PISD**	4.3	1.5	-2.8	phosphatidylserine decarboxylase
**TIMM44**	93.0	33.0	-2.8	translocase of inner mitochondrial membrane 44 homolog (yeast)
**GCAT**	8.2	2.9	-2.8	glycine C-acetyltransferase (2-amino-3-ketobutyrate coenzyme A ligase)
**KYNU**	1.0	0.4	-2.8	kynureninase (L-kynurenine hydrolase)
**ACOT9**	5.4	1.9	-2.8	acyl-CoA thioesterase 9
**FDX1**	5.5	2.0	-2.8	ferredoxin 1
**ACSM3**	0.8	0.3	-2.8	acyl-CoA synthetase medium-chain family member 3
**HADH**	58.0	21.2	-2.7	hydroxyacyl-Coenzyme A dehydrogenase
**ACSL4**	13.1	4.8	-2.7	acyl-CoA synthetase long-chain family member 4
**ISCU**	31.4	11.6	-2.7	IscU iron-sulfur cluster scaffold homolog (E. coli)
**NT5M**	11.4	4.2	-2.7	5',3'-nucleotidase, mitochondrial
**AGXT2**	1.2	0.4	-2.7	alanine-glyoxylate aminotransferase 2
**COMT**	88.3	33.7	-2.6	catechol-O-methyltransferase
**ECHDC3**	35.4	13.6	-2.6	enoyl Coenzyme A hydratase domain containing 3
**NUDT2**	9.0	3.5	-2.6	nudix (nucleoside diphosphate linked moiety X)-type motif 2
**SUPV3L1**	8.1	3.2	-2.6	suppressor of var1, 3-like 1 (S. cerevisiae)
**MPST**	35.0	13.7	-2.6	mercaptopyruvate sulfurtransferase
**MDH1**	112.7	44.2	-2.5	malate dehydrogenase 1, NAD (soluble)
**ALDH7A1**	33.3	13.2	-2.5	aldehyde dehydrogenase 7 family, member A1
**DHTKD1**	7.8	3.1	-2.5	dehydrogenase E1 and transketolase domain containing 1
**CPOX**	5.2	2.1	-2.5	coproporphyrinogen oxidase
**CYP24A1**	0.4	0.2	-2.5	cytochrome P450, family 24, subfamily A, polypeptide 1
**ADCK2**	6.5	2.6	-2.5	aarF domain containing kinase 2
**SLC25A15**	7.9	3.2	-2.5	solute carrier family 25 (mitochondrial carrier; ornithine transporter) member 15
**TST**	31.7	13.0	-2.4	thiosulfate sulfurtransferase (rhodanese)
**RILP**	2.1	0.9	-2.4	Rab interacting lysosomal protein
**SLC25A29**	2.8	1.2	-2.4	solute carrier family 25, member 29
**COX18**	2.4	1.0	-2.4	COX18 cytochrome c oxidase assembly homolog (S. cerevisiae)
**QDPR**	13.8	5.9	-2.3	quinoid dihydropteridine reductase
**NUDT19**	8.2	3.5	-2.3	nudix (nucleoside diphosphate linked moiety X)-type motif 19
**PCK2**	4.4	1.9	-2.3	phosphoenolpyruvate carboxykinase 2 (mitochondrial)
**SLC25A4**	24.7	10.7	-2.3	solute carrier family 25 (mitochondrial carrier; adenine nucleotide translocator), member 4
**MRPL15**	7.9	3.5	-2.3	mitochondrial ribosomal protein L15
**GNG5**	24.0	10.5	-2.3	guanine nucleotide binding protein (G protein), gamma 5
**IDH1**	130.9	57.3	-2.3	isocitrate dehydrogenase 1 (NADP+), soluble
**ABCB10**	4.3	1.9	-2.3	ATP-binding cassette, sub-family B (MDR/TAP), member 10
**THG1L**	6.9	3.1	-2.3	tRNA-histidine guanylyltransferase 1-like (S. cerevisiae)
**PARL**	15.2	6.8	-2.2	presenilin associated, rhomboid-like
**CA5B**	6.3	2.8	-2.2	carbonic anhydrase VB, mitochondrial

**RFK**	3.2	1.4	-2.2	riboflavin kinase
**FAHD1**	4.2	1.9	-2.2	fumarylacetoacetate hydrolase domain containing 1
**SERHL2**	28.1	12.8	-2.2	serine hydrolase-like 2
**NFS1**	9.9	4.5	-2.2	NFS1 nitrogen fixation 1 homolog (S. cerevisiae)
**GLRX5**	11.1	5.0	-2.2	glutaredoxin 5 homolog (S. cerevisiae)
**ZADH2**	9.0	4.1	-2.2	zinc binding alcohol dehydrogenase, domain containing 2
**ACADSB**	4.9	2.3	-2.2	acyl-Coenzyme A dehydrogenase, short/branched chain
**HSDL2**	26.5	12.2	-2.2	hydroxysteroid dehydrogenase like 2
**RAB8B**	10.2	4.7	-2.1	RAB8B, member RAS oncogene family
**NME2**	1960.6	912.7	-2.1	non-metastatic cells 2, protein (NM23B) expressed in
**CLIC4**	76.0	35.4	-2.1	chloride intracellular channel 4
**MRPL51**	14.6	6.8	-2.1	mitochondrial ribosomal protein L51
**BRP44L**	18.5	8.7	-2.1	brain protein 44-like
**SUCLG2**	5.4	2.5	-2.1	succinate-CoA ligase, GDP-forming, beta subunit
**ARMC1**	8.8	4.1	-2.1	armadillo repeat containing 1
**GFM2**	6.2	3.0	-2.1	G elongation factor, mitochondrial 2
**NME1**	4.6	2.2	-2.1	non-metastatic cells 1, protein (NM23A) expressed in
**GLDC**	23.3	11.1	-2.1	glycine dehydrogenase (decarboxylating)
**SLC25A36**	9.2	4.4	-2.1	solute carrier family 25, member 36
**PANK2**	16.2	7.8	-2.1	pantothenate kinase 2 (Hallervorden-Spatz syndrome)
**TOMM70A**	56.6	27.5	-2.1	translocase of outer mitochondrial membrane 70 homolog A (S. cerevisiae)
**MRPS6**	66.3	32.3	-2.1	mitochondrial ribosomal protein S6
**SLC30A6**	8.6	4.2	-2.0	solute carrier family 30 (zinc transporter), member 6
**LYRM2**	44.5	21.8	-2.0	LYR motif containing 2
**LDHA**	771.1	381.7	-2.0	lactate dehydrogenase A
**GLUD1**	25.0	12.4	-2.0	glutamate dehydrogenase 1
**RPL35A**	315.6	157.2	-2.0	ribosomal protein L35a
**CYP11A1**	0.6	0.3	-2.0	cytochrome P450, family 11, subfamily A, polypeptide 1
