# Supplementary material for: Differentiation State-Specific Mitochondrial Dynamic Regulatory Networks Are Revealed by Global Transcriptional Analysis of the Developing Chicken Lens
Source: G3 (Bethesda). 2014 Jun 13;4(8):1515–27. doi: 10.1534/g3.114.012120 (PMC4132181; doi:10.1534/g3.114.012120)
Supplement: Supporting Information [file supp_g3.114.012120_TableS10.pdf]

Table S10 Nuclear encoded mitochondrial protein transcript that demonstrated a two-fold increase in expression or greater during EQ to FP transition. Detected FPKM and fold change (Δ) shown.

Symbol	EQ	FP	Δ	Description
**SLC25A22**	0.8	32.5	42.5	solute carrier family 25 (mitochondrial carrier: glutamate), member 22
**SNPH**	0.4	14.6	40.8	syntaphilin
**ME1**	1.1	41.5	38.0	malic enzyme 1, NADP(+)-dependent, cytosolic
**GLRX**	20.8	469.8	22.6	glutaredoxin (thioltransferase)
**NIF3L1**	43.5	847.2	19.5	NIF3 NGG1 interacting factor 3-like 1 (S. pombe)
**RTN4IP1**	15.5	141.3	9.1	reticulon 4 interacting protein 1
**QRSL1**	7.5	53.8	7.2	glutaminyl-tRNA synthase (glutamine-hydrolyzing)-like 1
**MSRA**	10.1	71.2	7.1	methionine sulfoxide reductase A
**ME3**	8.1	55.3	6.9	malic enzyme 3, NADP(+)-dependent, mitochondrial
**TDRKH**	0.2	0.9	6.0	tudor and KH domain containing
**GLS**	12.7	63.9	5.0	glutaminase
**GPX1**	109.2	549.7	5.0	glutathione peroxidase 1
**ABCD2**	2.8	14.1	5.0	ATP-binding cassette, sub-family D (ALD), member 2
**MRPS7**	17.7	78.1	4.4	mitochondrial ribosomal protein S7
**SLC25A25**	3.9	16.5	4.3	solute carrier family 25 (mitochondrial carrier; phosphate carrier), member 25
**DMGDH**	0.1	0.6	4.2	dimethylglycine dehydrogenase
**DHRS1**	3.3	13.8	4.1	dehydrogenase/reductase (SDR family) member 1
**MLYCD**	3.4	13.9	4.1	malonyl-CoA decarboxylase
**AKAP10**	11.7	47.0	4.0	A kinase (PRKA) anchor protein 10
**ATPAF2**	10.5	42.0	4.0	ATP synthase mitochondrial F1 complex assembly factor 2
**AFG3L2**	11.4	45.6	4.0	AFG3 ATPase family gene 3-like 2 (yeast)
**DUSP26**	1.5	5.7	3.8	dual specificity phosphatase 26 (putative)
**HSD17B4**	9.1	33.8	3.7	hydroxysteroid (17-beta) dehydrogenase 4
**TMTC1**	0.4	1.5	3.6	transmembrane and tetratricopeptide repeat containing 1
**NDUFA12**	70.5	247.6	3.5	NADH dehydrogenase (ubiquinone) 1 alpha subcomplex, 12
**MRPS18C**	8.2	27.6	3.4	mitochondrial ribosomal protein S18C
**NDUFS5**	54.4	182.0	3.3	NADH dehydrogenase (ubiquinone) Fe-S protein 5, 15kDa (NADH-coenzyme Q reductase)
**NDUFB10**	126.7	424.3	3.3	NADH dehydrogenase (ubiquinone) 1 beta subcomplex, 10, 22kDa
**TRNT1**	37.6	125.4	3.3	tRNA nucleotidyl transferase, CCA-adding, 1
**NT5C3**	13.4	44.4	3.3	5'-nucleotidase, cytosolic III
**FDPS**	74.8	241.1	3.2	farnesyl diphosphate synthase (farnesyl pyrophosphate synthetase, dimethylallyltranstransferase, geranyltranstransferase)
**SFXN1**	58.0	184.2	3.2	sideroflexin 1
**ASAH2**	0.2	0.8	3.1	N-acylsphingosine amidohydrolase (non-lysosomal ceramidase) 2
**GATM**	0.1	0.3	3.1	glycine amidinotransferase (L-arginine:glycine amidinotransferase)
**RDH14**	3.5	10.4	3.0	retinol dehydrogenase 14 (all-trans/9-cis/11-cis)
**MTIF3**	27.1	77.9	2.9	mitochondrial translational initiation factor 3
**CLPX**	17.5	49.2	2.8	ClpX caseinolytic peptidase X homolog (E. coli)
**ALAS1**	11.8	33.2	2.8	aminolevulinate, delta-, synthase 1

**PDHX**	3.4	9.3	2.7	pyruvate dehydrogenase complex, component X
**SOD1**	108.1	282.2	2.6	superoxide dismutase 1, soluble (amyotrophic lateral sclerosis 1 (adult))
**SFXN2**	9.9	25.7	2.6	sideroflexin 2
**RAB11A**	24.6	63.4	2.6	RAB11A, member RAS oncogene family
**DNAJC15**	11.3	29.3	2.6	DnaJ (Hsp40) homolog, subfamily C, member 15
**MRPL2**	23.8	60.7	2.5	mitochondrial ribosomal protein L2
**TFB1M**	6.2	15.8	2.5	transcription factor B1, mitochondrial
**TIMM23**	28.3	70.0	2.5	translocase of inner mitochondrial membrane 23 homolog (yeast)
**SLC25A43**	13.1	32.3	2.5	solute carrier family 25, member 43
**PMPCA**	29.1	69.4	2.4	peptidase (mitochondrial processing) alpha
**HADHA**	27.2	63.4	2.3	hydroxyacyl-Coenzyme A dehydrogenase/3-ketoacyl-Coenzyme A thiolase/enoyl-Coenzyme A hydratase (trifunctional protein), alpha subunit
**NEU4**	5.2	11.9	2.3	sialidase 4
**COQ2**	2.1	4.7	2.3	coenzyme Q2 homolog, prenyltransferase (yeast)
**SLC25A10**	63.9	143.2	2.2	solute carrier family 25 (mitochondrial carrier; dicarboxylate transporter), member 10
**NDUFA2**	116.0	256.3	2.2	NADH dehydrogenase (ubiquinone) 1 alpha subcomplex, 2, 8kDa
**LARS2**	9.7	21.4	2.2	leucyl-tRNA synthetase 2, mitochondrial
**TRIAP1**	16.4	35.7	2.2	TP53 regulated inhibitor of apoptosis 1
**GARS**	28.2	60.4	2.1	glycyl-tRNA synthetase
**DACT2**	11.3	24.1	2.1	dapper, antagonist of beta-catenin, homolog 2 (Xenopus laevis)
**DNAJA3**	20.8	44.3	2.1	DnaJ (Hsp40) homolog, subfamily A, member 3
**CPS1**	0.5	1.1	2.1	carbamoyl-phosphate synthetase 1, mitochondrial
**TBRG4**	14.6	30.4	2.1	transforming growth factor beta regulator 4
**COQ4**	15.6	32.5	2.1	coenzyme Q4 homolog (S. cerevisiae)
**NDUFS8**	104.8	210.3	2.0	NADH dehydrogenase (ubiquinone) Fe-S protein 8, 23kDa (NADH-coenzyme Q reductase)
**SLC25A38**	3.4	6.7	2.0	solute carrier family 25, member 37
**ATPAF1**	10.0	20.0	2.0	ATP synthase mitochondrial F1 complex assembly factor 1
